# One model for the learning of language

**DOI:** 10.1073/pnas.2021865119

**Published:** 2022-01-24

**Authors:** Yuan Yang, Steven T. Piantadosi

**Affiliations:** ^a^College of Computing, Georgia Institute of Technology, Atlanta, GA 30332;; ^b^Department of Psychology, Helen Wills Neuroscience Institute, University of California, Berkeley, CA 94720

**Keywords:** computational linguistics, learning theory, program induction, formal language theory

## Abstract

A major goal of linguistics and cognitive science is to understand what class of learning systems can acquire natural language. Until recently, the computational requirements of language have been used to argue that learning is impossible without a highly constrained hypothesis space. Here, we describe a learning system that is maximally unconstrained, operating over the space of all computations, and is able to acquire many of the key structures present in natural language from positive evidence alone. We demonstrate this by providing the same learning model with data from 74 distinct formal languages which have been argued to capture key features of language, have been studied in experimental work, or come from an interesting complexity class. The model is able to successfully induce the latent system generating the observed strings from small amounts of evidence in almost all cases, including for regular (e.g., *a^n^*, ${\left(ab\right)}^{n}$, and ${\left\{a,b\right\}}^{+}$), context-free (e.g., $a^{n}b^{n},\;a^{n}b^{n+m}$, and $xx^{R}$), and context-sensitive (e.g., $a^{n}b^{n}c^{n},\;a^{n}b^{m}c^{n}d^{m}$, and *xx*) languages, as well as for many languages studied in learning experiments. These results show that relatively small amounts of positive evidence can support learning of rich classes of generative computations over structures. The model provides an idealized learning setup upon which additional cognitive constraints and biases can be formalized.

## Significance

It has long been hypothesized that language acquisition may be impossible without innate knowledge of the structures that occur in natural language. Here, we show that a domain general learning setup, originally developed in cognitive psychology to model rule learning, is able to acquire key pieces of natural language from relatively few examples of sentences. This develops a new approach to formalizing linguistic learning and highlights some features of language and language acquisition that may arise from general cognitive processes.

One of the central debates in language acquisition is whether the structures of natural language are genetically specified or learned through experience. A key tool in this debate has been the use of formal mathematical analysis to determine what learners could or could not logically induce from the type of data they observe. Perhaps the most influential formal result is Gold’s Theorem (1), which implies that there are classes of even regular languages (2) which contain members that cannot be identified by learners who rely on positive examples alone. Gold’s result has been interpreted to mean that human children could not succeed in learning natural language without substantially informative innate constraints, following arguments from Chomsky (3). Gold’s result gave rise to detailed formal theories of learning under similar assumptions (4–8). Learnability proofs for certain classes of grammars have also been formulated (9–16), as well as closely related theories of induction in computer science (17–22).

However, Gold’s negative learnability result is not widely accepted as relevant to human language (13, 23, 24). Gold relied on a worst-case analysis, which assumes that a parent would intentionally mislead a child through an unbounded number of incorrect hypotheses if they can. This made analysis tractable using his formal tools, but, critically, was disconnected from naturalistic parent–child interactions. In situations where some of Gold’s assumptions are altered, learnability can be established under less antagonistic assumptions (22, 25–27). A striking, recent result shows that languages can be learned using positive evidence alone, out of the maximally unconstrained space of all possible computations (28). This more optimistic analysis involves several critically different assumptions. For one, it assumes that sentences are sampled from a distribution, meaning that it uses an average-case analysis rather than worst case; it also quantifies learning through how well a learner could predict future strings. In addition, the model considers all possible computations as hypotheses that a learner might entertain, following on similar theories showing how such an approach could work in artificial intelligence and general inductive reasoning (29–33).

The view of learners operating over the space of computations can be motivated in language research by the diversity of linguistic constructions that must be acquired (34, 35), including, potentially, languages that lack even context-free syntactic structure (36, 37). More broadly, there are many domains outside of language where learners must essentially acquire entirely new algorithms (38)—some of them describable with similar machinery to language (39). It is ordinary for children to come to know new computational processes in learning tasks like driving, cooking, programming, or playing games. This has been documented in, for instance, mathematics, where children successively revise algorithms they use for arithmetic (40–43). Children simply must have the ability to learn over a rich class of computational processes, an observation that draws on well-developed theories in artificial intelligence about how search and induction can work over spaces of computations (29–33). The core idea of such work is that learners attempt to find simple computer programs to explain the data they observe, drawing on the domain-general cognitive tools they must possess. Learners, in this view, are much like scientists (44) who look at data and construct computational theories in order to explain the patterns that they observe; indeed, the approach builds on similar efforts to automate scientific discovery (45–47).

While the above work addresses learnability as a mathematical question, the central ideas remain relatively unconnected to contemporary ideas about mental representations. First, most theoretical analyses do not provide working implementations. Second, the representations prior analyses have used tended to focus on Turing machines or treated languages as abstract sets, but philosophers, cognitive scientists, and linguists have made a convincing case that compositional representations are natural for capturing human-like thinking (48–52) and computation itself (53–56). Indeed, theories of inductive inference centered on compositionality provide good fits to human learning curves across domains (57–61). Third, prior work has not targeted many of the specific computations that are thought to be important for natural language. Here, we address these problems by providing a learning model that uses components which have been independently argued for in concept learning experiments. We show how a compositional model can learn key generative pieces of natural language, including many that motivated classic theories of linguistic representation. We begin by considering simple linguistic patterns that inaugurated both modern linguistics and modern computer science.

Our starting point—and the inspiration for our title—is Chomsky’s “Three models for the description of language” (62) which noted that many dependencies in natural language could be captured abstractly with distinct kinds of computational devices (63–65). Some devices require a finite amount of memory (e.g., finite-state machines), some require an unbounded amount of memory in a stack, and some require even more powerful systems. Chomsky’s work, in collaboration with Marcel-Paul Schützenberger, charted out different kinds of string sets and classified what classes of devices could compute each. Following this literature, we use the term “formal language” to refer to a set of strings, typically one that is generated according to a simple computational pattern.

For example, the tail recursion allowed by English adjectives (“The adorable, friendly, young monkey”) mirrors the structure in the simple formal language $a^{*}=\{\epsilon ,a,aa,\mathrm{aaa},\ldots \}$, where zero (*ϵ*) or more successive adjectives (*a*) could be concatenated into a valid substring of English. In turn, this can be captured with a computational device with one internal state, which may optionally emit an *a* and return to the same state. Dependencies between determiner–noun pairs (“Bring me two boats, three accordions, and six babies.”) mirrors the formal language ${\left(ab\right)}^{+}$ where each determiner (*a*) requires a corresponding noun (*b*). The formal language $\left\{a^{n}b^{n}:n=1,2,\ldots \}=\{ab,\mathrm{aabb},\mathrm{aaabbb},\ldots \right\}$ might characterize the key dependencies in English “if–then” relationships. Specifically, every “if” (an “a”) must be followed by a “then” (a “b”), as in “If Mary cried then John was sad then John cares about Mary” (63).* This formal language cannot be generated or recognized by a computational device with a finite number of states but can be captured by a context-free grammar. Although the applicability of such examples to natural language is often contested (66), the study of such formal languages has provided a fertile ground for linguistics (67) to characterize what computations underlie human language (2, 39, 62, 63). A considerable amount of effort has gone toward understanding whether natural language can be captured entirely by context-free grammars or other systems (66, 68–70), or how non–context-free aspects may be handled (71), although many arguments that have been made are inadequate technically (72).

Here, we study a variety of formal languages which pose many of the key challenges that have attracted attention in learning theory, including the underdetermination of grammars by evidence, the “subset problem” of how learners appropriately constrain their generalizations, and the puzzle of how learners come to know productive generative processes. As we show, these can all be resolved by formalizing proper statistical inference over a space of computations. We apply our model to a variety of test cases spanning simple formal languages, versions of stimuli from experimental work, and a simple English grammar.

## Formal Model

Following a growing body of work in compositional Bayesian models (38, 58, 59, 61, 73–88), we assume that the representations learners must discover are built by combining primitives in a language of thought (LOT) (49) to form the mental analog of programs. In this setup, learners observe data (here, strings) and compare hypotheses that are built out of primitives, as a way to explain the data, much as scientists might consider possible physical laws which are compositions of mathematical operations (e.g., $F=G\cdot m_{1}\cdot m_{2}/r^{2}$). The specific primitives that we assume are motivated by minimalist functional programming languages like Scheme (54) which try to build in as little as is practical while remaining able to express all computations.

Table 1 lists the types of operations we consider, many of which are meant to be domain-general primitives that can be—and have been—deployed in other areas of concept learning. The first kind are list/string operations, pair, first, and rest, which build and manipulate sequences of characters from the alphabet. The functions pair and append are similar in spirit to “merge” in minimalist linguistics (89, 90), except they come with none of the associated machinery that is required in those theories; here, they only concatenate. The function insert puts one string in the middle of another (e.g., insert(`abcd',`efg') yields `abefgcd'), which allows concise construction of long-range dependencies because it permits dependent elements to be generated consecutively (e.g., *abcd*) and then displaced (e.g., ab…cd). The assumed Boolean operators include common logical connectives and conditionals. Both flip and sample are notable in that they are stochastic, allowing nondeterminism in the program (91, 92), an ability that is possibly itself useful for organisms with finite memory (93). We note that the classical Chomsky hierarchy no longer directly applies to formal languages in this probabilistic setting (94, 95). Finally, we allow a computation to call another function F1, F2, etc. (potentially itself). This call may be memoized, which means that it remembers any stochastic choices that were made on a previous call with the same arguments (91). This use of recursion is meant to be domain general, as humans deal with recursion outside of syntax, like pragmatics (96), and even outside language (97–99). We note the similarity between these primitives and others used in modeling human conceptual systems (59, 61, 73, 74, 76, 78, 91, 100).

**Table 1.** ** **Assumed primitive functions

Primitive	Description
Functions on lists (strings)	
pair(L,C)	Concatenates character *C* onto list *L*
first(L)	Return the first character of *L*
rest(L)	Return everything except the first character of *L*
insert(X,Y)	Insert list *X* into the middle of *Y*
append(X,Y)	Append lists *X* and *Y*
Logical functions	
flip(p)	Returns true with probability *p*
equals(X,Y)	True if string *X* is the same string as *Y*
empty(X)	True if string *X* is empty; otherwise, false
if(B,X,Y)	Return *X* if *B* else return *Y* (*X* and *Y* may be lists, sets, or probabilities)
and, or, not	Standard Boolean connectives (with short circuit evaluation)
Set functions	
Σ	The set of alphabet symbols
{s}	A set consisting of a single string
union(set,set)	Union of twos sets
setminus(set,s)	Remove a string from a set
sample(set)	Sample from *s* of strings
Strings and characters	
*ϵ*	Empty string symbol
*x*	The argument to the function
‘a’, ‘b’, ‘c’, …	Alphabet characters(language specific)
Function calls	
Fi(z), Fmi(z)	Calls factor *Fi* with argument *z*; the *Fmi* version memoizes probabilistic choices (see text)

The space of hypotheses consists of all compositions of these functions that respect the input and output types.

Valid compositions of primitives—those that respect the input and output types of each function—define an infinite set of hypotheses for learners to consider. For instance, the hypothesis

F0(x)≔pair(if(flip(1/3),ϵ,F0(ϵ)),a)

concatenates (pairs) an “a” with either an empty string (*ϵ*) or the outcome of calling itself with an empty string as an argument, F0(ϵ). When called with the default argument of an empty string (i.e., x=ϵ), F0 generates the set of strings $\left\{a,aa,\mathrm{aaa},\mathrm{aaaa},\ldots \}=\{a^{n}:n=1,2,3,\ldots \right\}$. Importantly, this function also gives a probability distribution over strings through its use of flip. Here, the distribution is geometric, meaning that the length of each output is determined by the number of times a 1/3-weighted coin can be flipped before getting heads, giving $P(a^{n})={\left(1-\frac{1}{3}\right)}^{n-1}\cdot 1/3$.

If we define *H* to be the space of all functions that can be constructed by composing the operations in Table 1 and define a variable *D* to be a multiset of observed strings, an idealized learner will compute a posterior distribution on hypotheses P(H|D) via Bayes rule P(H|D)∝P(H)·P(D|H). Here, *P*(*H*) is given by a probabilistic context-free grammar (PFG) on the operations in Table 1, which effectively penalizes complexity. Thus, *P*(*H*) implements a simplicity preference just as in induction of Turing machines (28–31), although the question of precisely which simplicity measure is psychologically accurate is an empirical one (63), and one which has been examined in closely related domains (59). P(D|H) is a likelihood specifying how likely the observed strings are to be generated by *H*, as discussed in the next section.

## Technical Innovations

Our implementation includes several important technical innovations that allow it to be scaled to interesting classes of formal languages. First, the choice of likelihood function P(D|H) is chosen to allow incremental improvements to hypotheses. In its simplest form, we might just run the program *H* and use its output set of strings. The problem with this is that it does not assign any partial credit to hypotheses which get most pieces of a string correct. For instance, if a hypothesis generates the strings {a,aa,aaa,…}, this likelihood would assign zero probability to observed data {ab,aab,aaab,…} even though most characters in most strings were produced. To address this, we apply a “prefix likelihood” which assumes a noise process that may delete and then append on the end of a string in a stochastic manner where each deletion or generation happens with a fixed probability. Thus, if a hypothesis generates *aaa*, it will assign *aaab* a nonzero likelihood equal to the probability of appending one *b*.

Second, we allow the possibility that hypotheses involve multiple LOT expressions (101), here called “factors” F0, F1, F2, etc. For example, a hypothesis might be

$$\begin{array}{ll} \mathrm{F0}(x) & ≔\mathrm{pair}(\mathrm{if}(\mathrm{flip}(1/3),\epsilon ,\mathrm{F0}(\epsilon )),a).\\ \mathrm{F1}(x) & ≔\mathrm{if}(\mathrm{empty}(x),\\ & \;\;\epsilon ,\\ & \;\;\mathrm{append}(\mathrm{pair}(\epsilon ,\mathrm{first}(x)),\mathrm{pair}(\mathrm{F1}(\mathrm{rest}(x)),b))).\\ \mathrm{F2}(x) & ≔\mathrm{F1}(\mathrm{F0}(\epsilon )) \end{array}$$

The first function, F0, is the *a^n^* formal language shown above. The second function, F1, takes an argument *x*, and recursively concatenates the first element of *x* with a recursive call to itself, followed by a *b*. Because this is recursive on a shortening string (rest(x)), one *b* will be added for each element of *x*. For instance, if we called F2 on the string *xyz*, it would return the string *xyzbbb*. Finally, F2 puts F0 and F1 together. Thus, the strings *a^n^* generated by F0 will be passed to F1, which will attach a single *b* for each *a*. This therefore generates strings of the form $a^{n}b^{n}$ (although it is not the simplest way). Allowing for multiple factors, in conjunction with the likelihood, allows for complex computations to be learned via individual pieces which are more manageable.

As described above, the expressions we evaluate may be nondeterministic. This provides a challenge for evaluating the set of strings that a given expression generates. Our implementation handles randomness by following multiple possible execution paths when a random primitive is encountered, enumerating the possible paths greedily in order of their probability, an idea drawing on techniques for evaluation of probabilistic programs more generally (102). We then compute the overall distribution of output strings, marginalizing approximately over execution paths. We use stochastic sampling methods to find high posterior probability hypotheses (see *Methods*). To do this, we developed a C++ library called Fleet (distributed under the GNU Public License v3 at https://github.com/piantado/Fleet). Notably, the Fleet implementation includes examples in other domains outside language, such as number learning (73) and logical rule induction (58), demonstrating the generality of the approach.

## Results

We first study the model’s ability to learn probabilistic versions of several formal languages which have been motivated by the structures present in natural language. We choose the targets of learning primarily following examples from reviews and theoretical pieces (67, 72, 103), while adding a number of other interesting examples. Our intention is not to engage the debate about which examples correspond to natural language; rather, we use these to motivate the range of formal systems that a learner is likely able to acquire. In all cases, we provide the learning model with positive examples only of the formal language. Moreover, for all formal languages and sections below, the inferential setup is identical between these languages—the only things that change are the data provided, the alphabet of symbols the model is expected to work over, and the amount of run time.

We visualize results by approximating the posterior model-average precision and recall for the most likely strings generated by each hypothesis (see *Methods*). When precision is high but recall is low, the model undergeneralizes (the strings it generates are all in the target formal language, but it does not capture all of them). Conversely, when recall is high and precision is low, the model overgeneralizes. When precision and recall are both 1.0, that means that the model has successfully learned the target formal language up to the approximation used to compute precision and recall. We also include a gray “Memorized (F)” line corresponding to what F score would be achieved by simply memorizing the observed data.

### Learning Simple Formal Languages.

**Fig. 1.**
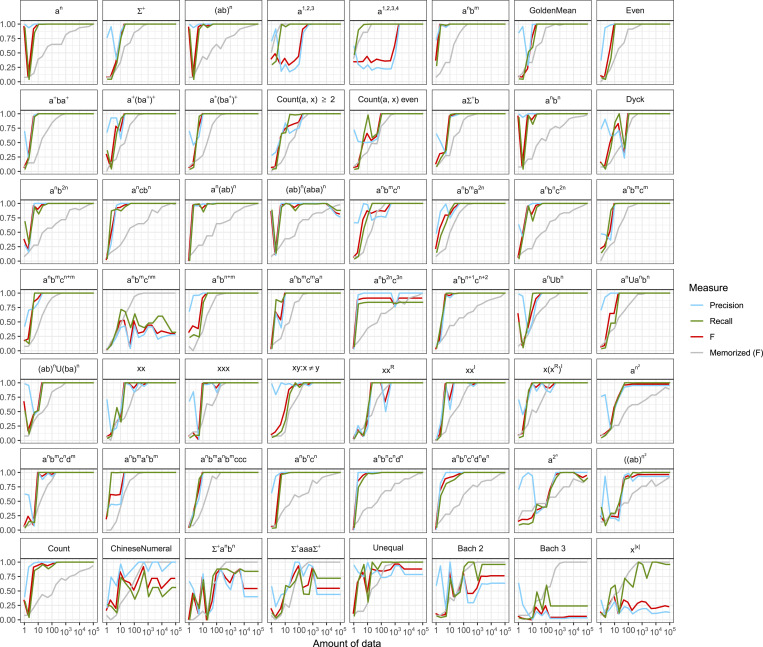
Learning curves for the model, provided with data from each target formal language. Precision (blue), Recall (green), and F score (red) are for the learning model, while Memorized (F) (gray) gives the F score for a model that only memorized the training data. Here, we have adopted the convention that Σ={a,b}, n,m∈N, *Count*(*v*,  *x*) counts the number of times the character *v* occurs in string *x*, and |w| is the number of characters in a string *w*. The *x* axis is the number of sampled tokens from the target formal language (note the logarithmic scale).

Fig. 1 first shows learning curves for 56 different simple formal languages. Each plot shows the estimated precision and recall (*y* axis) as a function of the amount of data provided to the model (*x* axis) (see *Methods*). In general, determining equivalence of hypotheses is uncomputable, so our precision and recall measures provide a quantification of how well the learner has acquired the target language, but we caution that, in all our figures, the results must be interpreted with care because these values are approximate.

#### The model is able to learn many types of formal languages.

Fig. 1 shows that the implemented model is able to learn programs for formal languages that vary in computational complexity, including regular (e.g., *a^n^*, ${\left(ab\right)}^{n}$, and $\Sigma^{+}$), context-free (e.g., $a^{n}b^{n},\;a^{n}b^{n+m}$, and $a^{n}b^{2n}$), and context-sensitive (e.g., $a^{n}b^{n}c^{n},\;a^{n}b^{n}c^{2n}$, and $a^{n}b^{n+1}c^{n+2}$). It succeeds on the Dyck language, consisting of balanced sequences of parentheses—valid parse trees—and the “mirror” language $xx^{R}$. Both are context-free, and versions of them have been examined in recent experimental work (104, 105). The model’s success across such diverse formal languages shows that techniques for program induction can work across levels of computational complexity.

Researchers have also explored formal languages that can be captured with other formalisms, including linear indexed grammars (106), tree-adjoining grammars (107) or combinatory categorial grammars (50). Formal languages relevant to these additional classes are learnable, such as $a^{n}b^{n}c^{n}d^{n}$. Several other interesting examples are also shown (GoldenMean, Count(a,x)≥2, *Count*(*a*,  *x*) is even, $a^{+}ba^{+},\;(a^{*}){\left(ba^{*}\right)}^{+},\;(a^{+}){\left(ba^{+}\right)}^{+}$), including those which distinguish other classes (103).^†^ The formal language $a^{n}b^{m}a^{n}b^{m}ccc$ is an example from ref. 64; the ChineseNumeral language $\left\{ab^{n_{1}}ab^{n_{2}}\ldots ab^{n_{k}}:n_{i}>n_{n+1}\right\}$ has been discussed previously as a model of numerals in Chinese (109, 110) and a motivating example for range concatenation grammars. This formal language is only learned approximately. The model learns $a^{n}b^{m}c^{n}d^{m}$, which was motivated by crossed-serial dependencies in natural language (69, 111–113). The formal language $xx=\{xx:x\in \Sigma^{+}\}$ is motivated by “respectively” examples (114, 115) where lists must be paired up 1–1, as in “Bob, Pietro, and Johnny are a guitarist, accordionist, and troubadour, respectively” as well as examples from Mohawk’s use of noun stems in verbs (116). Another interesting example is the Count language {ab,ababb,ababbabbb,…}, which shows that other computations—perhaps unlike those needed in natural language—can be inferred in the same setup.

#### The hypotheses the model constructs reflect importantly different underlying processes.

Examination of the specific hypotheses that the model constructs shows that it develops a generative model of the data, rather than simply memorizing patterns it sees. To illustrate a few examples, the model learns to construct a system equivalent to a context-free grammar when given strings from $a^{n}b^{n}$,

F0(x)≔append(pair(ϵ,a),pair(if(flip(1/3),x,F0(ϵ)),b)).

This puts an *a* at the start of a string, puts a *b* at the end, and flips a coin for whether to add a recursive call in between the two. Not only does this generate the right set of strings {ab,aabb,aaabbb,…}, it does so with the correct probabilities. Moreover, following the trace of recursive calls to F0 in generating a string reveals that the execution path of this program is tantamount to a parse tree,

[1] [a b],   [a [a b] b],   [a [a [a b] b] b],   … .

In cases like this, the learner acquires a structure matching what a grammar would generate, although note that, in general, our evaluation metric evaluates only the generated string set, not its latent structure. The model discovers a clever and nonobvious way to express $a^{n}b^{n}c^{n}$:

$$\begin{array}{l} \mathrm{F0}(x)≔\mathrm{append}(\mathrm{pair}(\epsilon ,a),\mathrm{pair}(\mathrm{if}(\mathrm{flip}(1/3),\mathrm{pair}(x,b),\\ \;\;\mathrm{Fm0}(\mathrm{pair}(x,b))),c)). \end{array}$$

*SI Appendix* contains listings of the top hypotheses found at the end of learning for each of the languages tested, as well as the observed number of data tokens. In most cases, the best hypotheses correspond to ones that can be seen to correctly compute each language, or come very close, showing that the model is genuinely discovering appropriate generative processes.

#### Learning takes little data, and the model does more than memorize.

Note that, in most cases, the amount of data required for learning to succeed is surprisingly small—often within less than 10 sentence tokens sampled from the target language. This accords with the small estimates of the total information required for syntax (117) and highlights that, even though the hypothesis space is large, that doesn’t mean that huge amounts of data will be required. Intuitively, since each observed string is unlikely under most hypotheses, only a few strings are enough to reduce the set of likely hypotheses to a manageable number. Moreover, in most cases where the model is able to learn, it learns much faster than a model which simply memorized the data, as shown by the model F score (red) generally being above the memorized F score (gray). For example, the model acquires a nearly perfect F score on $a^{n}b^{n}c^{n}$ after about 10 tokens, but it takes around 10^5^ tokens for memorization to achieve a similar level. We note that the slow speed of memorization results from how long it takes to sample the top 25 strings under the assumed distribution on string lengths. This comparison highlights that the model generalizes far beyond the data it has seen.

#### The model eagerly generalizes finite data to infinite string sets.

An interesting comparison can be made between the formal language *a^n^* and $a^{1,2,3}=\{a,aa,\mathrm{aaa}\}$. The latter is a subset of the former, but it has a finite cardinality. One of the most striking properties of natural language is that English grammar seems to permit arbitrarily long sentences (cf. ref. 118). This fact might be considered to be a core aspect of our innate linguistic endowment (119, 120) or a consequence of evolving a communication system with many signals (121). Alternatively, learners might even consider as statistical hypotheses that language was finite or infinite, as suggested by the purportedly finite languages in existence like Pirahã (36, 37). It might seem that there could be no data to show a learner that their language was infinite because that hypothesis necessarily goes beyond what has been observed. Indeed, infinite generalization is contrary to subset-principle accounts (6, 122–124) that posit learners make only the narrowest generalization possible from data (cf. refs. 125 and 126). Here, however, the issue comes down to whether finite or infinite languages are easier to express. The model shows that *a^n^* is easier to learn than $a^{1,2,3}$ or $a^{1,2,3,4}$, and this is because the infinite language has a simpler description, matching prior theoretical analysis (127). The model’s success in learning infinitely productive generative systems of rules can be contrasted with claims in generative linguistic textbooks. For instance, one states that “a productive system like the rules of Language probably could not be learned or acquired. Infinite systems are in principle, given certain assumptions, both unlearnable and unacquirable” (128).

We note that all of the learning results use data which are sampled from the target formal language, typically using geometrically distributed string lengths. In principle, however, this kind of model allows us to examine how many—or which—individual data points at different embedding depths license learners to infer that the language is infinite. Detailed investigation of these kinds of learning patterns should help to refine debates about the possible role of rare data in natural language acquisition, as even a few sentences can lead to unbounded productivity.

#### Not all formal languages are easily learned, even though the model is Turing complete.

Finally, it is often argued that models which have a capacity to learn any formal language are inappropriate for human language because humans appear unwilling to learn some patterns. But, even for unconstrained models like this one, the limitations of inference, the strength of priors, and the informativity of data make some languages effectively unlearnable. For instance, this model has difficulty with the Bach 3 formal language, which consists of all strings in ${\left\{a,b,c\right\}}^{+}$ with an equal number of *a*s, *b*s, and *c*s (129). This language might be more easily learnable with other operations like “scrambling” (130) but is difficult to express with our assumed primitives. The formal language $x^{\left|x\right|}$ isn’t learned, and others like $a^{2^{n}}$ can be learned only approximately even with large amounts of data. Which languages are difficult to learn is determined by a subtle combination of the available primitives, the assumed inferential biases, and the way in which the data distinguish close alternatives.

A related consideration in linguistics is whether learning models can acquire patterns which are not typologically attested. Of course, any model like this that operates over an infinite hypothesis space must acquire unattested languages, since there are only finitely many attested languages. Importantly, however, we do not take the model as making typological predictions, mainly because there are many other pressures that factor into the form of languages beyond structural biases, including considerations of communicative usefulness (131, 132). To illustrate, the formal language computed by if(flip(),a,b) is high probability in the prior (since it is short) and would be easy for the model to learn. But this language only contains two symbols and therefore cannot be used to communicate much information. The model does not predict that such small languages with just two sentences should exist in the natural sample of languages. Instead, it claims that people could learn a two-sentence formal language if given enough input consistent with it.

### Connections to Artificial Language Learning.

We next study simplified versions of languages that have been examined in artificial language learning studies. Experimental work has characterized specific biases and limitations in real biological learners, and it is not our intention to show how these can be built into the model. Instead, our goal is to articulate a coherent computational-level (133) framework for learning onto which one can later add specific limitations and abilities documented in empirical studies.

**Fig. 2.**
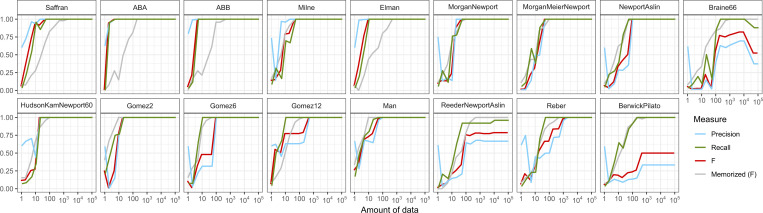
Learning curves for the model when run on examples from artificial language learning, including the Finite State grammar from Reber (149), a version of word segmentation motivated by Saffran et al. (134), “ABA” and “ABB” grammars from Marcus et al. (139), context-free grammars from Newport and coworkers (141, 146), a grammar from Morgan et al. (142), a finite set of strings consisting of all valid English words with “m,” “a,” and” n” (e.g., “man, am, an, a, mam”), a finite state machine for English auxiliaries from Berwick and Pilato (150), the QAXB grammar from Reeder et al. (154), a grammar modified from Hudson Kam and Newport (219), a finite language from Braine (147), a version of lexical learning from ref. 190, and string sets analogous to Gómez (144).

One of the most well-known artificial language paradigms, by Saffran et al. (134, 135), examines the ability of adults and children to segment a continuous speech stream into words. We provided the learning model with data modeled after ref. 134, with the primary change that the distribution of string lengths was geometric. This was necessary in order to avoid having to deal with hypotheses that generate infinite sequences (which are possible, although more challenging technically), and also may better match the observation that utterance boundaries provide an important cue in segmentation (136). Results are shown in Fig. 2 and illustrate that the model is able to create representations of the regularities in Saffran et al.’s stimuli. Notably, even though the model, like people, is provided only with an unsegmented stream of syllables, it is able to construct the appropriate recursive calls to generate these syllables as a stream of words. For instance, it learns to generate a word “tapiro” by constructing an internal structure pair(pair(pair(ϵ,ta),pi),ro). The model succeeds even though it is not told to search for words, nor is it given special cues like transitional probabilities. Words are learned simply as an efficient way of statistically “compressing” the observed data, in line with, for example, ref. 137. Such success shows how processes like segmentation, lexical learning, and syntactic category learning may all fall under the same umbrella (138).

Marcus et al. (139) studied infants’ ability to learn abstract variables in the form of languages that followed an “ABA” pattern (e.g., do–re-do) vs. an “ABB” pattern (e.g., do–re-re). The relevant feature of these languages is that “A” and “B” are variables which get realized as specific syllables. Infants’ success in learning these patterns suggests that the role of variables may be more broadly central to cognition (78, 140). Fig. 2 shows that the learning model is capable of learning these patterns by using the appropriate combination of function calls and variables. The representation learned in this case is

$$\begin{array}{l} \mathrm{F0}(x)≔\mathrm{append}(\mathrm{append}(\mathrm{sample}(\Sigma ),x),x).\\ \mathrm{F1}(x)≔\mathrm{Fm0}(\mathrm{sample}(\Sigma )). \end{array}$$

This hypothesis explicitly captures the ABB pattern in its structure.^‡^

**Fig. 3.**
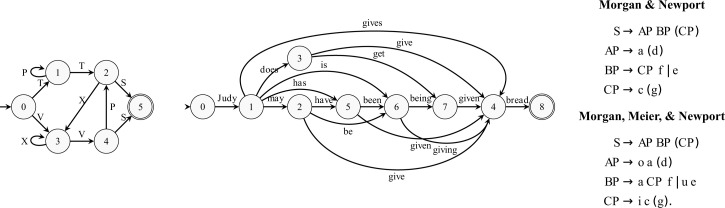
Finite state machines from Reber (149) (*Left*) and Berwick and Pilato (150) (*Center*). Words in this latter machine were converted to single characters to simplify the learning model. Grammars are from Morgan and Newport (141) and Morgan et al. (142) (*Right*).

“Morgan & Newport” (141) follows simple phrase structure grammar in Fig. 3, and has been examined in several empirical studies (142, 143). In this grammar, elements in parentheses are optional. “Morgan, Meier, & Newport” is a version from ref. 142 with “function words” (*o*, *a*, *u*, *i*) that mark the syntactic grouping and speed learning. Fig. 2 shows that both versions of the language are learnable with very little data (∼100 tokens), although note that, despite being presented as grammars, these are finite languages and are not learned faster than memorization. Work by Gómez (144) and Gómez and Maye (145) has examined languages of the form *aXb* where the number of possible *X* elements varied. In Fig. 2, several “Gómez” curves are shown, corresponding to languages with varying cardinality of 2, 6, and 12. As this makes clear, these languages are learnable; similarly, the model is also able to learn the nonadjacent dependencies studied by Newport and Aslin (146), consisting of sentences of the form {bXt,gXd,pXr,kXu,lXi} where *X* ranges over {1,2,3,4}. The model has some difficulty learning the simple finite grammar from Braine (147, 148) that mimicked some properties of serial order and phrase structure rules.

Fig. 2 also shows learning curves for finite state languages examined by Reber (149) and Berwick and Pilato (150), which are described in Fig. 3. These languages are interesting, in part, because they are fairly complex in terms of description length, yet are simple—finite state—in computational complexity. Reber’s was constructed to provide a learnable but nontrivial set of strings, and both it and similar languages have been modeled with neural networks (151, 152), as well as models based on chunking (153). Berwick and Pilato’s captures the English auxiliary system, and the model can only approximate it, likely due to the complexity of this language and the model’s limited inference scheme.

Reeder et al. (154, table 2) is an interesting case where some strings are held out from the language (and the human training). Learners and the model are given a “QAXB” pattern and then tested on unseen strings. This means that, for the model to perform well, it should exhibit a high recall (correctly reproducing the test strings) and a lower precision (generating strings outside of the training set). It does this, and generates, for instance, many strings like “scb” and “axB” which are not in the training set but do fit the intended pattern of the training data.

Overall, these results demonstrate that the model is capable of learning many of the patterns examined in experimental work. We expect that the model will not provide a tight fit to the details of human behavior in such tasks without incorporating additional cognitive considerations, including memory limits and perceptual salience.

### Toward Natural Language Grammars.

The problem faced in natural syntax is not just a series of unrelated, simple languages but that of learning a language that combines many patterns simultaneously. We next examine an English-like grammar that one might find in an introductory linguistic textbook,

[2] $$\begin{array}{ll} S & \to NP\;VP\\ NP & \to n|d\;n|d\;AP\;n|NP\;PP\\ AP & \to a|a\;AP\\ VP & \to v|v\;NP|v\;t\;S|VP\;PP\\ PP & \to p\;NP \end{array}.$$

This target grammar includes several syntactic structures of English, including multiple expansions of a nonterminal type (e.g., NP), tail recursion (in AP), transitive and intransitive verbs (in VP), sentential embedding (in VP), and prepositional phrases with embedded noun phrases. For instance, one string the model might observe is “d a n p d a n v t n v,” which corresponds to the part-of-speech sequence for a sentence like “The delightful accordionist by the old cafe knew that music heals.” As with all datasets, the model is not told there is a (e.g., context-free) grammar to learn, but is only told to construct something out of its primitives to explain the observed strings.

**Fig. 4.**
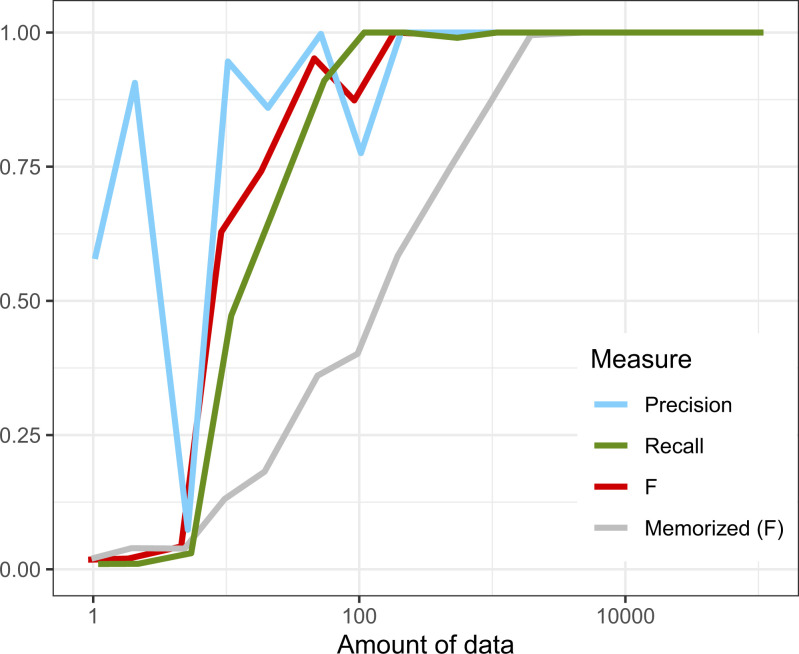
Learning curves for the simplified English grammar (2). Note that here, precision and recall was computed on the top 100 strings. This language is learned to high accuracy considerably faster than memorization.

The model’s learning curve for this grammar is shown in Fig. 4. The model is able to learn a program that closely approximates the target grammar after seeing a few hundred sampled string tokens, showing that the learning model is capable of rapidly learning the required patterns.

**Fig. 5.**
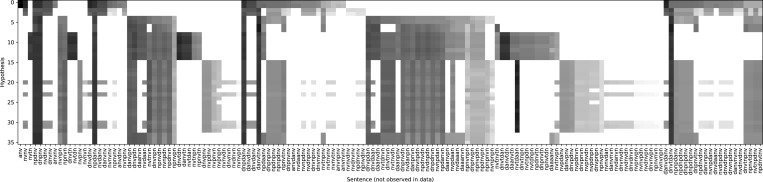
A visualization of the generalizations by the learning model for the simple English grammar, after observing 200 data points. This shows the posterior probability (greyscale) assigned to strings not observed in the data (columns) by each hypothesis (row). The rows are sorted with the highest posterior probability at the top, and duplicate rows with identical generalizations patterns have been removed. The pattern of generalizations is complex, structured (e.g., with correlations apparent between strings), and highly dependent on which hypothesis is selected, even among those with close posterior probabilities.

Fig. 5 visualizes the space of generalizations after observing 200 tokens. Each row shows a different hypothesis, and each column is a string, with cells colored according to the log probability that hypothesis assigns to each string. This figure shows only strings which are not seen in the data and therefore provides a view of how the model generalizes to rare data, a key consideration in many linguistic theories. First, the space of generalizations is complicated: The space of high probability strings does not vary smoothly with hypotheses. Sometimes, hypotheses which are close in posterior probability exhibit very subtly different patterns of generalization; sometimes, hypotheses which agree for many strings differ in other less frequent strings. This shows that the task of selecting exactly a parent’s grammar must be difficult, because top hypotheses on this dataset are only distinguished on rare strings. Additionally, because the space of computations is highly structured, learners will exhibit nontrivial beliefs about constructions they have never seen—even in the absence of language-specific constraints.

## Discussion

Although “poverty of the stimulus” arguments are often considered classic and foundational in linguistic theorizing (3, 155–159), prior reviews (35, 160–162) and theoretical results (28) have been devastating to the hypothesis that learners must be highly constrained in order to ensure that language can be learned. The model developed here provides a concrete alternative to pessimism about the power of learning. It acquires generative systems from relatively small amounts of positive data, working out of a broad class of computations. Ongoing developments in program induction models—such as the ability to reuse components (101), evaluate parital hypotheses (163), work in continuous spaces (164), and apply rich toolkits of programming methods (38)—are likely to help such learning scale.

Learners who operate over relatively unrestricted spaces show many hallmarks which have traditionally been attributed to universal grammar (UG), a domain-specific genetic endowment hypothesized to support language acquisition. Pullum and Scholz (161) identify and reject a number of common claims about language acquisition that have been used to support UG, including the speed with which children learn, the fact that children acquire a system that can deal with essentially unbounded sentences, the fact that their data underdetermine their grammar, and that different individuals in the same community learn similar grammars. The model provides an implementation which also shows such properties—quickly and reliably learning computations from underdetermined evidence—while operating in the most unrestricted space possible. Other authors have emphasized, in support of UG, that children’s knowledge of language goes beyond what they directly observe. The present work highlights one sense in which that fact might be unremarkable: Any learning system that works over a sufficiently rich space of computations will appear to know about strings that have not been observed. This can be seen in even the simplest case of *a^n^*, but the point is most clear for grammars which yield complex patterns of generalizations.

A huge body of literature has been dedicated to articulating and critiquing the subset problem of how it is that learners who use only positive evidence can avoid arriving at an overly general grammar (122, 125, 159, 165–170). If a learner hypothesizes that an ungrammatical string is actually grammatical, they will never receive directly contradictory evidence from positive examples. One solution to this can be found in the present model’s use of distributions, ideas which date back to ref. 22 (see ref. 171) and have a simple basis in probability theory (172): Since probabilities must sum to one, hypotheses that use some probability for unseen strings will necessarily have less probability for the data that are observed, lowering the hypothesis’ likelihood. This fact also interacts with the structural priors, which sometimes favor hypothesizing that unobserved strings are actually grammatical because this leads to a simpler computation.

It is worth emphasizing that the assumptions of no negative evidence (173) have been questioned by many language acquisition researchers (161, 174–178). Our general model could easily be made compatible with positive evidence, or even other forms, including pedagogical evidence, as has been examined in similar statistical setups (179). However, even if children do receive substantially informative negative evidence, they still face a deep inductive problem. To choose a simple example, the formal language *a^n^* is infinite, and yet any data a learner sees—positive or negative—will be finite. Apart from explicit metalinguistic instruction, there does not seem to be any form of negative evidence or rephrasing that could lead learners to deduce this fact directly. Instead, learners must have an inductive mechanism that can consider the infinite language as a possibility, and weigh it against alternatives. We suspect that many structural aspects in language acquisition will have this flavor, where the precise form of evidence is not as crucial as the inductive biases learners possess and the hypotheses they consider.

Our results demonstrate that positive evidence not only can work in theory for discovering the right computation, but it works in practice, using simple, domain-general search techniques that have been applied broadly across cognitive science. At the same time, these methods are still in their infancy. We have only applied them to some of the basic descriptions of linguistic phenomena—ideas like recursion, nesting, simple context-free phrase structure, and context-sensitive dependencies. This work leaves open the question of how more-detailed kinds of phenomena may be acquired—for instance, long-distance dependencies in syntactic islands (180, 181). We note, however, that many such phenomena have been hypothesized to result from UG, but may, in fact, be better explained as properties of constructions and pragmatics (182–184). Either way, the learning of such aspects of grammar have simply not yet been explored using powerful domain-general learning tools.

Our approach was motivated, in part, by Perfors et al. (27), who compared different grammars—for example, finite, finite state, and context-free—and showed that a small amount of child-directed speech from the CHILDES project (185) was sufficient for learners to infer that language had a hierarchical structure. One response to Perfors et al. has been to emphasize linguistic facts that go beyond the mere presence of hierarchical structure, often depending on subtle distinctions and connections between constructions or interpretations (157), although Bayesian models have shown success in learning some such phenomena (186). A more interesting—although we think ultimately misguided—critique of Perfors et al. is that their model “builds in” more than UG because learners consider multiple grammars. Surely it is a more parsimonious theory of human nature that learners come equipped with one single grammar than the seven Perfors et al. used? The argument also applies to our model: How could “building in” an infinite number of possible computations be a more parsimonious solution to the problem of language learning?

It turns out that this thinking is wrong, for a very interesting reason. The issue can best be understood by considering Jorge Luis Borges’ short story, “The Library of Babel” (187). Borges imagines an infinite library full of every possible book—every possible sequence of characters. The curious fact about the library which contains every possible book is that it actually contains essentially no information at all. Its contents can be completely specified with just a few words (“all possible books”), or compressed into an extremely short generating computer program, yielding a negligible minimum description length.^§^ Certainly, it is much easier to describe the entire library than to describe just one of its typical books. The story illustrates that it is often more parsimonious to build in a larger, unconstrained space of hypotheses because a large space can easily have a more concise description. Theories which hypothesize that minimal amounts of structure and content are innate should embrace this perspective.

Our results also address a critique of domain-general approaches to language learning: It is often argued that children must possess constrained sets of hypotheses because there are many constructions that children simply don’t learn. However, our results also showed that there are languages that are difficult to learn, even for a universal model. This fact is not remarkable—it is inevitable. Only a few languages can have short description lengths or equivalently high priors, and so almost all logically possible languages will be difficult to learn. Consequently, there simply must be logically possible generalizations children never make, regardless of whether there are constraints specific to language or not. Learnability analyses have too often ignored these issues and conflated the class which can practically be learned with the total hypothesis space (160).

This model tackles acquisition of language from a different perspective than connectionist models (188, 189), which often are taken as contrary to poverty of the stimulus claims due to their ability to acquire key aspects of language as well as model human performance on learning tasks (152, 190–196). Connectionist approaches have seen remarkable success in recent years in natural language tasks (197–199), including hierarchical languages (200). The datasets studied here may provide a compelling testbed for such neural network models, and are closely related to Turing-complete neural architectures (164).

Finally, our results suggest that the Chomsky–Schützenberger hierarchy—popular for characterizing human and animal communication (201, 202)—may not align with psychological notions of complexity. Because of the chosen form of the prior, the model’s inferences are sensitive to description length rather than hierarchy level: Some languages that have short descriptions are higher on the hierarchy (like $a^{n}b^{n}c^{n}$), and some languages (like Reber) that are lower on the hierarchy are nonetheless difficult because they do not have concise descriptions as programs. Theories of language acquisition would do well to prioritize measures of description length (203, 204) in considering the complexity of hypotheses, bringing such theories in line with experimental work in human learning (59, 88, 131, 205–207).

These results also point to several important directions for future modeling, experimentation, and theoretical work. Many aspects of learning—from words to grammars—may primarily require learners to find concise descriptions of observed data (29, 31, 204, 205). While some languages like the simple English grammar presuppose that categories are known, some of the formal languages—even simple ones like $a^{n}b^{n}$—have the model implicitly discover that different symbols (*a* and *b*) have different distributional properties. The goal of finding concise computational descriptions can likely be applied to other levels of linguistic representation, including part of speech categories or phonemes, where the relevant abstractions are thought to provide simple descriptions of apparent patterns. Generally, however, this work leaves open the question of what domain-specific constraints and knowledge are present in each of these levels of linguistic analysis and how those interface with domain-general capacities. At the very least, claims of domain-specific knowledge will only be tenable when formulated relative to demonstrable shortcomings in implemented domain-general learning theories.

While this model provides a theoretical proposal for learning the patterns that govern sequences of discrete symbols, it is important to remember that real-life language learning is closely tied to language use (208–210), including semantics, pragmatics, and social inference. None of these are captured in the present framework, which was designed to study a simplified setting akin to Gold’s. Although these other processes are central to how children learn language, the present work does suggest that there is unlikely to be an in principle learnability problem even without them. One additional limitation of our work is that it is not clear how to extend such models to a mental lexicon that contains thousands of lexical items—or, more specifically, how induction of structure interfaces with humans’ distinctive memory architecture. Relatedly, this learning model does not learn any meanings associated with strings. However, similar program-learning models have been used to acquire compositional representations of semantics (211–214) while operating over larger vocabularies. These models illustrate one way in which program-like learning models may be extended to richer settings.

## Conclusion

The model described in this paper shows how learners could begin with a simple set of domain-general computational operations and create grammars in order to explain observed data. The model shows that implemented program induction techniques can build representations of provably different computational power, including regular, context-free, and context-sensitive hypotheses. It also shows that such learning requires surprisingly little data, and that positive evidence is sufficient. This model provides an inferential foundation onto which psychological or linguistic constraints—perhaps including pressures in memory or computational limitations (215)—can be added in order to refine debates about what resources learners must necessarily bring to language acquisition. Notably, in this approach, grammars are just one kind of computation that learners might acquire, and the model’s key assumptions and mechanisms have been independently argued to explain nonlinguistic learning in other domains. The model thus points to how language acquisition might be unified with learning more broadly in cognition, including the many domains where children also acquire structures and abstraction from statistical evidence (216).

## Methods

In all formal languages, words and syllables in prior literature have been reduced to single characters so that the primary components that are generated are sequences of characters in a fixed alphabet. Languages are made probabilistic using generative (flip) parameters that prevent data generation from creating strings that are too long or recursions that are too deep. Note that, in evaluating a hypothesis, the initial value for *x* that is passed in is the empty string (*ϵ*); however, when one factor is called by another, it may pass in other arguments. In running, we enumerate execution paths down to a log probability of –15, 64 recursions, 1,024 program steps, or 256 different outputs. For the English example, we increased these bounds to output a larger distribution of strings. All of the code for specifying and sampling the input data and the input data themselves are available at the Fleet GitHub.

Inference was run using a custom implementation of the adaptive parallel tempering scheme from ref. 217 in Fleet, using proposals from ref. 58. This ran five temperatures from 1 to 1.2, spaced exponentially. The inference proposed swaps between chains every 0.25 s and adapting the temperature ladder every 5 s. This inference scheme was run on data consisting of 1, 2, 5, 10, 20, 50, 100, 200, 500, 1,000, 2,000, 5,000, 10,000, 50,000, and 100,000 strings sampled from the target grammar. This inference was run on times varying from 1 min to 7 d total, across all amounts of data (evenly divided). The specific times each language was run for are provided in *SI Appendix*. Note that, in general, it is difficult to search over factors, since changes to one factor may depend on what another factor computes. We therefore run multiple parallel searches while constraining the number of factors n=1,2,3,4 within each search. For instance, on the search with three factors, we reject any proposal that fails to call all three factors, although we note that this still often results in trivial factors. We ran this inference on a collection of Dell servers for varying amounts of time using GNU Parallel (218). We collected the 500 highest posterior hypotheses found in each chain at each amount of data and used this to plot the posterior-weighted F curves shown in figures. We note that such approximation and sampling is not intended as part of the high-level interpretation of our approach. We intend the model to be on Marr’s computational level (133) in that people solve the same problem of inferring the algorithm that generates strings; we do not claim that they necessarily use the same methods as our implementation.

For all languages but the simplified English grammar, we computed precision and recall using the most frequent 25 strings in the data and generated by each hypothesis. Let *S*(*h*) be the set of strings from a hypothesis *h* and let *S*(*D*) be the set of strings for a dataset *D*. Let $S_{25}(h)$ and $S_{25}(D)$ denote the most frequent (or high probability) 25 strings enumerated by the model and occurring in the data, respectively. Let $\hat{P}(h|D)$ denote the probability of *h* given the data, renormalized to sum to one over the top 500 hypotheses found (note that, since the hypothesis space is discrete, the top 500 hypotheses contain virtually all of the posterior probability mass). Then, posterior-weighted precision is defined as

[3] $$\text{precision}=\sum_{h\in H}\hat{P}(h|D)\frac{\left|S_{25}(h)\cap S(D)\right|}{\left|S_{25}(h)\right|},$$

with recall defined analogously. Thus, the precision measures the proportion of the top 25 model strings that occur in the data, and the recall measures the proportion of the top 25 data strings that are output by the model.

Because the simplified English grammar was substantially more complex than other languages, it was run on factor sizes of 1, 2, 3, 4, 5, and 6, with 15 threads each, for 7 d. Precision and recall was computed on the top 100 strings instead of the top 25 in order to better assess learning. In addition, these runs permitted more steps and smaller log probability in order to enumerate more strings.

Several languages (e.g., Braine 66) introduced nonuniform sentence probabilities into the original grammar. This was done so that the strings yielded could be more easily evaluated in our metric of number of top strings. In this way, the tested languages were sometimes more skewed in distribution than the original references. While this makes the string set easier to identify by our metric, it also makes the distribution harder to match, since uniform distributions should be very easy for the model to learn.

## Supplementary Material

Supplementary File

## Data Availability

The C++ library called Fleet, which we created, is distributed under the GNU Public License v3 at GitHub, https://github.com/piantado/Fleet.

## Footnotes

*Note here, and elsewhere, that the connection is analogous rather than a direct formalization of English grammar, since “If if then then” is not a valid English sentence. We focus on examples like $a^{n}b^{n}$ instead of more complex formal languages that directly capture intervening material in order to simplify presentation. Note that closure properties of formal languages under, for example, intersection and homomorphism likely mean that simple formal languages are relevant to richer sets of strings.

^†^The GoldenMean language—strings over Σ={a,b} with no two adjacent *a*s—is a classic example in symbolic dynamics. It contains a Fibonacci number of strings of each length and has a symbolic dynamic entropy equaling the log of the Golden Mean (108).

^‡^We did not require A≠B in this implementation.

^§^For example, F0(x)≔pair(F0(x),sample(Σ)).
