# Supplementary material for: One model for the learning of language
Source: Proc Natl Acad Sci U S A. 2022 Jan 24;119(5):e2021865119. doi: 10.1073/pnas.2021865119 (PMC8812683; doi:10.1073/pnas.2021865119)
Supplement: Supplementary File [file pnas.2021865119.sapp.pdf]

Appendix A: listing of learned representations

January 11, 2022

Language	Alphabet	Time	Factors	Data (Tokens)	Posterior Score	Prec.	Recall	Hypothesis
a^n	abcd	1m	8	1 (1)	-20.7385	1	0.84	$F0(x)$:=λx.pair(if(flip(1/2), Fm0(ϵ), ϵ), a).
a^n	abcd	1m	8	10 (6)	-40.487	1	1	$F0(x)$:=λx.pair(if(flip(1/4), ϵ, F0(ϵ)), a).
a^n	abcd	1m	8	100 (12)	-222.092	1	1	$F0(x)$:=λx.pair(if(flip(7/24), ϵ, F0(ϵ)), a).
a^n	abcd	1m	8	1000 (17)	-1896.89	1	1	$F0(x)$:=λx.pair(if(flip(1/3), ϵ, Fm0(ϵ)), a).
a^n	abcd	1m	8	10000 (22)	-19214.1	1	1	$F0(x)$:=λx.pair(if(flip(1/3), ϵ, Fm0(ϵ)), a).
a^n	abcd	1m	8	100000 (29)	-192125	1	1	$F0(x)$:=λx.pair(if(flip(1/3), ϵ, Fm0(ϵ)), a).
a^n	abcd	1m	8	1 (1)	-27.5165	1	0.04	$F0(x)$:=λx.append(x, append(x, x)).<br>$F1(x)$:=λx.F0(pair(pair(ϵ, a), a)).
a^n	abcd	1m	8	10 (6)	-51.0358	1	0.84	$F0(x)$:=λx.pair(if(flip(1/2), F1(ϵ), ϵ), a).<br>$F1(x)$:=λx.F0(ϵ).
a^n	abcd	1m	8	100 (12)	-229.41	1	1	$F0(x)$:=λx.pair(if(flip(7/24), ϵ, F1(ϵ)), a).<br>$F1(x)$:=λx.Fm0(ϵ).
a^n	abcd	1m	8	1000 (17)	-1904.21	1	1	$F0(x)$:=λx.pair(if(flip(1/3), ϵ, F1(ϵ)), a).<br>$F1(x)$:=λx.Fm0(ϵ).
a^n	abcd	1m	8	10000 (22)	-19221.4	1	1	$F0(x)$:=λx.pair(if(flip(1/3), ϵ, Fm1(ϵ)), a).<br>$F1(x)$:=λx.F0(ϵ).
a^n	abcd	1m	8	100000 (29)	-192133	1	1	$F0(x)$:=λx.pair(if(flip(1/3), x, F1(ϵ)), a).<br>$F1(x)$:=λx.Fm0(ϵ).
a^n	abcd	1m	8	1 (1)	-35.6452	1	0.04	$F0(x)$:=λx.x.<br>$F1(x)$:=λx.F0(append(x, append(x, x))).<br>$F2(x)$:=λx.F1(pair(pair(ϵ, a), a)).
a^n	abcd	1m	8	10 (6)	-58.3538	1	1	$F0(x)$:=λx.pair(if(not(flip(1/4)), Fm0(ϵ), Fm1(ϵ)), a).<br>$F1(x)$:=λx.ϵ.<br>$F2(x)$:=λx.Fm0(ϵ).
a^n	abcd	1m	8	100 (12)	-239.959	1	1	$F0(x)$:=λx.pair(if(not(flip(7/24)), F2(ϵ), Fm1(ϵ)), a).<br>$F1(x)$:=λx.ϵ.<br>$F2(x)$:=λx.Fm0(ϵ).
a^n	abcd	1m	8	1000 (17)	-1912.05	1	1	$F0(x)$:=λx.pair(F1(ϵ), a).<br>$F1(x)$:=λx.if(flip(1/3), ϵ, F0(ϵ)).<br>$F2(x)$:=λx.F0(ϵ).
a^n	abcd	1m	8	10000 (22)	-19229.3	1	1	$F0(x)$:=λx.pair(F1(ϵ), a).<br>$F1(x)$:=λx.if(flip(1/3), ϵ, F0(ϵ)).<br>$F2(x)$:=λx.F0(ϵ).
a^n	abcd	1m	8	100000 (29)	-192140	1	1	$F0(x)$:=λx.pair(F1(ϵ), a).<br>$F1(x)$:=λx.if(flip(1/3), ϵ, F0(ϵ)).<br>$F2(x)$:=λx.F0(ϵ).
a^n	abcd	1m	8	1 (1)	-59.1205	0.94117	0.64	$F0(x)$:=λx.ϵ.<br>$F1(x)$:=λx.F3(x).<br>$F2(x)$:=λx.F1(F0(ϵ)).<br>$F3(x)$:=λx.if(flip(1/24), ϵ, if(flip(1/8), ϵ, append(Fm2(ϵ), head(pair(ϵ, a))))).
a^n	abcd	1m	8	10 (6)	-65.0641	1	1	$F0(x)$:=λx.pair(if(flip(1/3), F2(ϵ), Fm1(ϵ)), a).<br>$F1(x)$:=λx.Fm0(ϵ).<br>$F2(x)$:=λx.x.<br>$F3(x)$:=λx.Fm1(ϵ).

a^n	abcd	1m	8	100 (12)	-246.109	1	0.64	$F0(x)$:=λx.pair(if(flip(1/3), ε, Fm1(ε)), a).<br>$F1(x)$:=λx.Fm3(ε).<br>$F2(x)$:=λx.ε.<br>$F3(x)$:=λx.Fm0(F2(ε)).
a^n	abcd	1m	8	1000 (17)	-1920.23	1	1	$F0(x)$:=λx.pair(if(flip(1/3), ε, Fm1(ε)), a).<br>$F1(x)$:=λx.Fm0(ε).<br>$F2(x)$:=λx.ε.<br>$F3(x)$:=λx.Fm0(F2(ε)).
a^n	abcd	1m	8	10000 (22)	-19237.5	1	1	$F0(x)$:=λx.pair(if(flip(1/3), F2(ε), Fm3(ε)), a).<br>$F1(x)$:=λx.ε.<br>$F2(x)$:=λx.Fm1(ε).<br>$F3(x)$:=λx.F0(ε).
a^n	abcd	1m	8	100000 (29)	-192149	1	1	$F0(x)$:=λx.pair(if(flip(1/3), F2(Fm1(ε)), F3(x)), a).<br>$F1(x)$:=λx.ε.<br>$F2(x)$:=λx.ε.<br>$F3(x)$:=λx.F0(ε).
Σ^+	abcd	10m	8	1 (1)	-6.6447	1	0.04	$F0(x)$:=λx.pair(ε, b).
Σ^+	abcd	10m	8	10 (7)	-72.6706	0.96666	0.6	$F0(x)$:=λx.insert(if(flip(1/2), ε, pair(ε, b)), if(flip(1/2), pair(Fm0(ε), a), ε)).
Σ^+	abcd	10m	8	100 (45)	-805.851	0.96774	1	$F0(x)$:=λx.sample(if(not(flip(7/24)), append(if(flip(1/2), pair(ε, b), pair(ε, a)), F0(ε)), ε)).
Σ^+	abcd	10m	8	1000 (159)	-5259.56	0.96774	1	$F0(x)$:=λx.sample(if(not(flip(7/24)), append(if(flip(1/2), pair(ε, b), pair(ε, a)), F0(ε)), ε)).
Σ^+	abcd	10m	8	10000 (737)	-51630.4	0.96774	1	$F0(x)$:=λx.sample(if(not(flip(7/24)), append(if(flip(1/2), pair(ε, b), pair(ε, a)), F0(ε)), ε)).
Σ^+	abcd	10m	8	100000 (3251)	-523789	0.96774	1	$F0(x)$:=λx.sample(if(not(flip(7/24)), append(if(flip(1/2), pair(ε, b), pair(ε, a)), F0(ε)), ε)).
Σ^+	abcd	10m	8	1 (1)	-13.2693	1	0.04	$F0(x)$:=λx.ε.<br>$F1(x)$:=λx.pair(Fm0(ε), b).
Σ^+	abcd	10m	8	10 (7)	-90.6419	0.375	0.84	$F0(x)$:=λx.append(sample(if(flip(11/24), Σ, pair(ε, a))), if(flip(11/24), ε, F0(ε))).<br>$F1(x)$:=λx.Fm0(x).
Σ^+	abcd	10m	8	100 (45)	-776.833	1	1	$F0(x)$:=λx.append(sample(if(flip(1/2), x, pair(ε, b))), if(flip(7/24), ε, Fm1(ε))).<br>$F1(x)$:=λx.Fm0(pair(ε, a)).
Σ^+	abcd	10m	8	1000 (159)	-4892.42	1	1	$F0(x)$:=λx.append(if(flip(3/8), ε, Fm1(ε)), sample((pair(ε, a) ∪ pair(ε, b)))).<br>$F1(x)$:=λx.F0(ε).
Σ^+	abcd	10m	8	10000 (737)	-47954.7	1	1	$F0(x)$:=λx.insert(pair(ε, a), if(flip(1/3), ε, F1(ε))).<br>$F1(x)$:=λx.if(flip(1/2), pair(if(flip(3/8), ε, Fm1(ε)), b), F0(ε)).
Σ^+	abcd	10m	8	100000 (3251)	-487270	1	1	$F0(x)$:=λx.append(if(flip(1/3), ε, F0(ε)), sample((pair(x, a) ∪ pair(ε, b)))).<br>$F1(x)$:=λx.Fm0(ε).
Σ^+	abcd	10m	8	1 (1)	-20.7049	1	0.04	$F0(x)$:=λx.pair(Fm1(ε), b).<br>$F1(x)$:=λx.ε.<br>$F2(x)$:=λx.F0(ε).
Σ^+	abcd	10m	8	10 (7)	-98.0145	0.16666	1	$F0(x)$:=λx.if(flip(1/2), F2(ε), ε).<br>$F1(x)$:=λx.F0(ε).<br>$F2(x)$:=λx.insert(F1(ε), sample(Σ)).
Σ^+	abcd	10m	8	100 (45)	-787.909	1	1	$F0(x)$:=λx.Fm1(sample((Σ\(pair(ε, d) ∪ pair(ε, c))))).<br>$F1(x)$:=λx.if(flip(7/24), x, append(F2(ε), x)).<br>$F2(x)$:=λx.F0(ε).
Σ^+	abcd	10m	8	1000 (159)	-4905.82	1	1	$F0(x)$:=λx.Fm1(sample((Σ\(pair(ε, d) ∪ pair(ε, c))))).<br>$F1(x)$:=λx.if(flip(3/8), x, append(Fm2(ε), x)).<br>$F2(x)$:=λx.F0(ε).
Σ^+	abcd	10m	8	10000 (737)	-47970	1	1	$F0(x)$:=λx.Fm1(sample((Σ\(pair(ε, d) ∪ pair(ε, c))))).<br>$F1(x)$:=λx.if(flip(3/8), x, append(Fm2(ε), x)).<br>$F2(x)$:=λx.F0(ε).
Σ^+	abcd	10m	8	100000 (3251)	-487283	1	1	$F0(x)$:=λx.Fm1(sample((Σ\(pair(ε, d) ∪ pair(ε, c))))).<br>$F1(x)$:=λx.if(flip(1/3), x, append(F0(ε), x)).<br>$F2(x)$:=λx.F0(ε).
Σ^+	abcd	10m	8	1 (1)	-28.598	1	0.04	$F0(x)$:=λx.Fm1(ε).<br>$F1(x)$:=λx.pair(ε, b).<br>$F2(x)$:=λx.F0(ε).<br>$F3(x)$:=λx.Fm2(ε).
Σ^+	abcd	10m	8	10 (7)	-104.257	0.96428	0.44	$F0(x)$:=λx.Fm1(if(flip(1/2), ε, x)).<br>$F1(x)$:=λx.if(flip(1/2), pair(F1(x), a), x).<br>$F2(x)$:=λx.F0(ε).<br>$F3(x)$:=λx.Fm0(pair(F2(ε), b)).

Σ^+	abcd	10m	8	100 (45)	-825.584	1	1	F0(x):=λx.Fm1(pair(ε, a)).<br>F1(x):=λx.if(flip(1/2), if((ε==ε), append(x, F3(ε)), ε), x).<br>F2(x):=λx.ε.<br>F3(x):=λx.if(flip(1/2), F1(pair(x, b)), append(ε, Fm0(F2(x)))).
Σ^+	abcd	10m	8	1000 (159)	-4916.01	1	1	F0(x):=λx.append(if(not(flip(3/8)), F3(ε), F1(ε)), sample(((Σ\x)\pair(ε, c)))).<br>F1(x):=λx.ε.<br>F2(x):=λx.F0(pair(ε, d)).<br>F3(x):=λx.F2(ε).
Σ^+	abcd	10m	8	10000 (737)	-47980.2	1	1	F0(x):=λx.append(if(not(flip(3/8)), F3(ε), F1(ε)), sample(((Σ\x)\pair(ε, c)))).<br>F1(x):=λx.ε.<br>F2(x):=λx.F0(pair(ε, d)).<br>F3(x):=λx.F2(ε).
Σ^+	abcd	10m	8	100000 (3251)	-487293	1	1	F0(x):=λx.append(if(not(flip(1/3)), F2(ε), F1(ε)), sample(((Σ\x)\pair(ε, c)))).<br>F1(x):=λx.ε.<br>F2(x):=λx.F3(ε).<br>F3(x):=λx.F0(pair(ε, d)).
$(ab)^n$	abcd	1m	8	1 (1)	-24.5189	1	0.84	F0(x):=λx.pair(pair(if(flip(1/2), ε, Fm0(ε)), a), b).
$(ab)^n$	abcd	1m	8	10 (7)	-46.199	1	1	F0(x):=λx.pair(pair(if(flip(1/4), ε, Fm0(ε)), a), b).
$(ab)^n$	abcd	1m	8	100 (12)	-220.463	1	1	F0(x):=λx.pair(pair(if(flip(1/3), ε, F0(ε)), a), b).
$(ab)^n$	abcd	1m	8	1000 (16)	-1969.62	1	1	F0(x):=λx.pair(pair(if(flip(1/3), ε, F0(ε)), a), b).
$(ab)^n$	abcd	1m	8	10000 (22)	-19462.8	1	1	F0(x):=λx.pair(pair(if(flip(1/3), ε, F0(ε)), a), b).
$(ab)^n$	abcd	1m	8	100000 (28)	-192894	1	1	F0(x):=λx.pair(pair(if(flip(1/3), ε, F0(x)), a), b).
$(ab)^n$	abcd	1m	8	1 (1)	-31.8366	1	0.84	F0(x):=λx.if(flip(1/2), ε, Fm1(ε)).<br>F1(x):=λx.pair(pair(F0(ε), a), b).
$(ab)^n$	abcd	1m	8	10 (7)	-53.8615	1	1	F0(x):=λx.if(flip(7/24), ε, Fm1(ε)).<br>F1(x):=λx.pair(pair(Fm0(ε), a), b).
$(ab)^n$	abcd	1m	8	100 (12)	-227.78	1	1	F0(x):=λx.if(flip(1/3), ε, Fm1(ε)).<br>F1(x):=λx.pair(pair(Fm0(ε), a), b).
$(ab)^n$	abcd	1m	8	1000 (16)	-1976.94	1	1	F0(x):=λx.if(flip(1/3), ε, Fm1(ε)).<br>F1(x):=λx.pair(pair(Fm0(ε), a), b).
$(ab)^n$	abcd	1m	8	10000 (22)	-19470.2	1	1	F0(x):=λx.if(flip(1/3), ε, Fm1(ε)).<br>F1(x):=λx.pair(pair(Fm0(ε), a), b).
$(ab)^n$	abcd	1m	8	100000 (28)	-192901	1	1	F0(x):=λx.if(flip(1/3), ε, Fm1(ε)).<br>F1(x):=λx.pair(pair(Fm0(ε), a), b).
$(ab)^n$	abcd	1m	8	1 (1)	-44.8696	1	0.88	F0(x):=λx.append(if(flip(5/24), ε, Fm1(ε)), x).<br>F1(x):=λx.Fm2(ε).<br>F2(x):=λx.Fm0(pair(pair(ε, a), b)).
$(ab)^n$	abcd	1m	8	10 (7)	-61.3578	1	0.88	F0(x):=λx.ε.<br>F1(x):=λx.if(flip(1/4), ε, Fm2(ε)).<br>F2(x):=λx.pair(pair(Fm1(Fm0(ε)), a), b).
$(ab)^n$	abcd	1m	8	100 (12)	-235.621	1	0.88	F0(x):=λx.ε.<br>F1(x):=λx.if(flip(1/3), ε, Fm2(ε)).<br>F2(x):=λx.pair(pair(Fm1(F0(ε)), a), b).
$(ab)^n$	abcd	1m	8	1000 (16)	-1984.78	1	0.88	F0(x):=λx.ε.<br>F1(x):=λx.if(flip(1/3), ε, Fm2(ε)).<br>F2(x):=λx.pair(pair(Fm1(F0(ε)), a), b).
$(ab)^n$	abcd	1m	8	10000 (22)	-19478.7	1	1	F0(x):=λx.ε.<br>F1(x):=λx.pair(x, a).<br>F2(x):=λx.pair(F1(if(flip(1/3), F0(ε), F2(ε))), b).
$(ab)^n$	abcd	1m	8	100000 (28)	-192909	1	1	F0(x):=λx.ε.<br>F1(x):=λx.pair(x, a).<br>F2(x):=λx.pair(F1(if(flip(1/3), F0(ε), F2(ε))), b).
$(ab)^n$	abcd	1m	8	1 (1)	-66.874	1	0.08	F0(x):=λx.append(x, append(x, x)).<br>F1(x):=λx.append(x, Fm2(pair(F0(x), a))).<br>F2(x):=λx.pair(x, b).<br>F3(x):=λx.Fm1(if(flip(1/4), pair(pair(ε, a), b), x)).
$(ab)^n$	abcd	1m	8	10 (7)	-70.9249	1	0.84	F0(x):=λx.pair(Fm2(pair(F1(if(flip(1/4), ε, Fm0(ε))), a)), b).<br>F1(x):=λx.x.<br>F2(x):=λx.x.<br>F3(x):=λx.F0(ε).

$(ab)^n$	abcd	1m	8	100 (12)	-244.495	1	1	$F0(x)$:=λx.pair(Fm1(pair(if(flip(1/3), Fm2(ϵ), Fm0(ϵ)), a)), b).<br>$F1(x)$:=λx.x.<br>$F2(x)$:=λx.ϵ.<br>$F3(x)$:=λx.F0(ϵ).
$(ab)^n$	abcd	1m	8	1000 (16)	-1993.66	1	1	$F0(x)$:=λx.pair(Fm1(pair(if(flip(1/3), Fm2(ϵ), Fm0(ϵ)), a)), b).<br>$F1(x)$:=λx.x.<br>$F2(x)$:=λx.ϵ.<br>$F3(x)$:=λx.F0(ϵ).
$(ab)^n$	abcd	1m	8	10000 (22)	-19486.9	1	1	$F0(x)$:=λx.pair(Fm1(pair(if(flip(1/3), Fm2(ϵ), Fm0(ϵ)), a)), b).<br>$F1(x)$:=λx.x.<br>$F2(x)$:=λx.ϵ.<br>$F3(x)$:=λx.F0(ϵ).
$(ab)^n$	abcd	1m	8	100000 (28)	-192918	1	1	$F0(x)$:=λx.pair(Fm1(pair(if(flip(1/3), Fm2(ϵ), Fm0(x)), a)), b).<br>$F1(x)$:=λx.x.<br>$F2(x)$:=λx.ϵ.<br>$F3(x)$:=λx.Fm0(ϵ).
$a^{1,2,3}$	abcd	10m	8	1 (1)	-6.6447	1	0.33333	$F0(x)$:=λx.pair(ϵ, a).
$a^{1,2,3}$	abcd	10m	8	10 (3)	-24.7998	1	0.33333	$F0(x)$:=λx.pair(ϵ, a).
$a^{1,2,3}$	abcd	10m	8	100 (3)	-118.283	0.75	1	$F0(x)$:=λx.pair(if(flip(3/8), Fm0(Fm0(x)), ϵ), a).
$a^{1,2,3}$	abcd	10m	8	1000 (3)	-1021.02	1	1	$F0(x)$:=λx.pair(if(flip(11/24), pair(if(flip(1/3), pair(ϵ, a), ϵ), a), ϵ), a).
$a^{1,2,3}$	abcd	10m	8	10000 (3)	-9717.24	1	1	$F0(x)$:=λx.pair(if(flip(5/12), pair(if(flip(1/3), pair(ϵ, a), ϵ), a), ϵ), a).
$a^{1,2,3}$	abcd	10m	8	100000 (3)	-97055.8	1	1	$F0(x)$:=λx.pair(if(flip(if(flip(1/2), 3/8, 1/2)), pair(if(flip(1/3), pair(x, a), ϵ), a), x), a).
$a^{1,2,3}$	abcd	10m	8	1 (1)	-13.2693	1	0.33333	$F0(x)$:=λx.ϵ.<br>$F1(x)$:=λx.pair(Fm0(ϵ), a).
$a^{1,2,3}$	abcd	10m	8	10 (3)	-31.4244	1	0.33333	$F0(x)$:=λx.ϵ.<br>$F1(x)$:=λx.pair(Fm0(ϵ), a).
$a^{1,2,3}$	abcd	10m	8	100 (3)	-123.557	1	1	$F0(x)$:=λx.pair(if(flip(3/8), x, ϵ), a).<br>$F1(x)$:=λx.F0(F0(pair(ϵ, a))).
$a^{1,2,3}$	abcd	10m	8	1000 (3)	-1027.23	1	1	$F0(x)$:=λx.pair(if(flip(5/12), x, ϵ), a).<br>$F1(x)$:=λx.F0(F0(pair(ϵ, a))).
$a^{1,2,3}$	abcd	10m	8	10000 (3)	-9724.64	1	1	$F0(x)$:=λx.append(x, if(flip(1/3), x, ϵ)).<br>$F1(x)$:=λx.pair(F0(if(flip(5/12), pair(ϵ, a), ϵ)), a).
$a^{1,2,3}$	abcd	10m	8	100000 (3)	-97057.7	1	1	$F0(x)$:=λx.append(x, if(flip(1/3), x, ϵ)).<br>$F1(x)$:=λx.pair(F0(if(flip(if(flip(1/6), 1/2, 5/12)), pair(ϵ, a), ϵ)), a).
$a^{1,2,3}$	abcd	10m	8	1 (1)	-20.7049	1	0.33333	$F0(x)$:=λx.pair(Fm1(ϵ), a).<br>$F1(x)$:=λx.ϵ.<br>$F2(x)$:=λx.F0(ϵ).
$a^{1,2,3}$	abcd	10m	8	10 (3)	-38.86	1	0.33333	$F0(x)$:=λx.pair(Fm1(ϵ), a).<br>$F1(x)$:=λx.ϵ.<br>$F2(x)$:=λx.F0(ϵ).
$a^{1,2,3}$	abcd	10m	8	100 (3)	-133.508	0.21428	1	$F0(x)$:=λx.pair(F1(ϵ), a).<br>$F1(x)$:=λx.if(flip(1/3), Fm0(ϵ), ϵ).<br>$F2(x)$:=λx.F0(ϵ).
$a^{1,2,3}$	abcd	10m	8	1000 (3)	-1035.77	1	1	$F0(x)$:=λx.if(flip(5/12), pair(x, a), ϵ).<br>$F1(x)$:=λx.pair(x, a).<br>$F2(x)$:=λx.F1(F0(F0(ϵ))).
$a^{1,2,3}$	abcd	10m	8	10000 (3)	-9734.01	1	1	$F0(x)$:=λx.ϵ.<br>$F1(x)$:=λx.if(flip(5/12), pair(if(not(flip(1/3)), ϵ, pair(ϵ, a)), a), F0(ϵ)).<br>$F2(x)$:=λx.pair(F1(ϵ), a).
$a^{1,2,3}$	abcd	10m	8	100000 (3)	-97065.3	1	1	$F0(x)$:=λx.append(if(or(flip(11/24), flip(5/24)), ϵ, pair(if(flip(1/3), x, ϵ), a)), x).<br>$F1(x)$:=λx.pair(ϵ, a).<br>$F2(x)$:=λx.Fm0(F1(ϵ)).
$a^{1,2,3}$	abcd	10m	8	1 (1)	-28.598	1	0.33333	$F0(x)$:=λx.pair(ϵ, a).<br>$F1(x)$:=λx.ϵ.<br>$F2(x)$:=λx.Fm0(Fm1(ϵ)).<br>$F3(x)$:=λx.F2(ϵ).
$a^{1,2,3}$	abcd	10m	8	10 (3)	-46.7531	1	0.33333	$F0(x)$:=λx.Fm2(ϵ).<br>$F1(x)$:=λx.Fm0(ϵ).<br>$F2(x)$:=λx.pair(ϵ, a).<br>$F3(x)$:=λx.F1(ϵ).

$a^{1,2,3}$	abcd	10m	8	100 (3)	-141.689	0.21428	1	$F0(x)$:=λx.F1(F2(ϵ)).<br>$F1(x)$:=λx.pair(if(flip(1/3), F3(ϵ), ϵ), a).<br>$F2(x)$:=λx.ϵ.<br>$F3(x)$:=λx.F0(ϵ).
$a^{1,2,3}$	abcd	10m	8	1000 (3)	-1045.96	1	1	$F0(x)$:=λx.F1(Fm1(pair(Fm2(ϵ), a))).<br>$F1(x)$:=λx.pair(if(not(flip(5/12)), ϵ, x), a).<br>$F2(x)$:=λx.ϵ.<br>$F3(x)$:=λx.F0(ϵ).
$a^{1,2,3}$	abcd	10m	8	10000 (3)	-9746.37	1	1	$F0(x)$:=λx.pair(if(not(flip(5/12)), ϵ, x), a).<br>$F1(x)$:=λx.pair(if(flip(1/3), pair(x, a), tail(F2(ϵ))), a).<br>$F2(x)$:=λx.ϵ.<br>$F3(x)$:=λx.F0(Fm1(ϵ)).
$a^{1,2,3}$	abcd	10m	8	100000 (3)	-97079	1	1	$F0(x)$:=λx.ϵ.<br>$F1(x)$:=λx.append(if(flip(1/3), x, Fm0(ϵ)), x).<br>$F2(x)$:=λx.pair(x, a).<br>$F3(x)$:=λx.Fm2(if(flip(1/2), ϵ, if(flip(1/8), ϵ, F1(Fm2(ϵ))))).
$a^{1,2,3,4}$	abcd	10m	8	1 (1)	-15.5719	1	0.25	$F0(x)$:=λx.pair(pair(pair(ϵ, a), a), a).
$a^{1,2,3,4}$	abcd	10m	8	10 (3)	-26.4093	0.19047	1	$F0(x)$:=λx.pair(if(flip(1/2), Fm0(ϵ), ϵ), a).
$a^{1,2,3,4}$	abcd	10m	8	100 (4)	-135.546	0.23529	1	$F0(x)$:=λx.pair(if(flip(5/12), F0(ϵ), ϵ), a).
$a^{1,2,3,4}$	abcd	10m	8	1000 (4)	-1191.67	0.57142	1	$F0(x)$:=λx.pair(if(flip(11/24), Fm0(if(flip(1/2), head(Fm0(x)), ϵ)), ϵ), a).
$a^{1,2,3,4}$	abcd	10m	8	10000 (4)	-11518.8	1	1	$F0(x)$:=λx.pair(if(flip(11/24), if(flip(7/24), pair(pair(ϵ, a), a), Fm0(tail(Fm0(ϵ)))), ϵ), a).
$a^{1,2,3,4}$	abcd	10m	8	100000 (4)	-115630	1	1	$F0(x)$:=λx.pair(if(and(flip(5/24), (x==ϵ)), Fm0(pair(ϵ, a)), if(flip(1/3), pair(x, a), x)), a).
$a^{1,2,3,4}$	abcd	10m	8	1 (1)	-22.1965	1	0.25	$F0(x)$:=λx.ϵ.<br>$F1(x)$:=λx.pair(pair(pair(F0(ϵ), a), a), a).
$a^{1,2,3,4}$	abcd	10m	8	10 (3)	-33.7271	0.19047	1	$F0(x)$:=λx.ϵ.<br>$F1(x)$:=λx.pair(if(flip(1/2), Fm1(ϵ), Fm0(ϵ)), a).
$a^{1,2,3,4}$	abcd	10m	8	100 (4)	-142.864	0.23529	1	$F0(x)$:=λx.ϵ.<br>$F1(x)$:=λx.pair(if(flip(5/12), F1(F0(ϵ)), ϵ), a).
$a^{1,2,3,4}$	abcd	10m	8	1000 (4)	-1201	0.23529	1	$F0(x)$:=λx.ϵ.<br>$F1(x)$:=λx.pair(if(flip(5/12), Fm1(F0(ϵ)), ϵ), a).
$a^{1,2,3,4}$	abcd	10m	8	10000 (4)	-11523.7	1	1	$F0(x)$:=λx.ϵ.<br>$F1(x)$:=λx.pair(if(flip(11/24), if(flip(7/24), pair(pair(ϵ, a), a), Fm1(F0(Fm1(ϵ)))), ϵ), a).
$a^{1,2,3,4}$	abcd	10m	8	100000 (4)	-115642	1	1	$F0(x)$:=λx.ϵ.<br>$F1(x)$:=λx.pair(if(flip(if(flip(1/12), 1/8, 1/2)), if(flip(7/24), pair(pair(ϵ, a), a), Fm1(F0(Fm1(ϵ)))), ϵ), a).
$a^{1,2,3,4}$	abcd	10m	8	1 (1)	-29.6321	1	0.25	$F0(x)$:=λx.ϵ.<br>$F1(x)$:=λx.pair(pair(pair(F0(ϵ), a), a), a).<br>$F2(x)$:=λx.Fm1(ϵ).
$a^{1,2,3,4}$	abcd	10m	8	10 (3)	-41.5681	0.19047	1	$F0(x)$:=λx.if(flip(1/2), ϵ, F1(ϵ)).<br>$F1(x)$:=λx.pair(Fm0(ϵ), a).<br>$F2(x)$:=λx.Fm1(ϵ).
$a^{1,2,3,4}$	abcd	10m	8	100 (4)	-150.705	0.23529	1	$F0(x)$:=λx.pair(Fm1(ϵ), a).<br>$F1(x)$:=λx.if(flip(5/12), Fm0(ϵ), ϵ).<br>$F2(x)$:=λx.F0(ϵ).
$a^{1,2,3,4}$	abcd	10m	8	1000 (4)	-1205.45	1	1	$F0(x)$:=λx.append(if(flip(7/24), x, if(flip(1/2), if(flip(3/8), ϵ, if(flip(1/3), ϵ, append(x, x))), ϵ)), x).<br>$F1(x)$:=λx.F0(if(flip(11/24), pair(x, a), ϵ)).<br>$F2(x)$:=λx.pair(F1(ϵ), a).
$a^{1,2,3,4}$	abcd	10m	8	10000 (4)	-11547.9	1	1	$F0(x)$:=λx.append(if(flip(7/24), x, if(flip(1/2), if(flip(1/2), ϵ, if(flip(5/24), ϵ, append(x, x))), tail(ϵ))), x).<br>$F1(x)$:=λx.F0(if(flip(11/24), pair(ϵ, a), ϵ)).<br>$F2(x)$:=λx.pair(F1(ϵ), a).
$a^{1,2,3,4}$	abcd	10m	8	100000 (4)	-115664	1	1	$F0(x)$:=λx.append(if(flip(7/24), x, if(flip(1/3), if(flip(1/8), ϵ, if(flip(7/24), ϵ, append(x, x))), ϵ)), x).<br>$F1(x)$:=λx.F0(if(flip(if(flip(1/4), 1/2, 11/24)), x, ϵ)).<br>$F2(x)$:=λx.pair(Fm1(pair(ϵ, a)), a).
$a^{1,2,3,4}$	abcd	10m	8	1 (1)	-38.2184	1	0.25	$F0(x)$:=λx.pair(x, a).<br>$F1(x)$:=λx.pair(pair(ϵ, a), a).<br>$F2(x)$:=λx.F0(F1(ϵ)).<br>$F3(x)$:=λx.F2(ϵ).
$a^{1,2,3,4}$	abcd	10m	8	10 (3)	-49.7489	0.19047	1	$F0(x)$:=λx.pair(if(flip(1/2), ϵ, Fm2(ϵ)), a).<br>$F1(x)$:=λx.Fm2(ϵ).<br>$F2(x)$:=λx.F0(ϵ).<br>$F3(x)$:=λx.F1(ϵ).

$a^{1,2,3,4}$	abcd	10m	8	100 (4)	-158.886	0.23529	1	F0(x):=λx.Fm2(ε).<br>F1(x):=λx.F0(ε).<br>F2(x):=λx.pair(if(flip(5/12), F1(ε), ε), a).<br>F3(x):=λx.F1(ε).
$a^{1,2,3,4}$	abcd	10m	8	1000 (4)	-1217.03	0.23529	1	F0(x):=λx.ε.<br>F1(x):=λx.pair(if(flip(5/12), Fm2(F0(ε)), ε), a).<br>F2(x):=λx.Fm1(ε).<br>F3(x):=λx.F2(ε).
$a^{1,2,3,4}$	abcd	10m	8	10000 (4)	-11765.5	0.36363	1	F0(x):=λx.pair(x, a).<br>F1(x):=λx.if(flip(1/3), pair(x, a), ε).<br>F2(x):=λx.if(or(flip(11/24), not(flip(5/12))), Fm1(F1(x)), Fm3(ε)).<br>F3(x):=λx.Fm0(Fm2(ε)).
$a^{1,2,3,4}$	abcd	10m	8	100000 (4)	-116352	1	1	F0(x):=λx.pair(x, a).<br>F1(x):=λx.if(flip(11/24), pair(x, a), ε).<br>F2(x):=λx.if((ε==x), Fm1(F1(Fm1(ε))), ε).<br>F3(x):=λx.Fm0(Fm2(ε)).
$a^n b^m$	abcd	1h	8	1 (1)	-24.5089	1	0.28	F0(x):=λx.pair(if(flip(1/2), pair(ε, a), F0(ε)), b).
$a^n b^m$	abcd	1h	8	10 (8)	-85.8615	1	1	F0(x):=λx.append(if(flip(5/24), pair(ε, a), F0(if(flip(1/6), pair(ε, a), x))), head(pair(x, b))).
$a^n b^m$	abcd	1h	8	100 (43)	-458.127	1	1	F0(x):=λx.append(if(flip(1/6), pair(ε, a), F0(if(and((x==ε), flip(1/3)), pair(ε, a), x))), head(pair(x, b))).
$a^n b^m$	abcd	1h	8	1000 (103)	-4135.61	1	1	F0(x):=λx.append(if(not(flip(if((x==head(x)), 1/4, 1/24))), ε, pair(pair(append(x, x), a), a)), if(or(empty(x), not(flip(1/3))), pair(Fm0(append(x, pair(head(x), a))), b), pair(if(not(flip(5/12)), ε, head(x)), a))).
$a^n b^m$	abcd	1h	8	10000 (189)	-40098	1	1	F0(x):=λx.append(if(not(flip(if((x==head(sample(x))), 1/4, 1/24))), ε, pair(pair(append(x, x), a), a)), if(or(empty(x), not(flip(1/3))), pair(Fm0(append(x, pair(head(x), a))), b), pair(if(not(flip(5/12)), ε, if(or(empty(ε), flip(1/4)), pair(ε, a), if(flip(11/24), ε, ε))), a))).
$a^n b^m$	abcd	1h	8	100000 (324)	-404010	1	1	F0(x):=λx.append(if(not(flip(if((x==head(sample(x))), 1/4, 1/24))), ε, pair(pair(append(x, x), a), a)), if(or(empty(x), not(flip(1/3))), pair(Fm0(append(x, pair(head(x), a))), b), pair(if(not(flip(5/12)), ε, if(or(empty(ε), flip(1/4)), pair(ε, a), if(flip(11/24), ε, x))), a))).
$a^n b^m$	abcd	1h	8	1 (1)	-31.8267	1	0.28	F0(x):=λx.pair(if(flip(1/2), pair(ε, a), F0(ε)), b).<br>F1(x):=λx.Fm0(ε).
$a^n b^m$	abcd	1h	8	10 (8)	-81.785	1	1	F0(x):=λx.pair(if(flip(7/24), ε, F0(ε)), a).<br>F1(x):=λx.pair(if(flip(1/3), Fm0(ε), F1(ε)), b).
$a^n b^m$	abcd	1h	8	100 (43)	-426.336	1	1	F0(x):=λx.pair(if(flip(7/24), ε, Fm0(ε)), a).<br>F1(x):=λx.pair(if(flip(3/8), F0(ε), F1(ε)), b).
$a^n b^m$	abcd	1h	8	1000 (103)	-3876.21	1	1	F0(x):=λx.pair(if(flip(1/3), ε, Fm0(ε)), a).<br>F1(x):=λx.pair(if(flip(1/3), Fm0(ε), Fm1(ε)), b).
$a^n b^m$	abcd	1h	8	10000 (189)	-38426.9	1	1	F0(x):=λx.pair(if(flip(1/3), ε, Fm0(ε)), a).<br>F1(x):=λx.pair(if(flip(1/3), Fm0(ε), Fm1(ε)), b).
$a^n b^m$	abcd	1h	8	100000 (324)	-384892	1	1	F0(x):=λx.pair(if(flip(1/3), ε, F0(ε)), a).<br>F1(x):=λx.pair(if(flip(1/3), F0(x), Fm1(ε)), b).
$a^n b^m$	abcd	1h	8	1 (1)	-39.6677	1	0.28	F0(x):=λx.ε.<br>F1(x):=λx.if(flip(1/2), pair(Fm0(ε), a), F2(ε)).<br>F2(x):=λx.pair(Fm1(ε), b).
$a^n b^m$	abcd	1h	8	10 (8)	-91.7126	1	1	F0(x):=λx.pair(if(flip(1/2), ε, F0(ε)), a).<br>F1(x):=λx.ε.<br>F2(x):=λx.pair(if(flip(1/2), Fm2(x), F0(F1(ε))), b).
$a^n b^m$	abcd	1h	8	100 (43)	-439.999	1	1	F0(x):=λx.pair(if(not(flip(7/24)), F0(ε), ε), a).<br>F1(x):=λx.if(not(flip(3/8)), Fm2(ε), F0(ε)).<br>F2(x):=λx.pair(F1(ε), b).
$a^n b^m$	abcd	1h	8	1000 (103)	-3889.87	1	1	F0(x):=λx.pair(if(not(flip(1/3)), F0(ε), ε), a).<br>F1(x):=λx.if(not(flip(1/3)), Fm2(ε), Fm0(ε)).<br>F2(x):=λx.pair(F1(ε), b).
$a^n b^m$	abcd	1h	8	10000 (189)	-38440.6	1	1	F0(x):=λx.pair(if(not(flip(1/3)), F0(ε), ε), a).<br>F1(x):=λx.if(not(flip(1/3)), Fm2(ε), Fm0(ε)).<br>F2(x):=λx.pair(F1(ε), b).
$a^n b^m$	abcd	1h	8	100000 (324)	-384905	1	1	F0(x):=λx.pair(if(not(flip(1/3)), F0(ε), ε), a).<br>F1(x):=λx.if(not(flip(1/3)), Fm2(ε), Fm0(ε)).<br>F2(x):=λx.pair(F1(ε), b).
$a^n b^m$	abcd	1h	8	1 (1)	-47.8486	1	0.28	F0(x):=λx.pair(if(flip(1/2), F3(ε), pair(F2(ε), a)), b).<br>F1(x):=λx.Fm0(ε).<br>F2(x):=λx.ε.<br>F3(x):=λx.Fm1(ε).
$a^n b^m$	abcd	1h	8	10 (8)	-99.1107	1	1	F0(x):=λx.Fm1(ε).<br>F1(x):=λx.pair(if(flip(1/3), ε, Fm1(ε)), a).<br>F2(x):=λx.pair(if(flip(1/2), F0(ε), F2(ε)), b).<br>F3(x):=λx.F2(ε).

$a^n b^m$	abcd	1h	8	100 (43)	-445.376	1	1	$F0(x)$:=λx.Fm1(ϵ).<br>$F1(x)$:=λx.pair(if(flip(7/24), ϵ, Fm1(ϵ)), a).<br>$F2(x)$:=λx.pair(if(flip(5/12), F0(ϵ), F2(x)), b).<br>$F3(x)$:=λx.F2(ϵ).
$a^n b^m$	abcd	1h	8	1000 (103)	-3908.01	1	1	$F0(x)$:=λx.ϵ.<br>$F1(x)$:=λx.if(not(flip(1/3)), F3(ϵ), F2(ϵ)).<br>$F2(x)$:=λx.sample(if(flip(1/3), pair(F0(ϵ), a), append(F2(ϵ), pair(ϵ, a)))).<br>$F3(x)$:=λx.pair(Fm1(ϵ), b).
$a^n b^m$	abcd	1h	8	10000 (189)	-38439.6	1	1	$F0(x)$:=λx.pair(Fm2(ϵ), a).<br>$F1(x)$:=λx.if(flip(1/3), F0(if(flip(1/8), ϵ, ϵ)), F3(ϵ)).<br>$F2(x)$:=λx.if(flip(1/3), ϵ, F0(ϵ)).<br>$F3(x)$:=λx.pair(Fm1(ϵ), b).
$a^n b^m$	abcd	1h	8	100000 (324)	-384908	1	1	$F0(x)$:=λx.pair(Fm2(ϵ), a).<br>$F1(x)$:=λx.if(flip(1/3), F0(ϵ), Fm3(ϵ)).<br>$F2(x)$:=λx.if(flip(1/3), ϵ, Fm0(ϵ)).<br>$F3(x)$:=λx.pair(Fm1(ϵ), b).
GoldenMean	abcd	1h	8	1 (1)	-11.1083	1	0.04	$F0(x)$:=λx.pair(pair(ϵ, b), b).
GoldenMean	abcd	1h	8	10 (8)	-80.2682	0.96774	0.6	$F0(x)$:=λx.append(x, if(flip(7/24), ϵ, Fm0(pair(if(flip(1/2), ϵ, pair(ϵ, a)), b)))).
GoldenMean	abcd	1h	8	100 (33)	-422.819	1	1	$F0(x)$:=λx.append(x, if(flip(if(empty(x), 1/6, 11/24)), sample(if(not(or(empty(x), flip(1/6))), ϵ, pair(ϵ, a))), Fm0(pair(if(not(flip(1/3)), ϵ, pair(ϵ, a)), b)))).
GoldenMean	abcd	1h	8	1000 (110)	-3901.99	1	1	$F0(x)$:=λx.append(x, if(flip(if(empty(x), 1/4, 1/2)), sample(if(not(or(empty(x), flip(1/4))), ϵ, pair(ϵ, a))), Fm0(pair(if(not(flip(1/3)), ϵ, pair(ϵ, a)), b)))).
GoldenMean	abcd	1h	8	10000 (408)	-38712.9	1	1	$F0(x)$:=λx.append(x, if(flip(if(empty(x), 1/6, 11/24)), sample(if(not(or(empty(x), flip(1/6))), ϵ, pair(ϵ, a))), Fm0(pair(if(not(flip(1/3)), ϵ, pair(ϵ, a)), b)))).
GoldenMean	abcd	1h	8	100000 (1434)	-384902	1	1	$F0(x)$:=λx.append(x, if(flip(if(empty(x), 1/6, 11/24)), sample(if(not(or(empty(x), flip(1/6))), ϵ, pair(ϵ, a))), Fm0(pair(if(not(flip(1/3)), ϵ, pair(ϵ, a)), b)))).
GoldenMean	abcd	1h	8	1 (1)	-17.7329	1	0.04	$F0(x)$:=λx.pair(ϵ, b).<br>$F1(x)$:=λx.pair(F0(ϵ), b).
GoldenMean	abcd	1h	8	10 (8)	-79.2416	1	0.6	$F0(x)$:=λx.if(flip(1/2), x, pair(x, a)).<br>$F1(x)$:=λx.pair(F0(if(flip(1/2), F1(ϵ), ϵ)), b).
GoldenMean	abcd	1h	8	100 (33)	-403.714	1	1	$F0(x)$:=λx.pair(if(flip(7/24), ϵ, Fm1(ϵ)), b).<br>$F1(x)$:=λx.if(flip(1/2), Fm0(ϵ), pair(if(flip(1/2), ϵ, F0(ϵ)), a)).
GoldenMean	abcd	1h	8	1000 (110)	-3867.62	1	1	$F0(x)$:=λx.pair(if(flip(1/4), ϵ, Fm1(ϵ)), b).<br>$F1(x)$:=λx.if(flip(1/2), F0(ϵ), pair(if(flip(1/2), ϵ, Fm0(ϵ)), a)).
GoldenMean	abcd	1h	8	10000 (408)	-38305.3	1	1	$F0(x)$:=λx.pair(if(flip(11/24), insert(ϵ, x), F1(pair(ϵ, a))), a).<br>$F1(x)$:=λx.if(and((x==ϵ), flip(1/3)), F0(ϵ), pair(if(flip(3/8), F0(ϵ), if(not(flip(11/24)), Fm1(pair(ϵ, b)), ϵ)), b)).
GoldenMean	abcd	1h	8	100000 (1434)	-380664	1	1	$F0(x)$:=λx.pair(if(flip(11/24), ϵ, F1(pair(x, b))), a).<br>$F1(x)$:=λx.if(and(empty(x), flip(3/8)), F0(ϵ), pair(if(flip(5/12), F0(x), if(not(flip(11/24)), Fm1(pair(ϵ, c)), ϵ)), b)).
GoldenMean	abcd	1h	8	1 (1)	-25.1685	1	0.04	$F0(x)$:=λx.ϵ.<br>$F1(x)$:=λx.ϵ.<br>$F2(x)$:=λx.pair(pair(F0(F1(ϵ)), b), b).
GoldenMean	abcd	1h	8	10 (8)	-88.1932	1	0.6	$F0(x)$:=λx.if(flip(11/24), x, pair(x, a)).<br>$F1(x)$:=λx.pair(F0(if(flip(1/2), F2(ϵ), ϵ)), b).<br>$F2(x)$:=λx.Fm1(ϵ).
GoldenMean	abcd	1h	8	100 (33)	-429.63	0.96	1	$F0(x)$:=λx.if(flip(1/2), x, pair(x, a)).<br>$F1(x)$:=λx.if(flip(1/8), pair(ϵ, b), F0(if(flip(3/8), ϵ, pair(F1(ϵ), b)))).<br>$F2(x)$:=λx.append(Fm1(ϵ), if(flip(1/2), pair(ϵ, b), ϵ)).
GoldenMean	abcd	1h	8	1000 (110)	-4116.25	0.96	1	$F0(x)$:=λx.if(flip(1/2), x, pair(x, a)).<br>$F1(x)$:=λx.if(flip(1/24), pair(append(ϵ, x), b), F0(if(flip(5/12), ϵ, pair(F1(pair(ϵ, b)), b)))).<br>$F2(x)$:=λx.append(F1(ϵ), if(flip(11/24), pair(x, b), ϵ)).
GoldenMean	abcd	1h	8	10000 (408)	-40724.3	0.96	1	$F0(x)$:=λx.if(flip(1/2), x, pair(x, a)).<br>$F1(x)$:=λx.if(flip(1/24), pair(append(ϵ, x), b), F0(if(flip(5/12), ϵ, pair(F1(pair(ϵ, b)), b)))).<br>$F2(x)$:=λx.append(F1(ϵ), if(flip(11/24), pair(x, b), ϵ)).
GoldenMean	abcd	1h	8	100000 (1434)	-404179	0.96	1	$F0(x)$:=λx.if(flip(1/2), x, pair(x, a)).<br>$F1(x)$:=λx.if(flip(1/24), pair(append(ϵ, x), b), F0(if(flip(5/12), ϵ, pair(F1(pair(ϵ, b)), b)))).<br>$F2(x)$:=λx.append(F1(ϵ), if(flip(11/24), pair(x, b), ϵ)).
GoldenMean	abcd	1h	8	1 (1)	-33.0616	1	0.04	$F0(x)$:=λx.ϵ.<br>$F1(x)$:=λx.pair(ϵ, b).<br>$F2(x)$:=λx.pair(Fm1(ϵ), b).<br>$F3(x)$:=λx.F2(Fm0(ϵ)).

GoldenMean	abcd	1h	8	10 (8)	-95.2635	1	0.6	$F0(x)$:=λx.if(flip(1/2), x, pair(x, a)).<br>$F1(x)$:=λx.if(flip(1/2), F2(ϵ), ϵ).<br>$F2(x)$:=λx.pair(F0(F1(ϵ)), b).<br>$F3(x)$:=λx.F2(ϵ).
GoldenMean	abcd	1h	8	100 (33)	-428.33	1	1	$F0(x)$:=λx.F1(F3(ϵ)).<br>$F1(x)$:=λx.append(pair(if(flip(1/2), ϵ, pair(ϵ, a)), b), x).<br>$F2(x)$:=λx.if(not(flip(3/8)), Fm1(ϵ), pair(ϵ, a)).<br>$F3(x)$:=λx.if(flip(1/2), Fm0(ϵ), Fm2(ϵ)).
GoldenMean	abcd	1h	8	1000 (110)	-3877.1	1	1	$F0(x)$:=λx.ϵ.<br>$F1(x)$:=λx.pair(if(not(flip(5/12)), Fm2(ϵ), Fm0(ϵ)), a).<br>$F2(x)$:=λx.pair(if(flip(7/24), ϵ, F3(ϵ)), b).<br>$F3(x)$:=λx.if(flip(1/2), F1(ϵ), F2(ϵ)).
GoldenMean	abcd	1h	8	10000 (408)	-38793.5	1	1	$F0(x)$:=λx.ϵ.<br>$F1(x)$:=λx.pair(if(not(flip(5/12)), Fm2(ϵ), ϵ), a).<br>$F2(x)$:=λx.pair(if(flip(1/4), ϵ, F3(ϵ)), b).<br>$F3(x)$:=λx.if(not(flip(1/2)), Fm1(Fm0(ϵ)), F2(ϵ)).
GoldenMean	abcd	1h	8	100000 (1434)	-385444	1	1	$F0(x)$:=λx.x.<br>$F1(x)$:=λx.pair(if(not(flip(5/12)), Fm2(ϵ), F0(ϵ)), a).<br>$F2(x)$:=λx.pair(if(flip(1/4), ϵ, F3(ϵ)), b).<br>$F3(x)$:=λx.if(flip(1/2), Fm1(ϵ), F2(ϵ)).
Even	abcd	1h	8	1 (1)	-33.4263	1	0	$F0(x)$:=λx.pair(pair(pair(pair(pair(pair(pair(ϵ, b), b), a), a), a), a), b).
Even	abcd	1h	8	10 (7)	-69.2871	1	1	$F0(x)$:=λx.append(sample((pair(pair(ϵ, a), a) $\cup$ pair(ϵ, b))), if(flip(1/2), F0(ϵ), ϵ)).
Even	abcd	1h	8	100 (38)	-468.823	1	1	$F0(x)$:=λx.append(if(not(flip(3/8)), Fm0(ϵ), ϵ), if(not(flip(5/12)), pair(ϵ, b), pair(pair(ϵ, a), a))).
Even	abcd	1h	8	1000 (171)	-5524.48	1	1	$F0(x)$:=λx.sample(if(flip(3/8), (pair(ϵ, b) $\cup$ pair(pair(ϵ, a), a)), append(sample(if(flip(5/12), pair(pair(ϵ, a), a), pair(ϵ, b))), Fm0(ϵ)))).
Even	abcd	1h	8	10000 (729)	-51151.2	1	1	$F0(x)$:=λx.append(if(not(flip(1/3)), F0(append(pair(x, d), x)), ϵ), if(not(flip(5/12)), pair(ϵ, b), pair(pair(ϵ, a), a))).
Even	abcd	1h	8	100000 (2978)	-515754	1	1	$F0(x)$:=λx.sample(if(flip(3/8), (pair(ϵ, b) $\cup$ pair(pair(ϵ, a), a)), append(sample(if(flip(5/12), pair(pair(ϵ, a), a), pair(ϵ, b))), Fm0(ϵ)))).
Even	abcd	1h	8	1 (1)	-37.8662	0.5	0	$F0(x)$:=λx.insert(x, x).<br>$F1(x)$:=λx.pair(F0(pair(append(pair(sample(Σ), a), ϵ), a)), b).
Even	abcd	1h	8	10 (7)	-80.9977	0.96969	1	$F0(x)$:=λx.if(flip(1/2), pair(pair(F0(ϵ), a), a), if(flip(1/2), ϵ, pair(Fm0(ϵ), b))).<br>$F1(x)$:=λx.F0(ϵ).
Even	abcd	1h	8	100 (38)	-479.578	1	1	$F0(x)$:=λx.if(flip(1/3), pair(pair(Fm0(ϵ), a), a), if(flip(1/2), x, pair(Fm0(x), b))).<br>$F1(x)$:=λx.F0(pair(ϵ, b)).
Even	abcd	1h	8	1000 (171)	-5526.91	1	1	$F0(x)$:=λx.pair(if(flip(3/8), ϵ, Fm1(pair(x, a))), b).<br>$F1(x)$:=λx.if(flip(5/12), pair(pair(if(not(flip(3/8)), F1(append(x, insert(x, x))), ϵ), a), a), F0(insert(head(x), pair(x, b)))).
Even	abcd	1h	8	10000 (729)	-51135	1	1	$F0(x)$:=λx.pair(if(flip(3/8), ϵ, F1(pair(x, b))), b).<br>$F1(x)$:=λx.if(flip(5/12), pair(pair(if(not(flip(3/8)), F1(append(x, append(x, x))), ϵ), a), a), F0(x)).
Even	abcd	1h	8	100000 (2978)	-514366	1	1	$F0(x)$:=λx.pair(if(flip(3/8), ϵ, F1(pair(append(x, x), c))), b).<br>$F1(x)$:=λx.if(flip(5/12), pair(pair(if(not(flip(1/3)), F1(append(x, append(x, x))), ϵ), a), a), Fm0(x)).
Even	abcd	1h	8	1 (1)	-42.4713	1	0.04	$F0(x)$:=λx.pair(pair(ϵ, b), a).<br>$F1(x)$:=λx.insert(x, x).<br>$F2(x)$:=λx.F1(pair(F0(ϵ), a)).
Even	abcd	1h	8	10 (7)	-82.0092	1	1	$F0(x)$:=λx.pair(pair(ϵ, a), a).<br>$F1(x)$:=λx.Fm0(ϵ).<br>$F2(x)$:=λx.append(if(flip(1/2), ϵ, F2(ϵ)), if(flip(1/2), Fm1(ϵ), pair(ϵ, b))).
Even	abcd	1h	8	100 (38)	-479.005	1	1	$F0(x)$:=λx.pair(pair(ϵ, a), a).<br>$F1(x)$:=λx.F0(ϵ).<br>$F2(x)$:=λx.append(if(flip(5/12), ϵ, F2(ϵ)), if(flip(5/12), Fm1(ϵ), pair(ϵ, b))).
Even	abcd	1h	8	1000 (171)	-5555.53	1	1	$F0(x)$:=λx.if(not(flip(5/12)), Fm2(ϵ), ϵ).<br>$F1(x)$:=λx.if(flip(3/8), pair(pair(ϵ, a), a), pair(ϵ, b)).<br>$F2(x)$:=λx.append(F0(ϵ), F1(ϵ)).
Even	abcd	1h	8	10000 (729)	-51325.2	1	1	$F0(x)$:=λx.if(not(flip(5/12)), Fm2(ϵ), ϵ).<br>$F1(x)$:=λx.if(flip(3/8), pair(pair(ϵ, a), a), pair(ϵ, b)).<br>$F2(x)$:=λx.append(F0(ϵ), F1(ϵ)).
Even	abcd	1h	8	100000 (2978)	-517974	1	1	$F0(x)$:=λx.if(not(flip(5/12)), F2(ϵ), x).<br>$F1(x)$:=λx.if(flip(5/12), pair(pair(ϵ, a), a), x).<br>$F2(x)$:=λx.append(F0(ϵ), F1(pair(ϵ, b))).
Even	abcd	1h	8	1 (1)	-54.6049	1	0.04	$F0(x)$:=λx.pair(x, a).<br>$F1(x)$:=λx.pair(ϵ, b).<br>$F2(x)$:=λx.append(F1(ϵ), pair(F0(x), a)).<br>$F3(x)$:=λx.F2(F2(ϵ)).

Even	abcd	1h	8	10 (7)	-90.19	1	1	$F0(x)$:=λx.if(flip(1/2), Fm2(ε), ε).<br>$F1(x)$:=λx.ε.<br>$F2(x)$:=λx.append(if(flip(1/2), pair(pair(ε, a), a), pair(ε, b)), F0(Fm1(ε))).<br>$F3(x)$:=λx.Fm2(ε).
Even	abcd	1h	8	100 (38)	-489.455	1	1	$F0(x)$:=λx.if(not(flip(5/12)), Fm3(ε), ε).<br>$F1(x)$:=λx.ε.<br>$F2(x)$:=λx.append(if(flip(3/8), pair(pair(ε, a), a), pair(ε, b)), F0(Fm1(ε))).<br>$F3(x)$:=λx.F2(ε).
Even	abcd	1h	8	1000 (171)	-5559.18	1	1	$F0(x)$:=λx.if(not(flip(5/12)), Fm3(ε), ε).<br>$F1(x)$:=λx.ε.<br>$F2(x)$:=λx.append(if(flip(3/8), pair(pair(ε, a), a), pair(ε, b)), F0(Fm1(ε))).<br>$F3(x)$:=λx.F2(ε).
Even	abcd	1h	8	10000 (729)	-51347.9	1	1	$F0(x)$:=λx.if(not(flip(3/8)), Fm2(ε), ε).<br>$F1(x)$:=λx.ε.<br>$F2(x)$:=λx.append(if(flip(5/12), pair(pair(ε, a), a), pair(ε, b)), F0(Fm1(ε))).<br>$F3(x)$:=λx.Fm2(ε).
Even	abcd	1h	8	100000 (2978)	-517641	1	1	$F0(x)$:=λx.if(not(flip(3/8)), Fm2(ε), ε).<br>$F1(x)$:=λx.ε.<br>$F2(x)$:=λx.append(if(flip(5/12), pair(pair(ε, a), a), pair(ε, b)), F0(Fm1(ε))).<br>$F3(x)$:=λx.Fm2(ε).
a^+ba^+	ab	2h	8	1 (1)	-13.4925	1	0.04	$F0(x)$:=λx.pair(pair(pair(ε, a), b), a).
a^+ba^+	ab	2h	8	10 (6)	-64.2217	1	1	$F0(x)$:=λx.pair(if(flip(1/2), if(empty(x), pair(Fm0(sample(Σ)), b), ε), F0(x)), a).
a^+ba^+	ab	2h	8	100 (23)	-327.196	1	1	$F0(x)$:=λx.pair(if(flip(1/2), if(empty(x), pair(Fm0(sample(Σ)), b), ε), F0(x)), a).
a^+ba^+	ab	2h	8	1000 (50)	-2830.67	1	1	$F0(x)$:=λx.pair(if(flip(1/2), if(empty(x), pair(Fm0(sample(Σ)), b), ε), F0(x)), a).
a^+ba^+	ab	2h	8	10000 (90)	-27736.5	1	1	$F0(x)$:=λx.pair(if(flip(1/2), if(empty(x), pair(Fm0(sample(Σ)), b), ε), F0(x)), a).
a^+ba^+	ab	2h	8	100000 (128)	-277586	1	1	$F0(x)$:=λx.pair(if(flip(1/2), if(empty(x), pair(Fm0(pair(ε, b)), b), ε), F0(x)), a).
a^+ba^+	ab	2h	8	1 (1)	-20.1171	1	0.04	$F0(x)$:=λx.ε.<br>$F1(x)$:=λx.pair(pair(pair(F0(ε), a), b), a).
a^+ba^+	ab	2h	8	10 (6)	-61.3143	1	1	$F0(x)$:=λx.pair(if(flip(1/2), F0(x), x), a).<br>$F1(x)$:=λx.Fm0(pair(Fm0(ε), b)).
a^+ba^+	ab	2h	8	100 (23)	-324.289	1	1	$F0(x)$:=λx.pair(if(flip(1/2), Fm0(x), x), a).<br>$F1(x)$:=λx.Fm0(pair(F0(ε), b)).
a^+ba^+	ab	2h	8	1000 (50)	-2827.76	1	1	$F0(x)$:=λx.pair(if(flip(1/2), Fm0(x), x), a).<br>$F1(x)$:=λx.Fm0(pair(F0(ε), b)).
a^+ba^+	ab	2h	8	10000 (90)	-27733.6	1	1	$F0(x)$:=λx.pair(if(flip(1/2), Fm0(x), x), a).<br>$F1(x)$:=λx.F0(pair(F0(ε), b)).
a^+ba^+	ab	2h	8	100000 (128)	-277583	1	1	$F0(x)$:=λx.pair(if(flip(1/2), Fm0(x), x), a).<br>$F1(x)$:=λx.F0(pair(F0(ε), b)).
a^+ba^+	ab	2h	8	1 (1)	-27.5526	1	0.04	$F0(x)$:=λx.pair(pair(pair(ε, a), b), a).<br>$F1(x)$:=λx.ε.<br>$F2(x)$:=λx.F0(F1(ε)).
a^+ba^+	ab	2h	8	10 (6)	-73.4128	1	1	$F0(x)$:=λx.append(F1(ε), pair(ε, b)).<br>$F1(x)$:=λx.pair(if(flip(1/2), Fm1(x), x), a).<br>$F2(x)$:=λx.F1(F0(ε)).
a^+ba^+	ab	2h	8	100 (23)	-332.535	1	1	$F0(x)$:=λx.pair(Fm1(ε), b).<br>$F1(x)$:=λx.pair(if(flip(1/2), Fm1(x), x), a).<br>$F2(x)$:=λx.F1(Fm0(ε)).
a^+ba^+	ab	2h	8	1000 (50)	-2836.01	1	1	$F0(x)$:=λx.pair(Fm1(ε), b).<br>$F1(x)$:=λx.pair(if(flip(1/2), Fm1(x), x), a).<br>$F2(x)$:=λx.F1(Fm0(ε)).
a^+ba^+	ab	2h	8	10000 (90)	-27741.8	1	1	$F0(x)$:=λx.pair(Fm1(ε), b).<br>$F1(x)$:=λx.pair(if(flip(1/2), Fm1(x), x), a).<br>$F2(x)$:=λx.F1(Fm0(ε)).
a^+ba^+	ab	2h	8	100000 (128)	-277591	1	1	$F0(x)$:=λx.pair(Fm1(ε), b).<br>$F1(x)$:=λx.pair(if(flip(1/2), Fm1(x), x), a).<br>$F2(x)$:=λx.F1(Fm0(ε)).
a^+ba^+	ab	2h	8	1 (1)	-35.4458	1	0.04	$F0(x)$:=λx.ε.<br>$F1(x)$:=λx.Fm0(ε).<br>$F2(x)$:=λx.pair(pair(F1(ε), a), b).<br>$F3(x)$:=λx.pair(Fm2(ε), a).

a^+ba^+	ab	2h	8	10 (6)	-78.0293	1	1	$F0(x)$:=λx.F2(ϵ).<br>$F1(x)$:=λx.ϵ.<br>$F2(x)$:=λx.pair(if(flip(1/2), Fm2(x), x), a).<br>$F3(x)$:=λx.Fm2(pair(Fm0(F1(ϵ)), b)).
a^+ba^+	ab	2h	8	100 (23)	-341.004	1	1	$F0(x)$:=λx.F2(ϵ).<br>$F1(x)$:=λx.ϵ.<br>$F2(x)$:=λx.pair(if(flip(1/2), Fm2(x), x), a).<br>$F3(x)$:=λx.Fm2(pair(Fm0(F1(ϵ)), b)).
a^+ba^+	ab	2h	8	1000 (50)	-2844.48	1	1	$F0(x)$:=λx.F2(ϵ).<br>$F1(x)$:=λx.ϵ.<br>$F2(x)$:=λx.pair(if(flip(1/2), Fm2(x), x), a).<br>$F3(x)$:=λx.Fm2(pair(Fm0(F1(ϵ)), b)).
a^+ba^+	ab	2h	8	10000 (90)	-27750.3	1	1	$F0(x)$:=λx.F2(ϵ).<br>$F1(x)$:=λx.ϵ.<br>$F2(x)$:=λx.pair(if(flip(1/2), Fm2(x), x), a).<br>$F3(x)$:=λx.Fm2(pair(Fm0(F1(ϵ)), b)).
a^+ba^+	ab	2h	8	100000 (128)	-277600	1	1	$F0(x)$:=λx.pair(if(flip(1/2), Fm0(x), x), a).<br>$F1(x)$:=λx.pair(Fm2(ϵ), b).<br>$F2(x)$:=λx.F0(ϵ).<br>$F3(x)$:=λx.F0(F1(x)).
$a^+(ba^+)^+$	ab	2d	8	1 (1)	-24.8038	1	0	$F0(x)$:=λx.pair(pair(pair(pair(pair(pair(ϵ, a), a), a), b), a), a).
$a^+(ba^+)^+$	ab	2d	8	10 (8)	-68.9696	0.56666	0.92	$F0(x)$:=λx.append(sample(Σ), if(flip(1/2), F0(ϵ), ϵ)).
$a^+(ba^+)^+$	ab	2d	8	100 (47)	-898.37	0.72	0.88	$F0(x)$:=λx.if(not(flip(11/24)), if(not(flip(if((x==head(x)), 1/24, 1/4))), pair(Fm0(pair(x, a)), a), pair(ϵ, a)), pair(if(flip(5/12), append(F0(if((x==pair(ϵ, b)), pair(pair(ϵ, a), a), append(pair(x, b), x))), x), if(flip(3/8), x, if(flip(1/4), pair(ϵ, a), ϵ))), b)).
$a^+(ba^+)^+$	ab	2d	8	1000 (154)	-7510.58	0.73076	0.88	$F0(x)$:=λx.if(not(flip(5/12)), if(not(flip(if((x==head(x)), 1/24, 1/4))), pair(Fm0(pair(x, a)), a), pair(ϵ, a)), pair(if(flip(11/24), append(F0(if((x==pair(ϵ, b)), x, append(pair(x, b), x))), x), if(flip(5/12), x, if(flip(7/24), pair(ϵ, a), ϵ))), b)).
$a^+(ba^+)^+$	ab	2d	8	10000 (346)	-71047.1	0.76	1	$F0(x)$:=λx.if(flip(1/2), pair(append(F0(pair(x, a)), if(not(flip(1/12)), ϵ, pair(pair(x, a), b))), a), append(head(if(not(flip(11/24)), sample(if(not(empty(x)), ϵ, Σ)), x)), append(sample(Σ), if(flip(1/2), pair(append(sample(if(not(flip(1/4)), (ϵ ∪ pair(append(pair(x, b), x), b)), Σ)), x), b), ϵ)))).
$a^+(ba^+)^+$	ab	2d	8	100000 (693)	-732861	0.76	1	$F0(x)$:=λx.if(flip(1/2), pair(append(F0(pair(x, a)), if(not(flip(1/12)), ϵ, pair(pair(x, a), b))), a), append(head(if(not(flip(11/24)), sample(if(not(empty(x)), ϵ, Σ)), x)), append(sample(Σ), if(flip(1/2), pair(append(sample(if(not(flip(1/4)), (ϵ ∪ pair(append(pair(x, b), x), b)), Σ)), x), b), ϵ)))).
$a^+(ba^+)^+$	ab	2d	8	1 (1)	-29.1259	1	0	$F0(x)$:=λx.append(pair(pair(x, a), b), x).<br>$F1(x)$:=λx.Fm0(pair(pair(ϵ, a), a)).
$a^+(ba^+)^+$	ab	2d	8	10 (8)	-75.3772	0.66666	0.96	$F0(x)$:=λx.if(flip(1/2), x, append(x, Fm0(sample(Σ)))).<br>$F1(x)$:=λx.append(F0(ϵ), Fm0(pair(ϵ, b))).
$a^+(ba^+)^+$	ab	2d	8	100 (47)	-637.128	1	1	$F0(x)$:=λx.if(flip(1/2), x, pair(F0(x), a)).<br>$F1(x)$:=λx.append(if(flip(11/24), F0(ϵ), F1(ϵ)), Fm0(pair(ϵ, b))).
$a^+(ba^+)^+$	ab	2d	8	1000 (154)	-4671.14	1	1	$F0(x)$:=λx.if(flip(1/2), x, pair(F0(x), a)).<br>$F1(x)$:=λx.append(if(not(flip(11/24)), F0(ϵ), F1(ϵ)), Fm0(pair(ϵ, b))).
$a^+(ba^+)^+$	ab	2d	8	10000 (346)	-46895.4	1	1	$F0(x)$:=λx.if(flip(1/2), x, pair(F0(x), a)).<br>$F1(x)$:=λx.append(if(flip(1/2), Fm0(ϵ), Fm1(ϵ)), Fm0(pair(ϵ, b))).
$a^+(ba^+)^+$	ab	2d	8	100000 (693)	-481521	1	1	$F0(x)$:=λx.if(flip(1/2), x, pair(F0(x), a)).<br>$F1(x)$:=λx.append(if(not(flip(11/24)), Fm0(ϵ), F1(x)), Fm0(pair(ϵ, b))).
$a^+(ba^+)^+$	ab	2d	8	1 (1)	-36.5614	1	0	$F0(x)$:=λx.append(pair(pair(x, a), b), x).<br>$F1(x)$:=λx.pair(pair(ϵ, a), a).<br>$F2(x)$:=λx.F0(F1(ϵ)).
$a^+(ba^+)^+$	ab	2d	8	10 (8)	-83.5034	0.61538	1	$F0(x)$:=λx.Fm1(Fm1(sample(Σ))).<br>$F1(x)$:=λx.append(x, if(flip(1/2), Fm1(sample(Σ)), ϵ)).<br>$F2(x)$:=λx.Fm0(ϵ).
$a^+(ba^+)^+$	ab	2d	8	100 (47)	-627.745	1	1	$F0(x)$:=λx.F1(ϵ).<br>$F1(x)$:=λx.if(flip(1/2), pair(F1(ϵ), a), ϵ).<br>$F2(x)$:=λx.if(or(empty(x), flip(1/2)), append(pair(Fm2(pair(ϵ, b)), b), Fm0(ϵ)), F1(ϵ)).
$a^+(ba^+)^+$	ab	2d	8	1000 (154)	-4691.33	1	1	$F0(x)$:=λx.F1(ϵ).<br>$F1(x)$:=λx.if(flip(1/2), pair(F0(ϵ), a), ϵ).<br>$F2(x)$:=λx.if(or(empty(x), flip(5/12)), append(pair(Fm2(pair(ϵ, a)), b), Fm1(ϵ)), Fm0(ϵ)).
$a^+(ba^+)^+$	ab	2d	8	10000 (346)	-46697.9	1	1	$F0(x)$:=λx.if(flip(1/2), pair(Fm0(x), a), ϵ).<br>$F1(x)$:=λx.append(pair(Fm0(x), b), if(flip(11/24), append(x, F1(x)), if(and(empty(x), or(flip(1/3), flip(1/2))), ϵ, x))).<br>$F2(x)$:=λx.Fm1(F0(ϵ)).
$a^+(ba^+)^+$	ab	2d	8	100000 (693)	-477910	1	1	$F0(x)$:=λx.if(flip(1/2), pair(Fm0(x), a), ϵ).<br>$F1(x)$:=λx.append(pair(Fm0(x), b), if(flip(11/24), append(x, F1(x)), if(and((ϵ==x), or(flip(1/3), flip(1/2))), ϵ, x))).<br>$F2(x)$:=λx.Fm1(F0(ϵ)).

$a^+(ba^+)^+$	ab	2d	8	1 (1)	-44.4546	1	0	$F0(x)$:=λx.ε.<br>$F1(x)$:=λx.append(pair(pair(x, a), b), x).<br>$F2(x)$:=λx.Fm1(pair(pair(Fm0(ε), a), a)).<br>$F3(x)$:=λx.Fm2(ε).
$a^+(ba^+)^+$	ab	2d	8	10 (8)	-91.7435	0.46428	0.76	$F0(x)$:=λx.Fm3(ε).<br>$F1(x)$:=λx.Fm2(if(flip(1/2), pair(Fm0(ε), a), sample(Σ))).<br>$F2(x)$:=λx.if(flip(1/3), pair(x, b), x).<br>$F3(x)$:=λx.Fm1(ε).
$a^+(ba^+)^+$	ab	2d	8	100 (47)	-627.233	1	1	$F0(x)$:=λx.if(flip(1/2), pair(F0(x), a), x).<br>$F1(x)$:=λx.Fm3(ε).<br>$F2(x)$:=λx.append(if(flip(1/2), F1(ε), F0(ε)), Fm0(pair(ε, b))).<br>$F3(x)$:=λx.Fm2(ε).
$a^+(ba^+)^+$	ab	2d	8	1000 (154)	-4846.26	1	1	$F0(x)$:=λx.sample(if(not(flip(1/4)), if(flip(1/3), pair(ε, a), x), pair(F2(Fm1(ε)), a))).<br>$F1(x)$:=λx.ε.<br>$F2(x)$:=λx.pair(F0(ε), a).<br>$F3(x)$:=λx.append(pair(if(not(flip(1/4)), if(flip(1/3), if(flip(1/24), x, if(flip(3/8), ε, ε)), Fm3(ε)), F2(ε)), b), Fm0(x)).
$a^+(ba^+)^+$	ab	2d	8	10000 (346)	-46690.3	1	1	$F0(x)$:=λx.append(F1(x), x).<br>$F1(x)$:=λx.pair(if(not(flip(1/2)), Fm0(x), Fm2(sample(if((ε==x), if(not(flip(1/12)), Σ, ε), x)))), b).<br>$F2(x)$:=λx.if(flip(1/2), pair(F2(ε), a), ε).<br>$F3(x)$:=λx.Fm0(Fm2(ε)).
$a^+(ba^+)^+$	ab	2d	8	100000 (693)	-477729	1	1	$F0(x)$:=λx.append(F1(x), x).<br>$F1(x)$:=λx.pair(if(not(flip(1/2)), Fm0(x), Fm2(sample(if((ε==x), if(not(flip(1/12)), Σ, ε), x)))), b).<br>$F2(x)$:=λx.if(flip(1/2), pair(F2(ε), a), ε).<br>$F3(x)$:=λx.Fm0(Fm2(ε)).
$a^*(ba^*)^+$	ab	2d	8	1 (1)	-17.2629	1	0.04	$F0(x)$:=λx.pair(pair(pair(pair(ε, a), a), b), a).
$a^*(ba^*)^+$	ab	2d	8	10 (9)	-133.62	0.60606	0.72	$F0(x)$:=λx.pair(if(flip(1/2), pair(append(F0(head(x)), x), b), if(not(flip(1/2)), ε, Fm0(pair(x, a)))), a).
$a^*(ba^*)^+$	ab	2d	8	100 (44)	-776.506	0.57692	1	$F0(x)$:=λx.pair(if(not(flip(1/3)), sample(if(not(flip(7/24)), Fm0(pair(pair(x, a), b)), ε)), pair(pair(if(flip(1/3), F0(pair(x, a)), if(not(flip(7/24)), ε, append(insert(x, if(flip(1/24), pair(ε, a), ε)), append(x, if(empty(x), pair(F0(ε), b), x))))), a), b)), a).
$a^*(ba^*)^+$	ab	2d	8	1000 (152)	-9310.72	0.48	1	$F0(x)$:=λx.if(flip(if((x==head(x)), 1/24, 5/12)), pair(append(x, if(not(flip(1/4)), ε, append(if(not(flip(11/24)), ε, append(x, sample(((x∪append(x, append(x, x)))∪ε)))), append(x, x)))), a), Fm0(if(flip(1/4), pair(pair(x, a), b), pair(x, a)))).
$a^*(ba^*)^+$	ab	2d	8	10000 (368)	-88323	0.48	1	$F0(x)$:=λx.if(flip(if((x==head(x)), 1/24, 5/12)), pair(append(x, if(not(flip(1/4)), ε, append(if(not(flip(11/24)), ε, append(x, sample(((x∪append(x, append(x, x)))∪ε)))), append(x, x)))), a), Fm0(if(flip(1/4), pair(pair(x, a), b), pair(x, a)))).
$a^*(ba^*)^+$	ab	2d	8	100000 (699)	-868532	0.48	1	$F0(x)$:=λx.if(flip(if((x==head(x)), 1/24, 5/12)), pair(append(x, if(not(flip(1/4)), ε, append(if(not(flip(11/24)), ε, append(x, sample(((x∪append(x, append(x, x)))∪ε)))), append(x, x)))), a), Fm0(if(flip(1/4), pair(pair(x, a), b), pair(x, a)))).
$a^*(ba^*)^+$	ab	2d	8	1 (1)	-23.8875	1	0.04	$F0(x)$:=λx.pair(ε, a).<br>$F1(x)$:=λx.pair(pair(pair(F0(ε), a), b), a).
$a^*(ba^*)^+$	ab	2d	8	10 (9)	-142.549	0.60714	1	$F0(x)$:=λx.if(flip(1/2), append(F0(x), pair(if(flip(1/2), ε, x), a)), pair(ε, a)).<br>$F1(x)$:=λx.F0(pair(if(flip(7/24), ε, pair(ε, a)), b)).
$a^*(ba^*)^+$	ab	2d	8	100 (44)	-468.536	1	1	$F0(x)$:=λx.pair(if(flip(1/2), ε, F0(ε)), a).<br>$F1(x)$:=λx.append(pair(Fm0(x), b), if(not(flip(1/2)), Fm0(pair(ε, a)), F1(pair(ε, a)))).
$a^*(ba^*)^+$	ab	2d	8	1000 (152)	-5205.14	1	1	$F0(x)$:=λx.pair(if(flip(1/2), ε, F0(append(x, x))), a).<br>$F1(x)$:=λx.append(pair(Fm0(x), b), if(not(flip(1/2)), Fm0(pair(ε, b)), F1(pair(ε, b)))).
$a^*(ba^*)^+$	ab	2d	8	10000 (368)	-50933.5	1	1	$F0(x)$:=λx.pair(if(flip(1/2), ε, F0(append(x, x))), a).<br>$F1(x)$:=λx.append(pair(Fm0(x), b), if(not(flip(1/2)), Fm0(pair(ε, b)), F1(pair(ε, b)))).
$a^*(ba^*)^+$	ab	2d	8	100000 (699)	-495034	1	1	$F0(x)$:=λx.pair(if(flip(1/2), ε, F0(ε)), a).<br>$F1(x)$:=λx.append(pair(Fm0(x), b), if(flip(1/2), Fm0(pair(ε, b)), F1(pair(ε, b)))).
$a^*(ba^*)^+$	ab	2d	8	1 (1)	-31.3231	1	0.04	$F0(x)$:=λx.pair(pair(pair(F1(ε), a), a), b).<br>$F1(x)$:=λx.ε.<br>$F2(x)$:=λx.pair(Fm0(ε), a).
$a^*(ba^*)^+$	ab	2d	8	10 (9)	-103.246	1	1	$F0(x)$:=λx.append(pair(x, b), Fm1(ε)).<br>$F1(x)$:=λx.pair(if(flip(1/2), F1(ε), ε), a).<br>$F2(x)$:=λx.Fm0(if(flip(1/2), F1(ε), F2(ε))).
$a^*(ba^*)^+$	ab	2d	8	100 (44)	-469.648	1	1	$F0(x)$:=λx.append(pair(x, b), Fm1(ε)).<br>$F1(x)$:=λx.pair(if(not(flip(1/2)), F1(ε), ε), a).<br>$F2(x)$:=λx.Fm0(if(flip(1/2), F1(ε), Fm2(ε))).
$a^*(ba^*)^+$	ab	2d	8	1000 (152)	-5275.69	1	1	$F0(x)$:=λx.append(pair(x, b), Fm1(ε)).<br>$F1(x)$:=λx.pair(if(flip(1/2), F1(ε), ε), a).<br>$F2(x)$:=λx.Fm0(if(flip(1/2), F1(ε), F2(ε))).
$a^*(ba^*)^+$	ab	2d	8	10000 (368)	-50276.6	1	1	$F0(x)$:=λx.pair(F1(ε), a).<br>$F1(x)$:=λx.append(if(flip(1/2), F0(ε), ε), ε).<br>$F2(x)$:=λx.append(append(Fm0(x), pair(ε, b)), sample(if(not(flip(11/24)), Fm0(pair(if(and(empty(x), flip(if(flip(1/2), 1/2, 5/12))), ε, ε), a)), Fm2(pair(ε, a))))).

$a^*(ba^*)^+$	ab	2d	8	100000 (699)	-489177	1	1	$F0(x)$:=λx.pair(F1(ε), a).<br>$F1(x)$:=λx.append(if(flip(1/2), F0(ε), ε), ε).<br>$F2(x)$:=λx.append(append(Fm0(x), pair(ε, b)), sample(if(not(flip(11/24)), Fm0(pair(if(and(empty(x), flip(if(flip(1/2), 1/2, 5/12))), ε, ε), a)), Fm2(pair(ε, a))))).
$a^*(ba^*)^+$	ab	2d	8	1 (1)	-39.2162	1	0.04	$F0(x)$:=λx.pair(pair(Fm1(ε), a), b).<br>$F1(x)$:=λx.pair(ε, a).<br>$F2(x)$:=λx.F0(ε).<br>$F3(x)$:=λx.pair(Fm2(ε), a).
$a^*(ba^*)^+$	ab	2d	8	10 (9)	-138.931	0.47222	0.6	$F0(x)$:=λx.append(x, if(flip(1/2), F2(ε), append(x, x))).<br>$F1(x)$:=λx.append(pair(ε, a), append(x, if(flip(1/2), ε, x))).<br>$F2(x)$:=λx.Fm1(F0(x)).<br>$F3(x)$:=λx.F2(pair(Fm0(ε), b)).
$a^*(ba^*)^+$	ab	2d	8	100 (44)	-490.075	1	1	$F0(x)$:=λx.sample(if(flip(1/2), Fm3(x), pair(Fm1(x), a))).<br>$F1(x)$:=λx.if(flip(1/2), x, pair(F1(x), a)).<br>$F2(x)$:=λx.pair(ε, b).<br>$F3(x)$:=λx.append(pair(Fm1(x), a), Fm0(Fm2(ε))).
$a^*(ba^*)^+$	ab	2d	8	1000 (152)	-5100.55	1	1	$F0(x)$:=λx.if(flip(1/2), Fm3(ε), F1(ε)).<br>$F1(x)$:=λx.pair(if(flip(if((head(x)==x), 1/2, 3/8)), ε, F1(append(x, x))), a).<br>$F2(x)$:=λx.ε.<br>$F3(x)$:=λx.append(append(F0(Fm2(ε)), pair(ε, b)), Fm1(pair(ε, a))).
$a^*(ba^*)^+$	ab	2d	8	10000 (368)	-50807	1	1	$F0(x)$:=λx.pair(append(x, if(not(flip(1/2)), ε, F2(pair(ε, a)))), a).<br>$F1(x)$:=λx.append(if(flip(1/2), F1(x), insert(pair(if(flip(1/4), pair(pair(F2(ε), a), a), ε), a), if(not(flip(7/24)), ε, pair(ε, a)))), x).<br>$F2(x)$:=λx.if(flip(1/2), x, Fm0(x)).<br>$F3(x)$:=λx.F1(F2(pair(pair(ε, b), a))).
$a^*(ba^*)^+$	ab	2d	8	100000 (699)	-489415	1	1	$F0(x)$:=λx.pair(if(flip(1/2), F0(ε), ε), a).<br>$F1(x)$:=λx.append(F0(ε), Fm2(ε)).<br>$F2(x)$:=λx.append(pair(if(flip(1/2), F2(append(x, pair(x, a))), if((x==ε), if(or((ε==pair(if(flip(11/24), ε, ε), a)), flip(3/8)), ε, ε), ε)), b), Fm0(ε)).<br>$F3(x)$:=λx.F1(ε).
$\Sigma^+aaa\Sigma^+$	abcd	2d	8	1 (1)	-26.0368	0.66666	0.12	$F0(x)$:=λx.pair(if(flip(1/2), Fm0(ε), ε), a).
$\Sigma^+aaa\Sigma^+$	abcd	2d	8	10 (10)	-142.803	0.60606	0.36	$F0(x)$:=λx.if(flip(1/2), pair(Fm0(pair(pair(head(x), a), a)), a), if(flip(1/2), x, pair(Fm0(pair(head(x), b)), b))).
$\Sigma^+aaa\Sigma^+$	abcd	2d	8	100 (83)	-1474.32	0.5	0.88	$F0(x)$:=λx.append(if(and(empty(x), flip(3/8)), ε, sample(((Σ\pair(ε, d))\pair(ε, c)))), if(flip(if((head(x)==x), 1/24, 1/2)), if(flip(7/24), pair(x, a), pair(if(flip(1/3), x, pair(if(flip(1/6), pair(head(x), b), x), b)), b)), F0(pair(x, a)))).
$\Sigma^+aaa\Sigma^+$	abcd	2d	8	1000 (521)	-16855.9	1	0.32	$F0(x)$:=λx.append(if(flip(1/2), pair(ε, b), pair(ε, a)), if(not(flip(7/24)), Fm0(ε), pair(pair(pair(pair(ε, a), a), a), b))).
$\Sigma^+aaa\Sigma^+$	abcd	2d	8	10000 (3033)	-173720	0.96	0.32	$F0(x)$:=λx.if(flip(if(empty(x), 1/24, 7/24)), pair(pair(pair(pair(ε, a), a), a), b), append(if(flip(if(empty(x), 1/2, 3/8)), pair(ε, b), pair(ε, a)), Fm0(append(pair(x, b), x)))).
$\Sigma^+aaa\Sigma^+$	abcd	2d	8	100000 (15681)	-1.72392e+06	0.96	0.32	$F0(x)$:=λx.if(flip(if(empty(x), 1/24, 7/24)), pair(pair(pair(head(pair(ε, a)), a), a), b), append(if(flip(if(empty(x), 1/2, 3/8)), pair(ε, b), pair(ε, a)), Fm0(append(x, pair(x, d))))).
$\Sigma^+aaa\Sigma^+$	abcd	2d	8	1 (1)	-31.8367	0.61904	0.12	$F0(x)$:=λx.pair(if(flip(1/2), ε, Fm0(ε)), a).<br>$F1(x)$:=λx.pair(F0(ε), b).
$\Sigma^+aaa\Sigma^+$	abcd	2d	8	10 (10)	-146.584	0.34615	0.8	$F0(x)$:=λx.if(flip(1/2), pair(if(flip(5/24), ε, append(F0(ε), x)), a), pair(if(flip(1/2), Fm0(pair(x, a)), ε), b)).<br>$F1(x)$:=λx.F0(pair(ε, a)).
$\Sigma^+aaa\Sigma^+$	abcd	2d	8	100 (83)	-1497.89	1	0.32	$F0(x)$:=λx.append(sample((pair(ε, b) ∪ x)), if(not(flip(7/24)), F1(ε), append(pair(x, a), pair(x, b)))).<br>$F1(x)$:=λx.Fm0(pair(ε, a)).
$\Sigma^+aaa\Sigma^+$	abcd	2d	8	1000 (521)	-16521.2	0.40740	0.48	$F0(x)$:=λx.if(not(flip(11/24)), pair(x, a), pair(if(not(flip(1/6)), x, pair(append(x, pair(pair(ε, a), a)), a)), b)).<br>$F1(x)$:=λx.Fm0(append(Fm0(F0(Fm0(if(not(flip(3/8)), pair(if(not(flip(5/24)), ε, pair(if(flip(1/2), Fm0(pair(ε, b)), if(flip(7/24), pair(ε, a), pair(if(flip(1/2), pair(pair(ε, a), b), ε), b))), a)), a), pair(ε, b))))), Fm0(ε))).
$\Sigma^+aaa\Sigma^+$	abcd	2d	8	10000 (3033)	-168109	0.62963	1	$F0(x)$:=λx.sample(if(flip(1/3), if(flip(1/3), pair(pair(x, b), a), pair(pair(x, a), a)), if(not(flip(3/8)), (append(x, pair(pair(ε, b), b)) ∪ pair(pair(x, a), b)), if(not(empty(x)), insert(x, pair(pair(ε, a), b)), ε)))).<br>$F1(x)$:=λx.F0(insert(pair(if(not(flip(3/8)), sample(((Σ\pair(ε, c))\pair(ε, d))), if(flip(1/2), ε, ε)), a), pair(if(flip(1/8), pair(ε, b), pair(if(flip(1/8), pair(ε, a), Fm0(if(not(flip(5/12)), ε, Fm0(ε)))), a)), a))).
$\Sigma^+aaa\Sigma^+$	abcd	2d	8	100000 (15681)	-1.65997e+06	0.62963	1	$F0(x)$:=λx.sample(if(flip(1/3), if(flip(1/3), pair(pair(x, b), a), pair(pair(x, a), a)), if(not(flip(3/8)), (append(x, pair(pair(ε, b), b)) ∪ pair(pair(x, a), b)), if(not((x==ε)), insert(x, pair(pair(ε, a), b)), x)))).<br>$F1(x)$:=λx.F0(insert(pair(if(not(flip(3/8)), sample(((Σ\pair(append(ε, ε), d))\pair(ε, c))), if(flip(1/2), ε, ε)), a), pair(if(flip(1/8), pair(x, b), pair(if(flip(1/8), pair(ε, a), Fm0(if(not(flip(5/12)), ε, Fm0(ε)))), a)), a))).
$\Sigma^+aaa\Sigma^+$	abcd	2d	8	1 (1)	-39.334	1	0.04	$F0(x)$:=λx.ε.<br>$F1(x)$:=λx.append(pair(x, a), pair(x, b)).<br>$F2(x)$:=λx.F1(pair(pair(F0(ε), a), a)).
$\Sigma^+aaa\Sigma^+$	abcd	2d	8	10 (10)	-152.344	0.88461	0.72	$F0(x)$:=λx.append(sample((x ∪ Fm1(x))), F1(if(flip(1/2), F1(x), ε))).<br>$F1(x)$:=λx.append(sample((Σ\(pair(ε, d) ∪ pair(ε, c)))), x).<br>$F2(x)$:=λx.Fm0(pair(pair(pair(if(flip(1/2), ε, F0(ε)), a), a), a)).

$\Sigma^+aaa\Sigma^+$	abcd	2d	8	100 (83)	-1467.55	1	0.88	$F0(x)$:=λx.append(if(flip(1/2), if(flip(1/2), pair(if(flip(1/2), pair(ε, b), ε), a), ε), pair(sample(if(flip(1/2), x, (ε∪append(pair(ε, a), pair(ε, b))))), b)), if(flip(7/24), pair(x, b), x)).<br>$F1(x)$:=λx.append(if(flip(1/24), insert(x, pair(x, b)), pair(if(flip(1/2), ε, pair(sample((if(flip(1/2), head(x), pair(ε, b))∪pair(x, a))), a)), a)), append(append(head(append(x, ε)), x), sample(if(flip(1/4), x, pair(ε, b))))).<br>$F2(x)$:=λx.append(Fm0(sample(((Σ\pair(ε, c))\pair(x, d)))), F1(if(flip(7/24), pair(pair(ε, a), b), pair(ε, a)))).
$\Sigma^+aaa\Sigma^+$	abcd	2d	8	1000 (521)	-16137.8	0.92	0.88	$F0(x)$:=λx.if(not(flip(if(empty(x), 3/8, 1/2))), x, pair(x, b)).<br>$F1(x)$:=λx.if(flip(11/24), Fm0(if(not(flip(11/24)), pair(x, b), pair(pair(x, a), a))), pair(append(sample(if(flip(1/2), (pair(pair(ε, b), b)∪ε), if(flip(1/2), (append(pair(ε, a), head(x))∪pair(pair(ε, b), a)), pair(ε, a)))), x), a)).<br>$F2(x)$:=λx.Fm1(pair(Fm1(pair(if(not(flip(5/12)), pair(sample(if(flip(11/24), if(flip(1/2), ε, pair(ε, a)), F0(ε))), a), pair(F0(ε), b)), a)), a)).
$\Sigma^+aaa\Sigma^+$	abcd	2d	8	10000 (3033)	-165396	0.44	0.72	$F0(x)$:=λx.append(x, if(not(flip(1/6)), sample((pair(ε, b)∪pair(ε, a))), Fm1(pair(ε, a)))).<br>$F1(x)$:=λx.pair(append(x, x), a).<br>$F2(x)$:=λx.if((head(x)==x), F0(Fm2(F0(x))), if(flip(1/8), head(x), F0(Fm0(x)))).
$\Sigma^+aaa\Sigma^+$	abcd	2d	8	100000 (15681)	-1.64391e+06	0.44	0.72	$F0(x)$:=λx.append(x, if(not(flip(1/6)), sample((pair(ε, b)∪pair(insert(ε, ε), a))), Fm1(pair(ε, a)))).<br>$F1(x)$:=λx.pair(append(x, x), a).<br>$F2(x)$:=λx.if((head(x)==x), F0(Fm2(F0(x))), if(flip(1/8), head(x), F0(Fm0(x)))).
$\Sigma^+aaa\Sigma^+$	abcd	2d	8	1 (1)	-47.2272	1	0.04	$F0(x)$:=λx.pair(ε, a).<br>$F1(x)$:=λx.append(pair(x, a), pair(x, b)).<br>$F2(x)$:=λx.pair(Fm0(ε), a).<br>$F3(x)$:=λx.F1(F2(ε)).
$\Sigma^+aaa\Sigma^+$	abcd	2d	8	10 (10)	-154.941	0.65384	0.68	$F0(x)$:=λx.if(flip(1/2), F1(ε), pair(ε, b)).<br>$F1(x)$:=λx.insert(x, pair(sample(if(flip(1/2), ε, Fm0(ε))), a)).<br>$F2(x)$:=λx.append(if(flip(1/2), x, append(F0(x), pair(x, b))), if(flip(1/2), x, ε)).<br>$F3(x)$:=λx.F1(Fm2(Fm1(F1(ε)))).
$\Sigma^+aaa\Sigma^+$	abcd	2d	8	100 (83)	-1491.03	0.76923	0.72	$F0(x)$:=λx.if(not(flip(1/12)), F3(ε), append(x, x)).<br>$F1(x)$:=λx.pair(insert(x, if(not(flip(3/8)), head(x), if(flip(1/2), pair(x, a), pair(ε, b)))), b).<br>$F2(x)$:=λx.append(sample(((Σ\pair(ε, c))\pair(ε, d))), if(not(flip(1/3)), Fm0(pair(pair(ε, a), a)), Fm1(pair(pair(ε, a), a)))).<br>$F3(x)$:=λx.Fm2(ε).
$\Sigma^+aaa\Sigma^+$	abcd	2d	8	1000 (521)	-16596.5	0.5	1	$F0(x)$:=λx.append(F1(ε), append(x, F1(ε))).<br>$F1(x)$:=λx.sample((pair(ε, a)∪pair(ε, b))).<br>$F2(x)$:=λx.sample(if(flip(1/6), if((x==if(flip(1/2), pair(ε, a), x)), if(not(flip(5/24)), Fm0(x), append(insert(x, pair(pair(ε, b), b)), x)), x), if(flip(1/3), ε, pair(F2(pair(ε, b)), a)))).<br>$F3(x)$:=λx.F0(Fm0(pair(Fm2(pair(ε, a)), a))).
$\Sigma^+aaa\Sigma^+$	abcd	2d	8	10000 (3033)	-170247	0.53846	0.48	$F0(x)$:=λx.Fm2(Fm1(x)).<br>$F1(x)$:=λx.if(flip(5/12), pair(x, b), pair(x, a)).<br>$F2(x)$:=λx.Fm1(append(x, if(not(flip(5/24)), ε, pair(pair(ε, a), a)))).<br>$F3(x)$:=λx.Fm2(F0(Fm0(Fm1(ε)))).
$\Sigma^+aaa\Sigma^+$	abcd	2d	8	100000 (15681)	-1.69488e+06	0.53846	0.48	$F0(x)$:=λx.Fm2(Fm1(x)).<br>$F1(x)$:=λx.if(flip(5/12), pair(x, b), pair(x, a)).<br>$F2(x)$:=λx.Fm1(append(x, if(not(flip(5/24)), ε, pair(pair(ε, a), a)))).<br>$F3(x)$:=λx.Fm2(F0(Fm0(Fm1(x)))).
Count(a,x) $\geq$ 2	ab	1d	8	1 (1)	-34.3386	0.25806	0.04	$F0(x)$:=λx.pair(pair(pair(if(flip(1/2), append(F0(ε), sample(Σ)), ε), a), a), b).
Count(a,x) $\geq$ 2	ab	1d	8	10 (10)	-119.333	0.86666	0.64	$F0(x)$:=λx.append(sample(Σ), if(flip(7/24), pair(ε, a), Fm0(ε))).
Count(a,x) $\geq$ 2	ab	1d	8	100 (63)	-837.066	0.57142	1	$F0(x)$:=λx.append(sample(Σ), if(flip(7/24), sample(Σ), Fm0(ε))).
Count(a,x) $\geq$ 2	ab	1d	8	1000 (287)	-7065.51	1	1	$F0(x)$:=λx.if(not(flip(1/3)), pair(if(and(not(empty(x)), flip(1/2)), if(flip(3/8), pair(if(flip(1/6), pair(x, b), if(flip(5/12), head(x), ε)), b), ε), F0(pair(x, b))), a), pair(F0(x), b)).
Count(a,x) $\geq$ 2	ab	1d	8	10000 (1314)	-70428.3	1	1	$F0(x)$:=λx.if(not(flip(1/3)), pair(if(and(not((ε==x)), flip(1/2)), if(flip(1/3), pair(if(flip(1/12), append(if(flip(1/2), ε, x), pair(pair(ε, b), b)), if(flip(3/8), head(x), ε)), b), ε), F0(pair(x, b))), a), pair(F0(x), b)).
Count(a,x) $\geq$ 2	ab	1d	8	100000 (5854)	-710977	1	1	$F0(x)$:=λx.if(not(flip(1/3)), pair(if(and(not((ε==x)), flip(1/2)), if(flip(1/3), pair(if(flip(1/12), append(if(flip(1/2), ε, x), pair(pair(ε, b), b)), if(flip(3/8), head(x), ε)), b), ε), F0(pair(x, b))), a), pair(F0(x), b)).
Count(a,x) $\geq$ 2	ab	1d	8	1 (1)	-37.4415	1	0	$F0(x)$:=λx.append(append(pair(x, b), pair(x, a)), x).<br>$F1(x)$:=λx.Fm0(pair(pair(pair(ε, a), a), b)).
Count(a,x) $\geq$ 2	ab	1d	8	10 (10)	-126.65	0.86666	0.64	$F0(x)$:=λx.pair(ε, a).<br>$F1(x)$:=λx.append(sample(Σ), if(flip(7/24), F0(ε), F1(ε))).
Count(a,x) $\geq$ 2	ab	1d	8	100 (63)	-832.191	0.71875	1	$F0(x)$:=λx.insert(sample(Σ), if(flip(1/2), append(x, if(flip(1/2), pair(sample(Σ), b), pair(sample((x∪Σ)), a))), x)).<br>$F1(x)$:=λx.Fm0(F0(sample((pair(ε, a)∪if(flip(5/24), Σ, ε))))).
Count(a,x) $\geq$ 2	ab	1d	8	1000 (287)	-7115.13	0.96	1	$F0(x)$:=λx.pair(sample(if(not(flip(if(empty(x), 1/24, 3/8))), Fm1(x), (Σ\(x∪pair(x, a))))), b).<br>$F1(x)$:=λx.if(flip(if(empty(x), 3/8, 5/12)), F0(x), pair(if(not(flip(if((ε==x), 5/24, 11/24))), Fm1(pair(x, b)), sample((Σ\pair(x, b)))), a)).
Count(a,x) $\geq$ 2	ab	1d	8	10000 (1314)	-71216.1	0.96	1	$F0(x)$:=λx.pair(sample(if(not(flip(if(empty(x), 1/24, 3/8))), Fm1(x), (Σ\(x∪pair(x, a))))), b).<br>$F1(x)$:=λx.if(flip(if(empty(x), 3/8, 5/12)), F0(x), pair(if(not(flip(if((ε==x), 5/24, 11/24))), Fm1(pair(x, b)), sample((Σ\pair(x, b)))), a)).
Count(a,x) $\geq$ 2	ab	1d	8	100000 (5854)	-716874	0.96	1	$F0(x)$:=λx.pair(sample(if(not(flip(if(empty(x), 1/24, 3/8))), Fm1(x), (Σ\(x∪pair(x, a))))), b).<br>$F1(x)$:=λx.if(flip(if(empty(x), 3/8, 5/12)), F0(x), pair(if(not(flip(if((ε==x), 5/24, 11/24))), Fm1(pair(x, b)), sample((Σ\pair(x, b)))), a)).

Count(a,x) ≥ 2	ab	1d	8	1 (1)	-44.8771	1	0	F0(x):=λx.pair(ε, a).<br>F1(x):=λx.append(pair(x, b), append(pair(x, a), x)).<br>F2(x):=λx.F1(pair(pair(F0(ε), a), b)).
Count(a,x) ≥ 2	ab	1d	8	10 (10)	-134.491	0.86666	0.64	F0(x):=λx.pair(ε, a).<br>F1(x):=λx.Fm2(ε).<br>F2(x):=λx.append(sample(Σ), if(flip(7/24), F0(ε), Fm1(ε))).
Count(a,x) ≥ 2	ab	1d	8	100 (63)	-837.077	0.57142	1	F0(x):=λx.append(append(append(sample(if(flip(1/2), if(flip(1/12), append(pair(ε, a), pair(x, a)), Σ), ε)), Fm1(ε)), sample(Σ)), x).<br>F1(x):=λx.if(not(flip(1/4)), ε, sample(Σ)).<br>F2(x):=λx.Fm0(F0(append(sample(if(flip(1/3), ε, Σ)), Fm1(ε)))).
Count(a,x) ≥ 2	ab	1d	8	1000 (287)	-7206.31	1	1	F0(x):=λx.append(pair(if(flip(1/6), insert(pair(ε, a), append(sample((Σ ∪ ε)), ε)), if(flip(3/8), pair(if(flip(1/12), pair(head(pair(x, a)), a), if(flip(1/2), ε, sample(Σ))), b), if(not(flip(1/12)), ε, pair(append(sample((pair(sample(Σ), b) ∪ Σ)), pair(ε, b)), b)))), a), x).<br>F1(x):=λx.F0(sample(if(flip(1/2), if(not(flip(1/4)), Σ, insert(x, if(flip(1/6), pair(pair(if(flip(1/3), ε, x), b), b), x))), ε))).<br>F2(x):=λx.Fm0(F1(pair(ε, b))).
Count(a,x) ≥ 2	ab	1d	8	10000 (1314)	-71740.2	1	1	F0(x):=λx.append(pair(if(flip(1/6), insert(pair(ε, a), append(sample((Σ ∪ ε)), ε)), if(flip(3/8), pair(if(flip(1/12), pair(head(pair(x, b)), a), if(not(flip(11/24)), ε, sample(Σ))), b), if(not(flip(1/8)), ε, pair(append(sample((pair(sample(Σ), b) ∪ Σ)), pair(ε, b)), b)))), a), x).<br>F1(x):=λx.F0(sample(if(flip(1/2), if(not(flip(1/4)), Σ, insert(x, if(flip(1/6), pair(pair(if(flip(7/24), ε, x), b), b), x))), ε))).<br>F2(x):=λx.Fm0(F1(pair(x, b))).
Count(a,x) ≥ 2	ab	1d	8	100000 (5854)	-722432	1	1	F0(x):=λx.append(pair(if(flip(1/6), insert(pair(ε, a), append(sample((Σ ∪ ε)), ε)), if(flip(3/8), pair(if(flip(1/12), pair(head(pair(x, b)), a), if(not(flip(11/24)), ε, sample(Σ))), b), if(not(flip(1/8)), ε, pair(append(sample((pair(sample(Σ), b) ∪ Σ)), pair(ε, b)), b)))), a), x).<br>F1(x):=λx.F0(sample(if(flip(1/2), if(not(flip(1/4)), Σ, insert(x, if(flip(1/6), pair(pair(if(flip(7/24), ε, x), b), b), x))), ε))).<br>F2(x):=λx.Fm0(F1(pair(x, b))).
Count(a,x) ≥ 2	ab	1d	8	1 (1)	-52.7702	1	0	F0(x):=λx.F1(pair(pair(pair(ε, a), a), b)).<br>F1(x):=λx.append(append(pair(x, b), pair(x, a)), x).<br>F2(x):=λx.ε.<br>F3(x):=λx.Fm0(Fm2(ε)).
Count(a,x) ≥ 2	ab	1d	8	10 (10)	-142.6	0.86666	0.64	F0(x):=λx.append(if(flip(1/4), ε, Fm0(F1(ε))), sample(Σ)).<br>F1(x):=λx.Fm2(ε).<br>F2(x):=λx.ε.<br>F3(x):=λx.pair(Fm0(ε), a).
Count(a,x) ≥ 2	ab	1d	8	100 (63)	-850.103	0.86842	0.96	F0(x):=λx.sample((pair(x, a) ∪ append(append(sample((ε ∪ pair(ε, a))), append(x, append(sample(Σ), sample(Σ)))), sample((Σ\pair(x, b)))))).<br>F1(x):=λx.append(append(Fm0(x), sample(Σ)), sample(((Σ ∪ F2(ε)) ∪ append(x, pair(x, b))))).<br>F2(x):=λx.ε.<br>F3(x):=λx.Fm1(sample((Σ ∪ ε))).
Count(a,x) ≥ 2	ab	1d	8	1000 (287)	-7045.25	1	1	F0(x):=λx.F1(ε).<br>F1(x):=λx.if(empty(F2(ε)), pair(x, a), Fm1(pair(x, b))).<br>F2(x):=λx.if(flip(3/8), pair(ε, a), ε).<br>F3(x):=λx.if(not(flip(5/12)), append(Fm3(ε), sample(Σ)), F1(Fm0(ε))).
Count(a,x) ≥ 2	ab	1d	8	10000 (1314)	-69961	1	1	F0(x):=λx.F1(ε).<br>F1(x):=λx.if(empty(F2(ε)), pair(x, a), Fm1(pair(x, b))).<br>F2(x):=λx.if(flip(3/8), pair(ε, a), ε).<br>F3(x):=λx.if(not(flip(5/12)), append(Fm3(ε), sample(Σ)), F1(Fm0(ε))).
Count(a,x) ≥ 2	ab	1d	8	100000 (5854)	-706290	1	1	F0(x):=λx.F1(ε).<br>F1(x):=λx.if(empty(F2(ε)), pair(x, a), Fm1(pair(x, b))).<br>F2(x):=λx.if(flip(3/8), pair(ε, a), x).<br>F3(x):=λx.if(not(flip(5/12)), append(Fm3(ε), sample(Σ)), F1(Fm0(ε))).
Count(a,x) even	ab	1d	8	1 (1)	-13.4925	1	0.04	F0(x):=λx.pair(pair(pair(ε, a), b), a).
Count(a,x) even	ab	1d	8	10 (7)	-83.3823	0.5	1	F0(x):=λx.append(if(flip(1/3), ε, F0(ε)), sample(Σ)).
Count(a,x) even	ab	1d	8	100 (31)	-477.086	1	1	F0(x):=λx.sample(if(flip(1/3), if(and(empty(x), flip(5/24)), pair(pair(ε, a), a), pair(Fm0(if(empty(x), pair(ε, a), ε)), a)), pair(if(flip(5/12), F0(x), x), b))).
Count(a,x) even	ab	1d	8	1000 (113)	-3934.03	1	1	F0(x):=λx.if(and(empty(x), flip(1/3)), pair(append(pair(if(flip(1/8), F0(pair(ε, b)), if(flip(5/24), pair(ε, b), ε)), a), sample(if(not(flip(5/12)), ε, F0(ε)))), a), pair(if(flip(11/24), Fm0(x), x), b)).
Count(a,x) even	ab	1d	8	10000 (579)	-39647	1	1	F0(x):=λx.if(and(empty(x), flip(1/3)), pair(append(pair(if(flip(1/8), F0(pair(ε, b)), if(flip(1/4), pair(ε, b), ε)), a), sample(if(not(flip(5/12)), ε, F0(ε)))), a), pair(if(flip(11/24), Fm0(x), x), b)).
Count(a,x) even	ab	1d	8	100000 (2559)	-398961	1	1	F0(x):=λx.if(and((ε==x), flip(1/3)), pair(append(pair(if(flip(1/8), F0(pair(ε, b)), if(flip(1/4), pair(ε, b), ε)), a), sample(if(not(flip(5/12)), ε, F0(ε)))), a), pair(if(flip(11/24), Fm0(x), x), b)).
Count(a,x) even	ab	1d	8	1 (1)	-20.1171	1	0.04	F0(x):=λx.ε.<br>F1(x):=λx.pair(pair(pair(F0(ε), a), b), a).
Count(a,x) even	ab	1d	8	10 (7)	-85.5641	0.59259	0.6	F0(x):=λx.if(flip(1/2), x, insert(Fm0(sample(Σ)), x)).<br>F1(x):=λx.Fm0(Fm0(pair(ε, b))).
Count(a,x) even	ab	1d	8	100 (31)	-521.948	0.51724	1	F0(x):=λx.append(sample(Σ), if(flip(1/3), sample(Σ), Fm0(ε))).<br>F1(x):=λx.if(flip(1/3), pair(ε, b), F0(ε)).

Count(a,x) even	ab	1d	8	1000 (113)	-3967.17	1	1	F0(x):=λx.if(empty(x), pair(ϵ, a), ϵ).<br>F1(x):=λx.if(not(flip(3/8)), pair(if(flip(1/2), x, F1(x)), b), pair(sample(if(and(empty(x), flip(1/3)), pair(ϵ, a), Fm1(F0(x)))), a)).
Count(a,x) even	ab	1d	8	10000 (579)	-39828.3	1	1	F0(x):=λx.if(empty(x), pair(ϵ, a), ϵ).<br>F1(x):=λx.if(not(flip(3/8)), pair(if(flip(1/2), x, F1(x)), b), pair(sample(if(and(empty(x), flip(1/3)), pair(ϵ, a), Fm1(F0(x)))), a)).
Count(a,x) even	ab	1d	8	100000 (2559)	-400666	1	1	F0(x):=λx.if((x==ϵ), pair(ϵ, a), ϵ).<br>F1(x):=λx.if(not(flip(3/8)), pair(if(flip(1/2), x, F1(x)), b), pair(sample(if(and(empty(x), flip(1/3)), pair(ϵ, a), Fm1(F0(x)))), a)).
Count(a,x) even	ab	1d	8	1 (1)	-27.5526	1	0.04	F0(x):=λx.ϵ.<br>F1(x):=λx.pair(F0(ϵ), a).<br>F2(x):=λx.pair(pair(Fm1(ϵ), b), a).
Count(a,x) even	ab	1d	8	10 (7)	-94.1985	0.51612	0.48	F0(x):=λx.F1(pair(ϵ, b)).<br>F1(x):=λx.if(flip(1/3), x, insert(Fm1(sample(Σ)), x)).<br>F2(x):=λx.Fm0(ϵ).
Count(a,x) even	ab	1d	8	100 (31)	-499.105	1	1	F0(x):=λx.sample(if(flip(1/3), pair(F0(if(empty(x), pair(ϵ, a), ϵ)), a), if(flip(1/2), (Σ\(x∪pair(x, b))), Fm1(x)))).<br>F1(x):=λx.pair(Fm0(x), b).<br>F2(x):=λx.Fm0(pair(ϵ, a)).
Count(a,x) even	ab	1d	8	1000 (113)	-3930	1	1	F0(x):=λx.sample(if(flip(3/8), x, if(flip(1/2), pair(Fm0(x), b), pair(F2(x), a)))).<br>F1(x):=λx.sample((Σ\head(pair(x, a)))).<br>F2(x):=λx.F0(Fm1(x)).
Count(a,x) even	ab	1d	8	10000 (579)	-39441.1	1	1	F0(x):=λx.sample(if(flip(1/3), x, if(flip(1/2), pair(Fm0(x), b), pair(F2(x), a)))).<br>F1(x):=λx.sample((Σ\head(pair(x, a)))).<br>F2(x):=λx.F0(F1(x)).
Count(a,x) even	ab	1d	8	100000 (2559)	-396869	1	1	F0(x):=λx.sample(if(flip(1/3), x, if(flip(1/2), pair(Fm0(x), b), pair(F2(x), a)))).<br>F1(x):=λx.sample((Σ\head(pair(x, a)))).<br>F2(x):=λx.F0(F1(x)).
Count(a,x) even	ab	1d	8	1 (1)	-35.4458	1	0.04	F0(x):=λx.pair(Fm2(ϵ), b).<br>F1(x):=λx.ϵ.<br>F2(x):=λx.pair(Fm1(ϵ), a).<br>F3(x):=λx.pair(Fm0(ϵ), a).
Count(a,x) even	ab	1d	8	10 (7)	-98.6572	0.54838	0.28	F0(x):=λx.insert(Fm1(F2(ϵ)), pair(ϵ, b)).<br>F1(x):=λx.append(x, if(flip(1/2), F1(sample(Σ)), x)).<br>F2(x):=λx.ϵ.<br>F3(x):=λx.F0(ϵ).
Count(a,x) even	ab	1d	8	100 (31)	-530.985	0.51724	1	F0(x):=λx.sample(Σ).<br>F1(x):=λx.pair(ϵ, b).<br>F2(x):=λx.if(flip(3/8), x, append(Fm2(Fm0(ϵ)), sample(Σ))).<br>F3(x):=λx.F2(F1(ϵ)).
Count(a,x) even	ab	1d	8	1000 (113)	-4162.78	1	1	F0(x):=λx.append(pair(if(flip(1/3), Fm3(x), F2(ϵ)), a), x).<br>F1(x):=λx.Fm0(Fm0(ϵ)).<br>F2(x):=λx.ϵ.<br>F3(x):=λx.if(not(flip(7/24)), pair(if(flip(5/12), Fm3(ϵ), ϵ), b), Fm1(ϵ)).
Count(a,x) even	ab	1d	8	10000 (579)	-39457.3	1	1	F0(x):=λx.x.<br>F1(x):=λx.Fm3(if(empty(x), pair(ϵ, b), ϵ)).<br>F2(x):=λx.x.<br>F3(x):=λx.if(flip(1/3), pair(F1(Fm0(Fm2(x))), a), sample(if(flip(1/2), pair(F3(x), b), (Σ\head(pair(x, a)))))).
Count(a,x) even	ab	1d	8	100000 (2559)	-396886	1	1	F0(x):=λx.x.<br>F1(x):=λx.Fm3(if(empty(x), pair(x, b), ϵ)).<br>F2(x):=λx.x.<br>F3(x):=λx.if(flip(1/3), pair(F1(Fm0(Fm2(x))), a), sample(if(flip(1/2), pair(F3(x), b), (Σ\head(pair(x, a)))))).
$a\Sigma^+b$	abcd	1h	8	1 (1)	-20.0355	1	0.04	F0(x):=λx.pair(pair(pair(pair(ϵ, a), b), b), b).
$a\Sigma^+b$	abcd	1h	8	10 (6)	-85.3092	0.53571	0.36	F0(x):=λx.pair(append(pair(if(flip(1/2), pair(ϵ, a), x), a), if(flip(1/2), Fm0(pair(ϵ, b)), sample((ϵ∪Σ)))), b).
$a\Sigma^+b$	abcd	1h	8	100 (39)	-674.025	0.5	1	F0(x):=λx.if(flip(3/8), pair(F0(ϵ), b), pair(if(flip(5/12), ϵ, Fm0(ϵ)), a)).
$a\Sigma^+b$	abcd	1h	8	1000 (159)	-5715.61	0.96428	0.96	F0(x):=λx.pair(if(flip(1/3), F0(ϵ), insert(if(not(flip(1/4)), ϵ, pair(ϵ, a)), pair(if(not(flip(1/4)), if(flip(5/24), pair(pair(if(flip(5/24), F0(ϵ), x), a), a), x), Fm0(x)), a))), b).
$a\Sigma^+b$	abcd	1h	8	10000 (763)	-59101.3	0.96296	0.96	F0(x):=λx.pair(if(flip(1/3), F0(ϵ), insert(if(not(flip(1/4)), ϵ, pair(ϵ, a)), pair(if(not(flip(1/4)), if(flip(1/6), pair(pair(if(flip(5/24), F0(ϵ), ϵ), a), a), ϵ), F0(ϵ)), a))), b).
$a\Sigma^+b$	abcd	1h	8	100000 (3222)	-588583	0.96296	0.96	F0(x):=λx.pair(if(flip(1/3), F0(ϵ), insert(if(not(flip(1/4)), ϵ, pair(ϵ, a)), pair(if(not(flip(1/4)), if(flip(1/6), pair(pair(if(flip(5/24), F0(ϵ), ϵ), a), a), ϵ), F0(ϵ)), a))), b).
$a\Sigma^+b$	abcd	1h	8	1 (1)	-26.6602	1	0.04	F0(x):=λx.pair(pair(ϵ, a), b).<br>F1(x):=λx.pair(pair(F0(ϵ), b), b).
$a\Sigma^+b$	abcd	1h	8	10 (6)	-81.7364	0.96	1	F0(x):=λx.if(flip(5/12), pair(Fm0(ϵ), a), if(flip(1/2), pair(ϵ, a), F1(ϵ))).<br>F1(x):=λx.pair(F0(ϵ), b).
$a\Sigma^+b$	abcd	1h	8	100 (39)	-570.099	1	0.68	F0(x):=λx.if(flip(7/24), pair(x, b), pair(if(flip(1/2), x, Fm0(x)), a)).<br>F1(x):=λx.pair(Fm0(if(flip(1/3), Fm1(ϵ), pair(ϵ, a))), b).

$a\Sigma^+b$	abcd	1h	8	1000 (159)	-5320.55	0.96774	1	$F0(x)$:=λx.if(not(flip(7/24)), Fm1(x), pair(x, b)).<br>$F1(x)$:=λx.Fm0(if(and(not(empty(x)), flip(1/2)), pair(x, b), pair(x, a))).
$a\Sigma^+b$	abcd	1h	8	10000 (763)	-55068.5	0.96774	1	$F0(x)$:=λx.if(not(flip(7/24)), Fm1(x), pair(x, b)).<br>$F1(x)$:=λx.Fm0(if(and(not(empty(x)), flip(1/2)), pair(x, b), pair(x, a))).
$a\Sigma^+b$	abcd	1h	8	100000 (3222)	-544026	0.96774	1	$F0(x)$:=λx.if(not(flip(7/24)), Fm1(x), pair(x, b)).<br>$F1(x)$:=λx.Fm0(if(and(not((x==ϵ)), flip(1/2)), pair(x, b), pair(x, a))).
$a\Sigma^+b$	abcd	1h	8	1 (1)	-34.0957	1	0.04	$F0(x)$:=λx.pair(pair(F1(ϵ), b), b).<br>$F1(x)$:=λx.pair(pair(ϵ, a), b).<br>$F2(x)$:=λx.Fm0(ϵ).
$a\Sigma^+b$	abcd	1h	8	10 (6)	-98.6195	0.5	0.76	$F0(x)$:=λx.append(if(flip(5/24), pair(pair(F1(ϵ), a), b), pair(x, a)), sample(if(flip(1/2), Fm0(ϵ), Σ))).<br>$F1(x)$:=λx.ϵ.<br>$F2(x)$:=λx.pair(F0(ϵ), b).
$a\Sigma^+b$	abcd	1h	8	100 (39)	-558.432	1	1	$F0(x)$:=λx.append(if(flip(1/2), pair(x, b), pair(x, a)), sample(if(not(flip(5/12)), Fm0(Fm1(x)), ϵ))).<br>$F1(x)$:=λx.ϵ.<br>$F2(x)$:=λx.pair(Fm0(pair(ϵ, a)), b).
$a\Sigma^+b$	abcd	1h	8	1000 (159)	-4965.79	1	1	$F0(x)$:=λx.append(if(not(flip(1/2)), pair(x, b), pair(x, a)), sample(if(not(flip(3/8)), if((ϵ==head(ϵ)), Fm0(Fm1(ϵ)), Σ), ϵ))).<br>$F1(x)$:=λx.ϵ.<br>$F2(x)$:=λx.pair(Fm0(pair(ϵ, a)), b).
$a\Sigma^+b$	abcd	1h	8	10000 (763)	-51404.7	1	1	$F0(x)$:=λx.append(if(not(flip(1/2)), pair(x, b), pair(x, a)), sample(if(not(flip(3/8)), if((ϵ==head(ϵ)), Fm0(Fm1(ϵ)), Σ), ϵ))).<br>$F1(x)$:=λx.ϵ.<br>$F2(x)$:=λx.pair(Fm0(pair(ϵ, a)), b).
$a\Sigma^+b$	abcd	1h	8	100000 (3222)	-507104	1	1	$F0(x)$:=λx.append(if(not(flip(1/2)), pair(x, b), pair(x, a)), sample(if(not(flip(3/8)), if((ϵ==head(ϵ)), Fm0(Fm1(ϵ)), Σ), ϵ))).<br>$F1(x)$:=λx.x.<br>$F2(x)$:=λx.pair(Fm0(pair(ϵ, a)), b).
$a\Sigma^+b$	abcd	1h	8	1 (1)	-41.9888	1	0.04	$F0(x)$:=λx.pair(F2(ϵ), a).<br>$F1(x)$:=λx.F0(ϵ).<br>$F2(x)$:=λx.ϵ.<br>$F3(x)$:=λx.pair(pair(pair(Fm1(ϵ), b), b), b).
$a\Sigma^+b$	abcd	1h	8	10 (6)	-97.1471	0.96153	1	$F0(x)$:=λx.F1(ϵ).<br>$F1(x)$:=λx.pair(if(flip(1/2), F2(ϵ), ϵ), a).<br>$F2(x)$:=λx.if(not(flip(1/4)), F0(ϵ), Fm3(ϵ)).<br>$F3(x)$:=λx.pair(Fm2(ϵ), b).
$a\Sigma^+b$	abcd	1h	8	100 (39)	-580.882	0.96	1	$F0(x)$:=λx.Fm2(pair(Fm1(x), b)).<br>$F1(x)$:=λx.x.<br>$F2(x)$:=λx.append(if(flip(1/3), x, pair(ϵ, a)), sample(if(flip(7/24), ϵ, Fm0(ϵ)))).<br>$F3(x)$:=λx.pair(F0(pair(ϵ, a)), b).
$a\Sigma^+b$	abcd	1h	8	1000 (159)	-5076.68	1	1	$F0(x)$:=λx.append(x, if(flip(1/2), insert(F1(x), F0(F2(ϵ))), sample((Σ\(pair(ϵ, d) ∪ pair(ϵ, c)))))).<br>$F1(x)$:=λx.ϵ.<br>$F2(x)$:=λx.if(flip(1/2), pair(ϵ, b), pair(ϵ, a)).<br>$F3(x)$:=λx.pair(Fm0(pair(x, a)), b).
$a\Sigma^+b$	abcd	1h	8	10000 (763)	-51414.2	1	1	$F0(x)$:=λx.append(F1(x), if(not(flip(3/8)), F0(F2(ϵ)), sample((Σ\(pair(ϵ, d) ∪ pair(ϵ, c)))))).<br>$F1(x)$:=λx.head(pair(x, a)).<br>$F2(x)$:=λx.if(flip(1/2), pair(ϵ, b), ϵ).<br>$F3(x)$:=λx.pair(Fm0(ϵ), b).
$a\Sigma^+b$	abcd	1h	8	100000 (3222)	-507107	1	1	$F0(x)$:=λx.if(not(flip(3/8)), F1(Fm2(ϵ)), pair(ϵ, a)).<br>$F1(x)$:=λx.append(append(Fm0(ϵ), sample(((Σ\pair(ϵ, c))\append(pair(ϵ, d), ϵ)))), x).<br>$F2(x)$:=λx.ϵ.<br>$F3(x)$:=λx.Fm1(pair(ϵ, b)).
a^nb^n	abcd	10m	8	1 (1)	-81.3698	0	0	$F0(x)$:=λx.pair(if(flip(1/12), ϵ, Fm0(ϵ)), a).
a^nb^n	abcd	10m	8	10 (3)	-37.5626	1	0.84	$F0(x)$:=λx.append(pair(ϵ, a), pair(if(flip(1/2), F0(ϵ), ϵ), b)).
a^nb^n	abcd	10m	8	100 (9)	-199.436	1	1	$F0(x)$:=λx.append(pair(ϵ, a), pair(if(flip(3/8), ϵ, F0(ϵ)), b)).
a^nb^n	abcd	10m	8	1000 (15)	-1988.14	1	1	$F0(x)$:=λx.append(pair(ϵ, a), pair(if(flip(1/3), ϵ, F0(ϵ)), b)).
a^nb^n	abcd	10m	8	10000 (22)	-19478.4	1	1	$F0(x)$:=λx.append(pair(ϵ, a), pair(if(flip(1/3), ϵ, F0(ϵ)), b)).
a^nb^n	abcd	10m	8	100000 (28)	-192931	1	1	$F0(x)$:=λx.append(pair(ϵ, a), pair(if(flip(1/3), x, F0(ϵ)), b)).
a^nb^n	abcd	10m	8	1 (1)	-39.1545	1	0.84	$F0(x)$:=λx.pair(if(flip(1/2), Fm1(ϵ), ϵ), b).<br>$F1(x)$:=λx.append(pair(ϵ, a), Fm0(ϵ)).
a^nb^n	abcd	10m	8	10 (3)	-44.8803	1	0.84	$F0(x)$:=λx.pair(ϵ, a).<br>$F1(x)$:=λx.append(Fm0(ϵ), pair(if(flip(1/2), Fm1(ϵ), ϵ), b)).
a^nb^n	abcd	10m	8	100 (9)	-206.753	1	1	$F0(x)$:=λx.append(pair(ϵ, a), if(flip(3/8), ϵ, Fm1(ϵ))).<br>$F1(x)$:=λx.pair(F0(ϵ), b).
a^nb^n	abcd	10m	8	1000 (15)	-1993.69	1	1	$F0(x)$:=λx.pair(if(flip(1/3), x, F1(x)), b).<br>$F1(x)$:=λx.F0(pair(x, a)).

a^nb^n	abcd	10m	8	10000 (22)	-19483.9	1	1	F0(x):=λx.pair(if(flip(1/3), x, F1(x)), b).<br>F1(x):=λx.F0(pair(x, a)).
a^nb^n	abcd	10m	8	100000 (28)	-192938	1	1	F0(x):=λx.pair(if(flip(1/3), x, F1(x)), b).<br>F1(x):=λx.F0(pair(x, a)).
a^nb^n	abcd	10m	8	1 (1)	-46.6092	0.95238	0.8	F0(x):=λx.x.<br>F1(x):=λx.if(flip(1/2), pair(F2(F0(pair(x, a))), b), x).<br>F2(x):=λx.F1(x).
a^nb^n	abcd	10m	8	10 (3)	-57.185	1	0.84	F0(x):=λx.ε.<br>F1(x):=λx.append(pair(ε, a), if(flip(1/2), pair(Fm2(ε), b), pair(ε, b))).<br>F2(x):=λx.F1(F0(ε)).
a^nb^n	abcd	10m	8	100 (9)	-214.594	1	0.88	F0(x):=λx.pair(ε, a).<br>F1(x):=λx.pair(if(flip(3/8), ε, F2(ε)), b).<br>F2(x):=λx.append(Fm0(ε), Fm1(ε)).
a^nb^n	abcd	10m	8	1000 (15)	-2003.3	1	0.88	F0(x):=λx.ε.<br>F1(x):=λx.pair(if(flip(1/3), ε, F2(ε)), b).<br>F2(x):=λx.append(pair(ε, a), F1(F0(ε))).
a^nb^n	abcd	10m	8	10000 (22)	-19493.5	1	1	F0(x):=λx.ε.<br>F1(x):=λx.pair(if(flip(1/3), F0(ε), Fm2(ε)), b).<br>F2(x):=λx.append(pair(ε, a), Fm1(ε)).
a^nb^n	abcd	10m	8	100000 (28)	-192949	1	1	F0(x):=λx.ε.<br>F1(x):=λx.pair(if(flip(1/3), Fm0(ε), Fm2(x)), b).<br>F2(x):=λx.append(pair(ε, a), F1(ε)).
a^nb^n	abcd	10m	8	1 (1)	-56.7858	1	0.84	F0(x):=λx.Fm1(ε).<br>F1(x):=λx.insert(pair(Fm2(ε), b), if(flip(1/2), F1(ε), ε)).<br>F2(x):=λx.pair(ε, a).<br>F3(x):=λx.Fm0(ε).
a^nb^n	abcd	10m	8	10 (3)	-60.9022	1	0.84	F0(x):=λx.append(Fm1(ε), pair(if(flip(1/2), F0(ε), ε), b)).<br>F1(x):=λx.pair(ε, a).<br>F2(x):=λx.ε.<br>F3(x):=λx.Fm0(Fm2(ε)).
a^nb^n	abcd	10m	8	100 (9)	-225.483	1	0.84	F0(x):=λx.append(Fm1(F2(ε)), pair(if(not(flip(3/8)), F0(ε), ε), b)).<br>F1(x):=λx.pair(ε, a).<br>F2(x):=λx.ε.<br>F3(x):=λx.Fm0(ε).
a^nb^n	abcd	10m	8	1000 (15)	-2014.19	1	0.84	F0(x):=λx.append(F1(ε), pair(if(not(flip(1/3)), F0(ε), ε), b)).<br>F1(x):=λx.pair(F2(ε), a).<br>F2(x):=λx.ε.<br>F3(x):=λx.Fm0(ε).
a^nb^n	abcd	10m	8	10000 (22)	-19505.1	1	1	F0(x):=λx.append(F1(ε), pair(if(not(flip(1/3)), F0(ε), ε), b)).<br>F1(x):=λx.pair(x, a).<br>F2(x):=λx.ε.<br>F3(x):=λx.Fm0(Fm2(ε)).
a^nb^n	abcd	10m	8	100000 (28)	-192959	1	1	F0(x):=λx.append(x, pair(if(not(flip(1/3)), F0(x), ε), b)).<br>F1(x):=λx.pair(Fm2(x), a).<br>F2(x):=λx.x.<br>F3(x):=λx.Fm0(Fm1(ε)).
Dyck	()	1d	8	1 (1)	-36.9935	0.48437	1	F0(x):=λx.if(flip(1/2), ε, append(pair(F0(ε), '('), pair(F0(ε), ')'))).
Dyck	()	1d	8	10 (6)	-69.2459	0.6	0.84	F0(x):=λx.pair(append(pair(if(flip(1/6), Fm0(pair(x, ')')), ε), '('), if(flip(1/2), Fm0(ε), ε)), ')').
Dyck	()	1d	8	100 (32)	-668.763	1	1	F0(x):=λx.pair(append(pair(x, '('), append(if(flip(if(empty(x), 5/24, 1/2)), pair(if(flip(if(empty(x), 1/6, 11/24)), F0(pair(ε, ')')), pair(ε, ')')), '('), ε), if(not(and(empty(x), flip(5/12))), ε, F0(ε)))), ')').
Dyck	()	1d	8	1000 (130)	-5460.44	1	1	F0(x):=λx.pair(append(pair(if(not(flip(1/3)), ε, F0(pair(ε, '('))), '('), if(and(empty(x), flip(11/24)), F0(ε), ε)), ')').
Dyck	()	1d	8	10000 (582)	-61107.8	1	1	F0(x):=λx.pair(append(pair(if(not(flip(1/3)), ε, F0(pair(ε, ”’))), '('), if(and(empty(x), flip(1/2)), F0(ε), ε)), ')').
Dyck	()	1d	8	100000 (2475)	-621867	1	1	F0(x):=λx.pair(append(pair(if(not(flip(1/3)), ε, F0(pair(ε, ”’))), '('), if(and(empty(x), flip(1/2)), F0(ε), ε)), ')').
Dyck	()	1d	8	1 (1)	-37.0552	1	0.04	F0(x):=λx.append(pair(x, '('), pair(x, ')')).<br>F1(x):=λx.Fm0(pair(pair(Fm0(ε), '('), ')')).
Dyck	()	1d	8	10 (6)	-72.4086	0.62963	0.52	F0(x):=λx.pair(append(pair(x, '('), if(flip(1/2), ε, Fm1(x))), ')').<br>F1(x):=λx.Fm0(if(flip(1/8), F1(ε), x)).
Dyck	()	1d	8	100 (32)	-951.49	0.88461	0.84	F0(x):=λx.append(if(not(flip(1/4)), ε, pair(if(flip(1/8), ε, x), ')')), pair(append(x, if(flip(1/24), insert(x, append(pair(ε, ')'), append(x, pair(sample((ε ∪ insert(x, pair(ε, ')')))), ')')))), sample(if(flip(11/24), F1(ε), ε)))), ')')).<br>F1(x):=λx.F0(pair(ε, '(')).
Dyck	()	1d	8	1000 (130)	-5462.26	1	1	F0(x):=λx.append(pair(if(not(flip(1/3)), ε, pair(F0(ε), ')')), '('), x).<br>F1(x):=λx.F0(pair(if(flip(11/24), F1(ε), ε), ')')).

Dyck	()	1d	8	10000 (582)	-61109.6	1	1	F0(x):=λx.append(pair(if(not(flip(1/3)), ϵ, pair(F0(ϵ), ’)’)), ’(’, x).<br>F1(x):=λx.F0(pair(if(flip(1/2), Fm1(ϵ), ϵ), ’)’)).
Dyck	()	1d	8	100000 (2475)	-621869	1	1	F0(x):=λx.append(pair(if(not(flip(1/3)), ϵ, pair(F0(ϵ), ’)’)), ’(’, x).<br>F1(x):=λx.F0(pair(if(flip(1/2), Fm1(ϵ), ϵ), ’)’)).
Dyck	()	1d	8	1 (1)	-41.2074	1	0.04	F0(x):=λx.F1(append(x, x)).<br>F1(x):=λx.pair(append(pair(x, ’(’, x), ’)’).<br>F2(x):=λx.F0(F0(ϵ)).
Dyck	()	1d	8	10 (6)	-81.3482	0.62963	0.48	F0(x):=λx.pair(x, ’)’).<br>F1(x):=λx.Fm0(append(pair(x, ’(’, if(flip(1/2), F2(x), ϵ))).<br>F2(x):=λx.F1(if(flip(1/8), Fm2(ϵ), x)).
Dyck	()	1d	8	100 (32)	-681.988	1	1	F0(x):=λx.x.<br>F1(x):=λx.pair(if(flip(7/24), pair(Fm1(F0(pair(x, ’(’))), ’)’), ϵ), ’(’).<br>F2(x):=λx.pair(append(F1(x), if(flip(1/2), Fm2(pair(head(x), ’(’)), ϵ)), ’)’).
Dyck	()	1d	8	1000 (130)	-5475.13	1	1	F0(x):=λx.append(pair(F1(ϵ), ’(’, pair(if(empty(x), ϵ, if(flip(11/24), F2(ϵ), ϵ)), ’)’)).<br>F1(x):=λx.if(flip(1/3), F0(ϵ), ϵ).<br>F2(x):=λx.F0(pair(ϵ, ’(’)).
Dyck	()	1d	8	10000 (582)	-61118.6	1	1	F0(x):=λx.ϵ.<br>F1(x):=λx.pair(append(if(flip(1/3), F1(ϵ), ϵ), append(pair(F0(ϵ), ’(’, x)), ’)’).<br>F2(x):=λx.Fm1(sample(if(flip(1/2), F2(ϵ), ϵ))).
Dyck	()	1d	8	100000 (2475)	-621878	1	1	F0(x):=λx.ϵ.<br>F1(x):=λx.pair(append(if(flip(1/3), F1(ϵ), ϵ), append(pair(F0(ϵ), ’(’, x)), ’)’).<br>F2(x):=λx.Fm1(sample(if(flip(1/2), F2(ϵ), ϵ))).
Dyck	()	1d	8	1 (1)	-49.3882	1	0.04	F0(x):=λx.Fm2(append(x, x)).<br>F1(x):=λx.F0(F0(ϵ)).<br>F2(x):=λx.pair(append(pair(x, ’(’, x), ’)’).<br>F3(x):=λx.Fm1(ϵ).
Dyck	()	1d	8	10 (6)	-92.3983	0.5	0.88	F0(x):=λx.if(flip(1/2), ϵ, Fm3(F2(x))).<br>F1(x):=λx.pair(append(x, Fm0(x)), ’)’).<br>F2(x):=λx.x.<br>F3(x):=λx.Fm1(pair(F0(x), ’(’)).
Dyck	()	1d	8	100 (32)	-755.644	1	1	F0(x):=λx.pair(if(flip(1/2), F3(ϵ), ϵ), ’)’).<br>F1(x):=λx.pair(if(flip(1/4), pair(F1(ϵ), ’)’), ϵ), ’(’).<br>F2(x):=λx.ϵ.<br>F3(x):=λx.append(F1(ϵ), F0(Fm2(ϵ))).
Dyck	()	1d	8	1000 (130)	-5479.25	1	1	F0(x):=λx.append(F1(ϵ), pair(if(flip(1/2), Fm3(ϵ), ϵ), ’)’)).<br>F1(x):=λx.pair(if(flip(1/3), pair(F1(ϵ), ’)’), Fm2(ϵ)), ’(’).<br>F2(x):=λx.ϵ.<br>F3(x):=λx.Fm0(ϵ).
Dyck	()	1d	8	10000 (582)	-61122.9	1	1	F0(x):=λx.append(F1(ϵ), pair(if(flip(1/2), Fm3(ϵ), ϵ), ’)’)).<br>F1(x):=λx.pair(if(flip(1/3), pair(F1(ϵ), ’)’), Fm2(ϵ)), ’(’).<br>F2(x):=λx.ϵ.<br>F3(x):=λx.Fm0(ϵ).
Dyck	()	1d	8	100000 (2475)	-621882	1	1	F0(x):=λx.append(F1(ϵ), pair(if(flip(1/2), Fm3(ϵ), ϵ), ’)’)).<br>F1(x):=λx.pair(if(flip(1/3), pair(F1(ϵ), ’)’), Fm2(ϵ)), ’(’).<br>F2(x):=λx.ϵ.<br>F3(x):=λx.Fm0(ϵ).
a^nb^{2n}	abcd	10m	8	1 (1)	-61.7125	0.04761	0.04	F0(x):=λx.if(flip(1/2), pair(Fm0(pair(x, a)), b), pair(x, b)).
a^nb^{2n}	abcd	10m	8	10 (4)	-69.8848	0.12	0.24	F0(x):=λx.if(not(flip(1/4)), pair(Fm0(pair(if(flip(5/24), ϵ, x), a)), b), pair(x, b)).
a^nb^{2n}	abcd	10m	8	100 (10)	-612.607	0.16	0.32	F0(x):=λx.if(not(flip(1/4)), pair(Fm0(pair(if(flip(1/12), ϵ, x), a)), b), append(x, pair(pair(pair(ϵ, a), b), b))).
a^nb^{2n}	abcd	10m	8	1000 (14)	-1999.55	1	1	F0(x):=λx.if(not(flip(1/3)), pair(pair(Fm0(pair(x, a)), b), b), append(x, pair(pair(pair(ϵ, a), b), b))).
a^nb^{2n}	abcd	10m	8	10000 (20)	-19528.7	1	1	F0(x):=λx.if(not(flip(1/3)), pair(pair(Fm0(pair(x, a)), b), b), append(x, pair(pair(pair(ϵ, a), b), b))).
a^nb^{2n}	abcd	10m	8	100000 (29)	-192423	1	1	F0(x):=λx.if(not(flip(1/3)), pair(pair(Fm0(pair(x, a)), b), b), append(x, pair(pair(pair(ϵ, a), b), b))).
a^nb^{2n}	abcd	10m	8	1 (1)	-56.1275	0.42307	0.72	F0(x):=λx.append(if(flip(1/8), x, pair(ϵ, a)), pair(x, b)).<br>F1(x):=λx.Fm0(if(flip(1/6), pair(x, b), F1(pair(x, b)))).
a^nb^{2n}	abcd	10m	8	10 (4)	-50.0371	1	0.84	F0(x):=λx.pair(ϵ, a).<br>F1(x):=λx.append(F0(ϵ), pair(pair(if(flip(1/2), Fm1(ϵ), ϵ), b), b)).
a^nb^{2n}	abcd	10m	8	100 (10)	-231.919	1	1	F0(x):=λx.pair(ϵ, a).<br>F1(x):=λx.append(Fm0(ϵ), pair(pair(if(not(flip(1/3)), F1(ϵ), ϵ), b), b)).
a^nb^{2n}	abcd	10m	8	1000 (14)	-1992.09	1	1	F0(x):=λx.pair(ϵ, a).<br>F1(x):=λx.append(Fm0(ϵ), pair(pair(if(not(flip(1/3)), F1(ϵ), ϵ), b), b)).
a^nb^{2n}	abcd	10m	8	10000 (20)	-19518.5	1	1	F0(x):=λx.pair(ϵ, a).<br>F1(x):=λx.append(Fm0(ϵ), pair(pair(if(flip(1/3), ϵ, F1(ϵ)), b), b)).

a^nb^{2n}	abcd	10m	8	100000 (29)	-192420	1	1	F0(x):=λx.pair(x, a).<br>F1(x):=λx.pair(pair(sample(if(flip(1/3), F0(x), F1(pair(x, a)))), b), b).
a^nb^{2n}	abcd	10m	8	1 (1)	-55.1242	0.25	0.76	F0(x):=λx.ϵ.<br>F1(x):=λx.append(pair(F0(ϵ), a), if(flip(1/2), append(x, x), F1(pair(x, b)))).<br>F2(x):=λx.F1(sample(Σ)).
a^nb^{2n}	abcd	10m	8	10 (4)	-58.5713	1	0.84	F0(x):=λx.pair(pair(x, b), b).<br>F1(x):=λx.ϵ.<br>F2(x):=λx.Fm0(append(pair(ϵ, a), if(flip(1/2), F2(ϵ), Fm1(ϵ)))).
a^nb^{2n}	abcd	10m	8	100 (10)	-237.745	1	0.88	F0(x):=λx.pair(Fm1(if(flip(1/3), ϵ, F2(ϵ))), b).<br>F1(x):=λx.append(pair(ϵ, a), x).<br>F2(x):=λx.pair(F0(ϵ), b).
a^nb^{2n}	abcd	10m	8	1000 (14)	-1997.92	1	0.88	F0(x):=λx.pair(Fm1(if(flip(1/3), ϵ, F2(ϵ))), b).<br>F1(x):=λx.append(pair(ϵ, a), x).<br>F2(x):=λx.pair(F0(ϵ), b).
a^nb^{2n}	abcd	10m	8	10000 (20)	-19527.1	1	0.88	F0(x):=λx.pair(Fm1(if(flip(1/3), ϵ, Fm2(ϵ))), b).<br>F1(x):=λx.append(pair(ϵ, a), x).<br>F2(x):=λx.pair(Fm0(ϵ), b).
a^nb^{2n}	abcd	10m	8	100000 (29)	-192430	1	1	F0(x):=λx.Fm1(append(x, x)).<br>F1(x):=λx.append(pair(ϵ, a), if(not(flip(1/3)), append(F1(x), x), x)).<br>F2(x):=λx.F0(pair(ϵ, b)).
a^nb^{2n}	abcd	10m	8	1 (1)	-64.2213	0.24	0.72	F0(x):=λx.append(sample(Σ), pair(pair(ϵ, b), b)).<br>F1(x):=λx.Fm0(x).<br>F2(x):=λx.if(flip(1/2), insert(Fm1(ϵ), F2(ϵ)), ϵ).<br>F3(x):=λx.Fm2(ϵ).
a^nb^{2n}	abcd	10m	8	10 (4)	-71.8035	1	0.44	F0(x):=λx.append(x, pair(Fm2(ϵ), b)).<br>F1(x):=λx.Fm0(F0(pair(ϵ, a))).<br>F2(x):=λx.if(flip(1/2), ϵ, Fm3(sample(ϵ))).<br>F3(x):=λx.F1(ϵ).
a^nb^{2n}	abcd	10m	8	100 (10)	-336.03	0.95238	0.8	F0(x):=λx.append(x, pair(ϵ, b)).<br>F1(x):=λx.Fm0(F0(pair(ϵ, a))).<br>F2(x):=λx.if(flip(1/2), insert(Fm1(ϵ), F3(ϵ)), ϵ).<br>F3(x):=λx.F2(ϵ).
a^nb^{2n}	abcd	10m	8	1000 (14)	-2309.75	1	0.52	F0(x):=λx.append(x, pair(Fm2(ϵ), b)).<br>F1(x):=λx.Fm0(F0(pair(ϵ, a))).<br>F2(x):=λx.if(flip(1/3), ϵ, Fm1(ϵ)).<br>F3(x):=λx.F1(ϵ).
a^nb^{2n}	abcd	10m	8	10000 (20)	-19550	1	1	F0(x):=λx.pair(ϵ, a).<br>F1(x):=λx.pair(ϵ, a).<br>F2(x):=λx.ϵ.<br>F3(x):=λx.pair(pair(if(not(flip(1/3)), Fm3(append(x, F0(ϵ))), append(Fm1(head(F2(ϵ))), x)), b), b).
a^nb^{2n}	abcd	10m	8	100000 (29)	-193039	1	1	F0(x):=λx.pair(ϵ, a).<br>F1(x):=λx.ϵ.<br>F2(x):=λx.pair(x, a).<br>F3(x):=λx.pair(pair(if(not(flip(1/3)), Fm3(F2(x)), append(Fm0(F1(ϵ)), x)), b), b).
a^ncb^n	abcd	10m	8	1 (1)	-27.5548	0	0	F0(x):=λx.pair(pair(pair(ϵ, a), a), c).
a^ncb^n	abcd	10m	8	10 (4)	-47.2645	1	0.84	F0(x):=λx.append(pair(ϵ, a), append(if(flip(1/2), pair(ϵ, c), Fm0(ϵ)), pair(ϵ, b))).
a^ncb^n	abcd	10m	8	100 (11)	-231.421	1	1	F0(x):=λx.append(pair(ϵ, a), append(if(flip(1/3), pair(ϵ, c), Fm0(ϵ)), pair(ϵ, b))).
a^ncb^n	abcd	10m	8	1000 (14)	-1974.97	1	1	F0(x):=λx.append(pair(ϵ, a), append(if(flip(1/3), pair(ϵ, c), Fm0(ϵ)), pair(ϵ, b))).
a^ncb^n	abcd	10m	8	10000 (23)	-19343.3	1	1	F0(x):=λx.pair(if(flip(1/3), pair(pair(x, a), c), Fm0(pair(x, a))), b).
a^ncb^n	abcd	10m	8	100000 (27)	-193306	1	1	F0(x):=λx.pair(if(flip(1/3), pair(pair(x, a), c), Fm0(pair(x, a))), b).
a^ncb^n	abcd	10m	8	1 (1)	-31.1238	1	0.04	F0(x):=λx.pair(pair(pair(pair(ϵ, a), a), c), b).<br>F1(x):=λx.pair(Fm0(ϵ), b).
a^ncb^n	abcd	10m	8	10 (4)	-50.7302	1	0.84	F0(x):=λx.append(pair(ϵ, a), if(flip(1/2), Fm1(ϵ), pair(ϵ, c))).<br>F1(x):=λx.pair(Fm0(ϵ), b).
a^ncb^n	abcd	10m	8	100 (11)	-237.595	1	1	F0(x):=λx.append(pair(ϵ, a), if(not(flip(1/3)), Fm1(ϵ), pair(ϵ, c))).<br>F1(x):=λx.pair(F0(ϵ), b).
a^ncb^n	abcd	10m	8	1000 (14)	-1981.14	1	1	F0(x):=λx.append(pair(ϵ, a), if(not(flip(1/3)), F1(ϵ), pair(ϵ, c))).<br>F1(x):=λx.pair(F0(ϵ), b).
a^ncb^n	abcd	10m	8	10000 (23)	-19351.4	1	1	F0(x):=λx.append(pair(ϵ, a), if(not(flip(1/3)), F1(ϵ), pair(ϵ, c))).<br>F1(x):=λx.pair(F0(ϵ), b).
a^ncb^n	abcd	10m	8	100000 (27)	-193314	1	1	F0(x):=λx.append(pair(x, a), if(not(flip(1/3)), Fm1(ϵ), pair(ϵ, c))).<br>F1(x):=λx.pair(Fm0(ϵ), b).

$a^n cb^n$	abcd	10m	8	1 (1)	-40.0872	0	0	F0(x):=λx.pair(pair(F1(ε), c), b).<br>F1(x):=λx.pair(pair(ε, a), a).<br>F2(x):=λx.Fm0(ε).
$a^n cb^n$	abcd	10m	8	10 (4)	-59.2644	1	0.84	F0(x):=λx.pair(x, b).<br>F1(x):=λx.ε.<br>F2(x):=λx.Fm0(append(pair(Fm1(ε), a), if(flip(1/2), pair(ε, c), Fm2(ε)))).
$a^n cb^n$	abcd	10m	8	100 (11)	-243.421	1	0.88	F0(x):=λx.pair(x, b).<br>F1(x):=λx.ε.<br>F2(x):=λx.Fm0(append(pair(ε, a), if(flip(1/3), pair(ε, c), Fm2(F1(ε))))).
$a^n cb^n$	abcd	10m	8	1000 (14)	-1986.97	1	0.88	F0(x):=λx.pair(x, b).<br>F1(x):=λx.ε.<br>F2(x):=λx.Fm0(append(pair(ε, a), if(flip(1/3), pair(ε, c), Fm2(F1(ε))))).
$a^n cb^n$	abcd	10m	8	10000 (23)	-19357.2	1	1	F0(x):=λx.pair(x, b).<br>F1(x):=λx.ε.<br>F2(x):=λx.Fm0(append(pair(ε, a), if(flip(1/3), pair(Fm1(ε), c), Fm2(ε)))).
$a^n cb^n$	abcd	10m	8	100000 (27)	-193320	1	1	F0(x):=λx.pair(x, b).<br>F1(x):=λx.x.<br>F2(x):=λx.Fm0(append(pair(ε, a), if(flip(1/3), pair(Fm1(ε), c), Fm2(ε)))).
$a^n cb^n$	abcd	10m	8	1 (1)	-47.8388	1	0.04	F0(x):=λx.F2(pair(x, b)).<br>F1(x):=λx.pair(pair(pair(ε, a), a), c).<br>F2(x):=λx.pair(x, b).<br>F3(x):=λx.F0(Fm1(ε)).
$a^n cb^n$	abcd	10m	8	10 (4)	-67.4452	1	0.84	F0(x):=λx.pair(ε, c).<br>F1(x):=λx.F2(append(pair(ε, a), if(flip(1/2), F0(ε), Fm3(ε)))).<br>F2(x):=λx.pair(x, b).<br>F3(x):=λx.Fm1(ε).
$a^n cb^n$	abcd	10m	8	100 (11)	-251.602	1	0.84	F0(x):=λx.pair(ε, c).<br>F1(x):=λx.F2(append(pair(ε, a), if(flip(1/3), F0(ε), Fm3(ε)))).<br>F2(x):=λx.pair(x, b).<br>F3(x):=λx.Fm1(ε).
$a^n cb^n$	abcd	10m	8	1000 (14)	-1995.15	1	0.84	F0(x):=λx.pair(ε, c).<br>F1(x):=λx.F2(append(pair(ε, a), if(flip(1/3), F0(ε), Fm3(ε)))).<br>F2(x):=λx.pair(x, b).<br>F3(x):=λx.Fm1(ε).
$a^n cb^n$	abcd	10m	8	10000 (23)	-19365.4	1	1	F0(x):=λx.pair(ε, c).<br>F1(x):=λx.F2(append(pair(ε, a), if(flip(1/3), F0(ε), Fm1(ε)))).<br>F2(x):=λx.pair(x, b).<br>F3(x):=λx.Fm1(ε).
$a^n cb^n$	abcd	10m	8	100000 (27)	-193328	1	1	F0(x):=λx.pair(ε, c).<br>F1(x):=λx.F2(append(pair(x, a), if(flip(1/3), F0(ε), Fm1(ε)))).<br>F2(x):=λx.pair(x, b).<br>F3(x):=λx.Fm1(ε).
$a^n(ab)^n$	abcd	10m	8	1 (1)	-32.0185	0	0	F0(x):=λx.pair(pair(pair(pair(ε, a), a), a), b).
$a^n(ab)^n$	abcd	10m	8	10 (4)	-53.8076	1	0.84	F0(x):=λx.append(pair(if(flip(1/2), append(pair(ε, a), F0(ε)), pair(ε, a)), a), pair(ε, b)).
$a^n(ab)^n$	abcd	10m	8	100 (12)	-228.051	1	1	F0(x):=λx.append(pair(if(not(flip(1/3)), append(pair(ε, a), F0(ε)), pair(ε, a)), a), pair(ε, b)).
$a^n(ab)^n$	abcd	10m	8	1000 (14)	-1960.36	1	1	F0(x):=λx.insert(append(pair(pair(ε, a), a), pair(ε, b)), if(flip(1/3), ε, Fm0(ε))).
$a^n(ab)^n$	abcd	10m	8	10000 (24)	-19333.4	1	1	F0(x):=λx.insert(append(pair(pair(ε, a), a), pair(ε, b)), if(flip(1/3), ε, Fm0(ε))).
$a^n(ab)^n$	abcd	10m	8	100000 (29)	-193289	1	1	F0(x):=λx.insert(append(pair(pair(ε, a), a), pair(ε, b)), if(flip(1/3), ε, Fm0(ε))).
$a^n(ab)^n$	abcd	10m	8	1 (1)	-40.6771	0	0	F0(x):=λx.pair(pair(sample(Σ), a), a).<br>F1(x):=λx.Fm0(ε).
$a^n(ab)^n$	abcd	10m	8	10 (4)	-59.5042	0.96	1	F0(x):=λx.append(append(x, if(flip(7/24), ε, pair(Fm0(pair(ε, a)), b))), x).<br>F1(x):=λx.Fm0(ε).
$a^n(ab)^n$	abcd	10m	8	100 (12)	-245.976	1	0.84	F0(x):=λx.append(append(x, if(not(or((x==ε), flip(1/2))), ε, pair(Fm0(pair(ε, a)), b))), x).<br>F1(x):=λx.F0(ε).
$a^n(ab)^n$	abcd	10m	8	1000 (14)	-2015.39	0.96	1	F0(x):=λx.append(append(x, if(flip(if(empty(x), 1/24, 1/3)), ε, pair(Fm0(pair(ε, a)), b))), x).<br>F1(x):=λx.Fm0(ε).
$a^n(ab)^n$	abcd	10m	8	10000 (24)	-19771.5	0.96	1	F0(x):=λx.append(append(x, if(flip(if(empty(x), 1/24, 1/3)), ε, pair(Fm0(pair(ε, a)), b))), x).<br>F1(x):=λx.Fm0(ε).
$a^n(ab)^n$	abcd	10m	8	100000 (29)	-197558	0.96	1	F0(x):=λx.append(append(x, if(flip(if(empty(x), 1/24, 1/3)), ε, pair(Fm1(pair(ε, a)), b))), x).<br>F1(x):=λx.F0(x).

$a^n(ab)^n$	abcd	10m	8	1 (1)	-43.5915	1	0.04	F0(x):=λx.append(pair(ϵ, a), append(pair(x, a), Fm1(ϵ))).<br>F1(x):=λx.pair(ϵ, b).<br>F2(x):=λx.Fm0(F0(ϵ)).
$a^n(ab)^n$	abcd	10m	8	10 (4)	-67.4985	1	0.84	F0(x):=λx.append(x, insert(x, if(flip(1/2), pair(F1(ϵ), b), ϵ))).<br>F1(x):=λx.Fm0(pair(ϵ, a)).<br>F2(x):=λx.pair(Fm1(ϵ), b).
$a^n(ab)^n$	abcd	10m	8	100 (12)	-241.113	1	0.88	F0(x):=λx.if(flip(1/3), pair(pair(ϵ, a), a), append(pair(ϵ, a), pair(F1(ϵ), a))).<br>F1(x):=λx.Fm2(ϵ).<br>F2(x):=λx.pair(F0(ϵ), b).
$a^n(ab)^n$	abcd	10m	8	1000 (14)	-1972.77	1	0.88	F0(x):=λx.pair(ϵ, a).<br>F1(x):=λx.pair(if(not(flip(1/3)), F2(ϵ), ϵ), a).<br>F2(x):=λx.pair(append(F0(ϵ), F1(ϵ)), b).
$a^n(ab)^n$	abcd	10m	8	10000 (24)	-19355.1	1	1	F0(x):=λx.append(x, insert(x, if(or(not(flip(1/3)), empty(x)), pair(F0(pair(F1(ϵ), a)), b), ϵ))).<br>F1(x):=λx.ϵ.<br>F2(x):=λx.F0(ϵ).
$a^n(ab)^n$	abcd	10m	8	100000 (29)	-193311	1	1	F0(x):=λx.append(x, insert(x, if(or(not(flip(1/3)), empty(x)), pair(F0(pair(ϵ, a)), b), ϵ))).<br>F1(x):=λx.ϵ.<br>F2(x):=λx.Fm0(Fm1(ϵ)).
$a^n(ab)^n$	abcd	10m	8	1 (1)	-54.8281	0	0	F0(x):=λx.F1(ϵ).<br>F1(x):=λx.F2(pair(ϵ, a)).<br>F2(x):=λx.append(append(x, x), pair(x, b)).<br>F3(x):=λx.Fm0(ϵ).
$a^n(ab)^n$	abcd	10m	8	10 (4)	-69.6063	1	0.84	F0(x):=λx.pair(F2(pair(ϵ, a)), b).<br>F1(x):=λx.ϵ.<br>F2(x):=λx.append(append(x, if(flip(1/2), Fm1(ϵ), Fm3(ϵ))), x).<br>F3(x):=λx.Fm0(ϵ).
$a^n(ab)^n$	abcd	10m	8	100 (12)	-241.141	1	0.64	F0(x):=λx.pair(F2(pair(ϵ, a)), b).<br>F1(x):=λx.ϵ.<br>F2(x):=λx.append(append(x, if(flip(1/3), ϵ, Fm3(ϵ))), x).<br>F3(x):=λx.Fm0(Fm1(ϵ)).
$a^n(ab)^n$	abcd	10m	8	1000 (14)	-1978.93	1	1	F0(x):=λx.ϵ.<br>F1(x):=λx.pair(pair(x, a), b).<br>F2(x):=λx.ϵ.<br>F3(x):=λx.Fm1(append(pair(ϵ, a), if(flip(1/3), Fm0(F2(ϵ)), F3(ϵ)))).
$a^n(ab)^n$	abcd	10m	8	10000 (24)	-19352	1	1	F0(x):=λx.ϵ.<br>F1(x):=λx.pair(pair(x, a), b).<br>F2(x):=λx.ϵ.<br>F3(x):=λx.Fm1(append(pair(ϵ, a), if(flip(1/3), Fm0(F2(ϵ)), F3(ϵ)))).
$a^n(ab)^n$	abcd	10m	8	100000 (29)	-193308	1	1	F0(x):=λx.pair(F2(pair(ϵ, a)), b).<br>F1(x):=λx.F0(ϵ).<br>F2(x):=λx.append(append(x, if(flip(1/3), ϵ, Fm0(ϵ))), x).<br>F3(x):=λx.Fm1(ϵ).
$(ab)^n(aba)^n$	abcd	5d	8	1 (1)	-46.7554	0	0	F0(x):=λx.pair(append(x, pair(if(flip(1/2), pair(F0(Fm0(ϵ)), a), x), a)), b).
$(ab)^n(aba)^n$	abcd	5d	8	10 (6)	-63.9489	1	1	F0(x):=λx.pair(pair(pair(append(pair(pair(ϵ, a), b), if(flip(5/24), ϵ, F0(ϵ))), a), b), a).
$(ab)^n(aba)^n$	abcd	5d	8	100 (12)	-242.907	1	1	F0(x):=λx.pair(append(pair(pair(ϵ, a), b), pair(pair(if(flip(3/8), ϵ, Fm0(ϵ)), a), b)), a).
$(ab)^n(aba)^n$	abcd	5d	8	1000 (17)	-2014.51	1	1	F0(x):=λx.append(pair(pair(ϵ, a), b), pair(pair(pair(if(flip(1/3), ϵ, Fm0(ϵ)), a), b), a)).
$(ab)^n(aba)^n$	abcd	5d	8	10000 (20)	-19423.2	1	1	F0(x):=λx.append(pair(pair(ϵ, a), b), pair(pair(pair(if(flip(1/3), ϵ, Fm0(ϵ)), a), b), a)).
$(ab)^n(aba)^n$	abcd	5d	8	100000 (25)	-194225	1	1	F0(x):=λx.append(pair(pair(ϵ, a), b), pair(pair(pair(if(flip(1/3), ϵ, Fm0(ϵ)), a), b), a)).
$(ab)^n(aba)^n$	abcd	5d	8	1 (1)	-46.415	1	1	F0(x):=λx.append(x, pair(append(if(flip(1/8), ϵ, F1(ϵ)), x), a)).<br>F1(x):=λx.F0(pair(pair(ϵ, a), b)).
$(ab)^n(aba)^n$	abcd	5d	8	10 (6)	-67.5778	1	1	F0(x):=λx.append(append(x, if(flip(5/24), ϵ, F1(ϵ))), pair(x, a)).<br>F1(x):=λx.F0(pair(pair(ϵ, a), b)).
$(ab)^n(aba)^n$	abcd	5d	8	100 (12)	-244.18	1	1	F0(x):=λx.pair(append(append(x, if(flip(1/3), ϵ, Fm1(ϵ))), x), a).<br>F1(x):=λx.Fm0(pair(pair(ϵ, a), b)).
$(ab)^n(aba)^n$	abcd	5d	8	1000 (17)	-2018.14	1	1	F0(x):=λx.append(x, append(if(flip(1/3), ϵ, Fm1(ϵ)), x)).<br>F1(x):=λx.pair(F0(pair(pair(ϵ, a), b)), a).
$(ab)^n(aba)^n$	abcd	5d	8	10000 (20)	-19426.9	1	1	F0(x):=λx.append(x, append(if(flip(1/3), ϵ, Fm1(ϵ)), x)).<br>F1(x):=λx.pair(F0(pair(pair(ϵ, a), b)), a).
$(ab)^n(aba)^n$	abcd	5d	8	100000 (25)	-194231	1	1	F0(x):=λx.pair(pair(if(flip(1/3), x, F1(x)), a), b).<br>F1(x):=λx.pair(F0(pair(pair(x, a), b)), a).

$(ab)^n(aba)^n$	abcd	5d	8	1 (1)	-54.256	1	0.88	F0(x):=λx.append(append(x, F1(ϵ)), pair(x, a)).<br>F1(x):=λx.if(flip(1/8), ϵ, F2(ϵ)).<br>F2(x):=λx.F0(pair(pair(ϵ, a), b)).
$(ab)^n(aba)^n$	abcd	5d	8	10 (6)	-74.6632	1	0.64	F0(x):=λx.pair(pair(x, a), b).<br>F1(x):=λx.F0(if(flip(5/24), x, Fm2(x))).<br>F2(x):=λx.pair(Fm1(Fm0(x)), a).
$(ab)^n(aba)^n$	abcd	5d	8	100 (12)	-251.265	1	0.64	F0(x):=λx.pair(pair(x, a), b).<br>F1(x):=λx.F0(if(flip(1/3), x, Fm2(x))).<br>F2(x):=λx.pair(Fm1(Fm0(x)), a).
$(ab)^n(aba)^n$	abcd	5d	8	1000 (17)	-2025.98	1	0.88	F0(x):=λx.pair(Fm1(pair(pair(ϵ, a), b)), a).<br>F1(x):=λx.append(x, append(if(flip(1/3), ϵ, Fm2(ϵ)), x)).<br>F2(x):=λx.F0(ϵ).
$(ab)^n(aba)^n$	abcd	5d	8	10000 (20)	-19434.7	1	1	F0(x):=λx.pair(Fm1(pair(pair(ϵ, a), b)), a).<br>F1(x):=λx.append(x, append(if(flip(1/3), ϵ, Fm0(ϵ)), x)).<br>F2(x):=λx.Fm0(ϵ).
$(ab)^n(aba)^n$	abcd	5d	8	100000 (25)	-194192	0.76	0.88	F0(x):=λx.if(flip(1/3), if(and(flip(1/12), flip(11/24)), if(flip(1/8), append(x, append(x, insert(append(x, pair(x, a)), append(append(x, pair(x, a)), pair(x, a))))), ϵ), ϵ), F2(ϵ)).<br>F1(x):=λx.append(append(x, F0(x)), x).<br>F2(x):=λx.pair(F1(pair(pair(ϵ, a), b)), a).
$(ab)^n(aba)^n$	abcd	5d	8	1 (1)	-62.4369	1	0.88	F0(x):=λx.append(append(x, if(flip(1/8), Fm2(ϵ), F1(ϵ))), pair(x, a)).<br>F1(x):=λx.F3(ϵ).<br>F2(x):=λx.ϵ.<br>F3(x):=λx.Fm0(pair(pair(ϵ, a), b)).
$(ab)^n(aba)^n$	abcd	5d	8	10 (6)	-83.5996	1	0.64	F0(x):=λx.pair(append(x, append(if(flip(5/24), ϵ, Fm3(Fm1(ϵ))), x)), a).<br>F1(x):=λx.ϵ.<br>F2(x):=λx.pair(pair(ϵ, a), b).<br>F3(x):=λx.Fm0(F2(ϵ)).
$(ab)^n(aba)^n$	abcd	5d	8	100 (12)	-260.202	1	0.84	F0(x):=λx.append(append(x, if(flip(1/3), F2(ϵ), Fm3(ϵ))), pair(x, a)).<br>F1(x):=λx.pair(pair(ϵ, a), b).<br>F2(x):=λx.ϵ.<br>F3(x):=λx.F0(Fm1(ϵ)).
$(ab)^n(aba)^n$	abcd	5d	8	1000 (17)	-2034.16	1	0.84	F0(x):=λx.append(append(x, if(flip(1/3), F2(ϵ), Fm3(ϵ))), pair(x, a)).<br>F1(x):=λx.pair(pair(ϵ, a), b).<br>F2(x):=λx.ϵ.<br>F3(x):=λx.F0(Fm1(ϵ)).
$(ab)^n(aba)^n$	abcd	5d	8	10000 (20)	-19442.9	1	1	F0(x):=λx.append(x, append(if(flip(1/3), Fm1(Fm2(ϵ)), F3(ϵ)), pair(x, a))).<br>F1(x):=λx.ϵ.<br>F2(x):=λx.ϵ.<br>F3(x):=λx.Fm0(pair(pair(ϵ, a), b)).
$(ab)^n(aba)^n$	abcd	5d	8	100000 (25)	-194245	1	1	F0(x):=λx.F2(ϵ).<br>F1(x):=λx.pair(append(x, append(if(flip(1/3), ϵ, F2(ϵ)), x)), a).<br>F2(x):=λx.F1(pair(pair(ϵ, a), b)).<br>F3(x):=λx.Fm0(ϵ).
$a^nb^mc^n$	abcd	2h	8	1 (1)	-33.4263	1	0.04	F0(x):=λx.pair(pair(pair(pair(pair(pair(pair(ϵ, a), a), b), b), b), c), c).
$a^nb^mc^n$	abcd	2h	8	10 (8)	-113.864	1	0.72	F0(x):=λx.append(pair(ϵ, a), if(flip(1/2), pair(F0(if(flip(1/2), pair(x, b), ϵ)), c), insert(pair(sample((pair(pair(ϵ, b), b)∪append(pair(append(x, x), b), append(x, x)))), c), x))).
$a^nb^mc^n$	abcd	2h	8	100 (40)	-631.69	0.69230	0.72	F0(x):=λx.if(flip(3/8), append(pair(if(flip(1/8), Fm0(pair(append(x, x), b)), x), b), sample(if(not(flip(7/24)), x, pair(append(x, append(x, x)), b)))), append(pair(ϵ, a), pair(F0(x), c))).
$a^nb^mc^n$	abcd	2h	8	1000 (102)	-8027.29	0.88	0.96	F0(x):=λx.if(flip(1/3), append(pair(if(flip(1/24), Fm0(pair(x, b)), x), b), sample(if(not(flip(1/2)), x, pair(append(x, append(append(x, head(x)), if(flip(5/24), pair(tail(x), b), head(x)))), b)))), append(pair(append(ϵ, ϵ), a), pair(F0(x), c))).
$a^nb^mc^n$	abcd	2h	8	10000 (200)	-81742.3	0.88	0.96	F0(x):=λx.if(flip(1/3), append(pair(if(flip(1/24), Fm0(pair(x, b)), x), b), sample(if(not(flip(1/2)), x, pair(append(x, append(append(x, head(x)), if(flip(5/24), pair(tail(x), b), head(x)))), b)))), append(pair(append(ϵ, ϵ), a), pair(F0(x), c))).
$a^nb^mc^n$	abcd	2h	8	100000 (324)	-807702	0.88	0.96	F0(x):=λx.if(flip(1/3), append(pair(if(flip(1/24), Fm0(pair(x, b)), x), b), sample(if(not(flip(1/2)), x, pair(append(x, append(append(x, head(x)), if(flip(5/24), pair(tail(x), b), head(x)))), b)))), append(pair(append(ϵ, ϵ), a), pair(F0(x), c))).
$a^nb^mc^n$	abcd	2h	8	1 (1)	-40.7639	0.04761	0.04	F0(x):=λx.pair(if(flip(1/2), F0(pair(x, a)), x), b).<br>F1(x):=λx.pair(pair(F0(ϵ), c), c).
$a^nb^mc^n$	abcd	2h	8	10 (8)	-112.955	1	0.84	F0(x):=λx.pair(sample(if(flip(1/2), Fm1(x), insert(x, sample(((ϵ∪pair(x, b))∪x))))), c).<br>F1(x):=λx.append(pair(ϵ, a), Fm0(pair(if(flip(1/2), ϵ, pair(x, b)), b))).
$a^nb^mc^n$	abcd	2h	8	100 (40)	-690.361	1	0.96	F0(x):=λx.pair(sample(if((ϵ==if(flip(1/3), x, ϵ)), Fm1(head(x)), insert(x, sample((ϵ∪append(append(if(flip(1/2), append(x, x), ϵ), ϵ), append(x, if(flip(1/6), ϵ, x)))))))), c).<br>F1(x):=λx.append(pair(ϵ, a), Fm0(pair(if(flip(1/24), pair(ϵ, b), ϵ), b))).

$a^nb^mc^n$	abcd	2h	8	1000 (102)	-7507.42	1	0.96	$F0(x)$:=λx.pair(sample(if((ε==if(flip(7/24), x, ε)), Fm1(ε), insert(append(ε, x), sample((ε∪append(append(if(flip(1/2), append(x, x), append(ε, ε)), ε), append(x, if(flip(1/8), ε, x)))))))), c).<br>$F1(x)$:=λx.append(pair(ε, a), Fm0(pair(if(flip(1/24), pair(ε, b), ε), b))).
$a^nb^mc^n$	abcd	2h	8	10000 (200)	-43956.6	1	1	$F0(x)$:=λx.sample(if(flip(1/3), append(pair(ε, b), pair(if(flip(5/24), pair(ε, b), Fm0(ε)), b)), sample(if(not(flip(if((pair(ε, b)==x), 1/8, 1/2))), Fm1(sample((ε\if((ε==ε), x, Σ)))), x)))).<br>$F1(x)$:=λx.pair(append(pair(ε, a), F0(pair(ε, b))), c).
$a^nb^mc^n$	abcd	2h	8	100000 (324)	-441486	1	1	$F0(x)$:=λx.sample(if(flip(7/24), append(pair(ε, b), pair(if(flip(3/8), pair(if(flip(1/3), pair(ε, b), ε), b), Fm0(ε)), b)), if(not(flip(if((pair(ε, b)==x), 1/8, 1/2))), Fm1(sample((x\ε))), x))).<br>$F1(x)$:=λx.pair(append(pair(ε, a), F0(pair(ε, b))), c).
$a^nb^mc^n$	abcd	2h	8	1 (1)	-45.4071	1	0.04	$F0(x)$:=λx.pair(insert(x, x), b).<br>$F1(x)$:=λx.pair(Fm0(pair(pair(ε, a), b)), c).<br>$F2(x)$:=λx.pair(Fm1(ε), c).
$a^nb^mc^n$	abcd	2h	8	10 (8)	-125.761	0.82142	1	$F0(x)$:=λx.pair(append(x, Fm1(ε)), c).<br>$F1(x)$:=λx.if(flip(1/2), Fm2(x), pair(if(flip(1/2), pair(pair(if(flip(1/2), F1(ε), ε), b), b), sample(if(flip(1/3), ε, pair(ε, b)))), b)).<br>$F2(x)$:=λx.Fm0(pair(ε, a)).
$a^nb^mc^n$	abcd	2h	8	100 (40)	-844.722	0.72	1	$F0(x)$:=λx.pair(append(pair(ε, a), x), c).<br>$F1(x)$:=λx.if(flip(5/12), F2(ε), pair(if(flip(7/24), pair(if(not(flip(3/8)), Fm1(x), if(flip(1/2), ε, ε)), b), ε), b)).<br>$F2(x)$:=λx.F0(F1(ε)).
$a^nb^mc^n$	abcd	2h	8	1000 (102)	-9274.6	0.34615	0.8	$F0(x)$:=λx.append(if(flip(7/24), pair(append(if(flip(1/2), x, if(flip(5/12), pair(ε, a), pair(pair(x, b), b))), F1(pair(ε, c))), b), Fm2(x)), Fm1(head(x))).<br>$F1(x)$:=λx.sample(((if(flip(7/24), (ε∪insert(pair(ε, c), ε)), Σ)\x)\pair(ε, d))).<br>$F2(x)$:=λx.Fm0(append(x, pair(ε, a))).
$a^nb^mc^n$	abcd	2h	8	10000 (200)	-92126.2	0.32	0.8	$F0(x)$:=λx.append(if(flip(7/24), pair(append(if(flip(1/2), x, if(flip(7/24), pair(head(ε), a), pair(pair(x, b), b))), F1(pair(ε, c))), b), Fm2(x)), Fm1(head(x))).<br>$F1(x)$:=λx.sample(((if(flip(7/24), (ε∪insert(pair(ε, c), ε)), Σ)\x)\pair(ε, d))).<br>$F2(x)$:=λx.Fm0(pair(x, a)).
$a^nb^mc^n$	abcd	2h	8	100000 (324)	-916965	0.32	0.8	$F0(x)$:=λx.append(if(flip(7/24), pair(append(if(flip(1/2), x, if(flip(7/24), pair(head(ε), a), pair(pair(x, b), b))), F1(pair(ε, c))), b), Fm2(x)), Fm1(head(x))).<br>$F1(x)$:=λx.sample(((if(flip(7/24), (ε∪insert(pair(ε, c), ε)), Σ)\x)\pair(ε, d))).<br>$F2(x)$:=λx.Fm0(pair(x, a)).
$a^nb^mc^n$	abcd	2h	8	1 (1)	-54.8281	0	0	$F0(x)$:=λx.ε.<br>$F1(x)$:=λx.insert(pair(x, c), pair(x, b)).<br>$F2(x)$:=λx.pair(pair(Fm0(ε), a), b).<br>$F3(x)$:=λx.F1(Fm2(ε)).
$a^nb^mc^n$	abcd	2h	8	10 (8)	-116.204	0.75	1	$F0(x)$:=λx.if(flip(1/2), F2(ε), pair(Fm0(ε), b)).<br>$F1(x)$:=λx.ε.<br>$F2(x)$:=λx.ε.<br>$F3(x)$:=λx.if(flip(1/2), append(pair(ε, a), append(Fm3(ε), pair(ε, c))), pair(Fm0(Fm1(ε)), b)).
$a^nb^mc^n$	abcd	2h	8	100 (40)	-485.668	0.76	1	$F0(x)$:=λx.if(flip(1/3), ε, pair(Fm2(ε), b)).<br>$F1(x)$:=λx.ε.<br>$F2(x)$:=λx.Fm0(ε).<br>$F3(x)$:=λx.if(not(flip(1/4)), append(pair(ε, a), append(F3(F1(ε)), pair(ε, c))), pair(F2(ε), b)).
$a^nb^mc^n$	abcd	2h	8	1000 (102)	-4018.54	1	1	$F0(x)$:=λx.pair(insert(if(not(flip(3/8)), ε, pair(head(x), b)), if(flip(5/24), ε, append(append(if(flip(5/12), pair(pair(if(not(flip(1/4)), ε, x), b), b), x), sample(x)), x))), b).<br>$F1(x)$:=λx.if(flip(1/8), pair(x, b), ε).<br>$F2(x)$:=λx.pair(ε, c).<br>$F3(x)$:=λx.append(append(pair(x, a), sample(if(or(empty(pair(ε, c)), flip(1/12)), pair(F0(pair(Fm1(pair(ε, b)), b)), b), if(not(flip(7/24)), Fm3(ε), F0(x))))), Fm2(ε)).
$a^nb^mc^n$	abcd	2h	8	10000 (200)	-40048	1	1	$F0(x)$:=λx.pair(insert(if(not(flip(1/3)), ε, pair(x, b)), if(flip(7/24), append(x, x), append(append(if(flip(5/12), pair(pair(if(not(flip(3/8)), ε, x), b), b), x), x), x))), b).<br>$F1(x)$:=λx.if(flip(1/6), pair(pair(append(ε, ε), b), b), x).<br>$F2(x)$:=λx.pair(ε, c).<br>$F3(x)$:=λx.append(append(pair(x, a), sample(if(flip(1/12), pair(F0(pair(Fm1(ε), b)), b), if(not(flip(7/24)), Fm3(ε), F0(ε))))), Fm2(ε)).
$a^nb^mc^n$	abcd	2h	8	100000 (324)	-404483	1	1	$F0(x)$:=λx.pair(insert(if(not(flip(1/3)), ε, pair(x, b)), if(flip(3/8), append(x, x), append(append(if(flip(11/24), pair(pair(if(not(flip(1/3)), ε, x), b), b), x), x), x))), b).<br>$F1(x)$:=λx.if(flip(1/8), pair(pair(append(ε, ε), b), b), ε).<br>$F2(x)$:=λx.pair(ε, c).<br>$F3(x)$:=λx.append(append(pair(ε, a), sample(if(flip(1/12), pair(F0(pair(Fm1(append(ε, pair(x, d))), b)), b), if(not(flip(1/4)), Fm3(ε), F0(x))))), Fm2(ε)).
$a^nb^ma^{2n}$	abcd	1d	8	1 (1)	-34.914	0.06666	0.04	$F0(x)$:=λx.append(if(flip(1/2), F0(pair(x, a)), pair(pair(x, a), b)), x).
$a^nb^ma^{2n}$	abcd	1d	8	10 (10)	-110.832	0.74074	0.84	$F0(x)$:=λx.append(x, if(flip(1/4), pair(if(flip(1/4), ε, pair(if(flip(1/2), Fm0(ε), ε), b)), b), pair(pair(Fm0(pair(ε, a)), a), a))).
$a^nb^ma^{2n}$	abcd	1d	8	100 (35)	-665.879	1	0.88	$F0(x)$:=λx.pair(if(flip(3/8), pair(pair(append(pair(ε, a), if(flip(1/2), if(flip(5/12), x, ε), append(pair(sample((pair(ε, b)∪(pair(x, b)∪ε))), b), if(flip(7/24), append(pair(x, b), pair(x, b)), ε)))), b), a), pair(append(pair(ε, a), Fm0(pair(if(flip(1/24), ε, x), b))), a)), a).

$a^nb^ma^{2n}$	abcd	1d	8	1000 (100)	-7024.33	0.88	0.96	$F0(x)$:=λx.pair(append(pair(ε, a), pair(sample(if(not(flip(1/3)), F0(pair(ε, b)), append(if(not(flip(1/3)), ε, pair(pair(sample((pair(append(pair(sample(Σ), b), append(x, x)), b)∪x)), b), b)), pair(sample(if(flip(7/24), x, if(flip(5/12), pair(x, b), ε))), b)))), a)), a).
$a^nb^ma^{2n}$	abcd	1d	8	10000 (185)	-75712.3	0.5	0.6	$F0(x)$:=λx.pair(append(pair(ε, a), if(flip(1/3), append(pair(if(flip(1/2), ε, pair(pair(if(not(flip(11/24)), ε, append(if(flip(7/24), pair(pair(if(flip(1/2), ε, pair(sample(Σ), b)), b), b), ε), pair(pair(ε, b), b))), b), b)), b), sample(((Σ\pair(ε, c))\pair(ε, d)))), pair(F0(ε), a))), a).
$a^nb^ma^{2n}$	abcd	1d	8	100000 (330)	-698746	0.96	1	$F0(x)$:=λx.if(or(empty(x), not(flip(1/3))), F0(pair(x, a)), append(if(flip(1/24), x, insert(if(not(flip(1/6)), append(x, x), append(pair(pair(pair(pair(x, b), b), b), b), x)), pair(if(flip(3/8), ε, append(if(not(flip(7/24)), ε, pair(pair(ε, b), b)), pair(if(flip(3/8), pair(ε, b), ε), b))), b))), x)).
$a^nb^ma^{2n}$	abcd	1d	8	1 (1)	-38.4613	0.06666	0.04	$F0(x)$:=λx.if(flip(1/2), append(F1(x), x), pair(x, b)).<br>$F1(x)$:=λx.Fm0(pair(x, a)).
$a^nb^ma^{2n}$	abcd	1d	8	10 (10)	-182.123	0.17857	0.32	$F0(x)$:=λx.pair(insert(if(flip(1/2), Fm0(pair(ε, b)), pair(sample(Σ), a)), x), a).<br>$F1(x)$:=λx.append(pair(ε, a), pair(sample(if(not(flip(5/24)), F1(if(not(empty(x)), pair(x, a), pair(ε, b))), F0(x))), a)).
$a^nb^ma^{2n}$	abcd	1d	8	100 (35)	-505.408	1	1	$F0(x)$:=λx.pair(append(x, if(flip(3/8), pair(sample((ε∪pair(if(flip(5/12), ε, pair(if(flip(11/24), pair(if(flip(1/2), pair(if(flip(1/2), pair(ε, b), ε), b), ε), b), ε), b)), b))), b), F1(pair(ε, b))))), a).<br>$F1(x)$:=λx.pair(Fm0(if(not(flip(1/24)), pair(ε, a), insert(pair(append(pair(pair(ε, a), b), append(pair(ε, b), pair(ε, b))), b), x))), a).
$a^nb^ma^{2n}$	abcd	1d	8	1000 (100)	-6352.38	0.92	1	$F0(x)$:=λx.append(if(empty(x), pair(Fm1(pair(ε, a)), a), sample((x∪(append(pair(x, b), pair(if(flip(1/2), ε, append(x, insert(if(flip(5/24), x, ε), if(flip(1/2), ε, head(x))))), b))∪(ε∪head(x))))))), head(pair(x, a))).<br>$F1(x)$:=λx.append(x, F0(if(flip(if(empty(x), 1/24, 1/3)), pair(pair(if(flip(3/8), pair(ε, b), ε), b), b), ε))).
$a^nb^ma^{2n}$	abcd	1d	8	10000 (185)	-45726.4	1	1	$F0(x)$:=λx.append(pair(ε, a), pair(sample(if(not(flip(1/3)), pair(F0(x), a), append(pair(if(flip(5/12), x, append(if(flip(5/24), head(x), if(not(flip(7/24)), if(flip(1/12), append(x, x), ε), append(pair(if(flip(1/2), head(x), ε), b), x))), insert(x, x))), a), ε))), a)).<br>$F1(x)$:=λx.Fm0(append(pair(ε, b), if(flip(5/12), sample(if(flip(1/2), pair(pair(if(not(flip(7/24)), ε, pair(ε, b)), b), b), ε)), ε))).
$a^nb^ma^{2n}$	abcd	1d	8	100000 (330)	-415976	1	1	$F0(x)$:=λx.pair(append(x, pair(if(empty(x), ε, if(not(flip(7/24)), F1(ε), pair(F1(x), b))), a)), a).<br>$F1(x)$:=λx.if(not(or((append(ε, ε)==x), flip(3/8))), pair(F1(x), b), if((x==ε), F0(pair(ε, a)), ε)).
$a^nb^ma^{2n}$	abcd	1d	8	1 (1)	-46.3024	0.06666	0.04	$F0(x)$:=λx.append(F1(pair(x, a)), x).<br>$F1(x)$:=λx.if(flip(1/2), pair(x, b), F0(x)).<br>$F2(x)$:=λx.Fm0(ε).
$a^nb^ma^{2n}$	abcd	1d	8	10 (10)	-112.532	0.52	1	$F0(x)$:=λx.if(flip(1/4), ε, pair(Fm0(ε), b)).<br>$F1(x)$:=λx.append(x, if(flip(1/6), Fm0(ε), pair(pair(Fm1(pair(ε, a)), a), a))).<br>$F2(x)$:=λx.Fm1(ε).
$a^nb^ma^{2n}$	abcd	1d	8	100 (35)	-423.501	1	1	$F0(x)$:=λx.insert(F1(ε), pair(ε, b)).<br>$F1(x)$:=λx.if(flip(3/8), ε, Fm0(ε)).<br>$F2(x)$:=λx.append(pair(ε, a), pair(if(not(flip(3/8)), pair(Fm2(ε), a), pair(F0(ε), a)), a)).
$a^nb^ma^{2n}$	abcd	1d	8	1000 (100)	-3888.89	1	1	$F0(x)$:=λx.pair(F1(ε), b).<br>$F1(x)$:=λx.if(flip(1/3), ε, Fm0(ε)).<br>$F2(x)$:=λx.append(pair(ε, a), pair(if(not(flip(1/3)), pair(F2(ε), a), pair(Fm0(ε), a)), a)).
$a^nb^ma^{2n}$	abcd	1d	8	10000 (185)	-39568	1	1	$F0(x)$:=λx.ε.<br>$F1(x)$:=λx.if(not(and((x==ε), flip(1/3))), pair(append(x, F1(ε)), b), ε).<br>$F2(x)$:=λx.if(empty(if(not(flip(7/24)), ε, x)), append(Fm2(pair(x, a)), pair(pair(sample((sample(tail(if(empty(Fm0(ε)), ε, ε)))∪ε)), a), a)), Fm1(x)).
$a^nb^ma^{2n}$	abcd	1d	8	100000 (330)	-412933	1	1	$F0(x)$:=λx.sample(if(empty(insert(ε, x)), if(not(flip(1/3)), pair(Fm0(sample(ε)), b), if(empty(ε), append(append(pair(ε, a), ε), head(head(pair(tail(if(flip(1/24), pair(ε, b), ε)), b)))), ε)), F2(x))).<br>$F1(x)$:=λx.pair(Fm0(if(flip(1/3), ε, pair(ε, a))), a).<br>$F2(x)$:=λx.append(append(x, F1(ε)), pair(ε, a)).
$a^nb^ma^{2n}$	abcd	1d	8	1 (1)	-55.8259	0.0625	0.04	$F0(x)$:=λx.append(sample(Σ), append(x, x)).<br>$F1(x)$:=λx.append(x, Fm0(x)).<br>$F2(x)$:=λx.F1(pair(x, a)).<br>$F3(x)$:=λx.F2(Fm2(ε)).
$a^nb^ma^{2n}$	abcd	1d	8	10 (10)	-115.065	1	1	$F0(x)$:=λx.append(append(pair(x, b), append(if(flip(1/2), Fm0(ε), ε), x)), x).<br>$F1(x)$:=λx.if(flip(1/4), ε, Fm2(ε)).<br>$F2(x)$:=λx.pair(Fm1(ε), a).<br>$F3(x)$:=λx.Fm0(F2(ε)).
$a^nb^ma^{2n}$	abcd	1d	8	100 (35)	-425.518	1	1	$F0(x)$:=λx.append(append(pair(x, b), append(if(flip(1/2), Fm0(ε), ε), x)), x).<br>$F1(x)$:=λx.if(flip(3/8), ε, Fm2(ε)).<br>$F2(x)$:=λx.pair(Fm1(ε), a).<br>$F3(x)$:=λx.Fm0(F2(ε)).
$a^nb^ma^{2n}$	abcd	1d	8	1000 (100)	-3897.36	1	1	$F0(x)$:=λx.pair(F3(ε), a).<br>$F1(x)$:=λx.ε.<br>$F2(x)$:=λx.pair(if(not(flip(1/3)), Fm2(ε), Fm1(ε)), b).<br>$F3(x)$:=λx.pair(append(pair(ε, a), if(flip(1/3), pair(F2(ε), a), F0(ε))), a).

$a^nb^ma^{2n}$	abcd	1d	8	10000 (185)	-40022.7	1	1	F0(x):=λx.append(x, sample(if(and((ϵ==x), flip(1/4)), ϵ, append(pair(F0(ϵ), b), append(x, x))))).<br>F1(x):=λx.pair(Fm2(ϵ), a).<br>F2(x):=λx.if(not(flip(5/24)), F1(ϵ), ϵ).<br>F3(x):=λx.F0(pair(if(flip(1/3), ϵ, Fm1(if(flip(1/8), Fm2(ϵ), ϵ))), a)).
$a^nb^ma^{2n}$	abcd	1d	8	100000 (330)	-412911	1	1	F0(x):=λx.ϵ.<br>F1(x):=λx.if(not(flip(1/3)), pair(Fm1(x), b), pair(pair(x, a), b)).<br>F2(x):=λx.if((head(x)==x), if(flip(1/4), ϵ, sample((Σ∪x))), ϵ).<br>F3(x):=λx.pair(pair(if(flip(1/3), F1(append(x, Fm0(F2(x)))), Fm3(pair(x, a))), a), a).
$a^nb^nc^{2n}$	abcd	1d	8	1 (1)	-20.0355	1	0.04	F0(x):=λx.pair(pair(pair(pair(ϵ, a), b), c), c).
$a^nb^nc^{2n}$	abcd	1d	8	10 (3)	-50.565	1	0.84	F0(x):=λx.append(pair(ϵ, a), pair(pair(insert(if(flip(1/2), Fm0(ϵ), ϵ), pair(ϵ, b)), c), c)).
$a^nb^nc^{2n}$	abcd	1d	8	100 (9)	-224.164	1	1	F0(x):=λx.append(pair(ϵ, a), insert(pair(if(flip(3/8), ϵ, F0(ϵ)), c), pair(pair(ϵ, b), c))).
$a^nb^nc^{2n}$	abcd	1d	8	1000 (17)	-1983.88	1	1	F0(x):=λx.append(pair(ϵ, a), insert(pair(if(flip(1/3), ϵ, Fm0(ϵ)), c), pair(pair(ϵ, b), c))).
$a^nb^nc^{2n}$	abcd	1d	8	10000 (20)	-19178.9	1	1	F0(x):=λx.append(pair(ϵ, a), insert(pair(if(flip(1/3), ϵ, Fm0(ϵ)), c), pair(pair(ϵ, b), c))).
$a^nb^nc^{2n}$	abcd	1d	8	100000 (27)	-192641	1	1	F0(x):=λx.append(pair(ϵ, a), insert(pair(if(flip(1/3), x, F0(ϵ)), c), pair(pair(ϵ, b), c))).
$a^nb^nc^{2n}$	abcd	1d	8	1 (1)	-26.6602	1	0.04	F0(x):=λx.pair(ϵ, a).<br>F1(x):=λx.pair(pair(pair(F0(ϵ), b), c), c).
$a^nb^nc^{2n}$	abcd	1d	8	10 (3)	-54.5007	1	0.84	F0(x):=λx.append(pair(ϵ, a), if(flip(1/2), F1(x), x)).<br>F1(x):=λx.pair(pair(F0(pair(x, b)), c), c).
$a^nb^nc^{2n}$	abcd	1d	8	100 (9)	-228.1	1	1	F0(x):=λx.append(pair(ϵ, a), pair(if(flip(3/8), x, Fm1(x)), c)).<br>F1(x):=λx.pair(F0(pair(x, b)), c).
$a^nb^nc^{2n}$	abcd	1d	8	1000 (17)	-1987.82	1	1	F0(x):=λx.append(pair(ϵ, a), pair(if(flip(1/3), x, F1(x)), c)).<br>F1(x):=λx.pair(F0(pair(x, b)), c).
$a^nb^nc^{2n}$	abcd	1d	8	10000 (20)	-19182.9	1	1	F0(x):=λx.append(pair(ϵ, a), pair(if(flip(1/3), x, F1(x)), c)).<br>F1(x):=λx.pair(F0(pair(x, b)), c).
$a^nb^nc^{2n}$	abcd	1d	8	100000 (27)	-192645	1	1	F0(x):=λx.append(pair(ϵ, a), pair(if(flip(1/3), x, F1(x)), c)).<br>F1(x):=λx.pair(F0(pair(x, b)), c).
$a^nb^nc^{2n}$	abcd	1d	8	1 (1)	-34.0957	1	0.04	F0(x):=λx.pair(pair(F1(ϵ), a), b).<br>F1(x):=λx.ϵ.<br>F2(x):=λx.pair(pair(Fm0(ϵ), c), c).
$a^nb^nc^{2n}$	abcd	1d	8	10 (3)	-62.3417	1	0.84	F0(x):=λx.pair(if(flip(1/2), F2(x), x), c).<br>F1(x):=λx.ϵ.<br>F2(x):=λx.pair(F0(append(pair(Fm1(ϵ), a), pair(x, b))), c).
$a^nb^nc^{2n}$	abcd	1d	8	100 (9)	-236.634	1	0.88	F0(x):=λx.append(pair(ϵ, a), F1(x)).<br>F1(x):=λx.pair(pair(if(flip(3/8), x, F2(x)), c), c).<br>F2(x):=λx.F0(pair(x, b)).
$a^nb^nc^{2n}$	abcd	1d	8	1000 (17)	-1998.37	1	1	F0(x):=λx.append(pair(ϵ, a), if(not(flip(1/3)), Fm1(x), x)).<br>F1(x):=λx.pair(pair(Fm0(pair(x, b)), c), c).<br>F2(x):=λx.F1(ϵ).
$a^nb^nc^{2n}$	abcd	1d	8	10000 (20)	-19191.4	1	0.88	F0(x):=λx.append(pair(ϵ, a), F1(x)).<br>F1(x):=λx.pair(pair(if(flip(1/3), x, Fm2(x)), c), c).<br>F2(x):=λx.F0(pair(x, b)).
$a^nb^nc^{2n}$	abcd	1d	8	100000 (27)	-192655	1	1	F0(x):=λx.append(pair(ϵ, a), if(not(flip(1/3)), Fm1(x), x)).<br>F1(x):=λx.pair(pair(Fm0(pair(x, b)), c), c).<br>F2(x):=λx.F1(ϵ).
$a^nb^nc^{2n}$	abcd	1d	8	1 (1)	-41.9888	1	0.04	F0(x):=λx.Fm1(ϵ).<br>F1(x):=λx.pair(pair(ϵ, a), b).<br>F2(x):=λx.pair(pair(F0(ϵ), c), c).<br>F3(x):=λx.F2(ϵ).
$a^nb^nc^{2n}$	abcd	1d	8	10 (3)	-70.5225	1	0.64	F0(x):=λx.ϵ.<br>F1(x):=λx.pair(pair(if(flip(1/2), x, F3(x)), c), c).<br>F2(x):=λx.Fm0(ϵ).<br>F3(x):=λx.append(pair(F2(ϵ), a), F1(pair(x, b))).
$a^nb^nc^{2n}$	abcd	1d	8	100 (9)	-244.122	1	0.84	F0(x):=λx.ϵ.<br>F1(x):=λx.pair(pair(if(flip(3/8), x, Fm3(x)), c), c).<br>F2(x):=λx.pair(F0(ϵ), a).<br>F3(x):=λx.append(Fm2(ϵ), F1(pair(x, b))).
$a^nb^nc^{2n}$	abcd	1d	8	1000 (17)	-2014.03	1	1	F0(x):=λx.pair(if((if(flip(1/3), x, ϵ)==ϵ), F3(x), F1(Fm2(x))), c).<br>F1(x):=λx.x.<br>F2(x):=λx.x.<br>F3(x):=λx.pair(Fm0(append(pair(ϵ, a), pair(x, b))), c).

$a^nb^nc^{2n}$	abcd	1d	8	10000 (20)	-19198.9	1	0.84	F0(x):=λx.Fm1(ϵ).<br>F1(x):=λx.ϵ.<br>F2(x):=λx.if(flip(1/3), x, F3(x)).<br>F3(x):=λx.pair(pair(append(pair(Fm0(ϵ), a), Fm2(pair(x, b))), c), c).
$a^nb^nc^{2n}$	abcd	1d	8	100000 (27)	-192662	1	1	F0(x):=λx.x.<br>F1(x):=λx.F0(x).<br>F2(x):=λx.if(flip(1/3), F1(x), Fm3(x)).<br>F3(x):=λx.pair(pair(append(pair(ϵ, a), Fm2(pair(x, b))), c), c).
$\Sigma^+a^nb^n$	abcd	7d	8	1 (1)	-77.6536	0	0	F0(x):=λx.pair(if(flip(1/2), if(flip(1/2), append(append(pair(pair(Fm0(ϵ), a), b), x), pair(pair(Fm0(ϵ), b), b)), x), Fm0(append(x, pair(head(x), a)))), b).
$\Sigma^+a^nb^n$	abcd	7d	8	10 (10)	-251.547	0.07692	0	F0(x):=λx.sample(if(not(flip(1/6)), if(flip(11/24), if(not(flip(5/24)), pair(F0(pair(pair(pair(pair(ϵ, a), a), b), b)), b), ϵ), append(pair(if(flip(1/2), F0(ϵ), pair(ϵ, a)), a), insert(x, x))), pair(pair(pair(if(flip(1/2), append(pair(ϵ, a), x), ϵ), b), a), b))).
$\Sigma^+a^nb^n$	abcd	7d	8	100 (90)	-3443.86	0	0.76	F0(x):=λx.append(if(not(flip(5/24)), Fm0(pair(x, a)), pair(if(flip(11/24), append(if(flip(1/6), pair(if(not((head(x)==x)), ϵ, if((ϵ==x), ϵ, pair(x, a))), b), ϵ), append(if(flip(1/2), ϵ, pair(if(flip(1/2), if(flip(5/12), pair(ϵ, a), F0(ϵ)), ϵ), b)), x)), pair(x, a)), a)), pair(ϵ, b)).
$\Sigma^+a^nb^n$	abcd	7d	8	1000 (599)	-32734.7	0	0.76	F0(x):=λx.insert(head(pair(ϵ, b)), sample(if(not(flip(if((ϵ==x), 1/12, 1/6))), if(not(and(not(empty(x)), flip(1/8))), Fm0(pair(x, a)), x), if(not(flip(7/24)), (append(x, pair(ϵ, a)) ∪ (append(pair(x, b), ϵ) ∪ (ϵ ∪ ϵ))), pair(append(Fm0(pair(ϵ, a)), x), a))))).
$\Sigma^+a^nb^n$	abcd	7d	8	10000 (3271)	-343349	0	0.76	F0(x):=λx.pair(if(flip(1/3), append(if(flip(11/24), pair(if(flip(1/12), sample((pair(pair(ϵ, a), b) ∪ pair(pair(ϵ, b), b))), ϵ), b), if(flip(1/6), pair(insert(F0(ϵ), if(flip(5/12), pair(ϵ, a), ϵ)), a), pair(ϵ, a))), x), F0(pair(x, a))), b).
$\Sigma^+a^nb^n$	abcd	7d	8	100000 (15092)	-3.44542e+06	0	0.76	F0(x):=λx.pair(if(flip(1/3), append(if(flip(11/24), pair(if(flip(1/12), sample((pair(pair(ϵ, a), b) ∪ pair(pair(ϵ, b), b))), ϵ), b), if(flip(1/6), pair(insert(F0(ϵ), if(flip(5/12), pair(ϵ, a), ϵ)), a), pair(ϵ, a))), x), F0(pair(x, a))), b).
$\Sigma^+a^nb^n$	abcd	7d	8	1 (1)	-62.4755	0.76923	0	F0(x):=λx.append(pair(ϵ, a), if(flip(1/8), ϵ, append(F0(ϵ), pair(x, b)))).<br>F1(x):=λx.append(Fm0(pair(ϵ, a)), pair(Fm0(ϵ), b)).
$\Sigma^+a^nb^n$	abcd	7d	8	10 (10)	-250.489	0.25925	0.24	F0(x):=λx.if(not(flip(7/24)), pair(F0(pair(x, a)), b), if(flip(1/3), pair(if(flip(1/2), pair(ϵ, b), pair(x, b)), a), pair(x, b))).<br>F1(x):=λx.F0(pair(if(flip(1/2), Fm0(ϵ), ϵ), a)).
$\Sigma^+a^nb^n$	abcd	7d	8	100 (90)	-3460.85	0.8	0.76	F0(x):=λx.pair(append(x, if(flip(1/4), if(flip(5/24), ϵ, pair(ϵ, a)), F0(pair(ϵ, a)))), b).<br>F1(x):=λx.F0(if(flip(5/24), if(flip(11/24), if(flip(1/8), pair(pair(ϵ, b), b), pair(if(not(flip(1/3)), ϵ, pair(ϵ, b)), a)), F0(pair(ϵ, b))), F0(ϵ))).
$\Sigma^+a^nb^n$	abcd	7d	8	1000 (599)	-30911.9	0.88	0.88	F0(x):=λx.pair(if(flip(3/8), pair(x, a), append(pair(x, a), F0(ϵ))), b).<br>F1(x):=λx.Fm0(sample(if(flip(1/6), pair(F0(ϵ), b), if(flip(1/6), pair(pair(if(or(flip(1/8), flip(1/2)), Fm0(ϵ), ϵ), b), b), F0(if(flip(3/8), sample(if(flip(5/12), pair(ϵ, b), pair(head(if(flip(3/8), ϵ, Fm0(ϵ))), a))), ϵ)))))).
$\Sigma^+a^nb^n$	abcd	7d	8	10000 (3271)	-324203	0.88	0.88	F0(x):=λx.pair(if(flip(3/8), pair(x, a), append(pair(x, a), F0(ϵ))), b).<br>F1(x):=λx.Fm0(sample(if(flip(1/6), pair(F0(ϵ), b), if(flip(1/6), pair(pair(if(or(flip(1/8), flip(1/2)), Fm0(ϵ), ϵ), b), b), F0(if(flip(3/8), sample(if(flip(5/12), pair(ϵ, b), pair(head(if(flip(3/8), ϵ, Fm0(ϵ))), a))), ϵ)))))).
$\Sigma^+a^nb^n$	abcd	7d	8	100000 (15092)	-3.24964e+06	0.88	0.88	F0(x):=λx.pair(if(flip(3/8), pair(x, a), append(pair(x, a), F0(ϵ))), b).<br>F1(x):=λx.Fm0(sample(if(flip(1/6), pair(F0(ϵ), b), if(flip(1/6), pair(append(x, pair(if(or(flip(1/8), flip(1/2)), F0(x), ϵ), b)), b), F0(if(flip(3/8), sample(if(flip(5/12), pair(ϵ, b), pair(head(if(flip(3/8), x, F0(ϵ))), a))), x)))))).
$\Sigma^+a^nb^n$	abcd	7d	8	1 (1)	-63.9262	0.72	0	F0(x):=λx.append(x, F1(ϵ)).<br>F1(x):=λx.if(flip(1/8), ϵ, pair(append(F0(pair(x, a)), x), b)).<br>F2(x):=λx.F0(F1(pair(ϵ, a))).
$\Sigma^+a^nb^n$	abcd	7d	8	10 (10)	-195.728	0.78571	0.68	F0(x):=λx.pair(if(not(flip(7/24)), Fm0(pair(if((x==ϵ), if(not(flip(1/3)), ϵ, sample(Σ)), x), a)), x), b).<br>F1(x):=λx.pair(sample((head(x) ∪ pair(x, b))), a).<br>F2(x):=λx.F0(pair(F0(if(flip(1/2), ϵ, Fm1(F1(ϵ)))), a)).
$\Sigma^+a^nb^n$	abcd	7d	8	100 (90)	-3113.74	0.72727	0.88	F0(x):=λx.pair(if(flip(1/12), if(flip(1/8), pair(ϵ, a), pair(ϵ, b)), ϵ), a).<br>F1(x):=λx.pair(if(flip(1/3), x, F1(pair(x, a))), b).<br>F2(x):=λx.if(flip(1/12), pair(pair(pair(pair(pair(F1(pair(Fm0(ϵ), a)), a), a), a), a), a), F1(if(flip(1/2), append(F0(ϵ), pair(F1(ϵ), a)), pair(F1(if(flip(1/12), pair(if(flip(1/4), pair(ϵ, a), ϵ), b), if(flip(1/2), ϵ, ϵ))), a)))).
$\Sigma^+a^nb^n$	abcd	7d	8	1000 (599)	-28921.4	0.52	0.84	F0(x):=λx.pair(if(flip(1/2), x, pair(if(not(and(flip(1/8), or(flip(7/24), flip(1/4)))), x, pair(x, a)), a)), b).<br>F1(x):=λx.insert(pair(if(not(flip(1/4)), Fm1(x), if(flip(5/24), x, ϵ)), b), pair(ϵ, a)).<br>F2(x):=λx.append(sample(if(not(flip(5/12)), F1(pair(ϵ, a)), (Fm0(pair(ϵ, b)) ∪ ϵ))), F1(pair(ϵ, b))).
$\Sigma^+a^nb^n$	abcd	7d	8	10000 (3271)	-327515	0.8	0.8	F0(x):=λx.Fm1(if(not(and(empty(x), flip(1/4))), pair(x, a), if(flip(5/24), pair(if(flip(1/4), F0(if(or(flip(1/24), flip(1/6)), ϵ, ϵ)), ϵ), b), ϵ))).<br>F1(x):=λx.pair(if(not(flip(11/24)), pair(if(flip(7/24), insert(pair(if(flip(1/3), if(and(not((ϵ==x)), flip(1/2)), pair(if(flip(1/6), head(x), ϵ), a), pair(ϵ, b)), ϵ), a), x), Fm0(pair(x, a))), b), x), b).<br>F2(x):=λx.Fm0(F0(x)).
$\Sigma^+a^nb^n$	abcd	7d	8	100000 (15092)	-3.27308e+06	0.8	0.8	F0(x):=λx.Fm1(if(not(and((ϵ==x), flip(1/4))), pair(x, a), if(flip(5/24), pair(if(flip(1/4), F0(if(or(flip(1/24), flip(1/6)), ϵ, ϵ)), ϵ), b), ϵ))).<br>F1(x):=λx.pair(if(not(flip(11/24)), pair(if(flip(7/24), insert(pair(if(flip(1/3), if(and(not((x==append(ϵ, ϵ))), flip(1/2)), pair(if(flip(1/6), head(x), ϵ), a), pair(ϵ, b)), tail(ϵ)), a), x), Fm0(pair(x, a))), b), x), b).<br>F2(x):=λx.Fm0(Fm0(ϵ)).
$\Sigma^+a^nb^n$	abcd	7d	8	1 (1)	-74.8604	0.53846	0	F0(x):=λx.append(pair(ϵ, a), append(F2(x), x)).<br>F1(x):=λx.ϵ.<br>F2(x):=λx.if(flip(1/8), Fm1(ϵ), append(F0(ϵ), pair(head(x), b))).<br>F3(x):=λx.Fm0(F2(ϵ)).

$\Sigma^+a^nb^n$	abcd	7d	8	10 (10)	-205.017	0.24	0.48	$F0(x)$:=λx.if(flip(1/2), x, pair(append(pair(pair(ε, a), a), pair(F0(ε), b)), b)).<br>$F1(x)$:=λx.pair(append(F2(ε), if(flip(1/2), pair(if(flip(1/3), Fm0(pair(ε, b)), ε), b), ε)), a).<br>$F2(x)$:=λx.if(flip(1/3), Fm1(ε), ε).<br>$F3(x)$:=λx.append(Fm1(ε), pair(F0(ε), b)).
$\Sigma^+a^nb^n$	abcd	7d	8	100 (90)	-3206.14	0.44	0.72	$F0(x)$:=λx.if(flip(if(empty(x), 1/24, 7/24)), x, pair(if(flip(1/24), if(and(empty(x), flip(1/2)), pair(pair(ε, a), b), pair(x, b)), F2(x)), b)).<br>$F1(x)$:=λx.Fm0(ε).<br>$F2(x)$:=λx.Fm0(pair(if(and((ε==x), flip(1/8)), pair(ε, b), x), a)).<br>$F3(x)$:=λx.if(not(flip(5/24)), Fm0(F1(ε)), pair(pair(pair(pair(pair(Fm2(ε), a), a), a), a), a)).
$\Sigma^+a^nb^n$	abcd	7d	8	1000 (599)	-29622.3	0.65384	0.64	$F0(x)$:=λx.F1(insert(x, F2(ε))).<br>$F1(x)$:=λx.sample(if(not(flip(1/3)), F0(x), x)).<br>$F2(x)$:=λx.pair(pair(ε, a), b).<br>$F3(x)$:=λx.append(F1(sample(if(not(flip(11/24)), pair(Fm2(ε), a), if(flip(1/2), pair(ε, b), pair(pair(if(flip(1/6), pair(if(not(flip(7/24)), ε, pair(ε, b)), b), if(flip(5/12), ε, x)), a), a))))), Fm1(pair(ε, b))).
$\Sigma^+a^nb^n$	abcd	7d	8	10000 (3271)	-318498	0.4	0.84	$F0(x)$:=λx.pair(Fm2(x), b).<br>$F1(x)$:=λx.if(flip(11/24), insert(x, append(pair(sample((pair(ε, a) ∪ ε)), b), if(flip(1/8), pair(ε, b), ε))), x).<br>$F2(x)$:=λx.if(flip(7/24), x, Fm0(pair(x, a))).<br>$F3(x)$:=λx.pair(F2(if(flip(7/24), pair(Fm2(if(flip(5/24), pair(ε, a), ε)), a), F1(F1(pair(ε, a))))), b).
$\Sigma^+a^nb^n$	abcd	7d	8	100000 (15092)	-3.19642e+06	0.4	0.84	$F0(x)$:=λx.pair(F2(x), b).<br>$F1(x)$:=λx.if(flip(11/24), insert(x, append(pair(sample((append(pair(ε, a), tail(ε)) ∪ ε)), b), if(flip(1/8), pair(ε, b), ε))), x).<br>$F2(x)$:=λx.if(flip(7/24), x, Fm0(pair(x, a))).<br>$F3(x)$:=λx.pair(F2(if(flip(7/24), pair(Fm2(if(flip(5/24), pair(ε, a), x)), a), F1(F1(pair(ε, a))))), b).
$a^nb^mc^m$	abcd	4h	8	1 (1)	-31.7805	0.96	0.28	$F0(x)$:=λx.if(flip(1/12), pair(pair(x, b), c), F0(pair(x, a))).
$a^nb^mc^m$	abcd	4h	8	10 (7)	-105.571	0.64	0.72	$F0(x)$:=λx.append(if(or(empty(x), flip(1/3)), pair(ε, a), ε), sample(if(flip(1/2), pair(Fm0(pair(x, b)), c), if(flip(5/24), pair(pair(ε, a), b), x)))).
$a^nb^mc^m$	abcd	4h	8	100 (42)	-2452.02	0.64	0.76	$F0(x)$:=λx.append(if(or((x==ε), flip(7/24)), pair(ε, a), ε), sample(if(or(empty(x), flip(1/2)), pair(Fm0(pair(x, b)), c), if(flip(1/4), if(flip(1/8), append(append(x, x), pair(x, c)), insert(head(x), pair(pair(ε, a), a))), x)))).
$a^nb^mc^m$	abcd	4h	8	1000 (97)	-14074.5	0.28	0.28	$F0(x)$:=λx.append(pair(if(flip(7/24), ε, F0(pair(x, b))), a), if(flip(if(empty(x), 11/24, 1/12)), pair(if(empty(x), pair(if(flip(1/2), ε, pair(if(flip(5/12), sample(Σ), ε), b)), b), pair(pair(append(append(if(flip(1/2), pair(ε, b), ε), pair(x, b)), pair(pair(pair(x, b), c), c)), c), c)), c), ε)).
$a^nb^mc^m$	abcd	4h	8	10000 (207)	-145485	0.22222	0.28	$F0(x)$:=λx.append(pair(if(flip(7/24), ε, F0(pair(x, b))), a), if(flip(if(empty(x), 11/24, 1/12)), pair(if(empty(x), pair(if(flip(11/24), x, pair(if(flip(5/12), sample(Σ), ε), b)), b), pair(pair(append(append(if(flip(1/2), pair(ε, b), ε), pair(x, b)), pair(pair(pair(x, b), c), c)), c), c)), c), ε)).
$a^nb^mc^m$	abcd	4h	8	100000 (330)	-1.43375e+06	0.22222	0.28	$F0(x)$:=λx.append(pair(if(flip(7/24), ε, F0(pair(x, b))), a), if(flip(if(empty(x), 11/24, 1/12)), pair(if(empty(x), pair(if(flip(11/24), x, pair(if(flip(5/12), sample(Σ), ε), b)), b), pair(pair(append(append(if(flip(1/2), pair(ε, b), ε), pair(x, b)), pair(pair(pair(x, b), c), c)), c), c)), c), ε)).
$a^nb^mc^m$	abcd	4h	8	1 (1)	-37.5917	1	0.28	$F0(x)$:=λx.pair(if(flip(1/8), ε, Fm0(ε)), a).<br>$F1(x)$:=λx.pair(pair(F0(ε), b), c).
$a^nb^mc^m$	abcd	4h	8	10 (7)	-140.447	0.36	0.6	$F0(x)$:=λx.if(flip(1/6), ε, x).<br>$F1(x)$:=λx.insert(sample(if(flip(5/12), pair(pair(pair(Fm0(x), a), b), c), pair(Fm1(pair(ε, a)), c))), if(flip(7/24), x, pair(ε, b))).
$a^nb^mc^m$	abcd	4h	8	100 (42)	-480.426	1	1	$F0(x)$:=λx.if(not(flip(7/24)), pair(Fm0(ε), a), ε).<br>$F1(x)$:=λx.append(Fm0(ε), insert(append(if(flip(1/3), pair(ε, a), F1(ε)), pair(ε, c)), pair(ε, b))).
$a^nb^mc^m$	abcd	4h	8	1000 (97)	-4149.56	1	1	$F0(x)$:=λx.if(not(flip(1/3)), pair(Fm0(ε), a), ε).<br>$F1(x)$:=λx.append(Fm0(ε), insert(append(if(flip(7/24), pair(ε, a), Fm1(ε)), pair(ε, c)), pair(ε, b))).
$a^nb^mc^m$	abcd	4h	8	10000 (207)	-40748.8	1	1	$F0(x)$:=λx.if(not(flip(1/3)), pair(Fm0(ε), a), ε).<br>$F1(x)$:=λx.append(Fm0(ε), insert(append(if(flip(7/24), pair(ε, a), Fm1(ε)), pair(ε, c)), pair(ε, b))).
$a^nb^mc^m$	abcd	4h	8	100000 (330)	-397765	1	1	$F0(x)$:=λx.if(not(flip(1/3)), pair(Fm0(ε), a), ε).<br>$F1(x)$:=λx.append(Fm0(ε), insert(append(if(flip(7/24), pair(ε, a), Fm1(ε)), pair(ε, c)), pair(ε, b))).
$a^nb^mc^m$	abcd	4h	8	1 (1)	-45.4327	1	0.28	$F0(x)$:=λx.pair(if(flip(1/8), ε, F0(ε)), a).<br>$F1(x)$:=λx.pair(Fm0(ε), b).<br>$F2(x)$:=λx.pair(F1(ε), c).
$a^nb^mc^m$	abcd	4h	8	10 (7)	-100.821	1	1	$F0(x)$:=λx.ε.<br>$F1(x)$:=λx.pair(if(flip(1/2), ε, Fm1(ε)), a).<br>$F2(x)$:=λx.pair(append(pair(if(empty(x), F1(Fm0(ε)), ε), b), if(flip(1/2), F2(pair(ε, a)), ε)), c).
$a^nb^mc^m$	abcd	4h	8	100 (42)	-489.052	1	1	$F0(x)$:=λx.Fm1(ε).<br>$F1(x)$:=λx.pair(if(flip(7/24), ε, Fm1(x)), a).<br>$F2(x)$:=λx.pair(append(pair(if((x==ε), Fm0(ε), ε), b), if(not(flip(7/24)), F2(pair(ε, c)), ε)), c).
$a^nb^mc^m$	abcd	4h	8	1000 (97)	-4156.52	1	1	$F0(x)$:=λx.F1(ε).<br>$F1(x)$:=λx.pair(if(flip(1/3), ε, F0(ε)), a).<br>$F2(x)$:=λx.pair(append(pair(if(empty(x), Fm1(ε), ε), b), if(not(flip(7/24)), F2(pair(ε, b)), ε)), c).
$a^nb^mc^m$	abcd	4h	8	10000 (207)	-40379.6	1	1	$F0(x)$:=λx.F1(ε).<br>$F1(x)$:=λx.pair(if(flip(3/8), ε, Fm0(ε)), a).<br>$F2(x)$:=λx.pair(append(pair(if(empty(x), Fm1(ε), ε), b), if(not(flip(7/24)), F2(pair(ε, d)), ε)), c).

$a^nb^mc^m$	abcd	4h	8	100000 (330)	-397733	1	1	$F0(x)$:=λx.F1(ε).<br>$F1(x)$:=λx.pair(if(flip(3/8), ε, Fm0(ε)), a).<br>$F2(x)$:=λx.pair(append(pair(if(empty(x), Fm0(x), ε), b), if(not(flip(1/3)), F2(pair(ε, a)), ε)), c).
$a^nb^mc^m$	abcd	4h	8	1 (1)	-54.4093	1	0.28	$F0(x)$:=λx.pair(if(flip(1/6), ε, Fm1(ε)), a).<br>$F1(x)$:=λx.Fm0(ε).<br>$F2(x)$:=λx.ε.<br>$F3(x)$:=λx.pair(pair(F0(Fm2(x)), b), c).
$a^nb^mc^m$	abcd	4h	8	10 (7)	-102.648	1	1	$F0(x)$:=λx.append(if(flip(1/2), pair(x, b), F0(pair(x, b))), pair(ε, c)).<br>$F1(x)$:=λx.if(flip(1/2), F2(ε), ε).<br>$F2(x)$:=λx.pair(Fm1(ε), a).<br>$F3(x)$:=λx.Fm0(F2(ε)).
$a^nb^mc^m$	abcd	4h	8	100 (42)	-489.862	1	1	$F0(x)$:=λx.pair(x, a).<br>$F1(x)$:=λx.append(pair(ε, b), if(flip(7/24), ε, pair(Fm1(ε), c))).<br>$F2(x)$:=λx.Fm0(if(flip(7/24), ε, Fm2(ε))).<br>$F3(x)$:=λx.append(Fm2(ε), pair(F1(ε), c)).
$a^nb^mc^m$	abcd	4h	8	1000 (97)	-4143.95	1	1	$F0(x)$:=λx.Fm1(tail(F2(ε))).<br>$F1(x)$:=λx.Fm2(append(pair(x, b), sample(if(not(flip(1/3)), F0(ε), ε)))).<br>$F2(x)$:=λx.pair(x, c).<br>$F3(x)$:=λx.if(flip(3/8), F1(pair(ε, a)), append(pair(ε, a), F3(ε))).
$a^nb^mc^m$	abcd	4h	8	10000 (207)	-40350	1	1	$F0(x)$:=λx.append(pair(ε, b), append(if(flip(7/24), ε, Fm0(F2(ε))), pair(ε, c))).<br>$F1(x)$:=λx.pair(if(not(flip(3/8)), F1(ε), ε), a).<br>$F2(x)$:=λx.ε.<br>$F3(x)$:=λx.pair(append(pair(F1(ε), b), if(flip(3/8), ε, Fm0(ε))), c).
$a^nb^mc^m$	abcd	4h	8	100000 (330)	-397831	1	1	$F0(x)$:=λx.append(pair(F2(ε), b), append(if(flip(7/24), x, Fm0(Fm2(ε))), pair(ε, c))).<br>$F1(x)$:=λx.pair(if(not(flip(1/3)), F1(ε), ε), a).<br>$F2(x)$:=λx.ε.<br>$F3(x)$:=λx.pair(append(pair(F1(ε), b), if(flip(1/3), ε, Fm0(ε))), c).
$a^nb^mc^{n+m}$	abcd	4d	8	1 (1)	-28.9627	1	0.04	$F0(x)$:=λx.pair(pair(pair(pair(pair(pair(ε, a), a), b), c), c), c).
$a^nb^mc^{n+m}$	abcd	4d	8	10 (8)	-86.8913	0.76	1	$F0(x)$:=λx.append(head(pair(x, a)), pair(if(flip(1/4), ε, Fm0(if(flip(1/3), pair(ε, b), x))), c)).
$a^nb^mc^{n+m}$	abcd	4d	8	100 (40)	-435.264	1	1	$F0(x)$:=λx.append(head(pair(x, a)), insert(if(flip(1/3), if(empty(x), F0(pair(ε, b)), ε), Fm0(x)), pair(ε, c))).
$a^nb^mc^{n+m}$	abcd	4d	8	1000 (96)	-3876.72	1	1	$F0(x)$:=λx.append(head(pair(x, a)), insert(if(flip(1/3), if(empty(x), F0(pair(ε, b)), ε), Fm0(x)), pair(ε, c))).
$a^nb^mc^{n+m}$	abcd	4d	8	10000 (201)	-41099	1	1	$F0(x)$:=λx.append(head(pair(x, a)), insert(if(flip(3/8), if(empty(x), Fm0(pair(ε, b)), ε), Fm0(x)), pair(ε, c))).
$a^nb^mc^{n+m}$	abcd	4d	8	100000 (326)	-411730	1	1	$F0(x)$:=λx.append(head(pair(x, a)), insert(if(flip(1/3), if(empty(x), F0(pair(x, b)), ε), Fm0(x)), pair(ε, c))).
$a^nb^mc^{n+m}$	abcd	4d	8	1 (1)	-35.5874	1	0.04	$F0(x)$:=λx.ε.<br>$F1(x)$:=λx.pair(pair(pair(pair(pair(pair(Fm0(ε), a), a), b), c), c), c).
$a^nb^mc^{n+m}$	abcd	4d	8	10 (8)	-89.173	1	1	$F0(x)$:=λx.append(head(pair(x, a)), pair(if(flip(1/2), F0(pair(ε, b)), if(empty(x), Fm1(ε), ε)), c)).<br>$F1(x)$:=λx.F0(ε).
$a^nb^mc^{n+m}$	abcd	4d	8	100 (40)	-440.152	1	1	$F0(x)$:=λx.append(if(not(empty(x)), pair(ε, a), pair(ε, b)), pair(if(not(flip(1/3)), Fm0(x), x), c)).<br>$F1(x)$:=λx.Fm0(Fm0(ε)).
$a^nb^mc^{n+m}$	abcd	4d	8	1000 (96)	-3881.61	1	1	$F0(x)$:=λx.append(if(not(empty(x)), pair(ε, a), pair(ε, b)), pair(if(not(flip(1/3)), Fm0(x), x), c)).<br>$F1(x)$:=λx.Fm0(Fm0(ε)).
$a^nb^mc^{n+m}$	abcd	4d	8	10000 (201)	-41116.6	1	1	$F0(x)$:=λx.sample(if(flip(3/8), x, insert(pair(x, c), F0(x)))).<br>$F1(x)$:=λx.append(pair(ε, a), pair(if(flip(3/8), pair(F0(pair(ε, b)), c), Fm1(ε)), c)).
$a^nb^mc^{n+m}$	abcd	4d	8	100000 (326)	-411227	1	1	$F0(x)$:=λx.append(if(not(empty(x)), pair(ε, a), pair(ε, b)), pair(if(not(flip(1/3)), Fm0(x), x), c)).<br>$F1(x)$:=λx.Fm0(Fm0(ε)).
$a^nb^mc^{n+m}$	abcd	4d	8	1 (1)	-43.0229	1	0.04	$F0(x)$:=λx.pair(F1(ε), c).<br>$F1(x)$:=λx.pair(pair(pair(pair(ε, a), a), b), c).<br>$F2(x)$:=λx.pair(F0(ε), c).
$a^nb^mc^{n+m}$	abcd	4d	8	10 (8)	-106.144	0.76	1	$F0(x)$:=λx.pair(x, c).<br>$F1(x)$:=λx.append(head(pair(x, a)), if(flip(1/4), ε, F2(if(not(flip(1/3)), x, pair(ε, b))))).<br>$F2(x)$:=λx.Fm0(Fm1(x)).
$a^nb^mc^{n+m}$	abcd	4d	8	100 (40)	-449.44	1	1	$F0(x)$:=λx.if(not(flip(1/3)), F2(ε), pair(pair(ε, a), c)).<br>$F1(x)$:=λx.if(flip(1/3), pair(pair(ε, b), c), F2(ε)).<br>$F2(x)$:=λx.insert(Fm0(ε), Fm1(ε)).
$a^nb^mc^{n+m}$	abcd	4d	8	1000 (96)	-3890.9	1	1	$F0(x)$:=λx.if(not(flip(1/3)), F2(ε), pair(pair(ε, a), c)).<br>$F1(x)$:=λx.if(flip(1/3), pair(pair(ε, b), c), F2(ε)).<br>$F2(x)$:=λx.insert(Fm0(ε), Fm1(ε)).
$a^nb^mc^{n+m}$	abcd	4d	8	10000 (201)	-41112.5	1	1	$F0(x)$:=λx.ε.<br>$F1(x)$:=λx.pair(append(head(pair(x, a)), if(not(flip(3/8)), F1(x), if(empty(x), Fm1(pair(ε, b)), ε))), c).<br>$F2(x)$:=λx.F1(F0(ε)).

$a^nb^mc^{n+m}$	abcd	4d	8	100000 (326)	-409857	1	1	F0(x):=λx.pair(if(not(flip(1/3)), append(head(insert(append(pair(append(x, ϵ), b), x), x)), Fm0(x)), if(empty(ϵ), x, if(and(or((ϵ==if(flip(1/2), ϵ, x)), flip(1/2)), flip(1/2)), ϵ, ϵ))), c).<br>F1(x):=λx.F0(append(pair(ϵ, a), x)).<br>F2(x):=λx.F1(F0(pair(if(flip(1/24), ϵ, ϵ), b))).
$a^nb^mc^{n+m}$	abcd	4d	8	1 (1)	-50.9161	1	0.04	F0(x):=λx.pair(pair(ϵ, a), a).<br>F1(x):=λx.pair(pair(Fm0(ϵ), b), c).<br>F2(x):=λx.ϵ.<br>F3(x):=λx.pair(pair(F1(Fm2(ϵ)), c), c).
$a^nb^mc^{n+m}$	abcd	4d	8	10 (8)	-108.729	0.84	1	F0(x):=λx.F2(ϵ).<br>F1(x):=λx.pair(if(flip(1/4), x, F1(pair(x, b))), c).<br>F2(x):=λx.ϵ.<br>F3(x):=λx.append(pair(ϵ, a), if(flip(1/2), pair(Fm3(F0(ϵ)), c), F1(ϵ))).
$a^nb^mc^{n+m}$	abcd	4d	8	100 (40)	-461.906	1	1	F0(x):=λx.Fm1(ϵ).<br>F1(x):=λx.append(pair(ϵ, b), if(flip(1/3), ϵ, pair(F1(ϵ), c))).<br>F2(x):=λx.F0(ϵ).<br>F3(x):=λx.pair(append(pair(ϵ, a), if(flip(1/3), pair(F2(ϵ), c), Fm3(ϵ))), c).
$a^nb^mc^{n+m}$	abcd	4d	8	1000 (96)	-3898.84	1	1	F0(x):=λx.ϵ.<br>F1(x):=λx.pair(append(x, Fm2(x)), c).<br>F2(x):=λx.if(flip(1/3), if((x==pair(ϵ, a)), F1(pair(ϵ, b)), ϵ), F1(x)).<br>F3(x):=λx.F1(pair(F0(ϵ), a)).
$a^nb^mc^{n+m}$	abcd	4d	8	10000 (201)	-41121.1	1	1	F0(x):=λx.ϵ.<br>F1(x):=λx.pair(append(x, F2(x)), c).<br>F2(x):=λx.if(flip(3/8), if((x==pair(ϵ, a)), F1(pair(ϵ, b)), ϵ), F1(x)).<br>F3(x):=λx.F1(pair(F0(ϵ), a)).
$a^nb^mc^{n+m}$	abcd	4d	8	100000 (326)	-410549	1	1	F0(x):=λx.append(if(not(and(empty(x), flip(1/3))), append(head(pair(x, b)), Fm2(x)), pair(ϵ, b)), pair(ϵ, c)).<br>F1(x):=λx.if(flip(1/12), ϵ, ϵ).<br>F2(x):=λx.F0(head(append(if(or((ϵ==x), flip(1/3)), Fm1(ϵ), append(ϵ, pair(ϵ, a))), ϵ))).<br>F3(x):=λx.Fm0(pair(ϵ, a)).
$a^nb^mc^{nm}$	abcd	7d	8	1 (1)	-55.1224	0	0	F0(x):=λx.if(flip(1/6), x, pair(pair(pair(F0(if(flip(1/2), pair(pair(x, b), b), pair(x, a))), c), c), c)).
$a^nb^mc^{nm}$	abcd	7d	8	10 (7)	-124.813	0.26666	0.52	F0(x):=λx.append(if(flip(1/2), x, pair(ϵ, a)), pair(if(not(flip(7/24)), Fm0(pair(ϵ, b)), pair(ϵ, b)), c)).
$a^nb^mc^{nm}$	abcd	7d	8	100 (39)	-1192.54	0.23076	0.44	F0(x):=λx.append(head(pair(x, a)), pair(if(not(flip(if(empty(x), 1/3, 1/2))), pair(F0(x), c), if(flip(if(empty(x), 1/2, 5/24)), F0(pair(x, b)), sample((pair(pair(pair(pair(pair(pair(pair(pair(x, c), c), c), c), c), c), c), c)∪pair(ϵ, b)))))), c)).
$a^nb^mc^{nm}$	abcd	7d	8	1000 (103)	-19605	0.08	0.08	F0(x):=λx.pair(append(head(pair(x, a)), if(not(flip(if(empty(x), 1/8, 1/3))), pair(if(flip(1/24), pair(pair(if(and(or(flip(7/24), empty(ϵ)), empty(x)), sample((Σ\Σ)), pair(pair(pair(pair(x, c), c), c), c)), c), c), F0(if(and(empty(x), flip(7/24)), pair(pair(ϵ, b), b), head(x)))), c), pair(ϵ, b))), c).
$a^nb^mc^{nm}$	abcd	7d	8	10000 (195)	-189632	0.08	0.08	F0(x):=λx.pair(append(head(pair(x, a)), if(not(flip(if(empty(x), 1/8, 1/3))), pair(if(flip(1/24), pair(pair(if(and(or(flip(7/24), empty(ϵ)), empty(x)), sample((Σ\Σ)), pair(pair(pair(pair(x, c), c), c), c)), c), c), F0(if(and(empty(x), flip(7/24)), pair(pair(ϵ, b), b), head(x)))), c), pair(ϵ, b))), c).
$a^nb^mc^{nm}$	abcd	7d	8	100000 (329)	-1.96918e+06	0.08	0.08	F0(x):=λx.pair(append(head(pair(x, a)), if(not(flip(if(empty(x), 1/8, 1/3))), pair(if(flip(1/24), pair(pair(if(and(or(flip(7/24), empty(x)), empty(x)), sample((Σ\Σ)), pair(pair(pair(pair(x, c), c), c), c)), c), c), F0(if(and(empty(x), flip(7/24)), pair(pair(x, b), b), head(x)))), c), pair(ϵ, b))), c).
$a^nb^mc^{nm}$	abcd	7d	8	1 (1)	-57.6557	0.04	0	F0(x):=λx.pair(append(pair(ϵ, a), pair(x, b)), b).<br>F1(x):=λx.pair(if(flip(1/24), F0(Fm0(F0(ϵ))), Fm1(ϵ)), c).
$a^nb^mc^{nm}$	abcd	7d	8	10 (7)	-113.026	0.4	0.72	F0(x):=λx.pair(if(flip(1/2), append(pair(ϵ, a), if(flip(1/2), x, F1(ϵ))), Fm1(x)), c).<br>F1(x):=λx.Fm0(pair(x, b)).
$a^nb^mc^{nm}$	abcd	7d	8	100 (39)	-1188.91	0.32	0.52	F0(x):=λx.append(x, pair(if(not(flip(7/24)), append(F0(if(flip(1/12), pair(ϵ, b), x)), pair(ϵ, c)), pair(pair(if(flip(1/4), pair(ϵ, b), if(not(flip(7/24)), ϵ, append(x, if(flip(1/2), append(if((ϵ==if((ϵ==ϵ), if(flip(1/2), ϵ, x), ϵ)), ϵ, append(x, sample(x))), x), ϵ)))), b), c)), c)).<br>F1(x):=λx.F0(pair(ϵ, a)).
$a^nb^mc^{nm}$	abcd	7d	8	1000 (103)	-20569.5	0.2	0.48	F0(x):=λx.append(pair(ϵ, a), pair(if(flip(1/4), if(or(empty(x), flip(1/12)), pair(append(append(x, if(flip(5/12), ϵ, head(x))), sample((pair(x, b)∪ϵ))), b), pair(x, c)), F0(x)), c)).<br>F1(x):=λx.if(not(flip(7/24)), pair(pair(pair(pair(Fm1(pair(x, b)), c), c), c), c), Fm0(x)).
$a^nb^mc^{nm}$	abcd	7d	8	10000 (195)	-170249	0.32	0.72	F0(x):=λx.append(pair(ϵ, a), if(not((if(not(flip(7/24)), ϵ, x)==x)), pair(F0(pair(pair(pair(x, c), c), c)), c), if(flip(1/8), pair(append(if(empty(ϵ), if(flip(3/8), tail(ϵ), head(if(flip(3/8), x, head(ϵ)))), if(flip(1/2), append(x, if(flip(1/2), ϵ, ϵ)), append(ϵ, ϵ))), sample(ϵ)), b), x))).<br>F1(x):=λx.if(not(flip(if(empty(x), 1/24, 3/8))), pair(Fm1(pair(x, b)), c), F0(x)).
$a^nb^mc^{nm}$	abcd	7d	8	100000 (329)	-1.72545e+06	0.4	0.36	F0(x):=λx.if(flip(1/3), pair(x, b), pair(if((ϵ==if(flip(1/24), append(ϵ, x), pair(x, b))), pair(pair(pair(pair(pair(pair(pair(pair(pair(pair(pair(ϵ, b), b), c), c), c), c), c), c), c), c), c), c), append(append(pair(x, b), Fm0(ϵ)), pair(ϵ, c))), c)).<br>F1(x):=λx.if(flip(1/3), Fm0(pair(ϵ, a)), pair(pair(append(pair(x, a), F1(ϵ)), c), c)).
$a^nb^mc^{nm}$	abcd	7d	8	1 (1)	-66.6739	0.15384	0.12	F0(x):=λx.append(if(flip(1/2), pair(ϵ, a), F1(ϵ)), pair(F2(pair(x, b)), c)).<br>F1(x):=λx.ϵ.<br>F2(x):=λx.if(flip(1/6), x, pair(pair(F0(x), c), c)).
$a^nb^mc^{nm}$	abcd	7d	8	10 (7)	-129.089	0.22580	0.6	F0(x):=λx.if(flip(1/2), x, if(and(empty(x), flip(1/2)), pair(ϵ, c), F2(x))).<br>F1(x):=λx.if(flip(1/2), x, pair(ϵ, a)).<br>F2(x):=λx.append(append(Fm1(x), Fm0(pair(ϵ, b))), pair(Fm0(ϵ), c)).

$a^nb^mc^{nm}$	abcd	7d	8	100 (39)	-1053.33	0.44	0.64	$F0(x)$:=λx.if(flip(1/2), append(Fm0(ε), sample(if(empty(x), Fm0(ε), pair(append(append(F0(x), pair(x, c)), x), c)))), pair(ε, b)).<br>$F1(x)$:=λx.append(pair(ε, a), if(flip(7/24), pair(x, c), Fm1(pair(x, c)))).<br>$F2(x)$:=λx.F1(F0(if(flip(1/24), ε, pair(if(flip(1/4), ε, pair(ε, c)), c)))).
$a^nb^mc^{nm}$	abcd	7d	8	1000 (103)	-17212.8	0.41379	0.48	$F0(x)$:=λx.F1(append(x, if(flip(1/8), if((ε==if(flip(1/4), if(flip(1/2), x, x), ε)), if(or((x==head(x)), flip(5/24)), pair(if(flip(1/4), append(pair(ε, a), x), x), a), pair(ε, b)), append(x, if(flip(1/2), x, pair(x, a)))), append(pair(ε, a), pair(ε, b))))).<br>$F1(x)$:=λx.pair(append(pair(x, b), if(flip(1/3), ε, pair(Fm1(ε), c))), c).<br>$F2(x)$:=λx.if(not(flip(1/3)), pair(pair(Fm2(pair(x, a)), c), c), c), F0(x)).
$a^nb^mc^{nm}$	abcd	7d	8	10000 (195)	-154776	0.24	0.6	$F0(x)$:=λx.append(pair(ε, a), if(flip(5/24), append(if(flip(5/12), ε, ε), append(pair(ε, a), x)), Fm2(if(flip(1/8), x, pair(pair(F1(pair(pair(pair(pair(Fm1(Fm1(pair(append(head(x), x), c))), c), c), c), c)), c), c)))))).<br>$F1(x)$:=λx.if(empty(x), F0(Fm1(pair(pair(pair(pair(ε, b), b), c), c))), pair(pair(x, c), c)).<br>$F2(x)$:=λx.if(not(flip(5/24)), append(head(pair(sample(insert(sample(pair(ε, a)), if(empty(tail(x)), x, ε))), b)), pair(Fm2(append(ε, x)), c)), if(flip(1/8), pair(head(ε), b), pair(F1(x), c))).
$a^nb^mc^{nm}$	abcd	7d	8	100000 (329)	-1.60699e+06	0.24	0.6	$F0(x)$:=λx.append(pair(ε, a), if(flip(5/24), append(if(flip(5/12), ε, ε), append(pair(ε, a), x)), Fm2(if(flip(1/8), x, pair(pair(F1(pair(pair(pair(pair(Fm1(Fm1(pair(append(pair(ε, b), x), c))), c), c), c), c)), c), c)))))).<br>$F1(x)$:=λx.if(empty(x), F0(Fm1(pair(pair(pair(pair(ε, b), b), c), c))), pair(pair(x, c), c)).<br>$F2(x)$:=λx.if(not(flip(5/24)), append(head(insert(append(x, pair(ε, a)), ε)), pair(Fm2(append(append(sample(tail(ε)), ε), x)), c)), if(flip(if(empty(ε), 1/8, 1/2)), pair(ε, b), pair(F1(x), c))).
$a^nb^mc^{nm}$	abcd	7d	8	1 (1)	-74.3061	0.04	0	$F0(x)$:=λx.insert(append(pair(Fm2(ε), a), x), x).<br>$F1(x)$:=λx.Fm0(pair(x, b)).<br>$F2(x)$:=λx.ε.<br>$F3(x)$:=λx.pair(if(not(flip(1/24)), F3(ε), F1(Fm1(ε))), c).
$a^nb^mc^{nm}$	abcd	7d	8	10 (7)	-132.55	0.57692	0.56	$F0(x)$:=λx.if(flip(1/12), pair(F0(if(flip(1/4), F1(ε), pair(pair(ε, a), a))), a), x).<br>$F1(x)$:=λx.ε.<br>$F2(x)$:=λx.if(not(flip(1/4)), Fm3(F0(x)), append(pair(ε, a), x)).<br>$F3(x)$:=λx.pair(Fm2(pair(x, b)), c).
$a^nb^mc^{nm}$	abcd	7d	8	100 (39)	-1283.64	0.4	0.56	$F0(x)$:=λx.pair(Fm2(x), c).<br>$F1(x)$:=λx.append(append(pair(append(pair(x, c), pair(pair(append(x, x), c), c)), c), pair(x, c)), append(pair(x, c), x)).<br>$F2(x)$:=λx.sample(if(not(flip(5/24)), append(x, if((append(ε, ε)==ε), F0(if(flip(1/24), pair(ε, b), x)), x)), if(flip(1/2), pair(x, b), pair(pair(pair(if(flip(1/3), pair(pair(append(pair(pair(x, b), b), Fm1(pair(head(ε), c))), c), c), pair(ε, c)), c), c), c)))).<br>$F3(x)$:=λx.pair(Fm2(pair(ε, a)), c).
$a^nb^mc^{nm}$	abcd	7d	8	1000 (103)	-19067.8	0.36	0.52	$F0(x)$:=λx.append(pair(ε, a), F2(ε)).<br>$F1(x)$:=λx.if(flip(7/24), append(x, if(not(and(flip(1/12), or(flip(11/24), empty(ε)))), ε, pair(if(flip(5/24), if(flip(3/8), x, ε), pair(x, b)), b))), pair(append(x, pair(pair(pair(Fm1(x), c), c), c)), c)).<br>$F2(x)$:=λx.pair(if(not(flip(1/3)), Fm3(ε), F1(pair(ε, b))), c).<br>$F3(x)$:=λx.F0(ε).
$a^nb^mc^{nm}$	abcd	7d	8	10000 (195)	-161202	0.2	0.6	$F0(x)$:=λx.if(flip(1/3), append(x, x), if(flip(1/2), x, ε)).<br>$F1(x)$:=λx.if(flip(if((append(x, if((head(x)==x), ε, Fm2(ε)))==x), if(empty(x), 3/8, 1/2), 1/24)), if((head(Fm2(x))==x), Fm0(x), pair(ε, b)), pair(F1(ε), b)).<br>$F2(x)$:=λx.if(not(flip(1/4)), pair(pair(pair(pair(x, c), c), c), c), if(flip(7/24), pair(x, c), ε)).<br>$F3(x)$:=λx.append(append(pair(ε, a), if(flip(if(empty(x), 11/24, 1/4)), pair(if(flip(1/24), pair(x, a), if(not(flip(1/12)), F1(pair(x, a)), x)), b), F3(pair(x, a)))), pair(pair(Fm2(ε), c), c)).
$a^nb^mc^{nm}$	abcd	7d	8	100000 (329)	-1.39981e+06	0.28	0.32	$F0(x)$:=λx.if(or((x==ε), not(flip(7/24))), pair(insert(if((head(x)==x), ε, pair(ε, c)), pair(F2(x), c)), c), insert(append(pair(ε, b), x), pair(ε, b))).<br>$F1(x)$:=λx.Fm3(x).<br>$F2(x)$:=λx.append(pair(ε, a), sample(if(empty(x), if(flip(1/3), pair(if(flip(1/12), pair(pair(pair(pair(x, a), a), a), a), if(flip(if(empty(sample(Σ)), 1/24, 5/24)), x, if(flip(1/4), pair(if(or(flip(7/24), flip(11/24)), x, pair(ε, a)), a), pair(x, b)))), b), F0(ε)), pair(Fm0(x), c)))).<br>$F3(x)$:=λx.pair(if(flip(1/3), pair(F2(x), c), Fm1(pair(x, b))), c).
a^nb^{n+m}	abcd	1h	8	1 (1)	-23.8158	0.95238	0.24	$F0(x)$:=λx.pair(if(flip(1/2), pair(ε, a), F0(ε)), b).
a^nb^{n+m}	abcd	1h	8	10 (9)	-99.7281	1	0.84	$F0(x)$:=λx.pair(if(flip(1/2), if(empty(x), F0(ε), if(flip(1/2), x, ε)), append(pair(x, a), pair(Fm0(pair(ε, a)), b))), b).
a^nb^{n+m}	abcd	1h	8	100 (39)	-418.657	1	1	$F0(x)$:=λx.if(flip(1/2), pair(F0(pair(x, a)), b), if(empty(x), pair(Fm0(ε), b), pair(x, b))).
a^nb^{n+m}	abcd	1h	8	1000 (96)	-3926.87	1	1	$F0(x)$:=λx.pair(append(head(pair(x, a)), if(not(flip(3/8)), F0(x), if(not(and(empty(x), flip(11/24))), pair(if(flip(3/8), pair(ε, b), if(flip(1/2), ε, ε)), b), Fm0(pair(ε, b))))), b).
a^nb^{n+m}	abcd	1h	8	10000 (187)	-39194.1	1	1	$F0(x)$:=λx.pair(append(head(pair(head(x), a)), if(not(flip(3/8)), F0(append(x, x)), if(not(and(empty(append(x, x)), flip(1/2))), pair(if(flip(5/12), pair(ε, b), ε), b), Fm0(pair(ε, b))))), b).
a^nb^{n+m}	abcd	1h	8	100000 (316)	-396647	1	1	$F0(x)$:=λx.pair(append(head(pair(head(append(ε, x)), a)), if(not(flip(3/8)), F0(append(x, x)), if(not(and(empty(append(x, x)), flip(3/8))), pair(if(flip(5/12), pair(ε, b), ε), b), Fm0(pair(x, b))))), b).
a^nb^{n+m}	abcd	1h	8	1 (1)	-28.0464	1	0.04	$F0(x)$:=λx.pair(pair(x, b), b).<br>$F1(x)$:=λx.F0(Fm0(pair(ε, a))).
a^nb^{n+m}	abcd	1h	8	10 (9)	-135.301	0.68	0.28	$F0(x)$:=λx.pair(if(flip(5/24), x, F0(ε)), a).<br>$F1(x)$:=λx.pair(if(not(flip(1/3)), Fm1(pair(x, b)), append(pair(Fm0(ε), b), append(pair(x, b), x))), b).
a^nb^{n+m}	abcd	1h	8	100 (39)	-412.198	1	1	$F0(x)$:=λx.pair(if(flip(3/8), if(not(empty(x)), F0(ε), pair(ε, a)), append(x, Fm0(x))), b).<br>$F1(x)$:=λx.F0(pair(ε, a)).
a^nb^{n+m}	abcd	1h	8	1000 (96)	-3847.21	1	1	$F0(x)$:=λx.pair(if(flip(1/3), if(not(empty(x)), x, pair(ε, a)), append(head(x), F0(x))), b).<br>$F1(x)$:=λx.F0(Fm0(ε)).

a^nb^{n+m}	abcd	1h	8	10000 (187)	-38296.1	1	1	F0(x):=λx.pair(if(flip(1/3), if(not(empty(x)), Fm0(ϵ), pair(ϵ, a)), append(x, Fm0(x))), b).<br>F1(x):=λx.F0(pair(ϵ, a)).
a^nb^{n+m}	abcd	1h	8	100000 (316)	-387363	1	1	F0(x):=λx.pair(if(flip(1/3), if(not(empty(x)), Fm0(ϵ), pair(ϵ, a)), append(x, Fm0(x))), b).<br>F1(x):=λx.F0(pair(ϵ, a)).
a^nb^{n+m}	abcd	1h	8	1 (1)	-37.0868	0.25	0.04	F0(x):=λx.pair(pair(x, b), b).<br>F1(x):=λx.Fm0(x).<br>F2(x):=λx.F0(F1(sample(Σ))).
a^nb^{n+m}	abcd	1h	8	10 (9)	-119.225	1	0.72	F0(x):=λx.Fm2(x).<br>F1(x):=λx.pair(x, b).<br>F2(x):=λx.if(flip(5/12), pair(if(not(empty(x)), F1(x), F0(ϵ)), b), pair(F0(pair(x, a)), b)).
a^nb^{n+m}	abcd	1h	8	100 (39)	-433.262	1	1	F0(x):=λx.Fm2(ϵ).<br>F1(x):=λx.x.<br>F2(x):=λx.if(flip(11/24), pair(if(not(empty(x)), x, F0(ϵ)), b), pair(F2(pair(Fm1(x), a)), b)).
a^nb^{n+m}	abcd	1h	8	1000 (96)	-3864.3	1	1	F0(x):=λx.append(x, pair(if(flip(if(empty(x), 1/3, 1/8)), pair(ϵ, b), F1(x)), b)).<br>F1(x):=λx.Fm0(if(or((x==ϵ), flip(1/4)), ϵ, x)).<br>F2(x):=λx.F0(pair(ϵ, a)).
a^nb^{n+m}	abcd	1h	8	10000 (187)	-38323.7	1	1	F0(x):=λx.append(x, pair(if(flip(if(empty(x), 1/3, 1/8)), pair(ϵ, b), F1(x)), b)).<br>F1(x):=λx.Fm0(if(or(empty(x), flip(1/4)), ϵ, x)).<br>F2(x):=λx.F0(pair(x, a)).
a^nb^{n+m}	abcd	1h	8	100000 (316)	-386216	1	1	F0(x):=λx.append(x, pair(if(flip(if((x==ϵ), 1/3, 1/8)), pair(ϵ, b), F1(x)), b)).<br>F1(x):=λx.Fm0(if(or(empty(x), flip(5/24)), ϵ, x)).<br>F2(x):=λx.F0(pair(ϵ, a)).
a^nb^{n+m}	abcd	1h	8	1 (1)	-45.4546	1	0.04	F0(x):=λx.pair(x, b).<br>F1(x):=λx.pair(F2(x), b).<br>F2(x):=λx.F0(x).<br>F3(x):=λx.Fm1(F1(pair(ϵ, a))).
a^nb^{n+m}	abcd	1h	8	10 (9)	-117.647	1	1	F0(x):=λx.pair(if(flip(1/3), ϵ, F0(ϵ)), b).<br>F1(x):=λx.ϵ.<br>F2(x):=λx.F3(F1(ϵ)).<br>F3(x):=λx.append(pair(ϵ, a), if(flip(5/24), pair(F0(ϵ), b), pair(F2(ϵ), b))).
a^nb^{n+m}	abcd	1h	8	100 (39)	-427.096	1	1	F0(x):=λx.pair(if(flip(5/12), ϵ, F2(Fm1(ϵ))), b).<br>F1(x):=λx.ϵ.<br>F2(x):=λx.F0(ϵ).<br>F3(x):=λx.append(pair(ϵ, a), if(flip(1/3), pair(F2(ϵ), b), pair(F3(ϵ), b))).
a^nb^{n+m}	abcd	1h	8	1000 (96)	-3862.29	1	1	F0(x):=λx.pair(ϵ, a).<br>F1(x):=λx.pair(if(not(flip(1/3)), Fm1(Fm2(ϵ)), ϵ), b).<br>F2(x):=λx.ϵ.<br>F3(x):=λx.append(Fm0(ϵ), pair(if(flip(1/3), F1(ϵ), Fm3(ϵ)), b)).
a^nb^{n+m}	abcd	1h	8	10000 (187)	-38304.9	1	1	F0(x):=λx.pair(ϵ, a).<br>F1(x):=λx.pair(if(not(flip(1/3)), Fm1(ϵ), ϵ), b).<br>F2(x):=λx.ϵ.<br>F3(x):=λx.append(Fm0(ϵ), pair(if(flip(1/3), F1(ϵ), Fm3(Fm2(ϵ))), b)).
a^nb^{n+m}	abcd	1h	8	100000 (316)	-385634	1	1	F0(x):=λx.pair(ϵ, a).<br>F1(x):=λx.pair(if(not(flip(3/8)), Fm1(Fm2(ϵ)), x), b).<br>F2(x):=λx.ϵ.<br>F3(x):=λx.append(Fm0(x), pair(if(flip(1/3), Fm1(ϵ), Fm3(ϵ)), b)).
$a^nb^mc^ma^n$	abcd	2d	8	1 (1)	-55.8458	0.04761	0.04	F0(x):=λx.if(flip(1/2), pair(pair(pair(pair(pair(x, b), c), c), c), c), pair(F0(pair(append(pair(ϵ, a), x), b)), a)).
$a^nb^mc^ma^n$	abcd	2d	8	10 (9)	-199.325	0.36363	0.88	F0(x):=λx.if(flip(1/2), pair(append(pair(ϵ, a), F0(ϵ)), a), pair(append(pair(ϵ, b), if(flip(1/2), ϵ, Fm0(ϵ))), c)).
$a^nb^mc^ma^n$	abcd	2d	8	100 (40)	-2195.47	0.46153	0.8	F0(x):=λx.pair(if(flip(1/3), append(if(flip(3/8), insert(pair(ϵ, a), x)), pair(x, a)), insert(sample((pair(ϵ, c)∪pair(pair(pair(sample((ϵ∪pair(ϵ, b))), b), c), c))), sample((pair(pair(pair(pair(ϵ, b), b), c), c)∪pair(ϵ, b)))))), Fm0(pair(x, a))), a).
$a^nb^mc^ma^n$	abcd	2d	8	1000 (101)	-4486.74	0.88	1	F0(x):=λx.insert(sample(if(empty(x), append(pair(ϵ, a), pair(ϵ, a)), pair(x, c))), if(flip(if(empty(x), 1/24, 5/12)), ϵ, Fm0(sample((x∪pair(ϵ, b)))))).
$a^nb^mc^ma^n$	abcd	2d	8	10000 (190)	-43916.2	0.88	1	F0(x):=λx.insert(sample(if(empty(x), append(pair(ϵ, a), pair(ϵ, a)), pair(x, c))), if(flip(if(empty(x), 1/24, 5/12)), ϵ, Fm0(sample((x∪pair(ϵ, b)))))).
$a^nb^mc^ma^n$	abcd	2d	8	100000 (332)	-453180	0.88	1	F0(x):=λx.insert(sample(if(empty(x), append(pair(ϵ, a), pair(ϵ, a)), pair(x, c))), if(flip(if((ϵ==x), 1/24, 5/12)), ϵ, Fm0(sample((x∪pair(ϵ, b)))))).
$a^nb^mc^ma^n$	abcd	2d	8	1 (1)	-56.822	0.01176	0.08	F0(x):=λx.append(if(flip(1/2), Fm0(sample(Σ)), x), insert(x, x)).<br>F1(x):=λx.pair(F0(pair(pair(ϵ, c), a)), a).
$a^nb^mc^ma^n$	abcd	2d	8	10 (9)	-95.716	1	1	F0(x):=λx.append(head(pair(x, a)), if(flip(1/2), pair(F0(pair(ϵ, b)), c), if(empty(x), F1(ϵ), ϵ))).<br>F1(x):=λx.pair(F0(ϵ), a).
$a^nb^mc^ma^n$	abcd	2d	8	100 (40)	-472.515	1	1	F0(x):=λx.append(head(pair(x, a)), if(flip(1/2), pair(F0(pair(ϵ, b)), c), if(empty(x), F1(ϵ), ϵ))).<br>F1(x):=λx.pair(F0(ϵ), a).
$a^nb^mc^ma^n$	abcd	2d	8	1000 (101)	-4493.4	1	1	F0(x):=λx.append(head(pair(x, a)), if(flip(5/12), pair(F0(pair(ϵ, b)), c), if(empty(x), F1(ϵ), ϵ))).<br>F1(x):=λx.pair(F0(ϵ), a).

$a^nb^mc^ma^n$	abcd	2d	8	10000 (190)	-46681.7	1	1	F0(x):=λx.append(head(pair(x, a)), if(flip(1/2), pair(F0(pair(ϵ, b)), c), if(empty(x), F1(ϵ), ϵ))).<br>F1(x):=λx.pair(F0(ϵ), a).
$a^nb^mc^ma^n$	abcd	2d	8	100000 (332)	-419735	1	1	F0(x):=λx.pair(if(flip(1/3), pair(x, b), Fm0(pair(x, b))), c).<br>F1(x):=λx.if(flip(3/8), append(F0(pair(ϵ, a)), pair(ϵ, a)), append(pair(ϵ, a), pair(F1(ϵ), a))).
$a^nb^mc^ma^n$	abcd	2d	8	1 (1)	-62.6651	0	0	F0(x):=λx.append(if(flip(1/2), x, append(append(Fm1(ϵ), x), x)), append(x, x)).<br>F1(x):=λx.Fm0(sample(Σ)).<br>F2(x):=λx.F1(ϵ).
$a^nb^mc^ma^n$	abcd	2d	8	10 (9)	-104.199	0.48	1	F0(x):=λx.pair(pair(ϵ, b), c).<br>F1(x):=λx.pair(if(flip(1/6), ϵ, Fm1(ϵ)), a).<br>F2(x):=λx.insert(if(flip(1/2), F1(ϵ), F2(ϵ)), Fm0(ϵ)).
$a^nb^mc^ma^n$	abcd	2d	8	100 (40)	-459.142	1	1	F0(x):=λx.append(pair(ϵ, a), if(flip(1/3), x, Fm0(pair(x, a)))).<br>F1(x):=λx.insert(if(not(flip(1/3)), Fm1(ϵ), pair(ϵ, a)), pair(pair(ϵ, b), c)).<br>F2(x):=λx.F0(Fm1(ϵ)).
$a^nb^mc^ma^n$	abcd	2d	8	1000 (101)	-3900.82	1	1	F0(x):=λx.if(or(empty(x), not(flip(7/24))), append(pair(ϵ, a), pair(Fm0(pair(ϵ, c)), a)), F1(x)).<br>F1(x):=λx.append(pair(ϵ, b), if(or(flip(3/8), flip(1/24)), x, pair(F1(x), c))).<br>F2(x):=λx.Fm0(ϵ).
$a^nb^mc^ma^n$	abcd	2d	8	10000 (190)	-40033	1	1	F0(x):=λx.if(flip(1/3), x, F1(x)).<br>F1(x):=λx.pair(F0(pair(x, b)), c).<br>F2(x):=λx.pair(if(flip(1/3), F1(pair(ϵ, a)), append(pair(ϵ, a), F2(ϵ))), a).
$a^nb^mc^ma^n$	abcd	2d	8	100000 (332)	-420174	1	1	F0(x):=λx.if(flip(1/3), x, F1(x)).<br>F1(x):=λx.pair(F0(pair(x, b)), c).<br>F2(x):=λx.pair(if(flip(1/3), F1(pair(ϵ, a)), append(pair(ϵ, a), F2(ϵ))), a).
$a^nb^mc^ma^n$	abcd	2d	8	1 (1)	-69.6466	1	0.2	F0(x):=λx.append(pair(x, b), append(pair(if(flip(1/2), Fm0(Fm2(ϵ)), ϵ), c), x)).<br>F1(x):=λx.pair(pair(ϵ, a), a).<br>F2(x):=λx.ϵ.<br>F3(x):=λx.F0(pair(F1(ϵ), a)).
$a^nb^mc^ma^n$	abcd	2d	8	10 (9)	-109.867	0.96551	1	F0(x):=λx.pair(ϵ, b).<br>F1(x):=λx.if(flip(1/2), insert(Fm1(pair(pair(ϵ, a), a)), F3(ϵ)), x).<br>F2(x):=λx.F1(pair(Fm0(ϵ), c)).<br>F3(x):=λx.Fm2(ϵ).
$a^nb^mc^ma^n$	abcd	2d	8	100 (40)	-467.288	1	1	F0(x):=λx.Fm2(pair(x, b)).<br>F1(x):=λx.pair(F0(pair(x, a)), c).<br>F2(x):=λx.if(flip(1/3), x, pair(F0(x), c)).<br>F3(x):=λx.if(flip(1/3), pair(F1(x), a), pair(F3(pair(x, a)), a)).
$a^nb^mc^ma^n$	abcd	2d	8	1000 (101)	-4464.98	1	1	F0(x):=λx.insert(if(flip(1/3), Fm1(pair(ϵ, a)), Fm3(ϵ)), x).<br>F1(x):=λx.if(flip(3/8), F2(append(x, x)), F1(pair(x, a))).<br>F2(x):=λx.x.<br>F3(x):=λx.F0(pair(pair(ϵ, b), c)).
$a^nb^mc^ma^n$	abcd	2d	8	10000 (190)	-40683.6	1	1	F0(x):=λx.insert(if(flip(1/3), Fm1(pair(ϵ, a)), Fm3(ϵ)), x).<br>F1(x):=λx.if(flip(3/8), F2(append(x, x)), F1(pair(x, a))).<br>F2(x):=λx.x.<br>F3(x):=λx.F0(pair(pair(ϵ, b), c)).
$a^nb^mc^ma^n$	abcd	2d	8	100000 (332)	-417990	1	1	F0(x):=λx.insert(sample(if(not(flip(1/3)), Fm0(x), if(empty(sample(ϵ)), append(x, x), sample((if(flip(1/2), ϵ, ϵ)\Σ))))), pair(pair(ϵ, b), c)).<br>F1(x):=λx.pair(F3(tail(ϵ)), a).<br>F2(x):=λx.if(flip(1/3), F0(x), append(insert(ϵ, x), Fm1(sample(((tail(x)\ϵ)∪(sample((head(ϵ)∪ϵ))∪ϵ)))))).<br>F3(x):=λx.Fm2(insert(pair(sample(ϵ), a), ϵ)).
$a^nb^{2n}c^{3n}$	abcd	5d	8	1 (1)	-41.3517	0.01162	0.04	F0(x):=λx.pair(if(flip(1/2), pair(ϵ, c), append(append(x, x), Fm0(sample(Σ)))), c).
$a^nb^{2n}c^{3n}$	abcd	5d	8	10 (6)	-72.217	1	0.84	F0(x):=λx.pair(append(pair(ϵ, a), pair(insert(pair(if(flip(1/4), ϵ, F0(ϵ)), c), pair(pair(ϵ, b), b)), c)), c).
$a^nb^{2n}c^{3n}$	abcd	5d	8	100 (12)	-245.205	1	0.84	F0(x):=λx.append(pair(ϵ, a), pair(pair(insert(pair(if(flip(1/3), ϵ, F0(ϵ)), c), pair(pair(ϵ, b), b)), c), c)).
$a^nb^{2n}c^{3n}$	abcd	5d	8	1000 (16)	-2918.29	1	0.84	F0(x):=λx.append(pair(ϵ, a), pair(pair(insert(pair(if(flip(1/3), ϵ, F0(ϵ)), c), pair(pair(ϵ, b), b)), c), c)).
$a^nb^{2n}c^{3n}$	abcd	5d	8	10000 (22)	-21113.2	1	0.84	F0(x):=λx.append(pair(ϵ, a), pair(pair(insert(pair(if(flip(1/3), ϵ, F0(ϵ)), c), pair(pair(ϵ, b), b)), c), c)).
$a^nb^{2n}c^{3n}$	abcd	5d	8	100000 (27)	-209565	1	0.84	F0(x):=λx.append(pair(ϵ, a), pair(pair(insert(pair(if(flip(1/3), ϵ, F0(ϵ)), c), pair(pair(ϵ, b), b)), c), c)).
$a^nb^{2n}c^{3n}$	abcd	5d	8	1 (1)	-44.899	0	0.04	F0(x):=λx.append(x, x).<br>F1(x):=λx.append(Fm0(sample(Σ)), pair(if(flip(1/2), Fm1(ϵ), ϵ), c)).
$a^nb^{2n}c^{3n}$	abcd	5d	8	10 (6)	-76.1527	1	0.84	F0(x):=λx.append(pair(ϵ, a), pair(pair(if(flip(1/4), x, F1(x)), c), c)).<br>F1(x):=λx.pair(Fm0(pair(pair(x, b), b)), c).
$a^nb^{2n}c^{3n}$	abcd	5d	8	100 (12)	-249.141	1	0.84	F0(x):=λx.append(pair(ϵ, a), pair(pair(if(flip(1/3), x, F1(x)), c), c)).<br>F1(x):=λx.pair(Fm0(pair(pair(x, b), b)), c).
$a^nb^{2n}c^{3n}$	abcd	5d	8	1000 (16)	-2922.23	1	0.84	F0(x):=λx.append(pair(ϵ, a), pair(pair(if(flip(1/3), x, F1(x)), c), c)).<br>F1(x):=λx.pair(Fm0(pair(pair(x, b), b)), c).
$a^nb^{2n}c^{3n}$	abcd	5d	8	10000 (22)	-21117.1	1	0.84	F0(x):=λx.append(pair(ϵ, a), pair(pair(if(flip(1/3), x, F1(x)), c), c)).<br>F1(x):=λx.pair(Fm0(pair(pair(x, b), b)), c).

$a^nb^{2n}c^{3n}$	abcd	5d	8	100000 (27)	-209569	1	0.84	$F0(x)$:=λx.append(pair(ε, a), pair(if(flip(1/3), append(x, x), Fm1(x)), c)).<br>$F1(x)$:=λx.pair(pair(F0(pair(x, b)), c), c).
$a^nb^{2n}c^{3n}$	abcd	5d	8	1 (1)	-51.0509	1	0.04	$F0(x)$:=λx.F1(pair(x, c)).<br>$F1(x)$:=λx.insert(x, x).<br>$F2(x)$:=λx.append(pair(pair(ε, a), a), F0(F0(pair(ε, b)))).
$a^nb^{2n}c^{3n}$	abcd	5d	8	10 (6)	-84.7685	1	0.84	$F0(x)$:=λx.append(pair(ε, a), pair(pair(pair(x, c), c), c)).<br>$F1(x)$:=λx.F0(if(flip(1/4), append(x, x), Fm2(x))).<br>$F2(x)$:=λx.Fm1(pair(x, b)).
$a^nb^{2n}c^{3n}$	abcd	5d	8	100 (12)	-257.757	1	0.84	$F0(x)$:=λx.append(pair(ε, a), pair(pair(pair(x, c), c), c)).<br>$F1(x)$:=λx.F0(if(flip(1/3), append(x, x), Fm2(x))).<br>$F2(x)$:=λx.Fm1(pair(x, b)).
$a^nb^{2n}c^{3n}$	abcd	5d	8	1000 (16)	-2506.85	0.76	0.84	$F0(x)$:=λx.append(x, if(flip(1/24), ε, F1(ε))).<br>$F1(x)$:=λx.pair(pair(F0(pair(ε, a)), b), b).<br>$F2(x)$:=λx.if(not(flip(1/3)), append(pair(ε, a), pair(pair(append(F2(pair(pair(x, b), b)), pair(ε, c)), c), c)), if(not(empty(x)), x, if(not(flip(1/24)), Fm2(ε), pair(Fm2(F1(ε)), c)))).
$a^nb^{2n}c^{3n}$	abcd	5d	8	10000 (22)	-21136	1	0.84	$F0(x)$:=λx.pair(pair(if(not(flip(1/3)), pair(F2(x), c), pair(x, c)), c), c).<br>$F1(x)$:=λx.pair(ε, a).<br>$F2(x)$:=λx.Fm0(append(F1(ε), append(pair(x, b), pair(ε, b)))).
$a^nb^{2n}c^{3n}$	abcd	5d	8	100000 (27)	-209578	1	0.84	$F0(x)$:=λx.pair(x, c).<br>$F1(x)$:=λx.append(pair(ε, a), if(flip(1/3), x, F2(x))).<br>$F2(x)$:=λx.Fm0(pair(pair(F1(pair(pair(x, b), b)), c), c)).
$a^nb^{2n}c^{3n}$	abcd	5d	8	1 (1)	-60.9209	0	0.04	$F0(x)$:=λx.Fm1(sample(Σ)).<br>$F1(x)$:=λx.append(append(x, x), Fm2(ε)).<br>$F2(x)$:=λx.pair(if(flip(1/2), Fm0(ε), ε), c).<br>$F3(x)$:=λx.F0(ε).
$a^nb^{2n}c^{3n}$	abcd	5d	8	10 (6)	-93.5609	1	0.64	$F0(x)$:=λx.if(flip(1/4), x, F3(x)).<br>$F1(x)$:=λx.F0(pair(x, b)).<br>$F2(x)$:=λx.append(pair(ε, a), pair(pair(x, c), c)).<br>$F3(x)$:=λx.Fm2(pair(F1(pair(x, b)), c)).
$a^nb^{2n}c^{3n}$	abcd	5d	8	100 (12)	-265.244	1	0.64	$F0(x)$:=λx.pair(ε, a).<br>$F1(x)$:=λx.if(flip(1/3), append(x, x), F3(x)).<br>$F2(x)$:=λx.Fm0(ε).<br>$F3(x)$:=λx.pair(append(F2(ε), pair(pair(Fm1(pair(x, b)), c), c)), c).
$a^nb^{2n}c^{3n}$	abcd	5d	8	1000 (16)	-2900.68	0.8	0.84	$F0(x)$:=λx.insert(pair(append(Fm2(ε), if(flip(1/3), ε, Fm3(ε))), c), pair(pair(pair(x, b), b), c)).<br>$F1(x)$:=λx.ε.<br>$F2(x)$:=λx.pair(if(not(and(flip(1/24), flip(1/24))), Fm1(ε), pair(ε, a)), a).<br>$F3(x)$:=λx.pair(F0(ε), c).
$a^nb^{2n}c^{3n}$	abcd	5d	8	10000 (22)	-21133.2	1	0.84	$F0(x)$:=λx.F1(ε).<br>$F1(x)$:=λx.pair(ε, a).<br>$F2(x)$:=λx.if(flip(1/3), append(x, x), Fm3(x)).<br>$F3(x)$:=λx.pair(append(Fm0(ε), pair(pair(Fm2(pair(x, b)), c), c)), c).
$a^nb^{2n}c^{3n}$	abcd	5d	8	100000 (27)	-209585	1	0.84	$F0(x)$:=λx.F1(ε).<br>$F1(x)$:=λx.pair(ε, a).<br>$F2(x)$:=λx.if(flip(1/3), append(x, x), Fm3(x)).<br>$F3(x)$:=λx.pair(append(Fm0(ε), pair(pair(Fm2(pair(x, b)), c), c)), c).
$a^nb^{n+1}c^{n+2}$	abcd	1d	8	1 (1)	-28.9627	1	0.04	$F0(x)$:=λx.pair(pair(pair(pair(pair(pair(ε, a), b), b), c), c), c).
$a^nb^{n+1}c^{n+2}$	abcd	1d	8	10 (7)	-67.2422	0.92	1	$F0(x)$:=λx.pair(if(flip(1/6), x, Fm0(pair(if(empty(x), ε, append(pair(ε, a), x)), b))), c).
$a^nb^{n+1}c^{n+2}$	abcd	1d	8	100 (10)	-241.944	1	1	$F0(x)$:=λx.pair(append(pair(ε, a), if(flip(1/3), pair(pair(pair(pair(x, b), b), c), c), Fm0(pair(x, b)))), c).
$a^nb^{n+1}c^{n+2}$	abcd	1d	8	1000 (16)	-2024.82	1	1	$F0(x)$:=λx.pair(append(pair(ε, a), if(flip(1/3), pair(pair(pair(pair(x, b), b), c), c), Fm0(pair(x, b)))), c).
$a^nb^{n+1}c^{n+2}$	abcd	1d	8	10000 (20)	-19409.2	1	1	$F0(x)$:=λx.pair(append(pair(ε, a), if(flip(1/3), pair(pair(pair(pair(x, b), b), c), c), Fm0(pair(x, b)))), c).
$a^nb^{n+1}c^{n+2}$	abcd	1d	8	100000 (26)	-193030	1	1	$F0(x)$:=λx.pair(append(pair(ε, a), if(flip(1/3), pair(pair(pair(pair(x, b), b), c), c), Fm0(pair(x, b)))), c).
$a^nb^{n+1}c^{n+2}$	abcd	1d	8	1 (1)	-35.482	0.0625	0.04	$F0(x)$:=λx.append(sample(Σ), insert(pair(x, c), x)).<br>$F1(x)$:=λx.Fm0(Fm0(ε)).
$a^nb^{n+1}c^{n+2}$	abcd	1d	8	10 (7)	-78.1221	0.96	1	$F0(x)$:=λx.ε.<br>$F1(x)$:=λx.pair(append(x, if(flip(5/24), pair(pair(ε, b), c), insert(Fm1(pair(ε, a)), pair(Fm0(ε), b)))), c).
$a^nb^{n+1}c^{n+2}$	abcd	1d	8	100 (10)	-250.232	1	1	$F0(x)$:=λx.ε.<br>$F1(x)$:=λx.append(x, pair(insert(if(and(not(empty(x)), flip(1/3)), pair(F0(ε), c), F1(pair(ε, a))), pair(ε, b)), c)).
$a^nb^{n+1}c^{n+2}$	abcd	1d	8	1000 (16)	-2033.11	1	1	$F0(x)$:=λx.ε.<br>$F1(x)$:=λx.append(x, pair(insert(if(and(not(empty(x)), flip(1/3)), pair(F0(ε), c), F1(pair(ε, a))), pair(ε, b)), c)).
$a^nb^{n+1}c^{n+2}$	abcd	1d	8	10000 (20)	-19417.5	1	1	$F0(x)$:=λx.ε.<br>$F1(x)$:=λx.append(x, pair(insert(if(and(not(empty(x)), flip(1/3)), pair(F0(ε), c), F1(pair(ε, a))), pair(ε, b)), c)).

$a^nb^{n+1}c^{n+2}$	abcd	1d	8	100000 (26)	-193033	1	1	$F0(x)$:=λx.append(pair(ε, a), if(flip(1/3), pair(pair(pair(x, b), c), c), F1(x))).<br>$F1(x)$:=λx.pair(Fm0(pair(x, b)), c).
$a^nb^{n+1}c^{n+2}$	abcd	1d	8	1 (1)	-43.0229	1	0.04	$F0(x)$:=λx.pair(pair(ε, a), b).<br>$F1(x)$:=λx.pair(pair(pair(F0(ε), b), c), c).<br>$F2(x)$:=λx.pair(Fm1(ε), c).
$a^nb^{n+1}c^{n+2}$	abcd	1d	8	10 (7)	-82.5811	0.95454	0.84	$F0(x)$:=λx.if(flip(5/24), pair(pair(x, b), c), Fm1(F2(pair(x, b)))).<br>$F1(x)$:=λx.append(pair(ε, a), x).<br>$F2(x)$:=λx.pair(F0(x), c).
$a^nb^{n+1}c^{n+2}$	abcd	1d	8	100 (10)	-260.504	1	0.88	$F0(x)$:=λx.if(not(flip(1/3)), pair(F2(x), c), pair(pair(pair(pair(x, b), c), c), c)).<br>$F1(x)$:=λx.ε.<br>$F2(x)$:=λx.Fm0(pair(append(pair(F1(ε), a), x), b)).
$a^nb^{n+1}c^{n+2}$	abcd	1d	8	1000 (16)	-2043.38	1	0.88	$F0(x)$:=λx.if(not(flip(1/3)), pair(F2(x), c), pair(pair(pair(pair(x, b), c), c), c)).<br>$F1(x)$:=λx.ε.<br>$F2(x)$:=λx.Fm0(pair(append(pair(F1(ε), a), x), b)).
$a^nb^{n+1}c^{n+2}$	abcd	1d	8	10000 (20)	-19424	1	1	$F0(x)$:=λx.append(pair(ε, a), if(not(flip(1/3)), F2(x), pair(pair(Fm1(x), c), c))).<br>$F1(x)$:=λx.pair(x, b).<br>$F2(x)$:=λx.pair(Fm0(pair(x, b)), c).
$a^nb^{n+1}c^{n+2}$	abcd	1d	8	100000 (26)	-193045	1	1	$F0(x)$:=λx.append(pair(ε, a), if(not(flip(1/3)), F2(x), pair(pair(Fm1(x), c), c))).<br>$F1(x)$:=λx.pair(x, b).<br>$F2(x)$:=λx.pair(Fm0(pair(x, b)), c).
$a^nb^{n+1}c^{n+2}$	abcd	1d	8	1 (1)	-50.9161	1	0.04	$F0(x)$:=λx.Fm2(ε).<br>$F1(x)$:=λx.pair(pair(pair(Fm0(ε), c), c), c).<br>$F2(x)$:=λx.pair(pair(pair(ε, a), b), b).<br>$F3(x)$:=λx.Fm1(ε).
$a^nb^{n+1}c^{n+2}$	abcd	1d	8	10 (7)	-91.9682	0.90909	0.8	$F0(x)$:=λx.pair(x, b).<br>$F1(x)$:=λx.ε.<br>$F2(x)$:=λx.F0(if(empty(x), Fm1(ε), append(pair(ε, a), x))).<br>$F3(x)$:=λx.pair(if(flip(1/6), x, F3(Fm2(x))), c).
$a^nb^{n+1}c^{n+2}$	abcd	1d	8	100 (10)	-264.222	1	0.64	$F0(x)$:=λx.ε.<br>$F1(x)$:=λx.Fm0(ε).<br>$F2(x)$:=λx.if(not(flip(1/3)), F3(x), pair(pair(pair(x, b), c), c)).<br>$F3(x)$:=λx.pair(Fm2(append(pair(F1(ε), a), pair(x, b))), c).
$a^nb^{n+1}c^{n+2}$	abcd	1d	8	1000 (16)	-2047.79	1	0.84	$F0(x)$:=λx.ε.<br>$F1(x)$:=λx.x.<br>$F2(x)$:=λx.if(not(flip(1/3)), Fm3(x), pair(pair(pair(Fm1(x), b), c), c)).<br>$F3(x)$:=λx.pair(Fm2(append(pair(Fm0(ε), a), pair(x, b))), c).
$a^nb^{n+1}c^{n+2}$	abcd	1d	8	10000 (20)	-19558.3	1	1	$F0(x)$:=λx.ε.<br>$F1(x)$:=λx.ε.<br>$F2(x)$:=λx.if(not(flip(3/8)), Fm3(x), pair(pair(pair(append(x, Fm1(F0(ε))), b), c), c)).<br>$F3(x)$:=λx.pair(Fm2(append(pair(ε, a), pair(x, b))), c).
$a^nb^{n+1}c^{n+2}$	abcd	1d	8	100000 (26)	-193064	1	1	$F0(x)$:=λx.append(pair(ε, a), x).<br>$F1(x)$:=λx.pair(Fm0(F2(x)), b).<br>$F2(x)$:=λx.pair(x, b).<br>$F3(x)$:=λx.pair(if(not(flip(1/3)), Fm3(pair(Fm0(x), b)), pair(pair(F1(x), c), c)), c).
$a^n \cup b^n$	abcd	10m	8	1 (1)	-19.3522	1	0.52	$F0(x)$:=λx.pair(if(flip(1/2), Fm0(ε), ε), a).
$a^n \cup b^n$	abcd	10m	8	10 (7)	-64.5679	0.52	0.96	$F0(x)$:=λx.insert(if(flip(1/2), head(pair(x, a)), Fm0(if(empty(x), sample(Σ), x))), x).
$a^n \cup b^n$	abcd	10m	8	100 (17)	-301.573	1	1	$F0(x)$:=λx.sample(if(flip(1/3), ((Σ\pair(ε, c))\pair(ε, d)), append(Fm0(ε), Fm0(ε)))).
$a^n \cup b^n$	abcd	10m	8	1000 (28)	-2663.92	1	1	$F0(x)$:=λx.sample(if(flip(1/3), ((Σ\pair(ε, c))\pair(ε, d)), append(Fm0(ε), Fm0(ε)))).
$a^n \cup b^n$	abcd	10m	8	10000 (41)	-26301.5	1	1	$F0(x)$:=λx.sample(if(flip(1/3), ((Σ\pair(ε, c))\pair(ε, d)), append(Fm0(ε), Fm0(ε)))).
$a^n \cup b^n$	abcd	10m	8	100000 (53)	-261274	1	1	$F0(x)$:=λx.sample(if(flip(1/3), ((Σ\pair(ε, c))\pair(ε, d)), append(Fm0(ε), Fm0(ε)))).
$a^n \cup b^n$	abcd	10m	8	1 (1)	-22.9713	1	0.04	$F0(x)$:=λx.append(x, x).<br>$F1(x)$:=λx.Fm0(pair(pair(ε, a), a)).
$a^n \cup b^n$	abcd	10m	8	10 (7)	-58.1664	0.5	1	$F0(x)$:=λx.append(if(flip(1/2), F0(x), ε), x).<br>$F1(x)$:=λx.F0(sample(Σ)).
$a^n \cup b^n$	abcd	10m	8	100 (17)	-302.177	1	1	$F0(x)$:=λx.sample(if(flip(1/2), x, pair(ε, b))).<br>$F1(x)$:=λx.append(Fm0(pair(ε, a)), if(flip(1/3), ε, Fm1(ε))).
$a^n \cup b^n$	abcd	10m	8	1000 (28)	-2663.83	1	1	$F0(x)$:=λx.sample(if(flip(1/2), pair(ε, b), pair(ε, a))).<br>$F1(x)$:=λx.append(Fm0(ε), if(flip(1/3), ε, Fm1(ε))).
$a^n \cup b^n$	abcd	10m	8	10000 (41)	-26301.4	1	1	$F0(x)$:=λx.sample(if(flip(1/2), pair(ε, b), pair(ε, a))).<br>$F1(x)$:=λx.append(Fm0(ε), if(flip(1/3), ε, Fm1(ε))).
$a^n \cup b^n$	abcd	10m	8	100000 (53)	-261274	1	1	$F0(x)$:=λx.sample(if(flip(1/2), pair(ε, b), pair(ε, a))).<br>$F1(x)$:=λx.append(Fm0(ε), if(flip(1/3), ε, Fm1(ε))).

$a^n \cup b^n$	abcd	10m	8	1 (1)	-30.4068	1	0.04	$F0(x)$:=λx.pair(pair(ϵ, a), a).<br>$F1(x)$:=λx.append(x, x).<br>$F2(x)$:=λx.Fm1(Fm0(ϵ)).
$a^n \cup b^n$	abcd	10m	8	10 (7)	-66.0075	0.5	1	$F0(x)$:=λx.ϵ.<br>$F1(x)$:=λx.append(x, if(flip(1/2), F1(x), Fm0(ϵ))).<br>$F2(x)$:=λx.Fm1(sample(Σ)).
$a^n \cup b^n$	abcd	10m	8	100 (17)	-309.354	1	1	$F0(x)$:=λx.ϵ.<br>$F1(x)$:=λx.append(x, if(not(flip(1/3)), F1(x), ϵ)).<br>$F2(x)$:=λx.Fm1(if(flip(1/2), pair(ϵ, a), pair(F0(ϵ), b))).
$a^n \cup b^n$	abcd	10m	8	1000 (28)	-2671.7	1	1	$F0(x)$:=λx.ϵ.<br>$F1(x)$:=λx.append(x, if(not(flip(1/3)), F1(x), ϵ)).<br>$F2(x)$:=λx.Fm1(if(flip(1/2), pair(ϵ, a), pair(F0(ϵ), b))).
$a^n \cup b^n$	abcd	10m	8	10000 (41)	-26307.9	1	1	$F0(x)$:=λx.append(Fm1(ϵ), if(not(flip(1/3)), F0(ϵ), ϵ)).<br>$F1(x)$:=λx.if(flip(1/2), pair(ϵ, b), pair(ϵ, a)).<br>$F2(x)$:=λx.F0(ϵ).
$a^n \cup b^n$	abcd	10m	8	100000 (53)	-261281	1	1	$F0(x)$:=λx.append(Fm1(ϵ), if(not(flip(1/3)), F0(ϵ), x)).<br>$F1(x)$:=λx.if(flip(1/2), pair(ϵ, b), pair(ϵ, a)).<br>$F2(x)$:=λx.Fm0(ϵ).
$a^n \cup b^n$	abcd	10m	8	1 (1)	-40.1925	0.5	0.08	$F0(x)$:=λx.append(x, x).<br>$F1(x)$:=λx.Fm2(ϵ).<br>$F2(x)$:=λx.sample(Σ).<br>$F3(x)$:=λx.F0(F0(F1(ϵ))).
$a^n \cup b^n$	abcd	10m	8	10 (7)	-73.4951	0.5	1	$F0(x)$:=λx.ϵ.<br>$F1(x)$:=λx.sample(Σ).<br>$F2(x)$:=λx.append(x, if(flip(1/2), ϵ, F3(ϵ))).<br>$F3(x)$:=λx.Fm2(Fm1(F0(ϵ))).
$a^n \cup b^n$	abcd	10m	8	100 (17)	-314.826	1	1	$F0(x)$:=λx.ϵ.<br>$F1(x)$:=λx.if(flip(1/2), pair(x, b), pair(ϵ, a)).<br>$F2(x)$:=λx.append(Fm1(F0(ϵ)), if(flip(1/3), ϵ, F2(x))).<br>$F3(x)$:=λx.F2(ϵ).
$a^n \cup b^n$	abcd	10m	8	1000 (28)	-2677.86	1	1	$F0(x)$:=λx.x.<br>$F1(x)$:=λx.if(flip(1/2), pair(ϵ, b), pair(ϵ, a)).<br>$F2(x)$:=λx.append(x, if(flip(1/3), ϵ, F2(F0(x)))).<br>$F3(x)$:=λx.Fm2(Fm1(ϵ)).
$a^n \cup b^n$	abcd	10m	8	10000 (41)	-26315.4	1	1	$F0(x)$:=λx.ϵ.<br>$F1(x)$:=λx.if(flip(1/2), pair(x, b), pair(Fm0(ϵ), a)).<br>$F2(x)$:=λx.append(x, if(flip(1/3), ϵ, F2(x))).<br>$F3(x)$:=λx.Fm2(Fm1(ϵ)).
$a^n \cup b^n$	abcd	10m	8	100000 (53)	-261288	1	1	$F0(x)$:=λx.ϵ.<br>$F1(x)$:=λx.if(flip(1/2), pair(x, b), pair(Fm0(ϵ), a)).<br>$F2(x)$:=λx.append(x, if(flip(1/3), ϵ, F2(x))).<br>$F3(x)$:=λx.Fm2(Fm1(ϵ)).
$a^n \cup a^n b^n$	abcd	1h	8	1 (1)	-6.6447	1	0.04	$F0(x)$:=λx.pair(ϵ, a).
$a^n \cup a^n b^n$	abcd	1h	8	10 (6)	-50.2794	1	0.48	$F0(x)$:=λx.pair(append(pair(ϵ, a), if(flip(1/2), ϵ, Fm0(ϵ))), b).
$a^n \cup a^n b^n$	abcd	1h	8	100 (16)	-391.443	0.48	0.76	$F0(x)$:=λx.if(flip(1/2), sample(((x\ϵ)∪insert(if(and(flip(1/2), empty(x)), F0(pair(if(flip(1/2), pair(ϵ, a), ϵ), a)), x), pair(insert(x, sample((ϵ∪x))), a)))), pair(F0(pair(x, a)), b)).
$a^n \cup a^n b^n$	abcd	1h	8	1000 (31)	-3263.56	0.48	1	$F0(x)$:=λx.append(pair(ϵ, a), sample(if(flip(1/8), F0(pair(x, a)), if(flip(1/4), if(not(flip(3/8)), pair(insert(x, if(flip(1/2), pair(if(flip(1/2), x, pair(x, a)), a), x)), a), x), pair(if(flip(5/24), ϵ, sample(if(empty(x), F0(ϵ), pair(pair(x, b), b)))), b))))).
$a^n \cup a^n b^n$	abcd	1h	8	10000 (42)	-30706.3	0.68	1	$F0(x)$:=λx.if(flip(1/8), ϵ, insert(if(not(flip(if(empty(x), 7/24, 1/24))), if(flip(1/4), x, pair(F0(pair(ϵ, b)), b)), pair(if(flip(5/12), ϵ, pair(if(flip(1/2), pair(Fm0(ϵ), a), ϵ), a)), a)), pair(ϵ, a))).
$a^n \cup a^n b^n$	abcd	1h	8	100000 (51)	-295018	0.72	1	$F0(x)$:=λx.if(flip(1/12), ϵ, insert(if(not(and(flip(if((x==ϵ), 3/8, 1/24)), (x==ϵ))), if(flip(7/24), x, pair(F0(pair(ϵ, b)), b)), pair(if(flip(1/3), ϵ, pair(if(not(flip(1/3)), pair(if(flip(1/4), ϵ, F0(ϵ)), a), ϵ), a)), a)), pair(ϵ, a))).
$a^n \cup a^n b^n$	abcd	1h	8	1 (1)	-13.2693	1	0.04	$F0(x)$:=λx.ϵ.<br>$F1(x)$:=λx.pair(Fm0(ϵ), a).
$a^n \cup a^n b^n$	abcd	1h	8	10 (6)	-55.8246	1	0.48	$F0(x)$:=λx.if(flip(1/2), Fm1(x), x).<br>$F1(x)$:=λx.pair(F0(pair(x, a)), b).
$a^n \cup a^n b^n$	abcd	1h	8	100 (16)	-329.234	0.6	1	$F0(x)$:=λx.if(flip(if(empty(x), 1/24, 1/2)), x, pair(Fm0(pair(x, a)), b)).<br>$F1(x)$:=λx.if(flip(1/2), pair(if(flip(1/2), x, F1(pair(x, a))), a), F0(x)).
$a^n \cup a^n b^n$	abcd	1h	8	1000 (31)	-2705.16	1	1	$F0(x)$:=λx.if(and(not((ϵ==x)), flip(1/3)), x, pair(Fm0(pair(x, a)), b)).<br>$F1(x)$:=λx.if(not(and(empty(x), flip(1/2))), pair(if(flip(1/3), ϵ, F1(pair(ϵ, a))), a), Fm0(ϵ)).
$a^n \cup a^n b^n$	abcd	1h	8	10000 (42)	-26259.5	1	1	$F0(x)$:=λx.if(and(not((ϵ==x)), flip(1/3)), x, pair(Fm0(pair(x, a)), b)).<br>$F1(x)$:=λx.if(not(and(empty(x), flip(1/2))), pair(if(flip(1/3), ϵ, F1(pair(ϵ, a))), a), Fm0(ϵ)).

$a^n \cup a^n b^n$	abcd	1h	8	100000 (51)	-261859	1	1	F0(x):=λx.if(and(not((ε==x)), flip(1/3)), x, pair(Fm0(pair(x, a)), b)).<br>F1(x):=λx.if(not(and(empty(x), flip(1/2))), pair(if(flip(1/3), ε, F1(pair(ε, a))), a), Fm0(ε)).
$a^n \cup a^n b^n$	abcd	1h	8	1 (1)	-20.7049	1	0.04	F0(x):=λx.pair(Fm1(ε), a).<br>F1(x):=λx.ε.<br>F2(x):=λx.F0(ε).
$a^n \cup a^n b^n$	abcd	1h	8	10 (6)	-65.4382	1	0.48	F0(x):=λx.pair(append(pair(F1(ε), a), if(flip(1/2), ε, Fm2(ε))), b).<br>F1(x):=λx.ε.<br>F2(x):=λx.F0(ε).
$a^n \cup a^n b^n$	abcd	1h	8	100 (16)	-301.393	1	1	F0(x):=λx.append(pair(ε, a), x).<br>F1(x):=λx.if(flip(1/2), x, ε).<br>F2(x):=λx.Fm0(if(not(flip(3/8)), F2(pair(x, b)), F1(pair(x, b)))).
$a^n \cup a^n b^n$	abcd	1h	8	1000 (31)	-2682.51	1	1	F0(x):=λx.insert(Fm1(ε), if(not(flip(1/3)), F2(ε), ε)).<br>F1(x):=λx.append(pair(ε, a), if(flip(1/2), pair(ε, b), ε)).<br>F2(x):=λx.Fm0(ε).
$a^n \cup a^n b^n$	abcd	1h	8	10000 (42)	-26237.5	1	1	F0(x):=λx.insert(Fm1(ε), if(not(flip(1/3)), F0(x), ε)).<br>F1(x):=λx.append(pair(ε, a), if(flip(1/2), pair(ε, b), ε)).<br>F2(x):=λx.Fm0(ε).
$a^n \cup a^n b^n$	abcd	1h	8	100000 (51)	-261837	1	1	F0(x):=λx.insert(Fm1(ε), if(not(flip(1/3)), F0(x), ε)).<br>F1(x):=λx.append(pair(ε, a), if(flip(1/2), pair(ε, b), ε)).<br>F2(x):=λx.Fm0(ε).
$a^n \cup a^n b^n$	abcd	1h	8	1 (1)	-28.598	1	0.04	F0(x):=λx.Fm2(ε).<br>F1(x):=λx.Fm0(ε).<br>F2(x):=λx.pair(ε, a).<br>F3(x):=λx.F1(ε).
$a^n \cup a^n b^n$	abcd	1h	8	10 (6)	-73.619	1	0.48	F0(x):=λx.append(Fm2(ε), pair(if(flip(1/2), Fm3(ε), Fm1(ε)), b)).<br>F1(x):=λx.ε.<br>F2(x):=λx.pair(ε, a).<br>F3(x):=λx.F0(ε).
$a^n \cup a^n b^n$	abcd	1h	8	100 (16)	-347.57	1	1	F0(x):=λx.pair(if(not(flip(3/8)), Fm0(head(ε)), ε), a).<br>F1(x):=λx.if(flip(7/24), ε, Fm3(pair(ε, b))).<br>F2(x):=λx.if(flip(1/2), pair(F1(Fm0(ε)), b), x).<br>F3(x):=λx.insert(Fm2(x), Fm0(ε)).
$a^n \cup a^n b^n$	abcd	1h	8	1000 (31)	-2690.67	1	1	F0(x):=λx.insert(if(flip(1/3), ε, Fm0(x)), x).<br>F1(x):=λx.pair(pair(x, a), b).<br>F2(x):=λx.pair(ε, a).<br>F3(x):=λx.F0(if(flip(1/2), F2(ε), Fm1(ε))).
$a^n \cup a^n b^n$	abcd	1h	8	10000 (42)	-26245	1	1	F0(x):=λx.insert(if(flip(1/3), ε, Fm0(x)), x).<br>F1(x):=λx.pair(x, b).<br>F2(x):=λx.ε.<br>F3(x):=λx.F0(if(flip(1/2), pair(F2(ε), a), Fm1(pair(ε, a)))).
$a^n \cup a^n b^n$	abcd	1h	8	100000 (51)	-261844	1	1	F0(x):=λx.insert(if(flip(1/3), ε, Fm0(x)), x).<br>F1(x):=λx.pair(x, b).<br>F2(x):=λx.ε.<br>F3(x):=λx.F0(if(flip(1/2), pair(F2(ε), a), Fm1(pair(ε, a)))).
$(ab)^n \cup (ba)^n$	abcd	1h	8	1 (1)	-25.8102	1	0.52	F0(x):=λx.pair(pair(if(flip(1/8), ε, Fm0(ε)), a), b).
$(ab)^n \cup (ba)^n$	abcd	1h	8	10 (8)	-74.562	0.5	0.52	F0(x):=λx.pair(pair(if(flip(7/24), if(flip(1/2), ε, pair(ε, b)), F0(ε)), a), b).
$(ab)^n \cup (ba)^n$	abcd	1h	8	100 (20)	-397.783	0.5	1	F0(x):=λx.insert(x, append(if(not(flip(7/24)), Fm0(pair(ε, b)), if(or(flip(3/8), empty(x)), pair(ε, b), ε)), if(and(empty(x), flip(1/2)), ε, pair(ε, a)))).
$(ab)^n \cup (ba)^n$	abcd	1h	8	1000 (27)	-3229.26	0.52	1	F0(x):=λx.if(not(flip(7/24)), append(pair(if(flip(if((x==ε), 3/8, 1/24)), x, pair(ε, b)), a), Fm0(pair(ε, b))), sample(if(or((ε==x), flip(3/8)), pair(pair(x, a), b), ε))).
$(ab)^n \cup (ba)^n$	abcd	1h	8	10000 (40)	-32429.8	0.52	1	F0(x):=λx.if(not(flip(7/24)), append(pair(if(and(empty(x), flip(5/12)), ε, pair(ε, b)), a), Fm0(pair(ε, b))), sample(if(or(flip(5/12), empty(x)), pair(pair(x, a), b), ε))).
$(ab)^n \cup (ba)^n$	abcd	1h	8	100000 (55)	-322631	0.52	1	F0(x):=λx.if(not(flip(7/24)), append(pair(if(and(empty(x), flip(if(flip(1/2), 1/2, 7/24))), ε, pair(ε, b)), a), Fm0(pair(ε, b))), sample(if(or(flip(5/12), empty(x)), pair(pair(x, a), b), ε))).
$(ab)^n \cup (ba)^n$	abcd	1h	8	1 (1)	-33.9415	0.96	0.52	F0(x):=λx.pair(F1(ε), a).<br>F1(x):=λx.if(flip(1/12), x, pair(Fm0(ε), b)).
$(ab)^n \cup (ba)^n$	abcd	1h	8	10 (8)	-81.5495	0.56	0.52	F0(x):=λx.pair(if(flip(1/12), ε, pair(if(not(flip(5/24)), F0(ε), ε), a)), b).<br>F1(x):=λx.Fm0(ε).
$(ab)^n \cup (ba)^n$	abcd	1h	8	100 (20)	-311.982	1	1	F0(x):=λx.if(flip(1/2), pair(pair(ε, b), a), pair(pair(ε, a), b)).<br>F1(x):=λx.append(if(not(flip(1/3)), Fm1(ε), ε), Fm0(ε)).
$(ab)^n \cup (ba)^n$	abcd	1h	8	1000 (27)	-2621.72	1	1	F0(x):=λx.if(flip(1/2), pair(pair(ε, b), a), pair(pair(ε, a), b)).<br>F1(x):=λx.append(if(not(flip(1/3)), Fm1(ε), ε), Fm0(ε)).

$(ab)^n \cup (ba)^n$	abcd	1h	8	10000 (40)	-26361.8	1	1	F0(x):=λx.if(flip(1/2), pair(pair(ϵ, b), a), pair(pair(ϵ, a), b)).<br>F1(x):=λx.append(if(not(flip(1/3)), Fm1(ϵ), ϵ), Fm0(ϵ)).
$(ab)^n \cup (ba)^n$	abcd	1h	8	100000 (55)	-261917	1	1	F0(x):=λx.if(flip(1/2), pair(pair(ϵ, b), a), pair(pair(ϵ, a), b)).<br>F1(x):=λx.append(if(not(flip(1/3)), Fm1(ϵ), ϵ), Fm0(ϵ)).
$(ab)^n \cup (ba)^n$	abcd	1h	8	1 (1)	-41.6622	1	0.52	F0(x):=λx.x.<br>F1(x):=λx.if(flip(1/8), ϵ, F2(ϵ)).<br>F2(x):=λx.F0(pair(pair(Fm1(ϵ), a), b)).
$(ab)^n \cup (ba)^n$	abcd	1h	8	10 (8)	-88.1891	0.6	0.52	F0(x):=λx.Fm1(if(flip(5/24), ϵ, F0(ϵ))).<br>F1(x):=λx.pair(if(flip(1/12), ϵ, pair(x, a)), b).<br>F2(x):=λx.F0(ϵ).
$(ab)^n \cup (ba)^n$	abcd	1h	8	100 (20)	-333.052	1	1	F0(x):=λx.insert(append(pair(ϵ, a), x), pair(if(flip(1/3), ϵ, Fm0(ϵ)), b)).<br>F1(x):=λx.if(flip(1/6), x, Fm0(if(flip(1/2), ϵ, x))).<br>F2(x):=λx.F1(pair(pair(ϵ, a), b)).
$(ab)^n \cup (ba)^n$	abcd	1h	8	1000 (27)	-2641.26	1	1	F0(x):=λx.append(pair(ϵ, a), pair(if(flip(3/8), ϵ, F1(ϵ)), b)).<br>F1(x):=λx.pair(pair(if(flip(1/3), ϵ, Fm1(ϵ)), b), a).<br>F2(x):=λx.sample(if(flip(1/2), F0(ϵ), F1(ϵ))).
$(ab)^n \cup (ba)^n$	abcd	1h	8	10000 (40)	-26383.6	1	1	F0(x):=λx.append(pair(ϵ, a), pair(if(flip(1/3), ϵ, F1(ϵ)), b)).<br>F1(x):=λx.pair(pair(if(flip(1/3), ϵ, F1(ϵ)), b), a).<br>F2(x):=λx.sample(if(flip(1/2), F0(ϵ), Fm1(ϵ))).
$(ab)^n \cup (ba)^n$	abcd	1h	8	100000 (55)	-261939	1	1	F0(x):=λx.append(pair(ϵ, a), pair(if(flip(1/3), ϵ, F1(ϵ)), b)).<br>F1(x):=λx.pair(pair(if(flip(1/3), ϵ, F1(ϵ)), b), a).<br>F2(x):=λx.sample(if(flip(1/2), F0(ϵ), Fm1(ϵ))).
$(ab)^n \cup (ba)^n$	abcd	1h	8	1 (1)	-49.2013	0.25	0.04	F0(x):=λx.pair(sample(Σ), b).<br>F1(x):=λx.Fm2(F0(ϵ)).<br>F2(x):=λx.append(x, append(x, x)).<br>F3(x):=λx.F2(Fm1(ϵ)).
$(ab)^n \cup (ba)^n$	abcd	1h	8	10 (8)	-105.968	0.125	1	F0(x):=λx.append(append(F1(x), Fm2(ϵ)), x).<br>F1(x):=λx.if(flip(1/2), F0(x), ϵ).<br>F2(x):=λx.sample(Σ).<br>F3(x):=λx.Fm0(sample(Σ)).
$(ab)^n \cup (ba)^n$	abcd	1h	8	100 (20)	-415.982	0.5	1	F0(x):=λx.ϵ.<br>F1(x):=λx.ϵ.<br>F2(x):=λx.if(or(not((x==F1(ϵ))), flip(1/2)), pair(x, b), F0(ϵ)).<br>F3(x):=λx.if(not(empty(if(flip(7/24), x, ϵ))), if(flip(1/2), x, pair(x, b)), Fm3(pair(Fm2(x), a))).
$(ab)^n \cup (ba)^n$	abcd	1h	8	1000 (27)	-3333.58	0.5	1	F0(x):=λx.pair(sample(if(flip(1/2), x, pair(ϵ, a))), b).<br>F1(x):=λx.F3(pair(ϵ, a)).<br>F2(x):=λx.ϵ.<br>F3(x):=λx.append(Fm0(x), if(not(flip(1/3)), F1(ϵ), sample((pair(F2(ϵ), a) ∪ ϵ)))).
$(ab)^n \cup (ba)^n$	abcd	1h	8	10000 (40)	-26413.1	1	1	F0(x):=λx.if(flip(5/24), pair(pair(Fm2(ϵ), a), b), append(Fm3(x), Fm3(ϵ))).<br>F1(x):=λx.pair(pair(ϵ, b), a).<br>F2(x):=λx.x.<br>F3(x):=λx.if(flip(1/6), Fm1(x), F0(ϵ)).
$(ab)^n \cup (ba)^n$	abcd	1h	8	100000 (55)	-262305	1	1	F0(x):=λx.if(flip(5/24), pair(x, b), append(Fm3(ϵ), Fm3(ϵ))).<br>F1(x):=λx.pair(pair(if(not(flip(1/8)), F2(ϵ), if(not(flip(1/12)), ϵ, Fm1(x))), b), a).<br>F2(x):=λx.ϵ.<br>F3(x):=λx.if(flip(1/6), Fm1(ϵ), F0(pair(ϵ, a))).
xx	ab	1h	8	1 (1)	-9.72199	1	0.04	F0(x):=λx.pair(pair(ϵ, a), a).
xx	ab	1h	8	10 (8)	-119.495	0.96774	1	F0(x):=λx.append(sample(if(flip(7/24), x, insert(Fm0(sample(Σ)), x))), x).
xx	ab	1h	8	100 (46)	-1308.75	0.96774	1	F0(x):=λx.append(sample(if(flip(7/24), x, insert(Fm0(sample(Σ)), x))), x).
xx	ab	1h	8	1000 (171)	-8549.2	0.96774	1	F0(x):=λx.append(sample(if(flip(7/24), x, insert(Fm0(sample(Σ)), x))), x).
xx	ab	1h	8	10000 (739)	-84508.5	0.96774	1	F0(x):=λx.append(sample(if(flip(7/24), x, insert(Fm0(sample(Σ)), x))), x).
xx	ab	1h	8	100000 (3165)	-847402	0.96774	1	F0(x):=λx.append(sample(if(flip(7/24), x, insert(Fm0(sample(Σ)), x))), x).
xx	ab	1h	8	1 (1)	-16.3466	1	0.04	F0(x):=λx.pair(ϵ, a).<br>F1(x):=λx.pair(F0(ϵ), a).
xx	ab	1h	8	10 (8)	-102.423	0.55555	0.32	F0(x):=λx.append(if(flip(1/2), ϵ, x), if(flip(1/2), append(pair(x, b), if(flip(1/2), sample(Σ), append(head(x), x))), x)).<br>F1(x):=λx.F0(Fm0(pair(sample(Σ), b))).
xx	ab	1h	8	100 (46)	-1274.69	1	1	F0(x):=λx.if(flip(1/3), append(x, x), F1(x)).<br>F1(x):=λx.F0(append(x, sample(Σ))).
xx	ab	1h	8	1000 (171)	-8164.14	1	1	F0(x):=λx.if(flip(3/8), append(x, x), F1(x)).<br>F1(x):=λx.F0(append(x, sample(Σ))).
xx	ab	1h	8	10000 (739)	-80595.3	1	1	F0(x):=λx.if(flip(3/8), append(x, x), F1(x)).<br>F1(x):=λx.F0(append(x, sample(Σ))).

xx	ab	1h	8	100000 (3165)	-808631	1	1	$F0(x)$:=λx.if(flip(3/8), append(x, x), F1(x)).<br>$F1(x)$:=λx.F0(append(x, sample(Σ))).
xx	ab	1h	8	1 (1)	-23.7822	1	0.04	$F0(x)$:=λx.F1(ε).<br>$F1(x)$:=λx.ε.<br>$F2(x)$:=λx.pair(pair(F0(ε), a), a).
xx	ab	1h	8	10 (8)	-150.963	0.05882	0.16	$F0(x)$:=λx.append(append(sample(Σ), x), if(flip(1/2), Fm0(if(flip(1/2), sample((Σ\x)), pair(x, b))), ε)).<br>$F1(x)$:=λx.Fm0(pair(ε, b)).<br>$F2(x)$:=λx.Fm1(ε).
xx	ab	1h	8	100 (46)	-1217.59	0.83333	1	$F0(x)$:=λx.append(sample(Σ), if(flip(3/8), ε, Fm0(ε))).<br>$F1(x)$:=λx.append(x, if(flip(1/8), pair(append(pair(pair(pair(ε, a), b), b), pair(head(x), b)), b), x)).<br>$F2(x)$:=λx.Fm1(F0(ε)).
xx	ab	1h	8	1000 (171)	-8175.38	1	1	$F0(x)$:=λx.if(not(flip(3/8)), Fm2(x), append(x, x)).<br>$F1(x)$:=λx.append(x, sample(Σ)).<br>$F2(x)$:=λx.Fm0(F1(x)).
xx	ab	1h	8	10000 (739)	-80606.6	1	1	$F0(x)$:=λx.if(not(flip(3/8)), F2(x), append(x, F1(x))).<br>$F1(x)$:=λx.x.<br>$F2(x)$:=λx.Fm0(append(x, sample(Σ))).
xx	ab	1h	8	100000 (3165)	-808643	1	1	$F0(x)$:=λx.if(not(flip(3/8)), F2(Fm1(x)), append(x, x)).<br>$F1(x)$:=λx.x.<br>$F2(x)$:=λx.Fm0(append(x, sample(Σ))).
xx	ab	1h	8	1 (1)	-31.6753	1	0.04	$F0(x)$:=λx.Fm1(ε).<br>$F1(x)$:=λx.ε.<br>$F2(x)$:=λx.F0(ε).<br>$F3(x)$:=λx.pair(pair(Fm2(ε), a), a).
xx	ab	1h	8	10 (8)	-112.742	0.86111	0.48	$F0(x)$:=λx.x.<br>$F1(x)$:=λx.x.<br>$F2(x)$:=λx.F0(if(flip(1/2), F2(append(x, sample(Σ))), append(F1(x), append(if(flip(1/6), ε, x), ε)))).<br>$F3(x)$:=λx.F2(pair(sample(Σ), b)).
xx	ab	1h	8	100 (46)	-1244.4	1	1	$F0(x)$:=λx.append(sample(Σ), if(flip(1/2), Fm2(ε), ε)).<br>$F1(x)$:=λx.append(x, x).<br>$F2(x)$:=λx.F0(ε).<br>$F3(x)$:=λx.F1(F0(ε)).
xx	ab	1h	8	1000 (171)	-8181.48	1	1	$F0(x)$:=λx.append(sample(Σ), if(not(flip(3/8)), F0(ε), Fm2(ε))).<br>$F1(x)$:=λx.append(x, x).<br>$F2(x)$:=λx.ε.<br>$F3(x)$:=λx.F1(Fm0(ε)).
xx	ab	1h	8	10000 (739)	-80612.7	1	1	$F0(x)$:=λx.append(sample(Σ), if(not(flip(3/8)), F0(ε), Fm2(ε))).<br>$F1(x)$:=λx.append(x, x).<br>$F2(x)$:=λx.ε.<br>$F3(x)$:=λx.F1(Fm0(ε)).
xx	ab	1h	8	100000 (3165)	-808180	1	1	$F0(x)$:=λx.append(x, x).<br>$F1(x)$:=λx.insert(sample(Σ), if(not(empty(ε)), ε, append(sample(if(flip(1/4), Σ, if(flip(1/2), Fm1(sample(Σ)), ε))), x))).<br>$F2(x)$:=λx.if(flip(1/3), sample(Σ), F1(sample(Σ))).<br>$F3(x)$:=λx.F0(F2(ε)).
xxx	ab	2h	8	1 (1)	-13.4925	1	0.04	$F0(x)$:=λx.pair(pair(pair(ε, b), b), b).
xxx	ab	2h	8	10 (7)	-186.654	0.16470	0.72	$F0(x)$:=λx.if(not(flip(11/24)), append(x, x), append(sample(Σ), F0(append(sample(Σ), x)))).
xxx	ab	2h	8	100 (37)	-1150.34	0.96774	1	$F0(x)$:=λx.if(flip(if(empty(x), 1/24, 5/12)), append(x, append(x, x)), F0(append(x, sample(Σ)))).
xxx	ab	2h	8	1000 (184)	-11848	0.96774	1	$F0(x)$:=λx.if(flip(if(empty(x), 1/24, 3/8)), append(x, append(x, x)), F0(append(x, sample(Σ)))).
xxx	ab	2h	8	10000 (769)	-121704	0.96774	1	$F0(x)$:=λx.if(flip(if(empty(x), 1/24, 3/8)), append(x, append(x, x)), F0(append(x, sample(Σ)))).
xxx	ab	2h	8	100000 (3216)	-1.18645e+06	0.96774	1	$F0(x)$:=λx.if(flip(if((x==ε), 1/24, 3/8)), append(x, append(x, x)), F0(append(x, sample(Σ)))).
xxx	ab	2h	8	1 (1)	-20.1171	1	0.04	$F0(x)$:=λx.ε.<br>$F1(x)$:=λx.pair(pair(pair(Fm0(ε), b), b), b).
xxx	ab	2h	8	10 (7)	-102.004	0.97142	1	$F0(x)$:=λx.x.<br>$F1(x)$:=λx.if(not(flip(1/3)), F1(append(Fm0(x), sample(if(empty(x), if(flip(1/8), Σ, pair(ε, b)), Σ)))), append(x, append(x, x))).
xxx	ab	2h	8	100 (37)	-1155.94	1	1	$F0(x)$:=λx.x.<br>$F1(x)$:=λx.if(or(empty(x), flip(1/2)), F1(append(x, sample(Σ))), append(append(x, x), F0(x))).
xxx	ab	2h	8	1000 (184)	-11821.2	1	1	$F0(x)$:=λx.x.<br>$F1(x)$:=λx.if(or(empty(x), not(flip(5/12))), F1(append(x, sample(Σ))), append(append(x, x), Fm0(x))).
xxx	ab	2h	8	10000 (769)	-121423	1	1	$F0(x)$:=λx.append(append(x, x), x).<br>$F1(x)$:=λx.if(not(flip(3/8)), F1(append(x, sample(Σ))), Fm0(insert(sample(Σ), x))).

xxx	ab	2h	8	100000 (3216)	-1.18348e+06	1	1	$F0(x)$:=λx.append(append(x, x), x).<br>$F1(x)$:=λx.if(not(flip(3/8)), F1(append(x, append(ε, sample(Σ)))), Fm0(insert(sample(Σ), x))).
xxx	ab	2h	8	1 (1)	-27.5526	1	0.04	$F0(x)$:=λx.pair(pair(pair(F1(ε), b), b), b).<br>$F1(x)$:=λx.ε.<br>$F2(x)$:=λx.Fm0(ε).
xxx	ab	2h	8	10 (7)	-127.039	0.22222	0.56	$F0(x)$:=λx.append(append(sample(if(flip(1/8), F1(x), (x∪Σ))), x), if(flip(5/24), pair(ε, b), x)).<br>$F1(x)$:=λx.pair(Fm0(if(flip(1/2), ε, pair(x, b))), b).<br>$F2(x)$:=λx.F0(F0(if(flip(1/2), ε, pair(ε, a)))).
xxx	ab	2h	8	100 (37)	-1692.81	0.38461	0.52	$F0(x)$:=λx.append(if(flip(1/2), if(flip(1/4), pair(Fm1(pair(sample(Σ), b)), a), sample(Σ)), F1(x)), x).<br>$F1(x)$:=λx.append(x, append(sample(if(flip(3/8), Σ, x)), if(not(flip(1/6)), ε, pair(ε, a)))).<br>$F2(x)$:=λx.F0(F0(sample(Σ))).
xxx	ab	2h	8	1000 (184)	-14032.8	1	1	$F0(x)$:=λx.if(flip(1/4), append(append(Fm1(x), insert(F1(tail(x)), ε)), head(x)), append(append(x, ε), append(x, x))).<br>$F1(x)$:=λx.append(append(sample((Σ∪ε)), x), sample(Σ)).<br>$F2(x)$:=λx.F0(Fm1(Fm0(x))).
xxx	ab	2h	8	10000 (769)	-138752	1	1	$F0(x)$:=λx.if(flip(5/24), append(append(F1(x), F1(head(x))), x), append(x, append(x, x))).<br>$F1(x)$:=λx.append(append(sample((Σ∪if((head(x)==x), x, ε))), x), sample(Σ)).<br>$F2(x)$:=λx.F0(Fm1(Fm0(ε))).
xxx	ab	2h	8	100000 (3216)	-1.38013e+06	1	1	$F0(x)$:=λx.if(flip(1/4), append(append(Fm1(x), insert(F1(tail(append(x, ε))), ε)), head(x)), append(append(x, head(ε)), append(x, x))).<br>$F1(x)$:=λx.append(append(sample((Σ∪ε)), x), sample(Σ)).<br>$F2(x)$:=λx.F0(Fm1(Fm0(x))).
xxx	ab	2h	8	1 (1)	-35.4458	1	0.04	$F0(x)$:=λx.pair(Fm1(ε), b).<br>$F1(x)$:=λx.pair(Fm2(ε), b).<br>$F2(x)$:=λx.ε.<br>$F3(x)$:=λx.pair(F0(ε), b).
xxx	ab	2h	8	10 (7)	-190.034	0.03448	0.28	$F0(x)$:=λx.Fm1(pair(pair(sample(append(x, x)), b), a)).<br>$F1(x)$:=λx.append(sample((x∪Σ)), append(sample(if(flip(3/8), x, Σ)), sample(Σ))).<br>$F2(x)$:=λx.F0(Fm0(if(flip(1/4), x, ε))).<br>$F3(x)$:=λx.F2(pair(pair(ε, a), b)).
xxx	ab	2h	8	100 (37)	-1111.64	0.83333	1	$F0(x)$:=λx.if(flip(1/2), append(sample(Σ), Fm0(ε)), sample(Σ)).<br>$F1(x)$:=λx.if(flip(1/12), pair(pair(x, a), b), append(append(x, x), x)).<br>$F2(x)$:=λx.F1(F0(x)).<br>$F3(x)$:=λx.Fm2(ε).
xxx	ab	2h	8	1000 (184)	-11780.4	1	1	$F0(x)$:=λx.insert(x, F2(sample(Σ))).<br>$F1(x)$:=λx.append(append(x, x), x).<br>$F2(x)$:=λx.sample(if(not(flip(1/2)), F0(x), x)).<br>$F3(x)$:=λx.Fm1(Fm0(ε)).
xxx	ab	2h	8	10000 (769)	-121459	1	1	$F0(x)$:=λx.insert(x, F2(sample(Σ))).<br>$F1(x)$:=λx.append(append(x, x), x).<br>$F2(x)$:=λx.sample(if(not(flip(5/12)), F0(x), x)).<br>$F3(x)$:=λx.Fm1(Fm0(ε)).
xxx	ab	2h	8	100000 (3216)	-1.18368e+06	1	1	$F0(x)$:=λx.insert(x, F2(sample(Σ))).<br>$F1(x)$:=λx.append(append(x, x), x).<br>$F2(x)$:=λx.sample(if(not(flip(5/12)), F0(x), x)).<br>$F3(x)$:=λx.Fm1(Fm0(ε)).
$xy : x \neq y$	ab	2d	8	1 (1)	-17.2629	1	0.04	$F0(x)$:=λx.pair(pair(pair(pair(ε, b), a), b), b).
$xy : x \neq y$	ab	2d	8	10 (10)	-140.637	0.97058	0.36	$F0(x)$:=λx.append(sample(Σ), if(flip(1/3), append(pair(ε, a), if(flip(1/4), x, pair(sample(Σ), a))), Fm0(pair(x, b)))).
$xy : x \neq y$	ab	2d	8	100 (76)	-1154.55	0.94444	1	$F0(x)$:=λx.append(sample(Σ), if(flip(3/8), insert(sample(Σ), sample((if(empty(x), if(and(flip(5/12), flip(11/24)), Σ, ε), pair(x, a))∪Σ))), F0(pair(x, b)))).
$xy : x \neq y$	ab	2d	8	1000 (438)	-11095.4	0.92857	1	$F0(x)$:=λx.append(sample(Σ), if(flip(if((head(x)==x), 5/24, 1/2)), append(sample(Σ), sample((head(x)∪Σ))), Fm0(pair(x, b)))).
$xy : x \neq y$	ab	2d	8	10000 (2277)	-111922	0.96666	1	$F0(x)$:=λx.append(append(sample((Σ∪x)), sample(Σ)), sample(if(flip(if((x==ε), 5/24, 1/2)), Σ, F0(pair(ε, a))))).
$xy : x \neq y$	ab	2d	8	100000 (10854)	-1.07347e+06	0.92857	1	$F0(x)$:=λx.insert(if(and((x==ε), not(flip(11/24))), Fm0(sample(pair(x, a))), if(not(flip(1/12)), append(sample(if(flip(1/6), append(x, if((x==ε), x, sample(Σ))), insert(sample((ε∪Σ)), sample((head(x)∪Σ))))), sample((Σ∪x))), sample((Σ∪x)))), append(sample(Σ), sample(Σ))).
$xy : x \neq y$	ab	2d	8	1 (1)	-23.8875	1	0.04	$F0(x)$:=λx.ε.<br>$F1(x)$:=λx.pair(pair(pair(pair(F0(ε), b), a), b), b).
$xy : x \neq y$	ab	2d	8	10 (10)	-152.687	1	0.6	$F0(x)$:=λx.append(sample(Σ), x).<br>$F1(x)$:=λx.F0(F0(if(flip(11/24), pair(Fm1(ε), b), Fm0(sample(Σ))))).
$xy : x \neq y$	ab	2d	8	100 (76)	-1130.04	1	0.92	$F0(x)$:=λx.append(insert(sample(Σ), x), sample((head(x)∪Σ))).<br>$F1(x)$:=λx.F0(Fm0(sample(if(flip(1/2), Fm0(if(flip(7/24), pair(sample(if(flip(1/2), F0(pair(pair(ε, a), a)), Σ)), b), ε)), ε)))).
$xy : x \neq y$	ab	2d	8	1000 (438)	-10822.7	0.92857	1	$F0(x)$:=λx.append(append(sample(if(flip(5/24), ε, Σ)), sample(Σ)), sample(Σ)).<br>$F1(x)$:=λx.append(if(flip(3/8), ε, F0(ε)), append(F0(ε), sample((ε∪Σ)))).

$xy : x \neq y$	ab	2d	8	10000 (2277)	-107985	0.92857	1	$F0(x)$:=λx.append(if(flip(1/2), pair(append(if((ε==x), if(flip(7/24), pair(ε, a), if(flip(1/24), pair(sample(Σ), b), ε)), ε), x), a), pair(x, b)), sample(Σ)).<br>$F1(x)$:=λx.F0(if(flip(5/24), sample(if(flip(7/24), ε, Σ)), append(append(sample(Σ), if(flip(5/24), if(flip(5/24), ε, x), insert(sample(Σ), sample(if(flip(7/24), ε, F0(ε)))))), sample(Σ)))).
$xy : x \neq y$	ab	2d	8	100000 (10854)	-1.07245e+06	0.92857	1	$F0(x)$:=λx.append(if(flip(1/2), pair(append(if((ε==x), if(flip(7/24), pair(ε, a), if(flip(1/24), pair(sample(Σ), b), ε)), ε), x), a), pair(x, b)), sample(Σ)).<br>$F1(x)$:=λx.F0(if(flip(5/24), sample(if(flip(7/24), ε, Σ)), append(append(sample(Σ), if(flip(5/24), if(flip(5/24), append(x, x), x), insert(sample(Σ), sample(if(flip(7/24), ε, F0(ε)))))), sample(Σ)))).
$xy : x \neq y$	ab	2d	8	1 (1)	-31.3231	1	0.04	$F0(x)$:=λx.ε.<br>$F1(x)$:=λx.pair(pair(pair(Fm0(ε), b), a), b).<br>$F2(x)$:=λx.pair(Fm1(ε), b).
$xy : x \neq y$	ab	2d	8	10 (10)	-159.069	0.92	0.84	$F0(x)$:=λx.if(flip(3/8), x, append(Fm1(x), x)).<br>$F1(x)$:=λx.Fm0(sample(Σ)).<br>$F2(x)$:=λx.append(F1(ε), Fm1(pair(ε, a))).
$xy : x \neq y$	ab	2d	8	100 (76)	-1142.97	1	1	$F0(x)$:=λx.append(sample(Σ), x).<br>$F1(x)$:=λx.append(sample((ε ∪ Σ)), x).<br>$F2(x)$:=λx.F0(F1(append(F1(sample(Σ)), F1(if(flip(1/2), Fm0(F0(pair(ε, b))), ε))))).
$xy : x \neq y$	ab	2d	8	1000 (438)	-10746.3	0.94444	1	$F0(x)$:=λx.if(not(flip(7/24)), F1(ε), ε).<br>$F1(x)$:=λx.append(x, sample((head(x) ∪ Σ))).<br>$F2(x)$:=λx.F1(append(F1(if(flip(3/8), Fm0(ε), append(Fm1(Fm1(ε)), append(sample(Σ), sample(Σ))))), if(flip(5/24), pair(pair(ε, a), b), sample(Σ)))).
$xy : x \neq y$	ab	2d	8	10000 (2277)	-108214	0.94444	1	$F0(x)$:=λx.if(not(flip(5/12)), F1(ε), ε).<br>$F1(x)$:=λx.append(x, sample((head(x) ∪ Σ))).<br>$F2(x)$:=λx.F1(append(F1(if(flip(3/8), Fm0(ε), append(Fm1(Fm1(x)), append(sample(Σ), sample(Σ))))), if(flip(1/8), pair(pair(ε, a), a), sample(Σ)))).
$xy : x \neq y$	ab	2d	8	100000 (10854)	-1.0738e+06	0.94444	1	$F0(x)$:=λx.if(not(flip(5/12)), F1(x), ε).<br>$F1(x)$:=λx.append(x, sample((head(x) ∪ Σ))).<br>$F2(x)$:=λx.F1(append(F1(if(flip(3/8), Fm0(ε), append(Fm1(Fm1(x)), append(sample(Σ), sample(Σ))))), if(flip(1/8), pair(pair(ε, a), a), sample(Σ)))).
$xy : x \neq y$	ab	2d	8	1 (1)	-39.2162	1	0.04	$F0(x)$:=λx.pair(pair(ε, b), a).<br>$F1(x)$:=λx.F0(F2(ε)).<br>$F2(x)$:=λx.ε.<br>$F3(x)$:=λx.pair(pair(F1(ε), b), b).
$xy : x \neq y$	ab	2d	8	10 (10)	-159.924	1	0.6	$F0(x)$:=λx.x.<br>$F1(x)$:=λx.F2(x).<br>$F2(x)$:=λx.insert(append(sample(Σ), if(flip(1/2), x, append(pair(head(x), a), Fm0(x)))), sample(Σ)).<br>$F3(x)$:=λx.Fm2(F1(if(flip(1/2), pair(pair(sample(Σ), b), b), ε))).
$xy : x \neq y$	ab	2d	8	100 (76)	-1171.26	0.92857	1	$F0(x)$:=λx.append(sample(Σ), append(sample((Σ ∪ ε)), x)).<br>$F1(x)$:=λx.Fm0(Fm2(if(flip(3/8), F0(ε), Fm0(F0(sample(Σ)))))).<br>$F2(x)$:=λx.x.<br>$F3(x)$:=λx.Fm1(ε).
$xy : x \neq y$	ab	2d	8	1000 (438)	-10723.6	1	1	$F0(x)$:=λx.append(sample((Σ\x)), x).<br>$F1(x)$:=λx.append(x, sample((if((x==head(x)), ε, Σ) ∪ Σ))).<br>$F2(x)$:=λx.append(sample(Σ), Fm1(x)).<br>$F3(x)$:=λx.Fm0(F2(if(flip(1/2), Fm2(Fm2(ε)), F1(if(flip(1/2), Fm1(ε), ε))))).
$xy : x \neq y$	ab	2d	8	10000 (2277)	-107745	1	1	$F0(x)$:=λx.append(sample((Σ\x)), x).<br>$F1(x)$:=λx.append(x, sample((if((x==head(x)), ε, Σ) ∪ Σ))).<br>$F2(x)$:=λx.append(sample(Σ), Fm1(x)).<br>$F3(x)$:=λx.Fm0(F2(if(not(flip(11/24)), Fm2(F2(x)), F1(if(flip(5/12), Fm1(ε), ε))))).
$xy : x \neq y$	ab	2d	8	100000 (10854)	-1.06851e+06	1	1	$F0(x)$:=λx.append(sample((Σ\sample(x))), x).<br>$F1(x)$:=λx.append(x, sample((if((x==head(x)), ε, Σ) ∪ Σ))).<br>$F2(x)$:=λx.append(sample(Σ), Fm1(x)).<br>$F3(x)$:=λx.Fm0(F2(if(not(flip(11/24)), Fm2(F2(x)), F1(if(flip(5/12), Fm1(ε), x))))).
xx^R	ab	1h	8	1 (1)	-68.685	0	0	$F0(x)$:=λx.append(if(flip(1/2), F0(pair(ε, b)), pair(pair(pair(x, b), a), b)), pair(if(flip(5/24), pair(x, b), ε), a)).
xx^R	ab	1h	8	10 (7)	-108.925	0.16666	0.88	$F0(x)$:=λx.append(sample(Σ), if(not(flip(1/2)), sample(Σ), append(Fm0(ε), sample(Σ)))).
xx^R	ab	1h	8	100 (35)	-978.277	1	1	$F0(x)$:=λx.append(if(or(empty(x), not(flip(5/12))), append(x, Fm0(sample(Σ))), x), x).
xx^R	ab	1h	8	1000 (172)	-7840.44	1	1	$F0(x)$:=λx.append(if(or(empty(x), not(flip(3/8))), append(x, Fm0(sample(Σ))), x), x).
xx^R	ab	1h	8	10000 (729)	-77506.8	1	1	$F0(x)$:=λx.append(if(or(empty(x), not(flip(3/8))), append(x, Fm0(sample(Σ))), x), x).
xx^R	ab	1h	8	100000 (3204)	-815907	1	1	$F0(x)$:=λx.append(if(or(empty(x), not(flip(3/8))), append(x, Fm0(sample(Σ))), x), x).
xx^R	ab	1h	8	1 (1)	-75.0009	0.07142	0.04	$F0(x)$:=λx.append(sample(if(flip(1/4), Σ, F0(ε))), if(flip(1/2), pair(pair(pair(pair(ε, a), a), a), b), pair(pair(ε, b), a))).<br>$F1(x)$:=λx.pair(pair(pair(Fm0(ε), a), b), b).

xx^R	ab	1h	8	10 (7)	-110.644	0.16666	0.8	F0(x):=λx.append(append(sample(Σ), x), if(flip(1/2), ϵ, F1(ϵ))).<br>F1(x):=λx.F0(sample(Σ)).
xx^R	ab	1h	8	100 (35)	-1233.09	0.16666	0.8	F0(x):=λx.append(append(sample(Σ), x), if(flip(1/2), ϵ, F1(ϵ))).<br>F1(x):=λx.F0(sample(Σ)).
xx^R	ab	1h	8	1000 (172)	-7840.18	1	1	F0(x):=λx.append(append(x, if(not(flip(3/8)), F1(ϵ), ϵ)), x).<br>F1(x):=λx.F0(sample(Σ)).
xx^R	ab	1h	8	10000 (729)	-77506.5	1	1	F0(x):=λx.append(append(x, if(not(flip(3/8)), F1(ϵ), ϵ)), x).<br>F1(x):=λx.F0(sample(Σ)).
xx^R	ab	1h	8	100000 (3204)	-815907	1	1	F0(x):=λx.append(append(x, if(not(flip(3/8)), F1(x), ϵ)), x).<br>F1(x):=λx.F0(sample(Σ)).
xx^R	ab	1h	8	1 (1)	-81.0072	0.02380	0	F0(x):=λx.pair(append(if(flip(1/2), pair(pair(ϵ, b), b), append(F0(append(x, x)), append(x, sample(Σ)))), if(flip(1/2), ϵ, pair(ϵ, a))), b).<br>F1(x):=λx.F0(x).<br>F2(x):=λx.Fm1(pair(ϵ, a)).
xx^R	ab	1h	8	10 (7)	-110.609	0.32142	0.4	F0(x):=λx.F1(if(flip(1/2), Fm0(append(sample(Σ), x)), append(pair(x, a), append(pair(ϵ, a), if(flip(1/2), ϵ, x))))).<br>F1(x):=λx.x.<br>F2(x):=λx.F0(ϵ).
xx^R	ab	1h	8	100 (35)	-991.311	1	1	F0(x):=λx.sample(if(not(flip(5/12)), append(x, Fm2(ϵ)), x)).<br>F1(x):=λx.append(Fm0(x), x).<br>F2(x):=λx.Fm1(sample(Σ)).
xx^R	ab	1h	8	1000 (172)	-7846.01	1	1	F0(x):=λx.F1(sample(Σ)).<br>F1(x):=λx.append(x, if(flip(3/8), x, append(F0(ϵ), x))).<br>F2(x):=λx.Fm0(ϵ).
xx^R	ab	1h	8	10000 (729)	-77512.3	1	1	F0(x):=λx.F1(sample(Σ)).<br>F1(x):=λx.append(x, if(flip(3/8), x, append(F0(ϵ), x))).<br>F2(x):=λx.Fm0(ϵ).
xx^R	ab	1h	8	100000 (3204)	-815912	1	1	F0(x):=λx.F1(sample(Σ)).<br>F1(x):=λx.append(x, if(flip(3/8), x, append(F0(ϵ), x))).<br>F2(x):=λx.Fm0(ϵ).
xx^R	ab	1h	8	1 (1)	-82.7853	0.03125	0	F0(x):=λx.pair(append(x, sample(Σ)), a).<br>F1(x):=λx.append(append(append(Fm0(head(x)), x), sample(Σ)), F0(x)).<br>F2(x):=λx.ϵ.<br>F3(x):=λx.Fm1(F1(Fm2(ϵ))).
xx^R	ab	1h	8	10 (7)	-127.195	0.19354	0.24	F0(x):=λx.F1(Fm2(ϵ)).<br>F1(x):=λx.append(x, if(flip(1/2), append(x, append(Fm0(x), append(x, pair(x, a)))), sample((Σ∪x)))).<br>F2(x):=λx.sample(Σ).<br>F3(x):=λx.F1(Fm1(ϵ)).
xx^R	ab	1h	8	100 (35)	-995.392	1	1	F0(x):=λx.Fm3(Fm2(ϵ)).<br>F1(x):=λx.append(x, if(flip(1/2), append(F0(ϵ), x), x)).<br>F2(x):=λx.ϵ.<br>F3(x):=λx.F1(sample(Σ)).
xx^R	ab	1h	8	1000 (172)	-7869.71	1	1	F0(x):=λx.Fm3(Fm2(ϵ)).<br>F1(x):=λx.append(x, if(flip(1/2), append(F0(ϵ), x), x)).<br>F2(x):=λx.ϵ.<br>F3(x):=λx.F1(sample(Σ)).
xx^R	ab	1h	8	10000 (729)	-77525.6	1	1	F0(x):=λx.append(x, head(x)).<br>F1(x):=λx.append(sample(Σ), F2(ϵ)).<br>F2(x):=λx.if(not(flip(3/8)), Fm3(ϵ), ϵ).<br>F3(x):=λx.Fm0(F1(ϵ)).
xx^R	ab	1h	8	100000 (3204)	-815926	1	1	F0(x):=λx.append(x, head(x)).<br>F1(x):=λx.append(sample(Σ), Fm2(ϵ)).<br>F2(x):=λx.if(not(flip(3/8)), Fm3(ϵ), ϵ).<br>F3(x):=λx.Fm0(Fm1(x)).
xx^I	ab	7d	8	1 (1)	-80.3446	0	0	F0(x):=λx.append(if(flip(1/2), Fm0(pair(pair(pair(pair(ϵ, b), b), b), a)), pair(pair(pair(pair(x, a), a), a), a)), insert(sample((pair(x, a)∪head(x))), sample(Σ))).
xx^I	ab	7d	8	10 (8)	-140.662	0.16666	0.8	F0(x):=λx.append(append(sample(Σ), sample(Σ)), if(flip(1/2), F0(ϵ), ϵ)).
xx^I	ab	7d	8	100 (36)	-598.39	0.96153	1	F0(x):=λx.sample(if(flip(if(empty(x), 1/2, 5/12)), pair(Fm0(append(pair(ϵ, a), x)), b), if(flip(if(empty(x), 1/24, 1/2)), x, pair(Fm0(append(pair(ϵ, b), x)), a)))).
xx^I	ab	7d	8	1000 (172)	-8347.04	0.96153	1	F0(x):=λx.sample(if(flip(if(empty(x), 1/2, 5/12)), pair(Fm0(append(pair(ϵ, a), x)), b), if(flip(if(empty(x), 1/24, 1/2)), x, pair(Fm0(append(pair(ϵ, b), x)), a)))).
xx^I	ab	7d	8	10000 (745)	-87061.8	0.96153	1	F0(x):=λx.sample(if(flip(if(empty(x), 1/2, 5/12)), pair(Fm0(append(pair(ϵ, a), x)), b), if(flip(if(empty(x), 1/24, 1/2)), x, pair(Fm0(append(pair(ϵ, b), x)), a)))).

xx^I	ab	7d	8	100000 (3176)	-864319	0.96153	1	$F0(x)$:=λx.sample(if(flip(if(empty(x), 1/2, 5/12)), pair(Fm0(append(pair(ϵ, a), x)), b), if(flip(if(empty(x), 1/24, 1/2)), x, pair(Fm0(append(pair(ϵ, b), x)), a)))).
xx^I	ab	7d	8	1 (1)	-76.1475	0	0	$F0(x)$:=λx.append(x, pair(append(sample(Σ), pair(x, a)), a)).<br>$F1(x)$:=λx.append(pair(pair(sample(if(flip(1/2), Fm1(ϵ), Σ)), b), b), F0(sample(Σ))).
xx^I	ab	7d	8	10 (8)	-131.345	0.53846	1	$F0(x)$:=λx.append(sample(($\Sigma\setminus$x)), insert(if(flip(1/2), Fm0(sample(Σ)), ϵ), x)).<br>$F1(x)$:=λx.F0(Fm0(ϵ)).
xx^I	ab	7d	8	100 (36)	-524.125	1	1	$F0(x)$:=λx.append(sample(($\Sigma\setminus$x)), if(not(flip(3/8)), insert(Fm1(ϵ), x), x)).<br>$F1(x)$:=λx.Fm0(sample(Σ)).
xx^I	ab	7d	8	1000 (172)	-7823.66	0.9375	1	$F0(x)$:=λx.insert(sample(if(not(flip(1/2)), (if(flip(1/24), append(x, x), Σ)$\setminus$x), F1(sample(($\Sigma\setminus$x))))), x).<br>$F1(x)$:=λx.append(F0(sample(Σ)), x).
xx^I	ab	7d	8	10000 (745)	-82744	1	1	$F0(x)$:=λx.append(x, insert(if(not(flip(3/8)), Fm1(ϵ), ϵ), sample(($\Sigma\setminus$x)))).<br>$F1(x)$:=λx.F0(sample(Σ)).
xx^I	ab	7d	8	100000 (3176)	-807836	1	1	$F0(x)$:=λx.append(x, insert(if(not(flip(3/8)), Fm1(ϵ), ϵ), sample(($\Sigma\setminus$x)))).<br>$F1(x)$:=λx.F0(sample(Σ)).
xx^I	ab	7d	8	1 (1)	-75.0559	0	0	$F0(x)$:=λx.pair(append(x, pair(pair(if(flip(1/2), Fm1(x), ϵ), a), a)), b).<br>$F1(x)$:=λx.append(append(Fm0(pair(sample(Σ), a)), x), x).<br>$F2(x)$:=λx.pair(Fm0(pair(pair(ϵ, b), b)), a).
xx^I	ab	7d	8	10 (8)	-147.709	0.17647	0.36	$F0(x)$:=λx.insert(if(flip(1/2), sample(Σ), pair(insert(x, x), b)), sample(($\Sigma\cup$pair(x, a)))).<br>$F1(x)$:=λx.F0(Fm0(Fm0(ϵ))).<br>$F2(x)$:=λx.Fm1(ϵ).
xx^I	ab	7d	8	100 (36)	-532.883	1	1	$F0(x)$:=λx.if(not(flip(3/8)), F2(ϵ), ϵ).<br>$F1(x)$:=λx.insert(sample(($\Sigma\setminus$x)), insert(F0(ϵ), x)).<br>$F2(x)$:=λx.Fm1(sample(Σ)).
xx^I	ab	7d	8	1000 (172)	-7562.14	1	1	$F0(x)$:=λx.ϵ.<br>$F1(x)$:=λx.append(if(flip(3/8), x, insert(F2(Fm0(ϵ)), x)), sample(($\Sigma\setminus$x))).<br>$F2(x)$:=λx.F1(sample(Σ)).
xx^I	ab	7d	8	10000 (745)	-82749.8	1	1	$F0(x)$:=λx.ϵ.<br>$F1(x)$:=λx.append(if(flip(3/8), x, insert(F2(Fm0(ϵ)), x)), sample(($\Sigma\setminus$x))).<br>$F2(x)$:=λx.F1(sample(Σ)).
xx^I	ab	7d	8	100000 (3176)	-807842	1	1	$F0(x)$:=λx.ϵ.<br>$F1(x)$:=λx.append(if(flip(3/8), x, insert(F2(Fm0(ϵ)), x)), sample(($\Sigma\setminus$x))).<br>$F2(x)$:=λx.F1(sample(Σ)).
xx^I	ab	7d	8	1 (1)	-92.1693	0	0	$F0(x)$:=λx.append(x, append(sample(Σ), pair(pair(x, a), a))).<br>$F1(x)$:=λx.Fm2(ϵ).<br>$F2(x)$:=λx.sample(if(flip(1/2), Fm3(ϵ), Σ)).<br>$F3(x)$:=λx.append(pair(pair(Fm1(ϵ), b), b), F0(sample(Σ))).
xx^I	ab	7d	8	10 (8)	-163.109	0.16666	0.92	$F0(x)$:=λx.F1(F1(Fm2(ϵ))).<br>$F1(x)$:=λx.append(sample(Σ), x).<br>$F2(x)$:=λx.if(flip(1/2), Fm0(ϵ), ϵ).<br>$F3(x)$:=λx.F0(ϵ).
xx^I	ab	7d	8	100 (36)	-540.147	1	1	$F0(x)$:=λx.append(x, Fm1(x)).<br>$F1(x)$:=λx.insert(if(not(flip(3/8)), F2(ϵ), ϵ), sample(($\Sigma\setminus$x))).<br>$F2(x)$:=λx.F0(sample(Σ)).<br>$F3(x)$:=λx.F2(ϵ).
xx^I	ab	7d	8	1000 (172)	-7573.03	1	1	$F0(x)$:=λx.append(x, Fm1(x)).<br>$F1(x)$:=λx.insert(if(not(flip(3/8)), F2(ϵ), ϵ), sample(($\Sigma\setminus$x))).<br>$F2(x)$:=λx.F0(sample(Σ)).<br>$F3(x)$:=λx.F2(ϵ).
xx^I	ab	7d	8	10000 (745)	-82760.7	1	1	$F0(x)$:=λx.append(x, Fm1(x)).<br>$F1(x)$:=λx.insert(if(not(flip(3/8)), F2(ϵ), ϵ), sample(($\Sigma\setminus$x))).<br>$F2(x)$:=λx.F0(sample(Σ)).<br>$F3(x)$:=λx.F2(ϵ).
xx^I	ab	7d	8	100000 (3176)	-807853	1	1	$F0(x)$:=λx.append(x, Fm1(x)).<br>$F1(x)$:=λx.insert(if(not(flip(3/8)), F2(ϵ), ϵ), sample(($\Sigma\setminus$x))).<br>$F2(x)$:=λx.F0(sample(Σ)).<br>$F3(x)$:=λx.F2(ϵ).
$x(x^R)^I$	ab	2d	8	1 (1)	-17.2629	1	0.04	$F0(x)$:=λx.pair(pair(pair(pair(ϵ, a), b), a), b).
$x(x^R)^I$	ab	2d	8	10 (9)	-74.0384	1	1	$F0(x)$:=λx.insert(sample((pair(pair(ϵ, a), b)$\cup$pair(pair(ϵ, b), a))), if(flip(1/2), F0(ϵ), ϵ)).
$x(x^R)^I$	ab	2d	8	100 (31)	-502.055	0.88	1	$F0(x)$:=λx.sample(if(not(flip(if(empty(x), 11/24, 7/24))), if(or(empty(x), flip(5/12)), pair(F0(pair(x, a)), b), if(flip(1/12), append(pair(ϵ, b), pair(x, a)), x))), pair(F0(pair(x, b)), a))).
$x(x^R)^I$	ab	2d	8	1000 (178)	-8407.7	0.84615	1	$F0(x)$:=λx.sample(if(not(flip(if(empty(x), 5/12, 1/4))), if(or(empty(x), flip(1/3)), pair(F0(pair(x, a)), b), if(flip(1/12), append(pair(ϵ, b), pair(x, a)), x)), pair(F0(pair(x, b)), a))).

$x(x^R)^I$	ab	2d	8	10000 (760)	-81357.7	1	1	$F0(x)$:=λx.if(flip(1/2), pair(append(pair(ε, a), if(flip(3/8), ε, Fm0(ε))), b), pair(append(pair(ε, b), if(flip(3/8), ε, Fm0(ε))), a)).
$x(x^R)^I$	ab	2d	8	100000 (3196)	-812242	1	1	$F0(x)$:=λx.if(flip(1/2), pair(append(pair(ε, a), if(flip(3/8), ε, Fm0(ε))), b), pair(append(pair(ε, b), if(flip(3/8), ε, Fm0(ε))), a)).
$x(x^R)^I$	ab	2d	8	1 (1)	-21.585	1	0.04	$F0(x)$:=λx.append(x, x).<br>$F1(x)$:=λx.Fm0(pair(pair(ε, a), b)).
$x(x^R)^I$	ab	2d	8	10 (9)	-73.4051	1	1	$F0(x)$:=λx.append(append(x, if(flip(1/2), Fm1(ε), ε)), sample((Σ\x))).<br>$F1(x)$:=λx.Fm0(sample(Σ)).
$x(x^R)^I$	ab	2d	8	100 (31)	-512.689	1	1	$F0(x)$:=λx.insert(sample((Σ\x)), sample(if(flip(5/12), x, append(x, Fm1(ε))))).<br>$F1(x)$:=λx.F0(sample(Σ)).
$x(x^R)^I$	ab	2d	8	1000 (178)	-7924.55	1	1	$F0(x)$:=λx.insert(sample((Σ\x)), sample(if(flip(3/8), x, append(x, Fm1(ε))))).<br>$F1(x)$:=λx.Fm0(sample(Σ)).
$x(x^R)^I$	ab	2d	8	10000 (760)	-81352.1	1	1	$F0(x)$:=λx.insert(sample((Σ\x)), sample(if(flip(3/8), x, append(x, Fm1(ε))))).<br>$F1(x)$:=λx.Fm0(sample(Σ)).
$x(x^R)^I$	ab	2d	8	100000 (3196)	-812235	1	1	$F0(x)$:=λx.append(sample((Σ\x)), if(not(flip(3/8)), insert(x, F1(ε)), x)).<br>$F1(x)$:=λx.Fm0(sample(Σ)).
$x(x^R)^I$	ab	2d	8	1 (1)	-29.0205	1	0.04	$F0(x)$:=λx.append(x, x).<br>$F1(x)$:=λx.ε.<br>$F2(x)$:=λx.F0(pair(pair(Fm1(ε), a), b)).
$x(x^R)^I$	ab	2d	8	10 (9)	-81.9393	1	1	$F0(x)$:=λx.append(sample((Σ\x)), if(flip(1/2), ε, Fm2(ε))).<br>$F1(x)$:=λx.append(F0(x), x).<br>$F2(x)$:=λx.Fm1(sample(Σ)).
$x(x^R)^I$	ab	2d	8	100 (31)	-515.815	1	1	$F0(x)$:=λx.insert(append(sample((Σ\x)), x), if(flip(1/2), F1(ε), ε)).<br>$F1(x)$:=λx.Fm0(sample(Σ)).<br>$F2(x)$:=λx.Fm1(ε).
$x(x^R)^I$	ab	2d	8	1000 (178)	-7932.97	1	1	$F0(x)$:=λx.sample((Σ\x)).<br>$F1(x)$:=λx.append(x, if(flip(3/8), Fm0(x), append(Fm2(ε), F0(x)))).<br>$F2(x)$:=λx.Fm1(sample(Σ)).
$x(x^R)^I$	ab	2d	8	10000 (760)	-81360.5	1	1	$F0(x)$:=λx.sample((Σ\x)).<br>$F1(x)$:=λx.append(x, if(flip(3/8), Fm0(x), append(Fm2(ε), F0(x)))).<br>$F2(x)$:=λx.Fm1(sample(Σ)).
$x(x^R)^I$	ab	2d	8	100000 (3196)	-812245	1	1	$F0(x)$:=λx.sample((Σ\x)).<br>$F1(x)$:=λx.append(x, if(flip(3/8), Fm0(x), append(Fm2(ε), F0(x)))).<br>$F2(x)$:=λx.Fm1(sample(Σ)).
$x(x^R)^I$	ab	2d	8	1 (1)	-36.9137	1	0.04	$F0(x)$:=λx.append(x, x).<br>$F1(x)$:=λx.Fm2(ε).<br>$F2(x)$:=λx.Fm0(pair(pair(ε, a), b)).<br>$F3(x)$:=λx.Fm1(ε).
$x(x^R)^I$	ab	2d	8	10 (9)	-91.0364	1	1	$F0(x)$:=λx.ε.<br>$F1(x)$:=λx.Fm0(ε).<br>$F2(x)$:=λx.insert(if(flip(1/2), Fm1(ε), Fm3(ε)), append(sample((Σ\x)), x)).<br>$F3(x)$:=λx.F2(sample(Σ)).
$x(x^R)^I$	ab	2d	8	100 (31)	-526.075	1	1	$F0(x)$:=λx.if(flip(1/2), insert(x, Fm3(ε)), x).<br>$F1(x)$:=λx.Fm0(append(F2(x), x)).<br>$F2(x)$:=λx.sample((Σ\x)).<br>$F3(x)$:=λx.F1(sample(Σ)).
$x(x^R)^I$	ab	2d	8	1000 (178)	-7927.19	1	1	$F0(x)$:=λx.F1(ε).<br>$F1(x)$:=λx.ε.<br>$F2(x)$:=λx.append(sample((Σ\x)), x).<br>$F3(x)$:=λx.insert(if(flip(1/2), Fm3(F0(F0(ε))), ε), Fm2(sample(Σ))).
$x(x^R)^I$	ab	2d	8	10000 (760)	-81364.5	1	1	$F0(x)$:=λx.if(flip(3/8), x, F3(x)).<br>$F1(x)$:=λx.append(x, F0(F2(x))).<br>$F2(x)$:=λx.sample((Σ\x)).<br>$F3(x)$:=λx.append(F1(sample(Σ)), x).
$x(x^R)^I$	ab	2d	8	100000 (3196)	-812249	1	1	$F0(x)$:=λx.if(flip(3/8), x, F3(x)).<br>$F1(x)$:=λx.append(x, F0(F2(x))).<br>$F2(x)$:=λx.sample((Σ\x)).<br>$F3(x)$:=λx.append(F1(sample(Σ)), x).
Unequal	ab	7d	8	1 (1)	-13.4925	1	0.04	$F0(x)$:=λx.pair(pair(pair(ε, b), b), b).
Unequal	ab	7d	8	10 (7)	-63.6662	1	0.28	$F0(x)$:=λx.append(if(flip(1/2), ε, pair(F0(ε), a)), sample(Σ)).
Unequal	ab	7d	8	100 (36)	-470.792	0.73333	1	$F0(x)$:=λx.append(if(flip(1/3), ε, F0(ε)), sample(Σ)).
Unequal	ab	7d	8	1000 (174)	-4853.8	0.73333	1	$F0(x)$:=λx.append(sample(Σ), if(flip(5/12), ε, append(sample(Σ), if(flip(5/24), ε, Fm0(ε))))).

Unequal	ab	7d	8	10000 (747)	-46487.3	0.85714	1	F0(x):=λx.if(flip(5/12), insert(append(sample(Σ), sample(Σ)), if(flip(1/24), append(pair(if(not(flip(1/2)), append(x, x), pair(x, a)), a), pair(ε, b)), F0(pair(ε, b))))), sample(if(flip(1/4), if(not(flip(3/8)), pair(sample(if(not(flip(1/3)), pair(ε, a), x)), a), pair(pair(ε, b), b)), Σ))).
Unequal	ab	7d	8	100000 (3163)	-461382	0.85714	1	F0(x):=λx.if(flip(5/12), insert(append(sample(Σ), sample(Σ)), if(flip(1/24), append(pair(if(flip(11/24), append(x, x), pair(x, a)), a), pair(ε, b)), F0(pair(ε, b))))), sample(if(flip(1/4), if(not(flip(5/12)), pair(sample(if(not(flip(7/24)), pair(ε, a), x)), a), pair(pair(ε, b), b)), Σ))).
Unequal	ab	7d	8	1 (1)	-20.1171	1	0.04	F0(x):=λx.ε.<br>F1(x):=λx.pair(pair(pair(Fm0(ε), b), b), b).
Unequal	ab	7d	8	10 (7)	-70.984	1	0.28	F0(x):=λx.append(if(flip(1/2), pair(F1(ε), a), ε), sample(Σ)).<br>F1(x):=λx.F0(ε).
Unequal	ab	7d	8	100 (36)	-478.074	0.88095	0.84	F0(x):=λx.if(flip(1/2), if(flip(1/2), append(x, x), x), insert(append(sample(Σ), x), Fm1(ε))).<br>F1(x):=λx.F0(sample(Σ)).
Unequal	ab	7d	8	1000 (174)	-4831.65	0.78571	1	F0(x):=λx.sample(if(flip(5/24), (pair(pair(ε, b), b) ∪ pair(pair(x, a), a)), (Σ ∪ pair(x, b)))).<br>F1(x):=λx.append(F0(x), if(flip(11/24), append(append(if(flip(5/12), insert(Fm0(pair(ε, a)), sample(Σ)), ε), append(sample(Σ), sample(if(not(flip(3/8)), ε, Σ)))), sample(Σ)), ε)).
Unequal	ab	7d	8	10000 (747)	-46125.9	0.75	1	F0(x):=λx.if(not(flip(1/24)), sample(if(not(flip(1/3)), if(not(flip(1/4)), insert(append(sample(if(flip(1/6), ε, Σ)), sample(Σ)), append(sample(Σ), x)), insert(head(pair(x, a)), head(x))), (Σ\x))), pair(x, b)).<br>F1(x):=λx.Fm0(if(flip(3/8), ε, F0(sample(Σ)))).
Unequal	ab	7d	8	100000 (3163)	-456766	0.75	1	F0(x):=λx.if(not(flip(1/12)), sample(if(not(flip(1/3)), if(not(flip(1/4)), insert(append(sample(if(flip(1/6), ε, Σ)), sample(Σ)), append(sample(Σ), x)), insert(head(pair(x, a)), head(x))), (Σ\x))), pair(x, b)).<br>F1(x):=λx.Fm0(if(flip(3/8), ε, F0(sample(Σ)))).
Unequal	ab	7d	8	1 (1)	-27.5526	1	0.04	F0(x):=λx.pair(pair(pair(F1(ε), b), b), b).<br>F1(x):=λx.ε.<br>F2(x):=λx.Fm0(ε).
Unequal	ab	7d	8	10 (7)	-78.3069	1	0.52	F0(x):=λx.append(x, Fm1(sample(Σ))).<br>F1(x):=λx.if(flip(1/2), x, Fm0(Fm0(x))).<br>F2(x):=λx.F0(ε).
Unequal	ab	7d	8	100 (36)	-477.903	0.96153	0.84	F0(x):=λx.ε.<br>F1(x):=λx.if(flip(11/24), append(append(x, F2(ε)), sample(Σ)), insert(if(flip(5/12), x, F0(ε)), x)).<br>F2(x):=λx.F1(sample(Σ)).
Unequal	ab	7d	8	1000 (174)	-4778.67	0.9	0.92	F0(x):=λx.if(flip(1/6), pair(x, b), if(not(flip(1/4)), ε, pair(x, a))).<br>F1(x):=λx.append(if(not(flip(7/24)), F0(append(if(flip(3/8), pair(sample(if(flip(1/4), (pair(x, b) ∪ pair(x, a)), Σ)), b), pair(sample((Σ ∪ ε)), a)), sample(Σ))), sample((ε ∪ head(x)))), x).<br>F2(x):=λx.F1(insert(F0(sample(Σ)), sample(Σ))).
Unequal	ab	7d	8	10000 (747)	-46115.6	0.9	1	F0(x):=λx.if(flip(1/6), pair(x, b), if(not(flip(5/24)), ε, pair(x, a))).<br>F1(x):=λx.append(if(not(flip(1/3)), F0(append(if(flip(11/24), pair(sample(if(flip(1/3), (pair(x, b) ∪ ε), Σ)), b), pair(sample((ε ∪ Σ)), a)), sample(Σ))), sample((head(ε) ∪ head(x)))), x).<br>F2(x):=λx.F1(insert(F0(sample(Σ)), sample(Σ))).
Unequal	ab	7d	8	100000 (3163)	-456527	0.9	1	F0(x):=λx.if(flip(1/6), pair(x, b), if(not(flip(5/24)), ε, pair(x, a))).<br>F1(x):=λx.append(if(not(flip(1/3)), F0(append(if(flip(11/24), pair(sample(if(flip(1/3), (pair(x, b) ∪ ε), Σ)), b), pair(sample((ε ∪ Σ)), a)), sample(Σ))), sample((head(ε) ∪ head(x)))), x).<br>F2(x):=λx.F1(insert(F0(sample(Σ)), sample(Σ))).
Unequal	ab	7d	8	1 (1)	-35.4458	1	0.04	F0(x):=λx.ε.<br>F1(x):=λx.pair(pair(pair(ε, b), b), b).<br>F2(x):=λx.F1(ε).<br>F3(x):=λx.F2(F0(ε)).
Unequal	ab	7d	8	10 (7)	-86.7754	1	0.52	F0(x):=λx.append(x, Fm2(sample(Σ))).<br>F1(x):=λx.ε.<br>F2(x):=λx.if(flip(1/2), x, Fm0(Fm0(x))).<br>F3(x):=λx.F0(F1(ε)).
Unequal	ab	7d	8	100 (36)	-494.132	0.73333	1	F0(x):=λx.F1(ε).<br>F1(x):=λx.F2(ε).<br>F2(x):=λx.append(if(flip(1/3), ε, Fm3(ε)), sample(Σ)).<br>F3(x):=λx.F0(ε).
Unequal	ab	7d	8	1000 (174)	-4763.17	0.9375	1	F0(x):=λx.Fm1(sample(Σ)).<br>F1(x):=λx.sample(if(flip(5/12), append(F2(ε), x), if(flip(1/4), append(head(x), x), head(x)))).<br>F2(x):=λx.append(sample(Σ), sample(Σ)).<br>F3(x):=λx.F1(F1(F0(ε))).
Unequal	ab	7d	8	10000 (747)	-45703	0.78571	1	F0(x):=λx.append(sample(Σ), x).<br>F1(x):=λx.F0(if(flip(7/24), head(x), insert(sample(Σ), x))).<br>F2(x):=λx.if(flip(1/2), sample(Σ), ε).<br>F3(x):=λx.if(not(flip(1/4)), append(Fm0(ε), sample((x ∪ if(or(flip(1/2), flip(1/2)), Fm0(F2(x)), if(flip(1/8), x, F1(sample(Σ))))))), Fm0(append(Fm1(Fm0(sample(Σ))), if(flip(5/24), ε, F1(ε))))).

Unequal	ab	7d	8	100000 (3163)	-451573	0.78571	1	$F0(x)$:=λx.append(sample(Σ), x).<br>$F1(x)$:=λx.F0(if(flip(7/24), head(x), insert(sample(Σ), x))).<br>$F2(x)$:=λx.if(flip(1/2), sample(Σ), x).<br>$F3(x)$:=λx.if(not(flip(1/4)), append(Fm0(x), sample((ε ∪ if(or(flip(1/2), flip(11/24)), Fm0(F2(ε)), append(ε, if(flip(1/4), ε, F1(sample(Σ)))))))), Fm0(append(Fm1(Fm0(sample(Σ))), if(flip(1/4), ε, F1(ε))))).
Bach 2	abcd	7d	8	1 (1)	-20.0355	1	0.04	$F0(x)$:=λx.pair(pair(pair(pair(ε, a), b), a), b).
Bach 2	abcd	7d	8	10 (9)	-130.223	0.96296	0.56	$F0(x)$:=λx.sample(if(flip(1/2), if(flip(1/2), pair(F0(pair(x, a)), b), x), insert(pair(Fm0(pair(if(flip(1/24), F0(ε), ε), b)), a), x))).
Bach 2	abcd	7d	8	100 (47)	-1312.14	0.39285	1	$F0(x)$:=λx.append(sample((pair(ε, a) ∪ pair(ε, b))), sample(if(flip(1/2), if(flip(11/24), pair(Fm0(ε), a), pair(ε, a)), pair(if(not(flip(1/2)), ε, F0(ε)), b)))).
Bach 2	abcd	7d	8	1000 (268)	-12886.3	0.33333	1	$F0(x)$:=λx.append(sample((pair(ε, a) ∪ pair(ε, b))), append(sample((pair(ε, a) ∪ pair(ε, b))), sample(if(flip(1/2), F0(ε), ε)))).
Bach 2	abcd	7d	8	10000 (1562)	-130579	0.65517	0.72	$F0(x)$:=λx.if(flip(11/24), append(pair(ε, a), sample(if(flip(11/24), Fm0(pair(x, b)), pair(x, b)))), append(pair(pair(sample(if(flip(1/3), pair(sample((pair(head(x), b) ∪ (head(x) ∪ pair(ε, a)))), b), ε)), b), a), if(not(flip(1/2)), x, append(Fm0(ε), head(x))))).
Bach 2	abcd	7d	8	100000 (8948)	-1.27625e+06	0.65517	0.72	$F0(x)$:=λx.if(flip(11/24), append(pair(ε, a), sample(if(flip(if(empty(x), 1/2, 11/24)), Fm0(pair(x, b)), pair(x, b)))), append(pair(pair(sample(if(flip(1/3), pair(sample((pair(head(x), b) ∪ (head(x) ∪ pair(ε, a)))), b), ε)), b), a), if(not(flip(1/2)), x, append(F0(ε), x)))).
Bach 2	abcd	7d	8	1 (1)	-22.9713	1	0.04	$F0(x)$:=λx.append(x, x).<br>$F1(x)$:=λx.Fm0(pair(pair(ε, a), b)).
Bach 2	abcd	7d	8	10 (9)	-118.114	1	0.72	$F0(x)$:=λx.append(if(empty(x), pair(ε, b), ε), pair(if(flip(1/2), Fm1(ε), ε), a)).<br>$F1(x)$:=λx.if(flip(1/2), pair(append(x, Fm1(pair(ε, a))), b), F0(x)).
Bach 2	abcd	7d	8	100 (47)	-1238.6	0.76	0.92	$F0(x)$:=λx.insert(sample(if(flip(5/24), pair(pair(ε, a), b), if(not(or(empty(x), flip(5/24))), ε, pair(sample(((x\ε) ∪ pair(ε, b))), a)))), x).<br>$F1(x)$:=λx.Fm0(append(if(flip(11/24), Fm0(F0(F0(ε))), ε), Fm0(Fm0(Fm0(ε))))).
Bach 2	abcd	7d	8	1000 (268)	-12222.1	0.46153	0.68	$F0(x)$:=λx.sample(if(flip(5/12), pair(pair(ε, a), b), if(flip(5/24), insert(if(not(flip(1/8)), x, append(pair(ε, b), x)), insert(if(flip(5/24), insert(if(flip(5/12), x, ε), x), ε), x)), x))).<br>$F1(x)$:=λx.append(sample(if(flip(1/2), if(flip(7/24), pair(sample(if(empty(x), pair(pair(append(if(flip(3/8), ε, pair(ε, b)), Fm0(ε)), a), a), if(flip(1/12), pair(ε, a), ε))), a), Fm1(pair(ε, b))), if(flip(1/24), pair(x, b), ε))), F0(pair(pair(ε, b), a))).
Bach 2	abcd	7d	8	10000 (1562)	-127661	0.29629	1	$F0(x)$:=λx.if(not(flip(11/24)), pair(x, b), pair(x, a)).<br>$F1(x)$:=λx.F0(if(flip(if((head(x)==x), 1/3, 1/2)), F0(x), F1(Fm0(x)))).
Bach 2	abcd	7d	8	100000 (8948)	-1.25419e+06	0.29629	1	$F0(x)$:=λx.if(not(and(flip(11/24), empty(ε))), pair(x, b), pair(x, a)).<br>$F1(x)$:=λx.F0(if(flip(if((head(x)==x), 1/3, 1/2)), Fm0(x), F1(Fm0(x)))).
Bach 2	abcd	7d	8	1 (1)	-30.4068	1	0.04	$F0(x)$:=λx.append(x, x).<br>$F1(x)$:=λx.ε.<br>$F2(x)$:=λx.F0(pair(pair(Fm1(ε), a), b)).
Bach 2	abcd	7d	8	10 (9)	-133.442	0.33333	0.4	$F0(x)$:=λx.ε.<br>$F1(x)$:=λx.if(flip(1/2), pair(if(flip(1/2), F2(Fm0(ε)), ε), a), pair(ε, b)).<br>$F2(x)$:=λx.append(Fm1(ε), F1(ε)).
Bach 2	abcd	7d	8	100 (47)	-1272.56	0.29629	1	$F0(x)$:=λx.if(not(flip(11/24)), x, F2(x)).<br>$F1(x)$:=λx.if(flip(11/24), pair(x, b), pair(x, a)).<br>$F2(x)$:=λx.F1(F0(Fm1(x))).
Bach 2	abcd	7d	8	1000 (268)	-12407.7	0.29629	1	$F0(x)$:=λx.if(flip(11/24), pair(x, a), pair(x, b)).<br>$F1(x)$:=λx.F0(if(flip(if((head(x)==x), 3/8, 1/2)), F0(x), Fm1(Fm0(x)))).<br>$F2(x)$:=λx.F1(ε).
Bach 2	abcd	7d	8	10000 (1562)	-125357	0.63414	0.96	$F0(x)$:=λx.if(flip(1/2), append(pair(ε, a), append(ε, x)), if((pair(ε, a)==head(x)), append(pair(x, b), head(x)), pair(x, a))).<br>$F1(x)$:=λx.if(flip(1/2), insert(pair(ε, a), if(flip(5/24), if(not(flip(1/3)), append(pair(ε, b), pair(pair(append(if(flip(11/24), head(x), pair(if(flip(1/6), head(pair(if(flip(1/8), ε, x), a)), ε), a)), x), b), a)), pair(pair(x, a), a)), x)), Fm0(Fm2(x))).<br>$F2(x)$:=λx.if(flip(1/2), Fm1(pair(x, b)), pair(append(x, F1(ε)), b)).
Bach 2	abcd	7d	8	100000 (8948)	-1.22935e+06	0.63414	0.96	$F0(x)$:=λx.if(flip(1/2), append(pair(ε, a), append(ε, x)), if((pair(ε, a)==head(x)), append(pair(x, b), head(x)), pair(x, a))).<br>$F1(x)$:=λx.if(flip(1/2), insert(pair(ε, a), if(flip(5/24), if(not(flip(1/3)), append(pair(ε, b), pair(pair(append(if(flip(11/24), head(x), pair(if(flip(1/6), head(pair(if(flip(1/8), ε, x), a)), ε), a)), x), b), a)), pair(pair(x, a), a)), x)), Fm0(Fm2(x))).<br>$F2(x)$:=λx.if(flip(1/2), Fm1(pair(x, b)), pair(append(x, F1(ε)), b)).
Bach 2	abcd	7d	8	1 (1)	-38.3	1	0.04	$F0(x)$:=λx.Fm1(ε).<br>$F1(x)$:=λx.Fm2(pair(pair(ε, a), b)).<br>$F2(x)$:=λx.append(x, x).<br>$F3(x)$:=λx.Fm0(ε).
Bach 2	abcd	7d	8	10 (9)	-141.014	0.61538	0.52	$F0(x)$:=λx.if(flip(1/2), x, Fm3(ε)).<br>$F1(x)$:=λx.if(flip(1/2), x, insert(Fm3(x), Fm3(ε))).<br>$F2(x)$:=λx.pair(pair(ε, b), a).<br>$F3(x)$:=λx.if(flip(1/3), append(pair(pair(Fm0(x), a), b), head(x)), F1(Fm2(ε))).
Bach 2	abcd	7d	8	100 (47)	-1280.3	0.29629	1	$F0(x)$:=λx.if(flip(11/24), Fm2(ε), ε).<br>$F1(x)$:=λx.sample(if(flip(11/24), pair(x, b), pair(x, a))).<br>$F2(x)$:=λx.Fm3(ε).<br>$F3(x)$:=λx.F1(Fm1(F0(ε))).

Bach 2	abcd	7d	8	1000 (268)	-12390.6	0.29629	1	F0(x):=λx.sample(if(flip(1/2), F3(x), ε)).<br>F1(x):=λx.insert(F2(ε), append(F2(ε), x)).<br>F2(x):=λx.if(flip(11/24), pair(ε, a), pair(ε, b)).<br>F3(x):=λx.Fm1(F0(pair(append(pair(x, a), x), d))).
Bach 2	abcd	7d	8	10000 (1562)	-128501	0.38461	1	F0(x):=λx.append(append(if(flip(1/8), append(pair(ε, a), pair(ε, b)), ε), F2(pair(ε, b))), x).<br>F1(x):=λx.F0(ε).<br>F2(x):=λx.if(not(flip(1/12)), sample((x ∪ pair(ε, a))), pair(pair(x, a), a)).<br>F3(x):=λx.Fm0(F0(if(flip(5/12), ε, F0(F1(ε))))).
Bach 2	abcd	7d	8	100000 (8948)	-1.25689e+06	0.38461	1	F0(x):=λx.append(append(if(flip(1/8), append(pair(ε, a), pair(ε, b)), ε), F2(pair(ε, b))), x).<br>F1(x):=λx.Fm0(ε).<br>F2(x):=λx.if(not(flip(1/12)), sample((x ∪ pair(ε, a))), pair(pair(x, a), a)).<br>F3(x):=λx.Fm0(F0(if(flip(5/12), x, F0(F1(x))))).
Bach 3	abcd	7d	8	1 (1)	-15.5719	1	0.04	F0(x):=λx.pair(pair(pair(ε, c), a), b).
Bach 3	abcd	7d	8	10 (10)	-426.793	0.05555	0.08	F0(x):=λx.append(sample((Σ\pair(ε, d))), if(flip(1/2), Fm0(pair(ε, a)), pair(if(flip(1/2), pair(if(flip(1/2), pair(pair(ε, a), b), insert(x, pair(x, c))), c), x), b))).
Bach 3	abcd	7d	8	100 (69)	-2783.77	0.09090	0.2	F0(x):=λx.append(sample((Σ\pair(ε, d))), if(flip(1/3), pair(if(not(flip(5/12)), pair(ε, a), pair(if(or(flip(7/24), flip(1/2)), ε, pair(pair(ε, a), b)), b)), c), if(flip(1/4), if(flip(7/24), pair(pair(pair(x, b), b), a), pair(pair(x, a), b)), F0(pair(ε, c))))).
Bach 3	abcd	7d	8	1000 (540)	-34841.9	0.22222	0.24	F0(x):=λx.sample(if(or(empty(x), flip(3/8)), append(sample((Σ\pair(ε, d))), insert(F0(sample((Σ\pair(ε, d)))), x)), append(sample((Σ\pair(ε, d))), x))).
Bach 3	abcd	7d	8	10000 (4068)	-343062	0.22222	0.24	F0(x):=λx.if(not(empty(x)), append(x, sample((Σ\pair(ε, d)))), F0(Fm0(sample(if(flip(1/2), (Σ\pair(ε, d)), if(not(flip(1/24)), Fm0(ε), (ε\ε))))))).
Bach 3	abcd	7d	8	100000 (29915)	-3.39421e+06	0.22222	0.24	F0(x):=λx.append(sample((Σ\pair(x, d))), append(if(flip(1/3), Fm0(ε), sample((Σ\pair(ε, d)))), sample((Σ\append(x, pair(x, d)))))).
Bach 3	abcd	7d	8	1 (1)	-22.1965	1	0.04	F0(x):=λx.ε.<br>F1(x):=λx.pair(pair(pair(F0(ε), c), a), b).
Bach 3	abcd	7d	8	10 (10)	-419.284	0	0.04	F0(x):=λx.if(flip(3/8), if(flip(1/2), pair(insert(if(flip(1/2), x, ε), pair(pair(sample(((Σ ∪ ε) ∪ pair(x, c))), a), a)), b), pair(pair(pair(pair(pair(x, b), b), c), a), c)), insert(x, sample((Σ\pair(ε, d))))).<br>F1(x):=λx.pair(Fm0(F0(Fm0(ε))), c).
Bach 3	abcd	7d	8	100 (69)	-2755.34	0.22222	0.24	F0(x):=λx.insert(sample((Σ\pair(ε, d))), x).<br>F1(x):=λx.Fm0(append(F0(F0(ε)), if(not(flip(1/3)), Fm0(F0(ε)), tail(sample(Σ))))).
Bach 3	abcd	7d	8	1000 (540)	-34201.7	0.02343	0.24	F0(x):=λx.append(x, sample((Σ\(pair(ε, d) ∪ x)))).<br>F1(x):=λx.Fm0(if(and(empty(x), not(or(flip(5/24), flip(1/4)))), Fm1(F0(ε)), F0(append(x, F0(ε))))).
Bach 3	abcd	7d	8	10000 (4068)	-335345	0.02343	0.24	F0(x):=λx.append(x, sample((Σ\(pair(ε, d) ∪ x)))).<br>F1(x):=λx.Fm0(if(and(empty(x), not(or(flip(5/24), flip(1/4)))), Fm1(F0(ε)), F0(append(x, F0(ε))))).
Bach 3	abcd	7d	8	100000 (29915)	-3.32338e+06	0.02343	0.24	F0(x):=λx.append(x, sample(((Σ\(ε ∪ x))\pair(ε, d)))).<br>F1(x):=λx.Fm0(if(and((x==ε), not(or(flip(5/24), flip(1/4)))), Fm1(F0(ε)), F0(append(x, F0(ε))))).
Bach 3	abcd	7d	8	1 (1)	-29.6321	1	0.04	F0(x):=λx.pair(pair(ε, c), a).<br>F1(x):=λx.pair(Fm0(ε), b).<br>F2(x):=λx.F1(ε).
Bach 3	abcd	7d	8	10 (10)	-431.078	0.13265	0.32	F0(x):=λx.append(sample((Σ\(pair(ε, d) ∪ x))), x).<br>F1(x):=λx.F0(Fm0(x)).<br>F2(x):=λx.F0(F1(F1(if(or(flip(1/2), flip(1/2)), pair(ε, c), pair(ε, b))))).
Bach 3	abcd	7d	8	100 (69)	-2719.21	0.15384	0.6	F0(x):=λx.append(x, F1(ε)).<br>F1(x):=λx.append(if(not(flip(1/3)), x, append(x, pair(if(flip(5/24), pair(x, a), ε), a))), if(flip(5/12), pair(ε, c), pair(ε, b))).<br>F2(x):=λx.Fm0(sample(if(flip(1/2), F0(ε), Fm1(F0(Fm0(ε)))))).
Bach 3	abcd	7d	8	1000 (540)	-34210.8	0.02343	0.24	F0(x):=λx.append(x, sample((Σ\(pair(F1(ε), d) ∪ x)))).<br>F1(x):=λx.ε.<br>F2(x):=λx.Fm0(if(and(empty(x), not(or(flip(5/24), flip(1/4)))), F2(F0(ε)), Fm0(append(x, Fm0(ε))))).
Bach 3	abcd	7d	8	10000 (4068)	-335354	0.02343	0.24	F0(x):=λx.append(x, sample((Σ\(pair(F1(ε), d) ∪ x)))).<br>F1(x):=λx.ε.<br>F2(x):=λx.Fm0(if(and(empty(x), not(or(flip(5/24), flip(1/4)))), F2(F0(ε)), Fm0(append(x, Fm0(ε))))).
Bach 3	abcd	7d	8	100000 (29915)	-3.32339e+06	0.02343	0.24	F0(x):=λx.append(x, sample(((Σ\pair(ε, d))\x))).<br>F1(x):=λx.x.<br>F2(x):=λx.Fm0(if(and((head(ε)==x), not(or(flip(5/24), flip(1/4)))), F2(F0(Fm1(x))), Fm0(append(x, F0(ε))))).
Bach 3	abcd	7d	8	1 (1)	-37.5252	1	0.04	F0(x):=λx.ε.<br>F1(x):=λx.F0(ε).<br>F2(x):=λx.pair(pair(pair(ε, c), a), b).<br>F3(x):=λx.F2(F1(ε)).
Bach 3	abcd	7d	8	10 (10)	-429.959	0	0.56	F0(x):=λx.F2(F2(x)).<br>F1(x):=λx.sample(if(flip(1/2), pair(ε, c), pair(ε, b))).<br>F2(x):=λx.append(if(flip(1/2), pair(ε, a), F1(ε)), x).<br>F3(x):=λx.Fm0(F0(F2(F1(ε)))).

Bach 3	abcd	7d	8	100 (69)	-2760.47	0.03333	0.24	F0(x):=λx.insert(x, Fm2(x)).<br>F1(x):=λx.F0(F0(F0(if(flip(1/2), F0(x), if(flip(7/24), append(x, x), ϵ))))).<br>F2(x):=λx.sample(((Σ\x)\pair(ϵ, d))).<br>F3(x):=λx.F1(F2(ϵ)).
Bach 3	abcd	7d	8	1000 (540)	-33902.3	0.02352	0.24	F0(x):=λx.ϵ.<br>F1(x):=λx.sample(((Σ\x)\pair(ϵ, d))).<br>F2(x):=λx.append(F1(x), append(x, F1(x))).<br>F3(x):=λx.F2(append(F1(Fm0(ϵ)), if(flip(1/3), ϵ, F2(ϵ)))).
Bach 3	abcd	7d	8	10000 (4068)	-332759	0.03448	0.24	F0(x):=λx.sample(((Σ\x)\pair(ϵ, d))).<br>F1(x):=λx.insert(Fm0(x), append(x, F0(x))).<br>F2(x):=λx.F1(x).<br>F3(x):=λx.Fm2(append(if(not(flip(1/3)), F2(ϵ), ϵ), F0(Fm0(if(flip(1/2), ϵ, Fm0(x)))))).
Bach 3	abcd	7d	8	100000 (29915)	-3.29719e+06	0.03448	0.24	F0(x):=λx.sample(((Σ\x)\pair(ϵ, d))).<br>F1(x):=λx.insert(Fm0(x), append(x, F0(x))).<br>F2(x):=λx.F1(x).<br>F3(x):=λx.Fm2(append(if(not(flip(1/3)), F2(ϵ), ϵ), F0(Fm0(if(flip(1/2), ϵ, Fm0(x)))))).
$x^{\vert x\vert}$	ab	7d	8	1 (1)	-17.2629	1	0.04	F0(x):=λx.pair(pair(pair(pair(ϵ, a), a), a), a).
$x^{\vert x\vert}$	ab	7d	8	10 (8)	-115.906	0.33333	0.4	F0(x):=λx.if(flip(1/4), Fm0(F0(pair(if(flip(1/2), pair(x, a), x), b))), append(if(flip(1/3), sample(if(flip(1/2), F0(x), Σ)), x), x)).
$x^{\vert x\vert}$	ab	7d	8	100 (37)	-2723.72	0.11111	0.24	F0(x):=λx.if(flip(7/24), F0(pair(x, b)), append(if(not(flip(if(empty(x), 1/3, 3/8))), append(x, append(sample(if(empty(x), (Σ∪pair(append(sample((ϵ∪Σ)), pair(pair(pair(ϵ, a), b), a)), b)), x)), if(flip(1/12), ϵ, append(x, x)))), F0(pair(x, a))), x)).
$x^{\vert x\vert}$	ab	7d	8	1000 (173)	-46751	0.19230	0.96	F0(x):=λx.if(flip(1/2), F0(if(flip(11/24), pair(x, a), pair(x, b))), append(if(empty(x), sample(if(flip(1/3), pair(pair(pair(pair(if(not(flip(5/12)), ϵ, sample(Σ)), a), b), a), b), Σ)), x), append(append(x, if(flip(1/24), if(flip(7/24), ϵ, x), append(append(x, x), x))), x))).
$x^{\vert x\vert}$	ab	7d	8	10000 (731)	-419279	0.19230	0.96	F0(x):=λx.if(flip(11/24), F0(if(flip(11/24), pair(x, a), pair(x, b))), append(if(empty(x), sample(if(flip(5/24), pair(pair(pair(if(flip(5/24), ϵ, sample(Σ)), b), a), b), Σ)), x), append(append(x, if(flip(1/24), if(flip(1/8), ϵ, x), append(append(x, x), x))), x))).
$x^{\vert x\vert}$	ab	7d	8	100000 (3193)	-4.15868e+06	0.19230	0.96	F0(x):=λx.if(flip(11/24), F0(if(flip(11/24), pair(x, a), pair(x, b))), append(if((ϵ==x), sample(if(flip(5/24), pair(pair(pair(if(flip(5/24), x, sample(Σ)), b), a), b), Σ)), x), append(append(x, if(flip(1/24), if(flip(1/8), ϵ, x), append(append(x, x), x))), x))).
$x^{\vert x\vert}$	ab	7d	8	1 (1)	-21.585	1	0.04	F0(x):=λx.append(x, x).<br>F1(x):=λx.Fm0(pair(pair(ϵ, a), a)).
$x^{\vert x\vert}$	ab	7d	8	10 (8)	-95.6085	0.32142	0.6	F0(x):=λx.insert(if(flip(11/24), F0(pair(ϵ, b)), x), sample(Σ)).<br>F1(x):=λx.append(if(flip(1/2), F1(ϵ), ϵ), Fm0(ϵ)).
$x^{\vert x\vert}$	ab	7d	8	100 (37)	-2465.77	0.2	0.64	F0(x):=λx.if(flip(5/24), ϵ, append(x, x)).<br>F1(x):=λx.sample(if(not(flip(5/12)), Fm1(append(x, sample(Σ))), append(if(empty(x), sample(if(flip(1/4), pair(pair(pair(pair(if(flip(7/24), ϵ, sample(Σ)), a), b), a), b), Σ)), F0(x)), append(append(x, append(x, x)), x)))).
$x^{\vert x\vert}$	ab	7d	8	1000 (173)	-47983.9	0.10526	0.52	F0(x):=λx.F1(x).<br>F1(x):=λx.if(flip(3/8), append(sample(if(flip(1/12), if((head(x)==x), (Σ\x), ϵ), append(x, if((x==head(x)), sample((Σ∪(ϵ\x))), append(x, x))))), append(append(if((x==pair(head(x), b)), ϵ, x), x), x)), F0(append(sample(Σ), x))).
$x^{\vert x\vert}$	ab	7d	8	10000 (731)	-426506	0.11428	0.52	F0(x):=λx.F1(x).<br>F1(x):=λx.if(flip(3/8), append(sample(if(flip(1/12), ϵ, append(x, if((x==head(x)), sample(if((ϵ==x), Σ, ϵ)), append(x, x))))), append(append(if((x==pair(head(x), a)), ϵ, x), x), x)), F0(append(sample(Σ), x))).
$x^{\vert x\vert}$	ab	7d	8	100000 (3193)	-4.10209e+06	0.12820	0.96	F0(x):=λx.sample((x\ϵ)).<br>F1(x):=λx.Fm0(if(flip(11/24), F1(append(sample(Σ), x)), append(if(flip(1/24), if(not(flip(7/24)), x, tail(sample(Σ))), append(x, append(x, if(empty(x), sample((Σ∪pair(pair(pair(pair(if(flip(11/24), pair(x, b), if(flip(5/24), pair(x, a), x)), a), b), a), b))), x)))), append(append(x, x), x)))).
$x^{\vert x\vert}$	ab	7d	8	1 (1)	-29.0205	1	0.04	F0(x):=λx.append(x, x).<br>F1(x):=λx.ϵ.<br>F2(x):=λx.F0(pair(pair(Fm1(ϵ), a), a)).
$x^{\vert x\vert}$	ab	7d	8	10 (8)	-107.178	0.25	0.52	F0(x):=λx.pair(pair(Fm1(sample(Σ)), a), b).<br>F1(x):=λx.sample((Σ∪(x∪pair(x, b)))).<br>F2(x):=λx.append(Fm1(Fm0(ϵ)), if(flip(1/2), ϵ, F2(ϵ))).
$x^{\vert x\vert}$	ab	7d	8	100 (37)	-2718.68	0.05405	0.56	F0(x):=λx.pair(pair(pair(insert(if(flip(1/2), head(x), pair(x, a)), if(flip(5/12), sample(Σ), ϵ)), b), a), b).<br>F1(x):=λx.append(if(not(flip(3/8)), Fm1(sample(Σ)), ϵ), x).<br>F2(x):=λx.append(if((head(x)==x), Fm2(pair(x, a)), append(if(flip(1/8), if(flip(5/24), F0(pair(x, b)), ϵ), Fm1(ϵ)), Fm1(ϵ))), Fm1(ϵ)).
$x^{\vert x\vert}$	ab	7d	8	1000 (173)	-48952.3	0.16666	1	F0(x):=λx.if(empty(x), if(flip(1/2), sample(Σ), sample((Σ∪pair(pair(pair(pair(ϵ, b), a), b), a)))), append(x, append(x, x))).<br>F1(x):=λx.if(not(empty(x)), x, pair(pair(pair(pair(ϵ, a), b), a), b)).<br>F2(x):=λx.Fm1(if(flip(3/8), append(x, append(if(not(flip(1/12)), append(Fm0(x), x), if(flip(5/24), x, append(x, x))), x)), Fm2(append(sample(Σ), x)))).
$x^{\vert x\vert}$	ab	7d	8	10000 (731)	-410987	0.11111	1	F0(x):=λx.append(append(x, x), if(or((head(x)==x), flip(1/24)), if(flip(5/24), ϵ, append(if(flip(1/2), x, if(flip(11/24), ϵ, if((Fm1(ϵ)==x), x, ϵ))), x)), append(append(x, x), append(x, x)))).<br>F1(x):=λx.append(sample(Σ), if(flip(11/24), Fm1(ϵ), ϵ)).<br>F2(x):=λx.if(or(flip(11/24), flip(1/24)), sample(Σ), F0(Fm1(ϵ))).

$x^{\vert x\vert}$	ab	7d	8	100000 (3193)	-4.13288e+06	0.11111	1	F0(x):=λx.append(append(x, x), if(or((head(x)==x), flip(1/24)), if(flip(5/24), ε, append(if(flip(1/2), x, if(flip(11/24), ε, if((Fm1(ε)==x), x, tail(x)))), x)), append(append(x, x), append(x, x))))).<br>F1(x):=λx.append(sample(Σ), if(flip(11/24), Fm1(ε), ε)).<br>F2(x):=λx.if(or(flip(11/24), flip(1/24)), sample(Σ), F0(Fm1(x))).
$x^{\vert x\vert}$	ab	7d	8	1 (1)	-36.9137	1	0.04	F0(x):=λx.pair(pair(ε, a), a).<br>F1(x):=λx.append(x, x).<br>F2(x):=λx.Fm1(Fm0(ε)).<br>F3(x):=λx.Fm2(ε).
$x^{\vert x\vert}$	ab	7d	8	10 (8)	-118.736	0.24	0.44	F0(x):=λx.ε.<br>F1(x):=λx.if(not(flip(1/4)), pair(append(x, Fm2(x)), b), sample(Σ)).<br>F2(x):=λx.sample((x∪Σ)).<br>F3(x):=λx.append(F1(Fm1(ε)), if(flip(1/2), Fm3(F0(ε)), ε)).
$x^{\vert x\vert}$	ab	7d	8	100 (37)	-2657.44	0.10526	0.6	F0(x):=λx.append(if(flip(1/2), F2(ε), ε), sample(Σ)).<br>F1(x):=λx.append(append(append(x, x), append(x, x)), if(flip(1/6), ε, append(x, x))).<br>F2(x):=λx.Fm0(ε).<br>F3(x):=λx.if(flip(11/24), Fm1(F0(ε)), sample(Σ)).
$x^{\vert x\vert}$	ab	7d	8	1000 (173)	-47916.1	0.22222	0.68	F0(x):=λx.append(append(x, if(not((head(x)==x)), append(x, x), sample(if(and(not((ε==x)), flip(1/8)), Fm1(pair(pair(ε, a), b)), ((ε∪Σ)\x))))), F2(x)).<br>F1(x):=λx.append(if(flip(5/24), if(or(flip(1/2), flip(3/8)), x, append(pair(ε, a), x)), ε), pair(pair(pair(pair(pair(ε, b), b), b), b), b)).<br>F2(x):=λx.if(flip(1/24), if(empty(x), append(pair(pair(ε, b), a), pair(ε, b)), ε), append(x, x)).<br>F3(x):=λx.if(flip(11/24), append(F0(x), x), Fm3(if(flip(11/24), pair(x, a), pair(x, b)))).
$x^{\vert x\vert}$	ab	7d	8	10000 (731)	-425299	0.11111	0.92	F0(x):=λx.Fm2(x).<br>F1(x):=λx.sample(Σ).<br>F2(x):=λx.if(flip(11/24), Fm0(append(x, sample(Σ))), append(append(x, append(if(flip(1/24), sample(if(flip(1/8), if((head(x)==x), x, ε), if(or(flip(7/24), flip(5/24)), ε, x))), append(append(x, x), x)), x)), x)).<br>F3(x):=λx.if(or(or(flip(1/4), flip(1/8)), flip(1/12)), sample(Σ), F0(F1(ε))).
$x^{\vert x\vert}$	ab	7d	8	100000 (3193)	-4.13479e+06	0.16216	0.56	F0(x):=λx.F2(append(append(if((x==head(x)), if((ε==x), if(flip(5/12), pair(if(flip(1/4), pair(ε, a), ε), b), pair(if(flip(1/2), pair(tail(if(empty(x), x, x)), b), tail(F1(ε))), a)), ε), x), x), x)).<br>F1(x):=λx.ε.<br>F2(x):=λx.append(x, if(not(or((x==head(x)), flip(1/12))), x, ε)).<br>F3(x):=λx.if(not(flip(3/8)), F3(append(sample(Σ), x)), F0(x)).
a^{n^2}	ab	2d	8	1 (1)	-5.95153	1	0.05	F0(x):=λx.pair(ε, a).
a^{n^2}	ab	2d	8	10 (3)	-43.4122	0.95238	1	F0(x):=λx.append(if(flip(1/2), F0(pair(pair(x, a), a)), ε), pair(x, a)).
a^{n^2}	ab	2d	8	100 (8)	-177.608	0.95238	1	F0(x):=λx.pair(append(if(flip(1/2), Fm0(pair(pair(x, a), a)), ε), x), a).
a^{n^2}	ab	2d	8	1000 (10)	-1416.98	0.95238	1	F0(x):=λx.pair(append(if(flip(1/2), Fm0(pair(pair(x, a), a)), ε), x), a).
a^{n^2}	ab	2d	8	10000 (13)	-14187.1	0.95238	1	F0(x):=λx.pair(append(if(flip(1/2), Fm0(pair(pair(x, a), a)), ε), x), a).
a^{n^2}	ab	2d	8	100000 (16)	-141087	0.95238	1	F0(x):=λx.append(pair(if(flip(1/2), F0(pair(x, a)), ε), a), append(x, x)).
a^{n^2}	ab	2d	8	1 (1)	-12.5762	1	0.05	F0(x):=λx.pair(ε, a).<br>F1(x):=λx.F0(ε).
a^{n^2}	ab	2d	8	10 (3)	-47.6527	0.95238	1	F0(x):=λx.append(x, if(flip(1/2), Fm1(pair(x, a)), ε)).<br>F1(x):=λx.Fm0(pair(x, a)).
a^{n^2}	ab	2d	8	100 (8)	-181.848	0.95238	1	F0(x):=λx.append(x, if(flip(1/2), ε, Fm1(pair(x, a)))).<br>F1(x):=λx.F0(pair(x, a)).
a^{n^2}	ab	2d	8	1000 (10)	-1421.22	0.95238	1	F0(x):=λx.append(x, if(flip(1/2), ε, Fm1(pair(x, a)))).<br>F1(x):=λx.F0(pair(x, a)).
a^{n^2}	ab	2d	8	10000 (13)	-14191.3	0.95238	1	F0(x):=λx.append(x, if(flip(1/2), ε, Fm1(pair(x, a)))).<br>F1(x):=λx.F0(pair(x, a)).
a^{n^2}	ab	2d	8	100000 (16)	-141091	0.95238	1	F0(x):=λx.append(x, if(flip(1/2), ε, Fm1(pair(x, a)))).<br>F1(x):=λx.F0(pair(x, a)).
a^{n^2}	ab	2d	8	1 (1)	-20.0117	1	0.05	F0(x):=λx.pair(Fm1(ε), a).<br>F1(x):=λx.ε.<br>F2(x):=λx.F0(ε).
a^{n^2}	ab	2d	8	10 (3)	-55.4937	0.95238	1	F0(x):=λx.ε.<br>F1(x):=λx.append(x, if(flip(1/2), F0(ε), F2(pair(x, a)))).<br>F2(x):=λx.F1(pair(x, a)).
a^{n^2}	ab	2d	8	100 (8)	-189.689	0.95238	1	F0(x):=λx.ε.<br>F1(x):=λx.append(if(flip(1/2), F0(ε), F2(pair(x, a))), x).<br>F2(x):=λx.F1(pair(x, a)).

a^{n^2}	ab	2d	8	1000 (10)	-1429.06	0.95238	1	F0(x):=λx.ϵ.<br>F1(x):=λx.append(if(flip(1/2), F0(ϵ), F2(pair(x, a))), x).<br>F2(x):=λx.F1(pair(x, a)).
a^{n^2}	ab	2d	8	10000 (13)	-14199.1	0.95238	1	F0(x):=λx.ϵ.<br>F1(x):=λx.append(if(flip(1/2), F0(ϵ), F2(pair(x, a))), x).<br>F2(x):=λx.F1(pair(x, a)).
a^{n^2}	ab	2d	8	100000 (16)	-141099	0.95238	1	F0(x):=λx.append(if(flip(1/2), ϵ, Fm1(pair(x, a))), x).<br>F1(x):=λx.F2(x).<br>F2(x):=λx.Fm0(pair(x, a)).
a^{n^2}	ab	2d	8	1 (1)	-27.9049	1	0.05	F0(x):=λx.ϵ.<br>F1(x):=λx.F2(ϵ).<br>F2(x):=λx.Fm0(ϵ).<br>F3(x):=λx.pair(F1(ϵ), a).
a^{n^2}	ab	2d	8	10 (3)	-63.6745	0.95238	1	F0(x):=λx.ϵ.<br>F1(x):=λx.append(if(flip(1/2), Fm3(pair(x, a)), F2(ϵ)), x).<br>F2(x):=λx.F0(ϵ).<br>F3(x):=λx.F1(pair(x, a)).
a^{n^2}	ab	2d	8	100 (8)	-197.87	0.95238	1	F0(x):=λx.Fm2(pair(x, a)).<br>F1(x):=λx.ϵ.<br>F2(x):=λx.append(if(flip(1/2), F0(pair(x, a)), F1(ϵ)), x).<br>F3(x):=λx.Fm0(ϵ).
a^{n^2}	ab	2d	8	1000 (10)	-1437.24	0.95238	1	F0(x):=λx.append(if(flip(1/2), Fm3(pair(x, a)), F1(F2(ϵ))), x).<br>F1(x):=λx.ϵ.<br>F2(x):=λx.ϵ.<br>F3(x):=λx.Fm0(pair(x, a)).
a^{n^2}	ab	2d	8	10000 (13)	-14207.3	0.95238	1	F0(x):=λx.append(if(flip(1/2), Fm3(pair(x, a)), F1(F2(ϵ))), x).<br>F1(x):=λx.ϵ.<br>F2(x):=λx.ϵ.<br>F3(x):=λx.Fm0(pair(x, a)).
a^{n^2}	ab	2d	8	100000 (16)	-141107	0.95238	1	F0(x):=λx.append(if(flip(1/2), Fm3(pair(x, a)), F1(F2(ϵ))), x).<br>F1(x):=λx.ϵ.<br>F2(x):=λx.ϵ.<br>F3(x):=λx.Fm0(pair(x, a)).
$a^nb^mc^nd^m$	abcd	4d	8	1 (1)	-28.9627	1	0.04	F0(x):=λx.pair(pair(pair(pair(pair(pair(ϵ, a), a), b), c), c), d).
$a^nb^mc^nd^m$	abcd	4d	8	10 (8)	-148.069	0.35714	0.48	F0(x):=λx.append(if(not(flip(5/24)), F0(pair(x, a)), append(pair(x, a), pair(if(flip(1/2), ϵ, pair(pair(if(flip(1/2), ϵ, pair(ϵ, b)), b), b)), b))), append(pair(ϵ, c), if(empty(x), pair(sample(if(flip(1/2), ϵ, pair(pair(ϵ, d), d))), d), ϵ))).
$a^nb^mc^nd^m$	abcd	4d	8	100 (37)	-2256.45	0	0	F0(x):=λx.pair(append(pair(ϵ, a), if(not(flip(3/8)), Fm0(ϵ), pair(sample((pair(sample((pair(if(flip(1/2), append(if(flip(1/2), ϵ, pair(ϵ, b)), pair(if(flip(1/2), pair(pair(if(flip(1/2), pair(pair(ϵ, b), b), ϵ), b), b), ϵ), b)), x), b) ∪ ϵ)), b) ∪ ϵ)), b))), c).
$a^nb^mc^nd^m$	abcd	4d	8	1000 (103)	-23922.2	0.04	0.08	F0(x):=λx.append(head(pair(x, a)), if(not(flip(if((x==ϵ), 1/24, 1/3))), F0(if(and((x==ϵ), flip(1/3)), pair(ϵ, b), x)), if(and(not(empty(x)), not(or(flip(1/6), flip(1/6)))), pair(pair(pair(pair(ϵ, c), c), c), c), pair(pair(pair(sample((pair(ϵ, b) ∪ pair(ϵ, c))), c), d), d)))).
$a^nb^mc^nd^m$	abcd	4d	8	10000 (203)	-230394	0	0	F0(x):=λx.append(pair(ϵ, a), if(flip(1/3), append(append(if(flip(7/24), x, ϵ), append(if(flip(5/24), pair(sample((ϵ ∪ append(append(pair(x, b), x), x))), b), ϵ), if(flip(3/8), pair(ϵ, b), ϵ))), pair(pair(if(flip(1/4), pair(pair(x, b), b), ϵ), b), c)), append(F0(pair(pair(ϵ, b), b)), pair(ϵ, c)))).
$a^nb^mc^nd^m$	abcd	4d	8	100000 (315)	-2.23492e+06	0	0	F0(x):=λx.if(and((x==ϵ), not(flip(1/4))), pair(append(pair(x, a), F0(x)), c), pair(sample(if(not(flip(1/3)), if(not(flip(3/8)), pair(Fm0(pair(x, b)), b), pair(ϵ, b)), ϵ)), b)).
$a^nb^mc^nd^m$	abcd	4d	8	1 (1)	-35.5874	1	0.04	F0(x):=λx.ϵ.<br>F1(x):=λx.pair(pair(pair(pair(pair(pair(Fm0(ϵ), a), a), b), c), c), d).
$a^nb^mc^nd^m$	abcd	4d	8	10 (8)	-150.852	0.38461	0.28	F0(x):=λx.append(pair(ϵ, a), if(not(flip(1/4)), pair(Fm0(x), c), if(flip(1/2), x, pair(pair(pair(if(flip(1/2), ϵ, x), b), b), b)))).<br>F1(x):=λx.pair(pair(F0(pair(ϵ, b)), c), d).
$a^nb^mc^nd^m$	abcd	4d	8	100 (37)	-443.341	0.92	1	F0(x):=λx.append(pair(ϵ, a), if(flip(3/8), x, Fm0(pair(x, c)))).<br>F1(x):=λx.if(flip(if(empty(x), 1/24, 1/3)), pair(F0(x), c), pair(F1(pair(x, b)), d)).
$a^nb^mc^nd^m$	abcd	4d	8	1000 (103)	-3996.36	0.96551	1	F0(x):=λx.append(pair(ϵ, a), if(flip(1/3), x, Fm0(pair(x, c)))).<br>F1(x):=λx.if(flip(if(empty(x), 1/24, 1/3)), pair(F0(x), c), pair(F1(pair(x, b)), d)).
$a^nb^mc^nd^m$	abcd	4d	8	10000 (203)	-44072.5	0.96551	1	F0(x):=λx.append(pair(ϵ, a), if(flip(1/3), x, Fm0(pair(x, c)))).<br>F1(x):=λx.if(flip(if(empty(x), 1/24, 1/3)), pair(F0(x), c), pair(F1(pair(x, b)), d)).
$a^nb^mc^nd^m$	abcd	4d	8	100000 (315)	-422840	0.96551	1	F0(x):=λx.append(pair(ϵ, a), if(flip(1/3), x, Fm0(pair(x, c)))).<br>F1(x):=λx.if(flip(if(empty(x), 1/24, 1/3)), pair(F0(x), c), pair(F1(pair(x, b)), d)).
$a^nb^mc^nd^m$	abcd	4d	8	1 (1)	-43.0229	1	0.04	F0(x):=λx.ϵ.<br>F1(x):=λx.pair(pair(pair(pair(pair(pair(F0(ϵ), a), a), b), c), c), d).<br>F2(x):=λx.Fm1(ϵ).

$a^n b^m c^n d^m$	abcd	4d	8	10 (8)	-139.768	0.36	0.56	$F0(x)$:=λx.append(x, if(not(empty(x)), pair(F0(if(flip(1/6), ϵ, x)), c), pair(if(flip(1/2), Fm0(ϵ), ϵ), b))).<br>$F1(x)$:=λx.if(flip(1/2), Fm0(pair(ϵ, a)), pair(Fm2(x), d)).<br>$F2(x)$:=λx.pair(Fm1(ϵ), d).
$a^n b^m c^n d^m$	abcd	4d	8	100 (37)	-454.591	0.92	1	$F0(x)$:=λx.append(pair(ϵ, a), sample(if(flip(3/8), x, F1(x)))).<br>$F1(x)$:=λx.pair(F0(x), c).<br>$F2(x)$:=λx.if(not(flip(if(empty(x), 1/24, 1/3))), pair(F2(pair(x, b)), d), F1(x)).
$a^n b^m c^n d^m$	abcd	4d	8	1000 (103)	-4007.61	0.96551	1	$F0(x)$:=λx.append(pair(ϵ, a), sample(if(flip(1/3), x, F1(x)))).<br>$F1(x)$:=λx.pair(F0(x), c).<br>$F2(x)$:=λx.if(not(flip(if(empty(x), 1/24, 1/3))), pair(F2(pair(x, b)), d), Fm1(x)).
$a^n b^m c^n d^m$	abcd	4d	8	10000 (203)	-43449.1	1	1	$F0(x)$:=λx.append(pair(ϵ, a), sample(if(flip(7/24), (x\ϵ), F1(x)))).<br>$F1(x)$:=λx.pair(Fm0(x), c).<br>$F2(x)$:=λx.if(not(flip(if((ϵ==x), 1/24, 1/3))), pair(F2(pair(x, b)), d), Fm1(x)).
$a^n b^m c^n d^m$	abcd	4d	8	100000 (315)	-410783	1	1	$F0(x)$:=λx.ϵ.<br>$F1(x)$:=λx.pair(append(x, if(flip(1/3), x, Fm1(x))), c).<br>$F2(x)$:=λx.insert(pair(if(flip(1/4), pair(Fm1(pair(ϵ, a)), c), if(and(flip(1/8), (ϵ==if(flip(1/3), x, F0(ϵ)))), pair(pair(ϵ, a), c), Fm2(ϵ))), d), pair(ϵ, b)).
$a^n b^m c^n d^m$	abcd	4d	8	1 (1)	-50.9161	1	0.04	$F0(x)$:=λx.F2(Fm1(ϵ)).<br>$F1(x)$:=λx.ϵ.<br>$F2(x)$:=λx.pair(pair(pair(pair(pair(ϵ, a), a), b), c), c).<br>$F3(x)$:=λx.pair(F0(ϵ), d).
$a^n b^m c^n d^m$	abcd	4d	8	10 (8)	-116.071	1	1	$F0(x)$:=λx.append(pair(F2(ϵ), a), x).<br>$F1(x)$:=λx.pair(Fm0(if(not(flip(1/4)), Fm1(x), pair(x, b))), c).<br>$F2(x)$:=λx.ϵ.<br>$F3(x)$:=λx.pair(if(flip(1/2), Fm3(pair(x, b)), F1(x)), d).
$a^n b^m c^n d^m$	abcd	4d	8	100 (37)	-451.035	1	1	$F0(x)$:=λx.pair(F1(pair(ϵ, b)), d).<br>$F1(x)$:=λx.if(flip(1/3), Fm2(x), F3(x)).<br>$F2(x)$:=λx.pair(append(pair(ϵ, a), if(not(flip(1/3)), Fm2(x), x)), c).<br>$F3(x)$:=λx.insert(F0(ϵ), x).
$a^n b^m c^n d^m$	abcd	4d	8	1000 (103)	-3965.07	1	1	$F0(x)$:=λx.pair(F1(pair(ϵ, b)), d).<br>$F1(x)$:=λx.if(flip(1/3), Fm2(x), F3(x)).<br>$F2(x)$:=λx.pair(append(pair(ϵ, a), if(not(flip(1/3)), Fm2(x), x)), c).<br>$F3(x)$:=λx.insert(F0(ϵ), x).
$a^n b^m c^n d^m$	abcd	4d	8	10000 (203)	-42889.6	1	1	$F0(x)$:=λx.pair(F1(pair(ϵ, b)), d).<br>$F1(x)$:=λx.if(flip(1/3), Fm2(x), F3(x)).<br>$F2(x)$:=λx.pair(append(pair(ϵ, a), if(not(flip(1/3)), Fm2(x), x)), c).<br>$F3(x)$:=λx.insert(F0(ϵ), x).
$a^n b^m c^n d^m$	abcd	4d	8	100000 (315)	-411666	1	1	$F0(x)$:=λx.pair(F1(ϵ), d).<br>$F1(x)$:=λx.F2(if(not(flip(1/3)), F1(ϵ), ϵ)).<br>$F2(x)$:=λx.pair(append(pair(ϵ, a), x), c).<br>$F3(x)$:=λx.insert(if(not(flip(1/3)), pair(F3(ϵ), d), F0(ϵ)), pair(ϵ, b)).
$a^n b^m a^n b^m$	abcd	1d	8	1 (1)	-40.9871	0.90476	0.16	$F0(x)$:=λx.pair(insert(if(flip(1/2), F0(pair(head(x), a)), x), pair(ϵ, b)), b).
$a^n b^m a^n b^m$	abcd	1d	8	10 (8)	-124.236	0.56	0.92	$F0(x)$:=λx.if(flip(1/2), append(x, x), F0(insert(x, append(if(or(empty(x), flip(1/4)), pair(head(x), a), ϵ), if(flip(1/6), if(flip(1/2), head(x), x), pair(ϵ, b)))))).
$a^n b^m a^n b^m$	abcd	1d	8	100 (40)	-1266.67	0.72	1	$F0(x)$:=λx.sample(if(not(flip(11/24)), if(empty(x), pair(insert(F0(ϵ), pair(ϵ, b)), b), ϵ), pair(append(pair(Fm0(pair(ϵ, b)), a), Fm0(pair(ϵ, d))), a))).
$a^n b^m a^n b^m$	abcd	1d	8	1000 (108)	-11168.6	0.72	1	$F0(x)$:=λx.sample(if(not(flip(11/24)), if(empty(x), pair(insert(F0(ϵ), pair(ϵ, b)), b), ϵ), pair(append(pair(Fm0(pair(ϵ, b)), a), Fm0(pair(ϵ, d))), a))).
$a^n b^m a^n b^m$	abcd	1d	8	10000 (197)	-98038.3	0.72	1	$F0(x)$:=λx.sample(if(not(flip(11/24)), if(empty(x), pair(insert(F0(ϵ), pair(ϵ, b)), b), ϵ), pair(append(pair(Fm0(pair(ϵ, b)), a), Fm0(pair(ϵ, d))), a))).
$a^n b^m a^n b^m$	abcd	1d	8	100000 (338)	-1.00574e+06	0.72	1	$F0(x)$:=λx.sample(if(not(flip(11/24)), if((x==ϵ), pair(insert(Fm0(ϵ), pair(ϵ, b)), b), ϵ), pair(append(pair(Fm0(pair(ϵ, c)), a), Fm0(pair(ϵ, b))), a))).
$a^n b^m a^n b^m$	abcd	1d	8	1 (1)	-42.0034	1	0.24	$F0(x)$:=λx.pair(if(flip(1/8), pair(ϵ, a), F0(ϵ)), b).<br>$F1(x)$:=λx.append(Fm0(ϵ), Fm0(ϵ)).
$a^n b^m a^n b^m$	abcd	1d	8	10 (8)	-95.3823	0.4	1	$F0(x)$:=λx.pair(if(flip(1/6), ϵ, Fm0(ϵ)), a).<br>$F1(x)$:=λx.pair(insert(if(flip(1/3), F0(ϵ), Fm1(ϵ)), pair(ϵ, b)), b).
$a^n b^m a^n b^m$	abcd	1d	8	100 (40)	-739.719	0.4	1	$F0(x)$:=λx.pair(if(flip(1/6), ϵ, Fm0(ϵ)), a).<br>$F1(x)$:=λx.pair(insert(if(flip(1/3), F0(ϵ), Fm1(ϵ)), pair(ϵ, b)), b).
$a^n b^m a^n b^m$	abcd	1d	8	1000 (108)	-4282.89	1	1	$F0(x)$:=λx.insert(pair(Fm1(if(flip(1/3), x, ϵ)), b), x).<br>$F1(x)$:=λx.if(not(empty(x)), pair(pair(if(not(flip(3/8)), Fm1(x), ϵ), a), a), F0(pair(ϵ, b))).
$a^n b^m a^n b^m$	abcd	1d	8	10000 (197)	-41766.9	1	1	$F0(x)$:=λx.insert(pair(Fm1(if(flip(1/3), x, ϵ)), b), x).<br>$F1(x)$:=λx.if(not(empty(x)), pair(pair(if(not(flip(1/3)), F1(x), ϵ), a), a), F0(pair(ϵ, b))).

$a^nb^ma^nb^m$	abcd	1d	8	100000 (338)	-419229	1	1	$F0(x)$:=λx.insert(pair(Fm1(if(flip(1/3), x, ε)), b), x).<br>$F1(x)$:=λx.if(not(empty(x)), pair(pair(if(not(flip(1/3)), F1(x), ε), a), a), F0(pair(ε, b))).
$a^nb^ma^nb^m$	abcd	1d	8	1 (1)	-46.5801	0.25	0.24	$F0(x)$:=λx.append(x, x).<br>$F1(x)$:=λx.pair(if(flip(1/8), sample(Σ), F1(ε)), b).<br>$F2(x)$:=λx.F0(F1(ε)).
$a^nb^ma^nb^m$	abcd	1d	8	10 (8)	-103.208	1	1	$F0(x)$:=λx.if(flip(1/2), Fm1(pair(ε, a)), pair(F0(ε), b)).<br>$F1(x)$:=λx.if(flip(1/2), F1(pair(x, a)), pair(x, b)).<br>$F2(x)$:=λx.append(Fm0(ε), Fm0(ε)).
$a^nb^ma^nb^m$	abcd	1d	8	100 (40)	-459.073	1	1	$F0(x)$:=λx.if(flip(1/3), pair(ε, a), pair(Fm0(ε), a)).<br>$F1(x)$:=λx.if(not(flip(1/3)), pair(F1(ε), b), Fm0(ε)).<br>$F2(x)$:=λx.append(pair(Fm1(ε), b), pair(Fm1(ε), b)).
$a^nb^ma^nb^m$	abcd	1d	8	1000 (108)	-4286.73	1	1	$F0(x)$:=λx.if(flip(3/8), pair(ε, a), pair(F0(ε), a)).<br>$F1(x)$:=λx.if(not(flip(1/3)), pair(F1(ε), b), F0(ε)).<br>$F2(x)$:=λx.append(pair(Fm1(ε), b), pair(Fm1(ε), b)).
$a^nb^ma^nb^m$	abcd	1d	8	10000 (197)	-41524.7	1	1	$F0(x)$:=λx.pair(append(x, if(flip(1/3), ε, F0(ε))), b).<br>$F1(x)$:=λx.if(and(not(and(empty(x), flip(1/3))), empty(x)), pair(F1(ε), a), append(x, x)).<br>$F2(x)$:=λx.Fm1(Fm0(pair(Fm1(ε), a))).
$a^nb^ma^nb^m$	abcd	1d	8	100000 (338)	-419245	1	1	$F0(x)$:=λx.pair(append(x, if(flip(1/3), Fm1(ε), F0(ε))), b).<br>$F1(x)$:=λx.if(and(not(and(empty(x), flip(1/3))), (ε==x)), pair(Fm1(ε), a), append(x, x)).<br>$F2(x)$:=λx.Fm1(Fm0(pair(x, a))).
$a^nb^ma^nb^m$	abcd	1d	8	1 (1)	-54.2548	1	0.24	$F0(x)$:=λx.pair(if(flip(1/8), pair(ε, a), F0(ε)), b).<br>$F1(x)$:=λx.F2(Fm0(ε)).<br>$F2(x)$:=λx.append(x, x).<br>$F3(x)$:=λx.Fm1(ε).
$a^nb^ma^nb^m$	abcd	1d	8	10 (8)	-98.573	1	1	$F0(x)$:=λx.F1(pair(x, b)).<br>$F1(x)$:=λx.if(flip(1/2), append(x, x), Fm0(x)).<br>$F2(x)$:=λx.pair(if(flip(1/2), ε, F2(ε)), a).<br>$F3(x)$:=λx.Fm0(Fm2(ε)).
$a^nb^ma^nb^m$	abcd	1d	8	100 (40)	-449.346	1	1	$F0(x)$:=λx.F1(pair(x, b)).<br>$F1(x)$:=λx.if(flip(1/3), append(x, x), Fm0(x)).<br>$F2(x)$:=λx.pair(if(flip(1/3), x, F2(ε)), a).<br>$F3(x)$:=λx.Fm0(Fm2(ε)).
$a^nb^ma^nb^m$	abcd	1d	8	1000 (108)	-4276.31	1	1	$F0(x)$:=λx.F1(pair(x, b)).<br>$F1(x)$:=λx.if(flip(1/3), append(x, x), Fm0(x)).<br>$F2(x)$:=λx.pair(if(flip(3/8), ε, F2(ε)), a).<br>$F3(x)$:=λx.Fm0(Fm2(ε)).
$a^nb^ma^nb^m$	abcd	1d	8	10000 (197)	-41541	1	1	$F0(x)$:=λx.append(if(not(flip(1/3)), Fm0(x), pair(ε, a)), pair(ε, b)).<br>$F1(x)$:=λx.if(not(flip(1/3)), append(head(x), Fm1(x)), x).<br>$F2(x)$:=λx.append(x, x).<br>$F3(x)$:=λx.Fm2(Fm1(insert(if(flip(1/12), ε, ε), F0(ε)))).
$a^nb^ma^nb^m$	abcd	1d	8	100000 (338)	-419815	1	1	$F0(x)$:=λx.append(if(not(flip(1/3)), Fm0(ε), pair(ε, a)), pair(ε, b)).<br>$F1(x)$:=λx.if(not(flip(1/3)), append(pair(ε, a), Fm1(ε)), Fm0(ε)).<br>$F2(x)$:=λx.append(x, x).<br>$F3(x)$:=λx.Fm2(Fm1(if(flip(1/12), ε, ε))).
$a^nb^ma^nb^mccc$	abcd	5d	8	1 (1)	-37.9098	0.04761	0.04	$F0(x)$:=λx.pair(if(flip(1/2), x, Fm0(pair(pair(pair(pair(x, a), a), b), b))), c).
$a^nb^ma^nb^mccc$	abcd	5d	8	10 (9)	-291.629	0	0	$F0(x)$:=λx.sample(if(flip(1/2), if(empty(x), pair(insert(Fm0(ε), pair(ε, b)), b), x), append(x, F0(pair(pair(ε, a), a))))).
$a^nb^ma^nb^mccc$	abcd	5d	8	100 (35)	-1552.68	0	0	$F0(x)$:=λx.pair(sample(if(flip(3/8), if(flip(5/24), append(append(append(x, Fm0(x)), x), pair(pair(pair(Fm0(x), c), c), c)), if(empty(if(flip(11/24), ε, x)), pair(sample((if((head(x)==x), x, ε) ∪ pair(head(x), a))), a), x)), Fm0(pair(x, a)))), b).
$a^nb^ma^nb^mccc$	abcd	5d	8	1000 (114)	-24268.1	0.84	0.88	$F0(x)$:=λx.sample(if(or((x==head(x)), flip(3/8)), Fm0(append(append(pair(ε, a), x), if(flip(7/24), pair(if(not(flip(1/4)), pair(if(flip(5/12), pair(if(flip(5/12), ε, pair(append(x, x), b)), b), ε), b), append(head(x), append(x, x))), b), ε))), append(x, pair(pair(pair(x, c), c), c)))).
$a^nb^ma^nb^mccc$	abcd	5d	8	10000 (196)	-70536.7	0.23076	1	$F0(x)$:=λx.if(flip(1/6), if(flip(1/8), pair(append(pair(insert(Fm0(pair(ε, a)), pair(x, b)), b), pair(pair(x, c), c)), c), append(x, x)), F0(append(head(pair(append(append(sample(x), append(x, x)), append(append(x, pair(append(x, x), b)), x)), a)), if((sample(ε)==if(empty(ε), ε, ε)), x, ε)))).
$a^nb^ma^nb^mccc$	abcd	5d	8	100000 (337)	-710737	0.23076	1	$F0(x)$:=λx.if(flip(1/6), if(flip(1/8), pair(append(pair(insert(Fm0(pair(ε, a)), pair(x, b)), b), pair(pair(x, c), c)), c), append(x, x)), F0(append(head(pair(append(append(sample(x), append(x, x)), append(append(x, pair(append(x, x), b)), x)), b)), if((sample(ε)==if(empty(ε), ε, x)), x, ε)))).
$a^nb^ma^nb^mccc$	abcd	5d	8	1 (1)	-45.2893	1	0.04	$F0(x)$:=λx.pair(append(pair(pair(pair(pair(ε, a), a), b), b), x), c).<br>$F1(x)$:=λx.F0(pair(Fm0(ε), c)).
$a^nb^ma^nb^mccc$	abcd	5d	8	10 (9)	-189.618	0.04	0.28	$F0(x)$:=λx.append(x, if(flip(7/24), pair(pair(pair(ε, b), b), b), if(flip(1/2), pair(pair(pair(x, c), c), c), ε))).<br>$F1(x)$:=λx.Fm0(sample(if(not(flip(1/3)), Fm1(pair(x, a)), pair(Fm0(Fm0(pair(x, b))), b)))).
$a^nb^ma^nb^mccc$	abcd	5d	8	100 (35)	-916.262	0.48	0.8	$F0(x)$:=λx.if(flip(1/2), append(x, if(empty(x), pair(ε, a), pair(pair(pair(x, c), c), c))), pair(Fm0(if(empty(x), ε, pair(pair(pair(pair(x, b), b), b), b))), a)).<br>$F1(x)$:=λx.Fm0(append(F0(ε), sample((pair(ε, b) ∪ pair(pair(if(flip(3/8), ε, pair(if(not(flip(7/24)), ε, pair(ε, b)), b)), b), b))))).

$a^nb^ma^nb^mccc$	abcd	5d	8	1000 (114)	-4367.98	1	1	$F0(x)$:=λx.append(pair(ϵ, b), if(flip(1/3), x, Fm0(pair(x, b)))).<br>$F1(x)$:=λx.if((ϵ==if(flip(7/24), x, ϵ)), append(head(x), F1(append(x, pair(ϵ, a)))), pair(append(pair(ϵ, a), pair(pair(pair(sample(if((x==ϵ), if(flip(1/2), if(flip(1/2), ϵ, ϵ), if(flip(1/2), ϵ, pair(ϵ, d))), F0(x))), b), c), c)), c)).
$a^nb^ma^nb^mccc$	abcd	5d	8	10000 (196)	-42210.2	1	1	$F0(x)$:=λx.if(flip(1/3), append(pair(pair(ϵ, a), b), if((x==pair(ϵ, a)), Fm0(pair(ϵ, b)), pair(pair(ϵ, c), c))), append(x, insert(Fm0(x), x))).<br>$F1(x)$:=λx.pair(F0(pair(ϵ, a)), c).
$a^nb^ma^nb^mccc$	abcd	5d	8	100000 (337)	-421103	1	1	$F0(x)$:=λx.if(flip(1/3), append(pair(pair(ϵ, a), b), if((x==pair(ϵ, a)), Fm0(pair(ϵ, b)), pair(pair(ϵ, c), c))), append(x, insert(Fm0(x), x))).<br>$F1(x)$:=λx.pair(F0(pair(x, a)), c).
$a^nb^ma^nb^mccc$	abcd	5d	8	1 (1)	-50.6454	1	0.04	$F0(x)$:=λx.pair(append(x, pair(pair(x, c), c)), c).<br>$F1(x)$:=λx.Fm0(insert(x, x)).<br>$F2(x)$:=λx.F1(pair(pair(ϵ, a), b)).
$a^nb^ma^nb^mccc$	abcd	5d	8	10 (9)	-114.655	1	1	$F0(x)$:=λx.pair(if(flip(7/24), F1(ϵ), F0(ϵ)), b).<br>$F1(x)$:=λx.pair(if(flip(7/24), ϵ, F1(ϵ)), a).<br>$F2(x)$:=λx.append(Fm0(ϵ), pair(pair(pair(Fm0(ϵ), c), c), c)).
$a^nb^ma^nb^mccc$	abcd	5d	8	100 (35)	-445.597	1	1	$F0(x)$:=λx.pair(if(flip(1/3), F1(ϵ), F0(ϵ)), b).<br>$F1(x)$:=λx.pair(if(flip(3/8), ϵ, F1(ϵ)), a).<br>$F2(x)$:=λx.append(Fm0(ϵ), pair(pair(pair(Fm0(ϵ), c), c), c)).
$a^nb^ma^nb^mccc$	abcd	5d	8	1000 (114)	-4282.32	1	1	$F0(x)$:=λx.pair(if(not(flip(1/3)), Fm0(ϵ), ϵ), a).<br>$F1(x)$:=λx.pair(if(flip(1/3), Fm0(ϵ), F1(ϵ)), b).<br>$F2(x)$:=λx.pair(append(Fm1(ϵ), pair(pair(Fm1(ϵ), c), c)), c).
$a^nb^ma^nb^mccc$	abcd	5d	8	10000 (196)	-41937.9	1	1	$F0(x)$:=λx.pair(append(x, append(pair(ϵ, b), if(not(flip(1/3)), F0(ϵ), Fm1(ϵ)))), b).<br>$F1(x)$:=λx.pair(sample(if(flip(1/24), ϵ, if(flip(7/24), ϵ, F1(ϵ)))), a).<br>$F2(x)$:=λx.pair(pair(pair(Fm0(Fm1(ϵ)), c), c), c).
$a^nb^ma^nb^mccc$	abcd	5d	8	100000 (337)	-422214	1	1	$F0(x)$:=λx.pair(if(flip(1/3), ϵ, Fm0(ϵ)), a).<br>$F1(x)$:=λx.if(not(flip(7/24)), pair(pair(append(x, pair(x, c)), c), c), F1(pair(pair(pair(x, b), b), b))).<br>$F2(x)$:=λx.F1(append(pair(Fm0(ϵ), b), sample((pair(if(flip(3/8), pair(if(flip(1/24), ϵ, ϵ), b), ϵ), b)∪ϵ)))).
$a^nb^ma^nb^mccc$	abcd	5d	8	1 (1)	-58.5386	1	0.04	$F0(x)$:=λx.append(x, pair(pair(pair(x, c), c), c)).<br>$F1(x)$:=λx.Fm2(pair(pair(ϵ, a), b)).<br>$F2(x)$:=λx.insert(x, x).<br>$F3(x)$:=λx.F0(Fm1(ϵ)).
$a^nb^ma^nb^mccc$	abcd	5d	8	10 (9)	-121.559	1	1	$F0(x)$:=λx.Fm2(pair(x, b)).<br>$F1(x)$:=λx.append(Fm2(pair(ϵ, a)), Fm0(ϵ)).<br>$F2(x)$:=λx.if(flip(7/24), x, F1(ϵ)).<br>$F3(x)$:=λx.pair(pair(append(Fm1(ϵ), pair(Fm1(ϵ), c)), c), c).
$a^nb^ma^nb^mccc$	abcd	5d	8	100 (35)	-452.597	1	1	$F0(x)$:=λx.pair(if(not(flip(1/3)), F0(ϵ), F1(ϵ)), b).<br>$F1(x)$:=λx.pair(if(flip(3/8), ϵ, Fm1(ϵ)), a).<br>$F2(x)$:=λx.append(x, pair(pair(pair(x, c), c), c)).<br>$F3(x)$:=λx.F2(Fm0(ϵ)).
$a^nb^ma^nb^mccc$	abcd	5d	8	1000 (114)	-4013.49	1	1	$F0(x)$:=λx.if(not(flip(1/3)), pair(F0(pair(x, a)), b), if(and(flip(1/24), flip(1/3)), append(x, x), pair(ϵ, b))).<br>$F1(x)$:=λx.insert(pair(pair(ϵ, a), a), if(flip(1/3), Fm0(ϵ), Fm1(ϵ))).<br>$F2(x)$:=λx.Fm0(ϵ).<br>$F3(x)$:=λx.append(Fm1(ϵ), pair(pair(pair(F2(ϵ), c), c), c)).
$a^nb^ma^nb^mccc$	abcd	5d	8	10000 (196)	-41132	1	1	$F0(x)$:=λx.if(flip(7/24), insert(append(x, x), pair(ϵ, b)), F0(append(head(x), x))).<br>$F1(x)$:=λx.ϵ.<br>$F2(x)$:=λx.sample(if(not(flip(7/24)), (pair(Fm2(F1(ϵ)), b)\(Σ∪if(not((append(ϵ, x)==ϵ)), insert(ϵ, if(flip(1/2), ϵ, sample(if(flip(1/2), x, ϵ)))), x))), pair(ϵ, a))).<br>$F3(x)$:=λx.pair(pair(insert(pair(ϵ, c), pair(Fm0(Fm2(if(and(flip(1/24), flip(5/12)), ϵ, x))), b)), c), c).
$a^nb^ma^nb^mccc$	abcd	5d	8	100000 (337)	-419455	1	1	$F0(x)$:=λx.if(flip(1/3), pair(ϵ, a), pair(F0(ϵ), a)).<br>$F1(x)$:=λx.if(not(flip(1/3)), append(Fm1(ϵ), pair(ϵ, b)), pair(Fm0(if(flip(1/24), ϵ, ϵ)), b)).<br>$F2(x)$:=λx.append(x, pair(pair(pair(x, c), c), c)).<br>$F3(x)$:=λx.F2(F1(ϵ)).
$a^nb^nc^n$	abcd	4h	8	1 (1)	-15.5719	1	0.04	$F0(x)$:=λx.pair(pair(pair(ϵ, a), b), c).
$a^nb^nc^n$	abcd	4h	8	10 (6)	-56.9152	1	1	$F0(x)$:=λx.append(pair(ϵ, a), pair(if(flip(1/3), pair(x, b), Fm0(pair(x, b))), c)).
$a^nb^nc^n$	abcd	4h	8	100 (10)	-221.499	1	1	$F0(x)$:=λx.append(pair(ϵ, a), pair(if(flip(3/8), pair(x, b), Fm0(pair(x, b))), c)).
$a^nb^nc^n$	abcd	4h	8	1000 (19)	-1980.23	1	1	$F0(x)$:=λx.pair(append(pair(ϵ, a), insert(if(flip(1/3), ϵ, F0(ϵ)), pair(ϵ, b))), c).
$a^nb^nc^n$	abcd	4h	8	10000 (24)	-19227.2	1	1	$F0(x)$:=λx.pair(append(pair(ϵ, a), insert(if(flip(1/3), ϵ, F0(ϵ)), pair(ϵ, b))), c).
$a^nb^nc^n$	abcd	4h	8	100000 (28)	-193351	1	1	$F0(x)$:=λx.append(pair(ϵ, a), pair(if(flip(1/3), pair(x, b), Fm0(pair(x, b))), c)).
$a^nb^nc^n$	abcd	4h	8	1 (1)	-22.1965	1	0.04	$F0(x)$:=λx.ϵ.<br>$F1(x)$:=λx.pair(pair(pair(F0(ϵ), a), b), c).
$a^nb^nc^n$	abcd	4h	8	10 (6)	-63.6249	0.96	1	$F0(x)$:=λx.Fm1(pair(x, b)).<br>$F1(x)$:=λx.if(flip(1/4), x, append(pair(ϵ, a), pair(Fm0(x), c))).
$a^nb^nc^n$	abcd	4h	8	100 (10)	-228.59	1	1	$F0(x)$:=λx.ϵ.<br>$F1(x)$:=λx.append(pair(ϵ, a), insert(pair(if(flip(1/3), ϵ, F1(ϵ)), c), pair(Fm0(ϵ), b))).

$a^nb^nc^n$	abcd	4h	8	1000 (19)	-1987.55	1	1	$F0(x)$:=λx.ϵ.<br>$F1(x)$:=λx.append(pair(ϵ, a), insert(pair(if(flip(1/3), ϵ, F1(ϵ)), c), pair(Fm0(ϵ), b))).
$a^nb^nc^n$	abcd	4h	8	10000 (24)	-19233.8	1	1	$F0(x)$:=λx.pair(if(not(flip(1/3)), F1(x), x), c).<br>$F1(x)$:=λx.append(pair(ϵ, a), Fm0(pair(x, b))).
$a^nb^nc^n$	abcd	4h	8	100000 (28)	-193358	1	1	$F0(x)$:=λx.ϵ.<br>$F1(x)$:=λx.append(pair(ϵ, a), insert(pair(if(flip(1/3), ϵ, F1(ϵ)), c), pair(Fm0(ϵ), b))).
$a^nb^nc^n$	abcd	4h	8	1 (1)	-29.6321	1	0.04	$F0(x)$:=λx.pair(pair(ϵ, a), b).<br>$F1(x)$:=λx.ϵ.<br>$F2(x)$:=λx.pair(Fm0(F1(ϵ)), c).
$a^nb^nc^n$	abcd	4h	8	10 (6)	-68.9967	1	0.88	$F0(x)$:=λx.pair(append(pair(ϵ, a), x), b).<br>$F1(x)$:=λx.pair(if(flip(1/3), x, Fm2(x)), c).<br>$F2(x)$:=λx.F1(Fm0(x)).
$a^nb^nc^n$	abcd	4h	8	100 (10)	-233.581	1	0.88	$F0(x)$:=λx.pair(append(pair(ϵ, a), x), b).<br>$F1(x)$:=λx.pair(if(flip(3/8), x, F2(x)), c).<br>$F2(x)$:=λx.F1(F0(x)).
$a^nb^nc^n$	abcd	4h	8	1000 (19)	-1992.7	1	0.88	$F0(x)$:=λx.pair(append(pair(ϵ, a), x), b).<br>$F1(x)$:=λx.pair(if(flip(1/3), x, Fm2(x)), c).<br>$F2(x)$:=λx.F1(Fm0(x)).
$a^nb^nc^n$	abcd	4h	8	10000 (24)	-19242.3	1	1	$F0(x)$:=λx.append(pair(ϵ, a), insert(F1(ϵ), pair(ϵ, b))).<br>$F1(x)$:=λx.pair(if(flip(1/3), ϵ, Fm0(ϵ)), c).<br>$F2(x)$:=λx.Fm0(ϵ).
$a^nb^nc^n$	abcd	4h	8	100000 (28)	-193366	1	1	$F0(x)$:=λx.append(pair(ϵ, a), insert(F1(ϵ), pair(ϵ, b))).<br>$F1(x)$:=λx.pair(if(flip(1/3), ϵ, Fm0(ϵ)), c).<br>$F2(x)$:=λx.Fm0(ϵ).
$a^nb^nc^n$	abcd	4h	8	1 (1)	-37.5252	1	0.04	$F0(x)$:=λx.ϵ.<br>$F1(x)$:=λx.pair(pair(ϵ, a), b).<br>$F2(x)$:=λx.ϵ.<br>$F3(x)$:=λx.pair(Fm1(Fm2(F0(ϵ))), c).
$a^nb^nc^n$	abcd	4h	8	10 (6)	-80.3364	1	0.64	$F0(x)$:=λx.append(if(flip(1/3), x, F3(x)), F2(ϵ)).<br>$F1(x)$:=λx.pair(ϵ, a).<br>$F2(x)$:=λx.pair(ϵ, c).<br>$F3(x)$:=λx.Fm0(pair(append(Fm1(ϵ), x), b)).
$a^nb^nc^n$	abcd	4h	8	100 (10)	-244.92	1	0.84	$F0(x)$:=λx.append(if(flip(3/8), x, Fm3(x)), pair(ϵ, c)).<br>$F1(x)$:=λx.F2(ϵ).<br>$F2(x)$:=λx.pair(ϵ, a).<br>$F3(x)$:=λx.Fm0(pair(append(Fm1(ϵ), x), b)).
$a^nb^nc^n$	abcd	4h	8	1000 (19)	-2001.57	1	0.84	$F0(x)$:=λx.x.<br>$F1(x)$:=λx.x.<br>$F2(x)$:=λx.pair(if(flip(1/3), F1(x), F3(x)), c).<br>$F3(x)$:=λx.F0(Fm2(pair(append(pair(ϵ, a), x), b))).
$a^nb^nc^n$	abcd	4h	8	10000 (24)	-19252.6	1	1	$F0(x)$:=λx.x.<br>$F1(x)$:=λx.append(pair(ϵ, a), sample(if(flip(1/3), Fm2(x), Fm3(x)))).<br>$F2(x)$:=λx.F0(x).<br>$F3(x)$:=λx.pair(F1(pair(x, b)), c).
$a^nb^nc^n$	abcd	4h	8	100000 (28)	-193376	1	1	$F0(x)$:=λx.x.<br>$F1(x)$:=λx.append(pair(ϵ, a), sample(if(flip(1/3), Fm2(x), Fm3(x)))).<br>$F2(x)$:=λx.F0(x).<br>$F3(x)$:=λx.pair(F1(pair(x, b)), c).
$a^nb^nc^nd^n$	abcd	5d	8	1 (1)	-33.8107	0	0.04	$F0(x)$:=λx.append(x, append(x, if(flip(1/2), Fm0(sample(Σ)), ϵ))).
$a^nb^nc^nd^n$	abcd	5d	8	10 (6)	-62.7144	1	1	$F0(x)$:=λx.append(pair(ϵ, a), insert(pair(if(flip(1/4), ϵ, F0(ϵ)), d), pair(pair(ϵ, b), c))).
$a^nb^nc^nd^n$	abcd	5d	8	100 (11)	-227.763	1	1	$F0(x)$:=λx.insert(append(pair(ϵ, a), pair(if(flip(1/3), ϵ, Fm0(ϵ)), d)), pair(pair(ϵ, b), c)).
$a^nb^nc^nd^n$	abcd	5d	8	1000 (17)	-1999.69	1	1	$F0(x)$:=λx.insert(append(pair(ϵ, a), pair(if(flip(1/3), ϵ, Fm0(ϵ)), d)), pair(pair(ϵ, b), c)).
$a^nb^nc^nd^n$	abcd	5d	8	10000 (20)	-19286.8	1	1	$F0(x)$:=λx.insert(append(pair(ϵ, a), pair(if(flip(1/3), ϵ, Fm0(ϵ)), d)), pair(pair(ϵ, b), c)).
$a^nb^nc^nd^n$	abcd	5d	8	100000 (27)	-192851	1	1	$F0(x)$:=λx.insert(append(pair(ϵ, a), pair(if(flip(1/3), ϵ, Fm0(ϵ)), d)), pair(pair(ϵ, b), c)).
$a^nb^nc^nd^n$	abcd	5d	8	1 (1)	-40.4354	0	0.04	$F0(x)$:=λx.append(x, x).<br>$F1(x)$:=λx.append(F0(sample(Σ)), if(flip(1/2), Fm1(ϵ), ϵ)).
$a^nb^nc^nd^n$	abcd	5d	8	10 (6)	-72.4388	1	0.84	$F0(x)$:=λx.insert(pair(append(pair(ϵ, a), if(flip(1/2), ϵ, Fm0(ϵ))), d), pair(pair(ϵ, b), c)).<br>$F1(x)$:=λx.Fm0(ϵ).
$a^nb^nc^nd^n$	abcd	5d	8	100 (11)	-235.774	1	1	$F0(x)$:=λx.append(pair(ϵ, a), pair(x, d)).<br>$F1(x)$:=λx.F0(insert(if(flip(1/3), ϵ, F1(ϵ)), pair(pair(ϵ, b), c))).
$a^nb^nc^nd^n$	abcd	5d	8	1000 (17)	-2007.71	1	1	$F0(x)$:=λx.append(pair(ϵ, a), pair(x, d)).<br>$F1(x)$:=λx.F0(insert(if(flip(1/3), ϵ, F1(ϵ)), pair(pair(ϵ, b), c))).

$a^n b^n c^n d^n$	abcd	5d	8	10000 (20)	-19294.8	1	1	F0(x):=λx.append(pair(ε, a), pair(insert(x, pair(pair(ε, b), c)), d)).<br>F1(x):=λx.Fm0(if(flip(1/3), ε, F1(ε))).
$a^n b^n c^n d^n$	abcd	5d	8	100000 (27)	-192859	1	1	F0(x):=λx.append(pair(ε, a), pair(insert(x, pair(pair(ε, b), c)), d)).<br>F1(x):=λx.Fm0(if(flip(1/3), ε, F1(ε))).
$a^n b^n c^n d^n$	abcd	5d	8	1 (1)	-48.2764	0	0.04	F0(x):=λx.append(if(flip(1/2), ε, F2(ε)), append(x, x)).<br>F1(x):=λx.sample(Σ).<br>F2(x):=λx.F0(F1(ε)).
$a^n b^n c^n d^n$	abcd	5d	8	10 (6)	-78.7412	1	0.88	F0(x):=λx.insert(if(flip(3/8), ε, F2(ε)), F1(ε)).<br>F1(x):=λx.pair(pair(ε, b), c).<br>F2(x):=λx.append(pair(ε, a), pair(Fm0(ε), d)).
$a^n b^n c^n d^n$	abcd	5d	8	100 (11)	-242.922	1	0.88	F0(x):=λx.append(pair(F1(ε), a), insert(pair(if(flip(1/3), ε, Fm2(ε)), d), pair(pair(ε, b), c))).<br>F1(x):=λx.ε.<br>F2(x):=λx.Fm0(ε).
$a^n b^n c^n d^n$	abcd	5d	8	1000 (17)	-2014.85	1	0.88	F0(x):=λx.append(pair(F1(ε), a), insert(pair(if(flip(1/3), ε, Fm2(ε)), d), pair(pair(ε, b), c))).<br>F1(x):=λx.ε.<br>F2(x):=λx.Fm0(ε).
$a^n b^n c^n d^n$	abcd	5d	8	10000 (20)	-19301.9	1	0.88	F0(x):=λx.append(pair(F1(ε), a), insert(pair(if(flip(1/3), ε, Fm2(ε)), d), pair(pair(ε, b), c))).<br>F1(x):=λx.ε.<br>F2(x):=λx.Fm0(ε).
$a^n b^n c^n d^n$	abcd	5d	8	100000 (27)	-192867	1	1	F0(x):=λx.append(pair(x, a), insert(pair(if(flip(1/3), ε, Fm0(ε)), d), pair(pair(Fm1(ε), b), c))).<br>F1(x):=λx.ε.<br>F2(x):=λx.Fm0(ε).
$a^n b^n c^n d^n$	abcd	5d	8	1 (1)	-56.4572	0	0.04	F0(x):=λx.F1(ε).<br>F1(x):=λx.if(flip(1/2), F3(ε), ε).<br>F2(x):=λx.append(Fm0(ε), append(x, x)).<br>F3(x):=λx.F2(sample(Σ)).
$a^n b^n c^n d^n$	abcd	5d	8	10 (6)	-86.054	1	0.64	F0(x):=λx.Fm3(ε).<br>F1(x):=λx.ε.<br>F2(x):=λx.ε.<br>F3(x):=λx.append(pair(ε, a), insert(pair(if(flip(1/4), ε, Fm0(ε)), d), pair(pair(F2(Fm1(ε)), b), c))).
$a^n b^n c^n d^n$	abcd	5d	8	100 (11)	-251.103	1	0.84	F0(x):=λx.pair(Fm1(ε), d).<br>F1(x):=λx.Fm2(ε).<br>F2(x):=λx.append(pair(ε, a), insert(if(flip(1/3), ε, Fm0(ε)), pair(pair(ε, b), c))).<br>F3(x):=λx.F0(ε).
$a^n b^n c^n d^n$	abcd	5d	8	1000 (17)	-2023.03	1	0.84	F0(x):=λx.pair(Fm1(ε), d).<br>F1(x):=λx.Fm2(ε).<br>F2(x):=λx.append(pair(ε, a), insert(if(flip(1/3), ε, Fm0(ε)), pair(pair(ε, b), c))).<br>F3(x):=λx.F0(ε).
$a^n b^n c^n d^n$	abcd	5d	8	10000 (20)	-19310.8	1	0.88	F0(x):=λx.x.<br>F1(x):=λx.ε.<br>F2(x):=λx.Fm3(ε).<br>F3(x):=λx.append(pair(F1(ε), a), insert(pair(if(flip(1/3), F0(ε), F2(ε)), d), pair(pair(ε, b), c))).
$a^n b^n c^n d^n$	abcd	5d	8	100000 (27)	-192876	1	1	F0(x):=λx.ε.<br>F1(x):=λx.ε.<br>F2(x):=λx.ε.<br>F3(x):=λx.append(pair(x, a), insert(pair(if(flip(1/3), Fm2(Fm0(x)), F3(ε)), d), pair(pair(F1(ε), b), c))).
$a^n b^n c^n d^n e^n$	abcde	7d	8	1 (1)	-87.2916	0	0	F0(x):=λx.if(flip(1/2), if(empty(x), pair(F0(ε), d), x), append(pair(ε, a), pair(Fm0(pair(x, b)), c))).
$a^n b^n c^n d^n e^n$	abcde	7d	8	10 (4)	-136.934	0.12	0.4	F0(x):=λx.if(flip(11/24), if(not(empty(x)), x, pair(Fm0(ε), e)), append(pair(ε, a), pair(Fm0(append(pair(ε, b), pair(x, c))), d))).
$a^n b^n c^n d^n e^n$	abcde	7d	8	100 (11)	-597.112	0.10714	0.4	F0(x):=λx.if(flip(1/2), if(not(empty(x)), x, pair(Fm0(ε), e)), append(pair(ε, a), pair(Fm0(append(pair(ε, b), pair(x, c))), d))).
$a^n b^n c^n d^n e^n$	abcde	7d	8	1000 (15)	-5314.83	0.10714	0.4	F0(x):=λx.if(flip(1/2), if(not(empty(x)), x, pair(Fm0(ε), e)), append(pair(ε, a), pair(Fm0(append(pair(ε, b), pair(x, c))), d))).
$a^n b^n c^n d^n e^n$	abcde	7d	8	10000 (19)	-40857.6	0.14285	0.48	F0(x):=λx.if((append(ε, sample(ε))==x), pair(Fm0(append(ε, sample(if(flip(1/3), pair(tail(ε), b), tail(ε))))), e), append(pair(ε, a), pair(insert(if(not(flip(1/3)), F0(head(x)), head(sample(ε))), pair(sample(x), c)), d))).
$a^n b^n c^n d^n e^n$	abcde	7d	8	100000 (27)	-413371	0.14285	0.48	F0(x):=λx.if((append(ε, sample(ε))==x), pair(Fm0(append(ε, sample(if(flip(1/3), pair(tail(ε), b), tail(ε))))), e), append(pair(ε, a), pair(insert(if(not(flip(1/3)), F0(head(x)), head(sample(ε))), pair(sample(x), c)), d))).
$a^n b^n c^n d^n e^n$	abcde	7d	8	1 (1)	-70.0723	0	0.04	F0(x):=λx.append(sample(if(flip(1/2), ε, F0(sample((Σ\x))))), insert(x, append(append(x, x), append(x, x)))).<br>F1(x):=λx.Fm0(pair(ε, e)).
$a^n b^n c^n d^n e^n$	abcde	7d	8	10 (4)	-81.3187	1	0.84	F0(x):=λx.insert(pair(pair(ε, a), d), insert(x, pair(pair(ε, b), c))).<br>F1(x):=λx.pair(if(flip(1/2), Fm1(F0(x)), Fm0(x)), e).
$a^n b^n c^n d^n e^n$	abcde	7d	8	100 (11)	-250.496	1	1	F0(x):=λx.insert(pair(pair(ε, a), d), insert(x, pair(pair(ε, b), c))).<br>F1(x):=λx.pair(if(not(flip(3/8)), Fm1(F0(x)), Fm0(x)), e).
$a^n b^n c^n d^n e^n$	abcde	7d	8	1000 (15)	-1977.12	1	1	F0(x):=λx.insert(pair(pair(ε, a), d), insert(x, pair(pair(ε, b), c))).<br>F1(x):=λx.pair(if(not(flip(1/3)), Fm1(Fm0(x)), F0(x)), e).

$a^nb^nc^nd^ne^n$	abcde	7d	8	10000 (19)	-19329.9	1	1	$F0(x)$:=λx.insert(pair(pair(ε, a), d), insert(x, pair(pair(ε, b), c))).<br>$F1(x)$:=λx.pair(if(not(flip(1/3)), Fm1(Fm0(x)), F0(x)), e).
$a^nb^nc^nd^ne^n$	abcde	7d	8	100000 (27)	-193046	1	1	$F0(x)$:=λx.insert(pair(pair(ε, a), d), insert(x, pair(pair(ε, b), c))).<br>$F1(x)$:=λx.pair(if(not(flip(1/3)), Fm1(Fm0(x)), F0(x)), e).
$a^nb^nc^nd^ne^n$	abcde	7d	8	1 (1)	-72.2379	0	0.04	$F0(x)$:=λx.append(if(flip(1/2), F0(sample((Σ\x))), ε), append(x, append(append(x, append(x, x)), x))).<br>$F1(x)$:=λx.F0(pair(ε, e)).<br>$F2(x)$:=λx.Fm1(ε).
$a^nb^nc^nd^ne^n$	abcde	7d	8	10 (4)	-88.8154	0.95454	0.84	$F0(x)$:=λx.pair(pair(ε, b), c).<br>$F1(x)$:=λx.insert(append(pair(ε, a), pair(x, d)), Fm0(ε)).<br>$F2(x)$:=λx.if(not(flip(1/4)), pair(Fm2(F1(x)), e), x).
$a^nb^nc^nd^ne^n$	abcde	7d	8	100 (11)	-265.758	1	0.84	$F0(x)$:=λx.pair(pair(ε, b), c).<br>$F1(x)$:=λx.insert(append(pair(ε, a), pair(x, d)), F0(ε)).<br>$F2(x)$:=λx.if(or(empty(x), flip(1/2)), pair(Fm2(Fm1(x)), e), x).
$a^nb^nc^nd^ne^n$	abcde	7d	8	1000 (15)	-2138	1	0.84	$F0(x)$:=λx.pair(pair(ε, b), c).<br>$F1(x)$:=λx.insert(append(pair(ε, a), pair(x, d)), F0(ε)).<br>$F2(x)$:=λx.if(or(empty(x), flip(1/2)), pair(Fm2(Fm1(x)), e), x).
$a^nb^nc^nd^ne^n$	abcde	7d	8	10000 (19)	-21010.1	1	0.84	$F0(x)$:=λx.pair(pair(ε, b), c).<br>$F1(x)$:=λx.insert(append(pair(ε, a), pair(x, d)), F0(ε)).<br>$F2(x)$:=λx.if(or(empty(x), flip(1/2)), pair(Fm2(Fm1(x)), e), x).
$a^nb^nc^nd^ne^n$	abcde	7d	8	100000 (27)	-224681	1	0.84	$F0(x)$:=λx.pair(pair(x, b), c).<br>$F1(x)$:=λx.insert(append(pair(ε, a), pair(x, d)), F0(ε)).<br>$F2(x)$:=λx.if(or(empty(x), flip(1/2)), pair(Fm2(F1(x)), e), x).
$a^nb^nc^nd^ne^n$	abcde	7d	8	1 (1)	-79.644	0	0.04	$F0(x)$:=λx.append(if(flip(1/2), Fm0(sample((Σ\x))), ε), Fm1(append(x, x))).<br>$F1(x)$:=λx.append(append(x, head(x)), x).<br>$F2(x)$:=λx.ε.<br>$F3(x)$:=λx.F0(pair(Fm2(ε), e)).
$a^nb^nc^nd^ne^n$	abcde	7d	8	10 (4)	-131.815	0.10714	0.28	$F0(x)$:=λx.if(flip(1/2), pair(Fm0(F1(x)), d), if(empty(x), append(Fm3(ε), pair(ε, e)), x)).<br>$F1(x)$:=λx.append(pair(ε, a), Fm2(pair(x, c))).<br>$F2(x)$:=λx.insert(x, pair(ε, b)).<br>$F3(x)$:=λx.pair(Fm0(ε), e).
$a^nb^nc^nd^ne^n$	abcde	7d	8	100 (11)	-534.278	0.12	0.52	$F0(x)$:=λx.if(not(flip(if(empty(x), 1/2, 7/24))), pair(Fm0(F1(x)), d), if(empty(x), Fm3(ε), x)).<br>$F1(x)$:=λx.append(pair(ε, a), Fm2(x)).<br>$F2(x)$:=λx.insert(pair(x, c), pair(ε, b)).<br>$F3(x)$:=λx.pair(Fm0(ε), e).
$a^nb^nc^nd^ne^n$	abcde	7d	8	1000 (15)	-4576.22	0.14285	0.52	$F0(x)$:=λx.insert(F2(Fm1(ε)), pair(pair(ε, b), c)).<br>$F1(x)$:=λx.append(pair(Fm2(ε), a), pair(if(not(flip(1/3)), Fm0(ε), ε), d)).<br>$F2(x)$:=λx.x.<br>$F3(x)$:=λx.pair(if(not(flip(1/3)), F3(ε), F0(ε)), e).
$a^nb^nc^nd^ne^n$	abcde	7d	8	10000 (19)	-48215	0.14285	0.52	$F0(x)$:=λx.append(append(pair(x, d), sample(if(not(flip(1/3)), (Fm0(ε)\ε), insert(insert(tail(ε), if(empty(x), ε, if(empty(sample(if(flip(1/12), ε, Σ))), sample(if(empty(x), ε, ε)), if(flip(1/2), ε, ε)))), sample(ε))))), pair(sample(ε), e)).<br>$F1(x)$:=λx.pair(x, b).<br>$F2(x)$:=λx.if(flip(1/3), Fm1(x), pair(append(head(x), F2(pair(x, b))), c)).<br>$F3(x)$:=λx.F0(append(F2(pair(ε, a)), pair(ε, c))).
$a^nb^nc^nd^ne^n$	abcde	7d	8	100000 (27)	-436423	0.16	0.56	$F0(x)$:=λx.if((x==if(flip(1/8), x, append(x, Fm1(ε)))), x, pair(Fm2(x), c)).<br>$F1(x)$:=λx.if(not(flip(5/12)), append(pair(ε, d), pair(F1(ε), e)), ε).<br>$F2(x)$:=λx.append(pair(ε, a), F0(pair(x, b))).<br>$F3(x)$:=λx.pair(append(pair(pair(Fm2(tail(Fm2(ε))), c), d), Fm1(ε)), e).
a^{2^n}	abcd	7d	8	1 (1)	-6.6447	1	0.1	$F0(x)$:=λx.pair(ε, a).
a^{2^n}	abcd	7d	8	10 (2)	-18.8083	1	0.1	$F0(x)$:=λx.pair(ε, a).
a^{2^n}	abcd	7d	8	100 (3)	-79.1795	0.44444	0.4	$F0(x)$:=λx.pair(if(flip(1/6), Fm0(ε), ε), a).
a^{2^n}	abcd	7d	8	1000 (5)	-669.3	1	1	$F0(x)$:=λx.if(flip(5/24), F0(append(x, pair(x, a))), pair(x, a)).
a^{2^n}	abcd	7d	8	10000 (8)	-6513.75	1	1	$F0(x)$:=λx.if(flip(5/24), F0(append(x, pair(x, a))), pair(x, a)).
a^{2^n}	abcd	7d	8	100000 (8)	-64824.4	1	0.9	$F0(x)$:=λx.if(or(flip(1/6), flip(1/24)), Fm0(append(pair(x, a), x)), pair(x, a)).
a^{2^n}	abcd	7d	8	1 (1)	-13.2693	1	0.1	$F0(x)$:=λx.ε.<br>$F1(x)$:=λx.pair(Fm0(ε), a).
a^{2^n}	abcd	7d	8	10 (2)	-25.4329	1	0.1	$F0(x)$:=λx.ε.<br>$F1(x)$:=λx.pair(Fm0(ε), a).
a^{2^n}	abcd	7d	8	100 (3)	-86.4972	0.44444	0.4	$F0(x)$:=λx.ε.<br>$F1(x)$:=λx.pair(if(flip(1/6), Fm1(ε), Fm0(ε)), a).
a^{2^n}	abcd	7d	8	1000 (5)	-671.461	1	1	$F0(x)$:=λx.append(x, x).<br>$F1(x)$:=λx.if(flip(5/24), Fm0(Fm1(ε)), pair(ε, a)).

a^{2^n}	abcd	7d	8	10000 (8)	-6515.91	1	1	$F0(x)$:=λx.append(x, x).<br>$F1(x)$:=λx.if(flip(5/24), Fm0(Fm1(ϵ)), pair(ϵ, a)).
a^{2^n}	abcd	7d	8	100000 (8)	-64826.8	1	0.9	$F0(x)$:=λx.append(x, x).<br>$F1(x)$:=λx.if(or(flip(1/24), flip(1/6)), Fm0(F1(ϵ)), pair(ϵ, a)).
a^{2^n}	abcd	7d	8	1 (1)	-20.7049	1	0.1	$F0(x)$:=λx.pair(Fm1(ϵ), a).<br>$F1(x)$:=λx.ϵ.<br>$F2(x)$:=λx.F0(ϵ).
a^{2^n}	abcd	7d	8	10 (2)	-32.8685	1	0.1	$F0(x)$:=λx.pair(Fm1(ϵ), a).<br>$F1(x)$:=λx.ϵ.<br>$F2(x)$:=λx.F0(ϵ).
a^{2^n}	abcd	7d	8	100 (3)	-94.3383	0.44444	0.4	$F0(x)$:=λx.ϵ.<br>$F1(x)$:=λx.pair(if(flip(1/6), F1(F0(ϵ)), ϵ), a).<br>$F2(x)$:=λx.F1(ϵ).
a^{2^n}	abcd	7d	8	1000 (5)	-679.302	1	1	$F0(x)$:=λx.append(x, x).<br>$F1(x)$:=λx.ϵ.<br>$F2(x)$:=λx.if(flip(5/24), Fm0(F2(F1(ϵ))), pair(ϵ, a)).
a^{2^n}	abcd	7d	8	10000 (8)	-6523.75	1	1	$F0(x)$:=λx.append(x, x).<br>$F1(x)$:=λx.ϵ.<br>$F2(x)$:=λx.if(flip(5/24), Fm0(F2(F1(ϵ))), pair(ϵ, a)).
a^{2^n}	abcd	7d	8	100000 (8)	-64834.6	1	0.9	$F0(x)$:=λx.F2(ϵ).<br>$F1(x)$:=λx.append(x, x).<br>$F2(x)$:=λx.if(or(flip(1/24), flip(1/6)), Fm1(Fm0(ϵ)), pair(ϵ, a)).
a^{2^n}	abcd	7d	8	1 (1)	-28.598	1	0.1	$F0(x)$:=λx.ϵ.<br>$F1(x)$:=λx.ϵ.<br>$F2(x)$:=λx.pair(ϵ, a).<br>$F3(x)$:=λx.Fm2(Fm0(F1(ϵ))).
a^{2^n}	abcd	7d	8	10 (2)	-40.7616	1	0.1	$F0(x)$:=λx.ϵ.<br>$F1(x)$:=λx.ϵ.<br>$F2(x)$:=λx.pair(ϵ, a).<br>$F3(x)$:=λx.Fm2(Fm0(F1(ϵ))).
a^{2^n}	abcd	7d	8	100 (3)	-102.519	0.44444	0.4	$F0(x)$:=λx.if(flip(1/6), Fm3(ϵ), ϵ).<br>$F1(x)$:=λx.F2(ϵ).<br>$F2(x)$:=λx.pair(Fm0(ϵ), a).<br>$F3(x)$:=λx.F1(ϵ).
a^{2^n}	abcd	7d	8	1000 (5)	-687.482	1	1	$F0(x)$:=λx.append(x, x).<br>$F1(x)$:=λx.Fm3(ϵ).<br>$F2(x)$:=λx.if(flip(5/24), F0(F1(ϵ)), pair(ϵ, a)).<br>$F3(x)$:=λx.F2(ϵ).
a^{2^n}	abcd	7d	8	10000 (8)	-6531.93	1	1	$F0(x)$:=λx.append(x, x).<br>$F1(x)$:=λx.if(flip(5/24), Fm0(F1(ϵ)), pair(ϵ, a)).<br>$F2(x)$:=λx.Fm1(ϵ).<br>$F3(x)$:=λx.Fm2(ϵ).
a^{2^n}	abcd	7d	8	100000 (8)	-64842.5	1	0.9	$F0(x)$:=λx.Fm3(ϵ).<br>$F1(x)$:=λx.append(x, x).<br>$F2(x)$:=λx.if(or(flip(1/6), flip(1/24)), F1(Fm0(ϵ)), pair(ϵ, a)).<br>$F3(x)$:=λx.Fm2(ϵ).
$((ab)^{n^2}$	abcd	7d	8	1 (1)	-23.8257	0.19047	0.30769	$F0(x)$:=λx.pair(pair(if(flip(1/2), ϵ, Fm0(ϵ)), a), b).
$((ab)^{n^2}$	abcd	7d	8	10 (2)	-32.3237	0.19047	0.30769	$F0(x)$:=λx.pair(pair(if(flip(1/2), ϵ, Fm0(ϵ)), a), b).
$((ab)^{n^2}$	abcd	7d	8	100 (4)	-125.899	1	0.92307	$F0(x)$:=λx.pair(pair(append(append(x, x), if(flip(7/24), F0(pair(pair(x, a), b)), ϵ)), a), b).
$((ab)^{n^2}$	abcd	7d	8	1000 (8)	-1004.12	0.92857	1	$F0(x)$:=λx.pair(pair(append(append(x, x), if(flip(1/3), F0(pair(pair(x, a), b)), ϵ)), a), b).
$((ab)^{n^2}$	abcd	7d	8	10000 (8)	-9793.97	0.92857	1	$F0(x)$:=λx.if(or(empty(x), flip(1/3)), append(x, append(x, Fm0(pair(pair(x, a), b)))), x).
$((ab)^{n^2}$	abcd	7d	8	100000 (11)	-97599.4	0.92857	1	$F0(x)$:=λx.if(or(empty(x), flip(1/3)), append(x, append(x, Fm0(pair(pair(x, a), b)))), x).
$((ab)^{n^2}$	abcd	7d	8	1 (1)	-28.128	1	0.07692	$F0(x)$:=λx.append(x, x).<br>$F1(x)$:=λx.Fm0(Fm0(pair(pair(ϵ, a), b))).
$((ab)^{n^2}$	abcd	7d	8	10 (2)	-39.6415	0.19047	0.30769	$F0(x)$:=λx.ϵ.<br>$F1(x)$:=λx.pair(pair(if(flip(1/2), F1(ϵ), Fm0(ϵ)), a), b).
$((ab)^{n^2}$	abcd	7d	8	100 (4)	-124.982	1	0.92307	$F0(x)$:=λx.append(if(flip(7/24), append(Fm1(x), x), ϵ), x).<br>$F1(x)$:=λx.Fm0(pair(pair(x, a), b)).

$((ab)^{n^2}$	abcd	7d	8	1000 (8)	-1003.2	0.92857	1	$F0(x)$:=λx.append(if(flip(1/3), append(x, F1(x)), ϵ), x).<br>$F1(x)$:=λx.Fm0(pair(pair(x, a), b)).
$((ab)^{n^2}$	abcd	7d	8	10000 (8)	-9794.4	0.92857	1	$F0(x)$:=λx.append(if(flip(1/3), append(x, F1(x)), ϵ), x).<br>$F1(x)$:=λx.Fm0(pair(pair(x, a), b)).
$((ab)^{n^2}$	abcd	7d	8	100000 (11)	-97599.8	0.92857	1	$F0(x)$:=λx.append(if(flip(1/3), append(x, F1(x)), ϵ), x).<br>$F1(x)$:=λx.Fm0(pair(pair(x, a), b)).
$((ab)^{n^2}$	abcd	7d	8	1 (1)	-35.6452	1	0.07692	$F0(x)$:=λx.append(x, x).<br>$F1(x)$:=λx.append(x, x).<br>$F2(x)$:=λx.Fm0(F1(pair(pair(ϵ, a), b))).
$((ab)^{n^2}$	abcd	7d	8	10 (2)	-47.4825	0.19047	0.30769	$F0(x)$:=λx.F2(ϵ).<br>$F1(x)$:=λx.if(flip(1/2), F0(ϵ), ϵ).<br>$F2(x)$:=λx.pair(pair(F1(ϵ), a), b).
$((ab)^{n^2}$	abcd	7d	8	100 (4)	-132.823	1	0.92307	$F0(x)$:=λx.append(if(flip(7/24), append(x, F2(x)), F1(ϵ)), x).<br>$F1(x)$:=λx.ϵ.<br>$F2(x)$:=λx.Fm0(pair(pair(x, a), b)).
$((ab)^{n^2}$	abcd	7d	8	1000 (8)	-1011.04	0.92857	1	$F0(x)$:=λx.ϵ.<br>$F1(x)$:=λx.append(if(flip(1/3), append(F2(x), x), F0(ϵ)), x).<br>$F2(x)$:=λx.F1(pair(pair(x, a), b)).
$((ab)^{n^2}$	abcd	7d	8	10000 (8)	-9802.24	0.92857	1	$F0(x)$:=λx.ϵ.<br>$F1(x)$:=λx.append(if(flip(1/3), append(F2(x), x), F0(ϵ)), x).<br>$F2(x)$:=λx.F1(pair(pair(x, a), b)).
$((ab)^{n^2}$	abcd	7d	8	100000 (11)	-97607.7	0.92857	1	$F0(x)$:=λx.ϵ.<br>$F1(x)$:=λx.append(if(flip(1/3), append(F2(x), x), F0(ϵ)), x).<br>$F2(x)$:=λx.F1(pair(pair(x, a), b)).
$((ab)^{n^2}$	abcd	7d	8	1 (1)	-43.5383	1	0.07692	$F0(x)$:=λx.append(x, x).<br>$F1(x)$:=λx.Fm0(Fm2(pair(pair(ϵ, a), b))).<br>$F2(x)$:=λx.append(x, x).<br>$F3(x)$:=λx.Fm1(ϵ).
$((ab)^{n^2}$	abcd	7d	8	10 (2)	-55.6633	0.25	0.30769	$F0(x)$:=λx.pair(pair(if(flip(1/2), ϵ, Fm3(F2(ϵ))), a), b).<br>$F1(x)$:=λx.ϵ.<br>$F2(x)$:=λx.ϵ.<br>$F3(x)$:=λx.Fm0(F1(ϵ)).
$((ab)^{n^2}$	abcd	7d	8	100 (4)	-141.004	1	0.92307	$F0(x)$:=λx.ϵ.<br>$F1(x)$:=λx.append(if(flip(7/24), append(x, Fm2(x)), Fm0(ϵ)), x).<br>$F2(x)$:=λx.Fm1(pair(pair(x, a), b)).<br>$F3(x)$:=λx.Fm2(ϵ).
$((ab)^{n^2}$	abcd	7d	8	1000 (8)	-1019.22	0.92857	1	$F0(x)$:=λx.append(if(flip(1/3), append(Fm3(x), x), Fm1(ϵ)), x).<br>$F1(x)$:=λx.F2(ϵ).<br>$F2(x)$:=λx.ϵ.<br>$F3(x)$:=λx.F0(pair(pair(x, a), b)).
$((ab)^{n^2}$	abcd	7d	8	10000 (8)	-9810.42	0.92857	1	$F0(x)$:=λx.append(if(flip(1/3), append(x, F1(x)), ϵ), x).<br>$F1(x)$:=λx.F0(pair(pair(x, a), b)).<br>$F2(x)$:=λx.ϵ.<br>$F3(x)$:=λx.Fm1(Fm2(ϵ)).
$((ab)^{n^2}$	abcd	7d	8	100000 (11)	-97615.9	0.92857	1	$F0(x)$:=λx.append(if(flip(1/3), append(x, F1(x)), ϵ), x).<br>$F1(x)$:=λx.F0(pair(pair(x, a), b)).<br>$F2(x)$:=λx.ϵ.<br>$F3(x)$:=λx.Fm1(Fm2(ϵ)).
Count	abcd	2d	8	1 (1)	-29.7573	0.95238	0.8	$F0(x)$:=λx.if(flip(1/2), append(pair(x, a), Fm0(pair(x, b))), x).
Count	abcd	2d	8	10 (5)	-48.9576	1	0.84	$F0(x)$:=λx.append(pair(x, a), pair(if(flip(1/2), F0(pair(x, b)), ϵ), b)).
Count	abcd	2d	8	100 (7)	-193.585	1	1	$F0(x)$:=λx.append(pair(x, a), pair(if(flip(5/12), ϵ, Fm0(pair(x, b))), b)).
Count	abcd	2d	8	1000 (13)	-1773.04	1	1	$F0(x)$:=λx.append(pair(x, a), pair(if(flip(3/8), ϵ, Fm0(pair(x, b))), b)).
Count	abcd	2d	8	10000 (18)	-17022.4	1	1	$F0(x)$:=λx.append(pair(x, a), pair(if(or(flip(1/24), flip(3/8)), ϵ, Fm0(pair(x, b))), b)).
Count	abcd	2d	8	100000 (24)	-170440	1	1	$F0(x)$:=λx.append(pair(x, a), pair(if(or(flip(1/24), flip(3/8)), ϵ, Fm0(pair(x, b))), b)).
Count	abcd	2d	8	1 (1)	-34.2011	1	0.04	$F0(x)$:=λx.pair(insert(pair(x, b), pair(x, a)), b).<br>$F1(x)$:=λx.F0(Fm0(ϵ)).
Count	abcd	2d	8	10 (5)	-53.1981	1	0.84	$F0(x)$:=λx.if(flip(1/2), x, F1(x)).<br>$F1(x)$:=λx.append(pair(x, a), Fm0(pair(x, b))).
Count	abcd	2d	8	100 (7)	-197.826	1	1	$F0(x)$:=λx.if(flip(5/12), x, Fm1(x)).<br>$F1(x)$:=λx.append(pair(x, a), Fm0(pair(x, b))).

Count	abcd	2d	8	1000 (13)	-1777.28	1	1	F0(x):=λx.if(flip(3/8), x, F1(x)).<br>F1(x):=λx.append(pair(x, a), Fm0(pair(x, b))).
Count	abcd	2d	8	10000 (18)	-17026.6	1	1	F0(x):=λx.if(or(flip(3/8), flip(1/24)), x, Fm1(x)).<br>F1(x):=λx.append(pair(x, a), F0(pair(x, b))).
Count	abcd	2d	8	100000 (24)	-170444	1	1	F0(x):=λx.if(or(flip(3/8), flip(1/24)), x, Fm1(x)).<br>F1(x):=λx.append(pair(x, a), F0(pair(x, b))).
Count	abcd	2d	8	1 (1)	-42.0421	1	0.04	F0(x):=λx.insert(pair(x, b), pair(pair(x, a), b)).<br>F1(x):=λx.ϵ.<br>F2(x):=λx.Fm0(F0(F1(ϵ))).
Count	abcd	2d	8	10 (5)	-60.346	1	0.84	F0(x):=λx.Fm1(pair(x, b)).<br>F1(x):=λx.append(x, if(flip(1/2), F0(x), ϵ)).<br>F2(x):=λx.Fm0(pair(ϵ, a)).
Count	abcd	2d	8	100 (7)	-205.634	1	0.84	F0(x):=λx.Fm1(pair(x, b)).<br>F1(x):=λx.append(x, if(flip(1/2), F0(x), ϵ)).<br>F2(x):=λx.Fm0(pair(ϵ, a)).
Count	abcd	2d	8	1000 (13)	-1784.42	1	1	F0(x):=λx.Fm1(pair(x, b)).<br>F1(x):=λx.append(x, if(flip(3/8), ϵ, Fm0(x))).<br>F2(x):=λx.Fm0(pair(ϵ, a)).
Count	abcd	2d	8	10000 (18)	-17033.8	1	1	F0(x):=λx.append(x, if(or(flip(3/8), flip(1/24)), ϵ, F1(x))).<br>F1(x):=λx.Fm0(pair(x, b)).<br>F2(x):=λx.Fm1(pair(ϵ, a)).
Count	abcd	2d	8	100000 (24)	-170451	1	1	F0(x):=λx.append(x, if(or(flip(3/8), flip(1/24)), ϵ, F1(x))).<br>F1(x):=λx.Fm0(pair(x, b)).<br>F2(x):=λx.Fm1(pair(ϵ, a)).
Count	abcd	2d	8	1 (1)	-52.4655	1	0.04	F0(x):=λx.ϵ.<br>F1(x):=λx.Fm2(F2(ϵ)).<br>F2(x):=λx.append(pair(pair(ϵ, a), b), append(x, pair(x, b))).<br>F3(x):=λx.F1(F0(ϵ)).
Count	abcd	2d	8	10 (5)	-69.22	1	0.84	F0(x):=λx.if(flip(1/2), append(x, F2(x)), x).<br>F1(x):=λx.F2(pair(ϵ, a)).<br>F2(x):=λx.F0(pair(x, b)).<br>F3(x):=λx.F1(ϵ).
Count	abcd	2d	8	100 (7)	-213.155	1	1	F0(x):=λx.pair(ϵ, a).<br>F1(x):=λx.Fm2(pair(x, b)).<br>F2(x):=λx.append(x, if(flip(5/12), ϵ, Fm1(x))).<br>F3(x):=λx.Fm1(Fm0(ϵ)).
Count	abcd	2d	8	1000 (13)	-1792.6	1	1	F0(x):=λx.Fm2(pair(x, b)).<br>F1(x):=λx.Fm0(pair(ϵ, a)).<br>F2(x):=λx.append(x, if(flip(3/8), ϵ, F0(x))).<br>F3(x):=λx.F1(ϵ).
Count	abcd	2d	8	10000 (18)	-17042.6	1	1	F0(x):=λx.append(pair(x, a), F2(pair(x, b))).<br>F1(x):=λx.ϵ.<br>F2(x):=λx.if(or(flip(3/8), flip(1/24)), x, F0(x)).<br>F3(x):=λx.F0(Fm1(ϵ)).
Count	abcd	2d	8	100000 (24)	-170459	1	1	F0(x):=λx.append(x, if(or(flip(1/24), flip(3/8)), ϵ, Fm1(x))).<br>F1(x):=λx.Fm0(pair(x, b)).<br>F2(x):=λx.pair(ϵ, a).<br>F3(x):=λx.F1(Fm2(ϵ)).
ChineseNumeral	ab	7d	8	1 (1)	-28.5743	1	0.04	F0(x):=λx.pair(pair(pair(pair(pair(pair(pair(ϵ, a), b), b), b), a), b), b).
ChineseNumeral	ab	7d	8	10 (10)	-201.074	1	0.76	F0(x):=λx.pair(if(not(flip(1/2)), F0(pair(pair(x, b), b)), pair(if(flip(1/2), ϵ, pair(append(Fm0(pair(x, b)), x), b)), a)), b).
ChineseNumeral	ab	7d	8	100 (73)	-10723.7	0.89285	0.52	F0(x):=λx.pair(if(flip(1/8), pair(if(not(flip(1/3)), pair(append(pair(ϵ, a), pair(append(if(not(and((x==head(x)), flip(1/2))), append(x, append(pair(pair(if(flip(1/2), head(x), ϵ), b), b), head(x))), x), if(flip(1/2), ϵ, append(pair(ϵ, a), x))), b)), b), ϵ), a), F0(pair(x, b))), b).
ChineseNumeral	ab	7d	8	1000 (473)	-72821.5	0.88	0.6	F0(x):=λx.pair(if(flip(1/8), pair(if(or(flip(1/3), flip(1/2)), pair(append(pair(ϵ, a), pair(append(if(not(and((x==head(x)), flip(3/8))), append(x, append(pair(pair(if(flip(1/2), pair(ϵ, b), ϵ), b), b), head(x))), x), if(flip(1/2), ϵ, append(pair(ϵ, a), x))), b)), b), ϵ), a), F0(pair(x, b))), b).
ChineseNumeral	ab	7d	8	10000 (3185)	-672035	0.88461	0.6	F0(x):=λx.pair(if(flip(1/8), pair(if(not(flip(7/24)), pair(append(pair(ϵ, a), pair(append(if(not(and((head(x)==x), flip(1/3))), append(x, append(pair(pair(if(flip(1/3), pair(ϵ, b), ϵ), b), b), head(x))), x), if(flip(1/2), ϵ, append(pair(ϵ, a), x))), b)), b), ϵ), a), F0(pair(x, b))), b).
ChineseNumeral	ab	7d	8	100000 (21365)	-6.75518e+06	0.92307	0.6	F0(x):=λx.pair(if(flip(1/8), pair(if(not(flip(1/3)), pair(append(pair(ϵ, a), pair(append(if(not(and((x==head(x)), flip(1/3))), append(x, append(pair(pair(if(flip(1/3), pair(ϵ, b), ϵ), b), b), head(x))), x), if(flip(1/2), ϵ, append(pair(ϵ, a), x))), b)), b), ϵ), a), F0(pair(x, b))), b).
ChineseNumeral	ab	7d	8	1 (1)	-29.1259	1	0.04	F0(x):=λx.append(pair(x, b), x).<br>F1(x):=λx.F0(pair(pair(pair(ϵ, a), b), b)).
ChineseNumeral	ab	7d	8	10 (10)	-202.154	0.81481	0.64	F0(x):=λx.pair(if(flip(11/24), Fm0(pair(pair(x, b), b)), pair(if(flip(11/24), append(Fm1(pair(x, b)), x), ϵ), a)), b).<br>F1(x):=λx.pair(F0(x), b).

ChineseNumeral	ab	7d	8	100 (73)	-10088.2	1	0.48	F0(x):=λx.pair(if(flip(1/12), pair(ε, a), F0(ε)), b).<br>F1(x):=λx.append(if(flip(1/2), pair(append(pair(F1(pair(append(x, x), b)), b), append(x, pair(if(empty(x), ε, if(flip(1/2), ε, x)), b))), b), ε), Fm0(ε)).
ChineseNumeral	ab	7d	8	1000 (473)	-68691.8	1	0.56	F0(x):=λx.pair(if(flip(1/8), pair(if(flip(11/24), append(pair(x, b), if(not(flip(7/24)), if(flip(11/24), append(pair(if(flip(5/12), pair(ε, b), if(flip(1/2), ε, ε)), b), x), pair(pair(ε, b), b)), x)), if(flip(1/4), x, ε)), a), Fm0(pair(x, b))), b).<br>F1(x):=λx.F0(pair(append(pair(pair(ε, a), b), pair(ε, b)), b)).
ChineseNumeral	ab	7d	8	10000 (3185)	-634018	1	0.56	F0(x):=λx.pair(if(flip(1/8), pair(if(not(flip(1/3)), append(pair(x, b), if(not(flip(7/24)), if(flip(5/12), append(pair(if(flip(1/2), pair(if(flip(11/24), ε, ε), b), if(flip(5/12), pair(pair(ε, b), b), ε)), b), x), pair(pair(ε, b), b)), x)), if(and(flip(1/4), flip(1/12)), x, ε)), a), Fm0(pair(x, b))), b).<br>F1(x):=λx.F0(pair(append(pair(pair(ε, a), b), pair(ε, b)), b)).
ChineseNumeral	ab	7d	8	100000 (21365)	-6.35349e+06	1	0.56	F0(x):=λx.pair(if(flip(1/8), pair(if(not(flip(1/3)), append(pair(x, b), if(not(flip(7/24)), if(flip(5/12), append(pair(if(flip(1/2), pair(if(flip(11/24), ε, ε), b), if(flip(5/12), pair(pair(ε, b), b), ε)), b), x), pair(pair(ε, b), b)), x)), if(and(flip(1/4), flip(1/12)), x, ε)), a), Fm0(pair(x, b))), b).<br>F1(x):=λx.F0(pair(append(pair(pair(ε, a), b), pair(ε, b)), b)).
ChineseNumeral	ab	7d	8	1 (1)	-36.5614	1	0.04	F0(x):=λx.append(pair(x, b), x).<br>F1(x):=λx.Fm0(pair(pair(pair(ε, a), b), b)).<br>F2(x):=λx.Fm1(ε).
ChineseNumeral	ab	7d	8	10 (10)	-215.316	1	0.76	F0(x):=λx.ε.<br>F1(x):=λx.pair(pair(x, b), b).<br>F2(x):=λx.pair(if(flip(1/2), pair(if(flip(1/2), Fm0(ε), pair(append(Fm2(pair(x, b)), x), b)), a), Fm2(Fm1(x))), b).
ChineseNumeral	ab	7d	8	100 (73)	-9922.79	1	0.52	F0(x):=λx.pair(if(flip(if(empty(sample(sample(ε))), if(empty(ε), 1/24, 1/2), 1/2)), pair(ε, a), F0(ε)), b).<br>F1(x):=λx.if(flip(5/12), append(pair(F2(insert(x, x)), b), if(flip(1/12), Fm0(ε), x)), ε).<br>F2(x):=λx.append(F1(pair(x, b)), Fm0(ε)).
ChineseNumeral	ab	7d	8	1000 (473)	-70663.4	0.96	0.32	F0(x):=λx.pair(if(flip(1/8), x, F0(pair(x, b))), b).<br>F1(x):=λx.append(if(flip(1/2), pair(pair(Fm1(append(x, append(if(flip(11/24), pair(ε, b), ε), pair(ε, b)))), b), b), ε), x).<br>F2(x):=λx.Fm1(Fm0(pair(ε, a))).
ChineseNumeral	ab	7d	8	10000 (3185)	-667189	0.92	0.56	F0(x):=λx.append(append(pair(append(pair(ε, a), if((pair(ε, b)==x), ε, x)), b), sample((ε ∪ pair(pair(if(flip(1/2), ε, pair(if(or(flip(3/8), empty(x)), pair(ε, b), ε), b))), b), a)))), if(not(or(flip(1/6), (x==head(x)))), append(pair(ε, b), append(pair(x, a), x)), x)).<br>F1(x):=λx.if(flip(1/12), pair(x, b), pair(Fm1(if(empty(if((append(ε, x)==x), ε, x)), ε, ε)), b)).<br>F2(x):=λx.F0(if(flip(1/3), if(not(flip(5/12)), pair(ε, b), x), F1(pair(x, b)))).
ChineseNumeral	ab	7d	8	100000 (21365)	-6.69625e+06	0.92	0.56	F0(x):=λx.append(append(pair(append(pair(ε, a), if((pair(ε, b)==x), ε, x)), b), sample((ε ∪ pair(pair(if(flip(1/2), ε, pair(if(or(flip(3/8), (x==ε)), pair(ε, b), ε), b))), b), a)))), if(not(or(flip(1/6), (x==head(x)))), append(head(x), append(pair(x, a), x)), x)).<br>F1(x):=λx.if(flip(1/12), pair(x, b), pair(Fm1(append(ε, append(tail(append(pair(ε, b), pair(ε, b))), ε))), b)).<br>F2(x):=λx.F0(if(flip(1/3), if(or(flip(3/8), flip(3/8)), pair(ε, b), ε), F1(pair(ε, b)))).
ChineseNumeral	ab	7d	8	1 (1)	-44.4546	1	0.04	F0(x):=λx.F2(ε).<br>F1(x):=λx.append(pair(x, b), x).<br>F2(x):=λx.F1(pair(pair(pair(ε, a), b), b)).<br>F3(x):=λx.F0(ε).
ChineseNumeral	ab	7d	8	10 (10)	-219.125	0.84848	0.64	F0(x):=λx.if(flip(1/2), Fm2(pair(x, b)), F3(Fm1(x))).<br>F1(x):=λx.pair(pair(x, b), b).<br>F2(x):=λx.pair(pair(if(not(flip(1/2)), append(F0(x), x), ε), a), b).<br>F3(x):=λx.pair(F0(x), b).
ChineseNumeral	ab	7d	8	100 (73)	-9748.44	0.92	0.36	F0(x):=λx.pair(pair(Fm1(ε), b), b).<br>F1(x):=λx.if(flip(1/24), pair(append(ε, if(empty(sample(ε)), ε, ε)), a), append(append(ε, ε), pair(F1(head(head(pair(append(head(x), ε), b)))), b))).<br>F2(x):=λx.pair(if(and(empty(ε), not(flip(11/24))), F2(ε), Fm0(ε)), b).<br>F3(x):=λx.if(flip(1/4), if(or(flip(if(flip(1/2), 11/24, 11/24)), or(flip(5/12), flip(5/12))), pair(pair(ε, a), b), Fm0(sample(Σ))), append(Fm2(ε), append(F0(ε), Fm1(x)))).
ChineseNumeral	ab	7d	8	1000 (473)	-66711.9	0.84	0.4	F0(x):=λx.if(flip(3/8), pair(Fm0(ε), b), if(flip(3/8), pair(pair(ε, b), b), ε)).<br>F1(x):=λx.if(not(flip(1/6)), pair(if(flip(1/24), pair(x, a), pair(F1(pair(pair(x, b), b)), b)), b), if(flip(5/12), pair(pair(ε, a), b), append(x, pair(append(pair(Fm0(ε), b), append(ε, x)), a)))).<br>F2(x):=λx.Fm1(pair(sample((pair(x, b) ∪ x)), b)).<br>F3(x):=λx.F2(pair(pair(ε, a), b)).
ChineseNumeral	ab	7d	8	10000 (3185)	-633325	0.84	0.4	F0(x):=λx.if(flip(3/8), pair(Fm0(ε), b), if(flip(3/8), pair(pair(ε, b), b), ε)).<br>F1(x):=λx.if(not(flip(1/6)), pair(if(flip(1/24), pair(x, a), pair(F1(pair(pair(x, b), b)), b)), b), if(flip(5/12), pair(pair(ε, a), b), append(x, pair(append(pair(Fm0(ε), b), append(ε, x)), a)))).<br>F2(x):=λx.Fm1(pair(sample((pair(x, b) ∪ x)), b)).<br>F3(x):=λx.F2(pair(pair(ε, a), b)).
ChineseNumeral	ab	7d	8	100000 (21365)	-6.35429e+06	0.84	0.4	F0(x):=λx.if(flip(3/8), pair(Fm0(append(ε, ε)), b), if(flip(3/8), pair(pair(ε, b), b), ε)).<br>F1(x):=λx.if(not(flip(1/6)), pair(if(flip(1/24), pair(x, a), pair(F1(pair(pair(x, b), b)), b)), b), if(flip(11/24), pair(pair(head(ε), a), b), append(x, pair(append(pair(Fm0(ε), b), append(ε, x)), a)))).<br>F2(x):=λx.Fm1(pair(sample((pair(x, b) ∪ x)), b)).<br>F3(x):=λx.F2(pair(pair(ε, a), b)).
Newport & Aslin (2004)	btgdprkuli1	7d	8	1 (1)	-19.3302	1	0.05	F0(x):=λx.pair(pair(pair(ε, k), 4), u).

Newport & Aslin (2004)	btgdprkuli1	7d	8	10 (9)	-115.949	0.28571	0.4	F0(x):=λx.insert(if(flip(1/2), pair(pair(ε, k), u), if(flip(1/2), pair(pair(ε, p), r), sample(Σ))), sample(Σ)).
Newport & Aslin (2004)	btgdprkuli1	7d	8	100 (20)	-513.752	0.29824	1	F0(x):=λx.insert(if(flip(1/6), pair(pair(ε, p), r), sample(if(flip(1/2), if(flip(1/2), pair(pair(ε, k), u), pair(pair(ε, b), t)), if(flip(1/2), pair(pair(ε, g), d), pair(pair(ε, l), i))))), if(not(flip(7/24)), sample(Σ), pair(x, 3))).
Newport & Aslin (2004)	btgdprkuli1	7d	8	1000 (20)	-4211.9	0.29824	1	F0(x):=λx.insert(if(flip(1/6), pair(pair(x, p), r), sample(if(flip(1/2), if(flip(1/2), pair(pair(ε, k), u), pair(pair(ε, b), t)), if(flip(1/2), pair(pair(x, g), d), pair(pair(ε, l), i))))), if(not(flip(1/4)), sample(Σ), pair(ε, 3))).
Newport & Aslin (2004)	btgdprkuli1	7d	8	10000 (20)	-41347.2	0.29824	1	F0(x):=λx.insert(if(flip(1/6), pair(pair(x, p), r), sample(if(flip(1/2), if(flip(1/2), pair(pair(ε, k), u), pair(pair(ε, b), t)), if(flip(1/2), pair(pair(x, g), d), pair(pair(ε, l), i))))), if(not(flip(1/4)), sample(Σ), pair(ε, 3))).
Newport & Aslin (2004)	btgdprkuli1	7d	8	100000 (20)	-412928	0.29824	1	F0(x):=λx.insert(if(flip(1/6), pair(pair(x, p), r), sample(if(flip(1/2), if(flip(1/2), pair(pair(ε, k), u), pair(pair(ε, b), t)), if(flip(1/2), pair(pair(x, g), d), pair(pair(ε, l), i))))), if(not(flip(1/4)), sample(Σ), pair(ε, 3))).
Newport & Aslin (2004)	btgdprkuli1	7d	8	1 (1)	-25.9549	1	0.05	F0(x):=λx.ε.<br>F1(x):=λx.pair(pair(pair(F0(ε), k), 4), u).
Newport & Aslin (2004)	btgdprkuli1	7d	8	10 (9)	-122.27	0.28571	0.4	F0(x):=λx.pair(ε, p).<br>F1(x):=λx.insert(sample((pair(F0(ε), r) ∪ if(flip(1/2), Σ, pair(pair(ε, k), u)))), sample(Σ)).
Newport & Aslin (2004)	btgdprkuli1	7d	8	100 (20)	-503.499	0.34782	1	F0(x):=λx.if(flip(1/3), if(flip(1/2), pair(pair(ε, p), r), insert(pair(ε, t), pair(ε, b))), sample(((pair(pair(ε, k), u) ∪ x) ∪ pair(pair(ε, l), i)))).<br>F1(x):=λx.insert(Fm0(pair(pair(ε, g), d)), sample(if(flip(1/2), if(flip(1/2), pair(ε, 3), pair(ε, 2)), Σ))).
Newport & Aslin (2004)	btgdprkuli1	7d	8	1000 (20)	-3258.62	1	1	F0(x):=λx.sample(if(not(flip(5/24)), (pair(pair(ε, g), d) ∪ if(flip(3/8), pair(pair(ε, p), r), (pair(pair(ε, b), t) ∪ pair(pair(ε, l), i)))), pair(pair(ε, k), u))).<br>F1(x):=λx.insert(Fm0(ε), sample((((((Σ\pair(ε, u))\pair(ε, g))\(pair(x, d) ∪ pair(ε, i)))\(((pair(x, p) ∪ pair(x, t)) ∪ pair(ε, l)) ∪ pair(ε, r)))\(pair(ε, b) ∪ pair(ε, k))))).
Newport & Aslin (2004)	btgdprkuli1	7d	8	10000 (20)	-30743.1	1	1	F0(x):=λx.sample(if(not(flip(5/24)), (pair(pair(ε, g), d) ∪ if(flip(3/8), pair(pair(ε, p), r), (pair(pair(ε, b), t) ∪ pair(pair(ε, l), i)))), pair(pair(ε, k), u))).<br>F1(x):=λx.insert(Fm0(ε), sample((((((Σ\pair(ε, u))\pair(ε, g))\(pair(x, d) ∪ pair(ε, i)))\(((pair(x, p) ∪ pair(x, t)) ∪ pair(ε, l)) ∪ pair(ε, r)))\(pair(ε, b) ∪ pair(ε, k))))).
Newport & Aslin (2004)	btgdprkuli1	7d	8	100000 (20)	-305811	1	1	F0(x):=λx.sample(if(not(flip(5/24)), (pair(pair(ε, g), d) ∪ if(flip(3/8), pair(pair(ε, p), r), (pair(pair(ε, b), t) ∪ pair(pair(ε, l), i)))), pair(pair(ε, k), u))).<br>F1(x):=λx.insert(Fm0(ε), sample((((((Σ\pair(ε, u))\pair(ε, g))\(pair(x, d) ∪ pair(ε, i)))\(((pair(x, p) ∪ pair(x, t)) ∪ pair(ε, l)) ∪ pair(ε, r)))\(pair(ε, b) ∪ pair(ε, k))))).
Newport & Aslin (2004)	btgdprkuli1	7d	8	1 (1)	-33.3904	1	0.05	F0(x):=λx.pair(pair(pair(ε, k), 4), u).<br>F1(x):=λx.F0(ε).<br>F2(x):=λx.F1(ε).
Newport & Aslin (2004)	btgdprkuli1	7d	8	10 (9)	-130.702	0.28571	0.4	F0(x):=λx.if(flip(1/2), if(flip(1/2), Fm1(ε), sample(Σ)), pair(pair(x, k), u)).<br>F1(x):=λx.pair(pair(ε, p), r).<br>F2(x):=λx.insert(Fm0(ε), sample(Σ)).
Newport & Aslin (2004)	btgdprkuli1	7d	8	100 (20)	-455.18	1	1	F0(x):=λx.pair(pair(ε, p), r).<br>F1(x):=λx.insert(if(flip(7/24), pair(pair(ε, g), d), if(flip(1/2), sample(if(flip(1/2), Fm0(ε), pair(pair(ε, b), t))), if(flip(1/2), pair(pair(ε, l), i), pair(pair(ε, k), u)))), if(flip(1/3), pair(ε, 3), x)).<br>F2(x):=λx.F1(sample(if(flip(1/2), (pair(ε, 2) ∪ pair(x, 1)), pair(ε, 4)))).
Newport & Aslin (2004)	btgdprkuli1	7d	8	1000 (20)	-3180.76	1	1	F0(x):=λx.pair(pair(ε, p), r).<br>F1(x):=λx.insert(if(flip(1/4), pair(pair(ε, g), d), if(flip(1/2), sample(if(flip(1/2), Fm0(ε), pair(pair(ε, b), t))), if(flip(1/2), pair(pair(ε, l), i), pair(pair(ε, k), u)))), if(flip(7/24), pair(ε, 3), x)).<br>F2(x):=λx.F1(sample(if(not(flip(3/8)), (pair(x, 2) ∪ pair(ε, 1)), append(ε, pair(ε, 4))))).
Newport & Aslin (2004)	btgdprkuli1	7d	8	10000 (20)	-30370.7	1	1	F0(x):=λx.pair(append(x, pair(x, p)), r).<br>F1(x):=λx.insert(if(flip(5/24), pair(insert(pair(ε, g), ε), d), if(flip(1/2), sample(if(flip(1/2), append(ε, F0(ε)), pair(pair(ε, b), t))), if(flip(1/2), pair(pair(ε, l), i), pair(pair(ε, k), u)))), if(flip(7/24), pair(ε, 3), x)).<br>F2(x):=λx.F1(sample(if(or(flip(1/3), flip(1/2)), (pair(ε, 2) ∪ pair(ε, 1)), pair(ε, 4)))).
Newport & Aslin (2004)	btgdprkuli1	7d	8	100000 (20)	-301817	1	1	F0(x):=λx.insert(if(not(flip(5/24)), if(flip(1/4), pair(pair(ε, p), r), if(flip(1/3), pair(pair(ε, l), i), if(flip(1/2), x, pair(pair(ε, k), u)))), pair(pair(ε, b), t)), Fm1(ε)).<br>F1(x):=λx.sample((((Σ\(pair(x, p) ∪ pair(x, d)))\(((pair(ε, l) ∪ pair(ε, k)) ∪ pair(x, g)) ∪ ((pair(ε, r) ∪ pair(ε, i)) ∪ (pair(x, t) ∪ pair(x, b)))))\pair(ε, u))).<br>F2(x):=λx.F0(pair(pair(x, g), d)).
Newport & Aslin (2004)	btgdprkuli1	7d	8	1 (1)	-41.2835	1	0.05	F0(x):=λx.pair(pair(pair(Fm2(Fm1(ε)), k), 4), u).<br>F1(x):=λx.ε.<br>F2(x):=λx.ε.<br>F3(x):=λx.F0(ε).
Newport & Aslin (2004)	btgdprkuli1	7d	8	10 (9)	-146.479	0.01904	0.2	F0(x):=λx.insert(F2(ε), sample(Σ)).<br>F1(x):=λx.ε.<br>F2(x):=λx.if(flip(1/2), sample(Σ), pair(pair(ε, k), u)).<br>F3(x):=λx.F0(Fm1(ε)).
Newport & Aslin (2004)	btgdprkuli1	7d	8	100 (20)	-459.593	1	1	F0(x):=λx.pair(pair(ε, b), t).<br>F1(x):=λx.pair(ε, k).<br>F2(x):=λx.if(flip(1/2), if(flip(1/2), if(flip(1/2), pair(pair(x, l), i), pair(pair(ε, p), r)), pair(Fm1(ε), u)), if(flip(1/2), pair(pair(ε, g), d), Fm0(ε))).<br>F3(x):=λx.insert(Fm2(x), sample((if(flip(1/2), pair(x, 2), pair(ε, 1)) ∪ if(flip(1/2), pair(ε, 3), pair(ε, 4))))).

Newport & Aslin (2004)	btgdprkuli1	7d	8	1000 (20)	-3200.8	1	1	F0(x):=λx.pair(pair(ε, b), t).<br>F1(x):=λx.pair(x, k).<br>F2(x):=λx.if(flip(1/2), if(not(flip(3/8)), if(flip(1/2), pair(pair(ε, l), i), pair(pair(ε, p), r)), pair(F1(ε), u)), if(flip(1/2), pair(pair(insert(ε, x), g), d), F0(ε))).<br>F3(x):=λx.insert(Fm2(ε), sample((if(flip(1/2), pair(ε, 2), pair(ε, 1)) ∪ if(flip(1/2), pair(x, 3), pair(x, 4))))).
Newport & Aslin (2004)	btgdprkuli1	7d	8	10000 (20)	-30393	1	1	F0(x):=λx.sample(((((Σ\(pair(x, b) ∪ pair(ε, k)))\pair(ε, d))\pair(F1(ε), p))\((pair(ε, l) ∪ pair(ε, r)) ∪ ((pair(ε, t) ∪ (pair(ε, g) ∪ pair(ε, i))) ∪ pair(ε, u))))).<br>F1(x):=λx.ε.<br>F2(x):=λx.sample(if(flip(1/4), pair(pair(ε, k), u), if(not(flip(1/3)), if(flip(1/2), pair(pair(ε, p), r), pair(pair(ε, b), t)), append(pair(ε, l), pair(ε, i))))).<br>F3(x):=λx.insert(if(not(flip(5/24)), F2(x), pair(pair(ε, g), d)), F0(x)).
Newport & Aslin (2004)	btgdprkuli1	7d	8	100000 (20)	-301825	1	1	F0(x):=λx.sample(((((Σ\(pair(ε, b) ∪ pair(x, k)))\pair(F1(ε), d))\pair(ε, p))\((pair(x, l) ∪ (pair(ε, r) ∪ pair(ε, u))) ∪ (pair(x, t) ∪ (pair(ε, g) ∪ pair(ε, i)))))).<br>F1(x):=λx.ε.<br>F2(x):=λx.sample(if(flip(1/4), pair(pair(x, k), u), if(or(flip(1/3), flip(1/2)), if(flip(1/2), pair(pair(x, p), r), pair(pair(x, b), t)), append(pair(ε, l), pair(ε, i))))).<br>F3(x):=λx.insert(if(or(flip(1/24), not(flip(5/24))), Fm2(x), pair(pair(ε, g), d)), F0(ε)).
Morgan & Newport (1981)	ACDEF	5d	8	1 (1)	-20.9281	1	0.05555	F0(x):=λx.pair(pair(pair(pair(ε, A), E), C), D).
Morgan & Newport (1981)	ACDEF	5d	8	10 (9)	-91.9081	0.10714	0.72222	F0(x):=λx.append(if(flip(3/8), pair(ε, A), F0(ε)), sample((Σ\pair(ε, A)))).
Morgan & Newport (1981)	ACDEF	5d	8	100 (18)	-366.518	1	1	F0(x):=λx.append(pair(ε, A), append(append(if(flip(1/2), ε, pair(ε, D)), sample((pair(sample((pair(ε, C) ∪ pair(pair(ε, C), D))), F) ∪ pair(ε, E)))), if(flip(1/2), append(pair(ε, C), if(flip(1/2), ε, pair(ε, D))), ε))).
Morgan & Newport (1981)	ACDEF	5d	8	1000 (18)	-2907.19	1	1	F0(x):=λx.append(pair(ε, A), append(append(if(flip(1/2), ε, pair(ε, D)), sample((pair(sample((pair(ε, C) ∪ pair(pair(ε, C), D))), F) ∪ pair(ε, E)))), if(flip(1/2), append(pair(ε, C), if(flip(1/2), ε, pair(ε, D))), ε))).
Morgan & Newport (1981)	ACDEF	5d	8	10000 (18)	-27978.1	1	1	F0(x):=λx.append(pair(ε, A), append(append(if(flip(1/2), ε, pair(ε, D)), sample((pair(sample((pair(ε, C) ∪ pair(pair(ε, C), D))), F) ∪ pair(ε, E)))), if(flip(1/2), append(pair(ε, C), if(flip(1/2), ε, pair(ε, D))), ε))).
Morgan & Newport (1981)	ACDEF	5d	8	100000 (18)	-278441	1	1	F0(x):=λx.append(pair(ε, A), append(append(if(flip(1/2), ε, pair(ε, D)), sample((pair(sample((pair(ε, C) ∪ pair(pair(ε, C), D))), F) ∪ pair(ε, E)))), if(flip(1/2), append(pair(ε, C), if(flip(1/2), ε, pair(ε, D))), ε))).
Morgan & Newport (1981)	ACDEF	5d	8	1 (1)	-27.5527	1	0.05555	F0(x):=λx.ε.<br>F1(x):=λx.pair(pair(pair(pair(F0(ε), A), E), C), D).
Morgan & Newport (1981)	ACDEF	5d	8	10 (9)	-95.9254	0.10714	0.72222	F0(x):=λx.append(if(flip(3/8), x, F1(ε)), sample((Σ\x))).<br>F1(x):=λx.Fm0(pair(ε, A)).
Morgan & Newport (1981)	ACDEF	5d	8	100 (18)	-358.992	1	1	F0(x):=λx.if(flip(1/2), x, pair(x, D)).<br>F1(x):=λx.append(insert(F0(ε), pair(ε, A)), append(sample((pair(ε, E) ∪ pair(append(pair(ε, C), Fm0(ε)), F))), if(flip(1/2), ε, F0(pair(ε, C))))).
Morgan & Newport (1981)	ACDEF	5d	8	1000 (18)	-2899.66	1	1	F0(x):=λx.if(flip(1/2), x, pair(x, D)).<br>F1(x):=λx.append(insert(F0(ε), pair(ε, A)), append(sample((pair(ε, E) ∪ pair(append(pair(ε, C), Fm0(ε)), F))), if(flip(1/2), ε, F0(pair(ε, C))))).
Morgan & Newport (1981)	ACDEF	5d	8	10000 (18)	-27970.5	1	1	F0(x):=λx.if(flip(1/2), x, pair(x, D)).<br>F1(x):=λx.append(insert(F0(ε), pair(ε, A)), append(sample((pair(ε, E) ∪ pair(append(pair(ε, C), Fm0(ε)), F))), if(flip(1/2), ε, F0(pair(ε, C))))).
Morgan & Newport (1981)	ACDEF	5d	8	100000 (18)	-278434	1	1	F0(x):=λx.if(flip(1/2), x, pair(x, D)).<br>F1(x):=λx.append(insert(F0(ε), pair(ε, A)), append(sample((pair(ε, E) ∪ pair(append(pair(ε, C), Fm0(ε)), F))), if(flip(1/2), ε, F0(pair(ε, C))))).
Morgan & Newport (1981)	ACDEF	5d	8	1 (1)	-34.9883	1	0.05555	F0(x):=λx.pair(pair(ε, A), E).<br>F1(x):=λx.ε.<br>F2(x):=λx.pair(pair(F0(F1(ε)), C), D).
Morgan & Newport (1981)	ACDEF	5d	8	10 (9)	-103.766	0.10714	0.72222	F0(x):=λx.ε.<br>F1(x):=λx.append(if(flip(3/8), x, F2(Fm0(ε))), sample((Σ\x))).<br>F2(x):=λx.F1(pair(ε, A)).
Morgan & Newport (1981)	ACDEF	5d	8	100 (18)	-377.943	0.85714	1	F0(x):=λx.append(F1(pair(ε, A)), if(flip(1/2), F1(pair(x, C)), x)).<br>F1(x):=λx.append(x, if(flip(1/2), pair(ε, D), ε)).<br>F2(x):=λx.Fm0(insert(sample((pair(pair(ε, C), F) ∪ pair(ε, E))), Fm1(ε))).
Morgan & Newport (1981)	ACDEF	5d	8	1000 (18)	-2902.44	1	1	F0(x):=λx.append(F1(pair(ε, A)), append(if(flip(1/2), x, pair(ε, E)), sample(if(flip(1/2), F1(head(x)), ε)))).<br>F1(x):=λx.sample((x ∪ pair(x, D))).<br>F2(x):=λx.F0(pair(F1(pair(ε, C)), F)).
Morgan & Newport (1981)	ACDEF	5d	8	10000 (18)	-27973.3	1	1	F0(x):=λx.append(F1(pair(ε, A)), append(if(flip(1/2), x, pair(ε, E)), sample(if(flip(1/2), F1(head(x)), ε)))).<br>F1(x):=λx.sample((x ∪ pair(x, D))).<br>F2(x):=λx.F0(pair(F1(pair(ε, C)), F)).
Morgan & Newport (1981)	ACDEF	5d	8	100000 (18)	-278437	1	1	F0(x):=λx.append(F1(pair(ε, A)), append(if(flip(1/2), x, pair(ε, E)), sample(if(flip(1/2), F1(head(x)), ε)))).<br>F1(x):=λx.sample((x ∪ pair(x, D))).<br>F2(x):=λx.F0(pair(F1(pair(x, C)), F)).
Morgan & Newport (1981)	ACDEF	5d	8	1 (1)	-42.8814	1	0.05555	F0(x):=λx.pair(F2(ε), A).<br>F1(x):=λx.pair(pair(F0(ε), E), C).<br>F2(x):=λx.ε.<br>F3(x):=λx.pair(F1(ε), D).

Morgan & Newport (1981)	ACDEF	5d	8	10 (9)	-111.947	0.10714	0.72222	F0(x):=λx.append(if(flip(3/8), x, Fm3(Fm2(Fm1(ε)))), sample((Σ\x))).<br>F1(x):=λx.ε.<br>F2(x):=λx.ε.<br>F3(x):=λx.Fm0(pair(ε, A)).
Morgan & Newport (1981)	ACDEF	5d	8	100 (18)	-361.491	1	1	F0(x):=λx.if(flip(1/2), x, pair(x, D)).<br>F1(x):=λx.ε.<br>F2(x):=λx.append(Fm0(pair(ε, A)), if(flip(1/2), F0(pair(x, C)), x)).<br>F3(x):=λx.F2(if(flip(1/2), pair(F0(pair(ε, C)), F), pair(Fm1(ε), E))).
Morgan & Newport (1981)	ACDEF	5d	8	1000 (18)	-2902.16	1	1	F0(x):=λx.if(flip(1/2), x, pair(x, D)).<br>F1(x):=λx.ε.<br>F2(x):=λx.append(Fm0(pair(ε, A)), if(flip(1/2), F0(pair(x, C)), x)).<br>F3(x):=λx.F2(if(flip(1/2), pair(F0(pair(ε, C)), F), pair(Fm1(ε), E))).
Morgan & Newport (1981)	ACDEF	5d	8	10000 (18)	-27973	1	1	F0(x):=λx.if(flip(1/2), x, pair(x, D)).<br>F1(x):=λx.ε.<br>F2(x):=λx.append(Fm0(pair(ε, A)), if(flip(1/2), F0(pair(x, C)), x)).<br>F3(x):=λx.F2(if(flip(1/2), pair(F0(pair(ε, C)), F), pair(Fm1(ε), E))).
Morgan & Newport (1981)	ACDEF	5d	8	100000 (18)	-278436	1	1	F0(x):=λx.if(flip(1/2), x, pair(x, D)).<br>F1(x):=λx.ε.<br>F2(x):=λx.append(Fm0(pair(ε, A)), if(flip(1/2), F0(pair(x, C)), x)).<br>F3(x):=λx.F2(if(flip(1/2), pair(F0(pair(ε, C)), F), pair(Fm1(ε), E))).
Morgan et al. (1987)	ACDEFoua	7d	8	1 (1)	-56.9895	0.01111	0.05555	F0(x):=λx.append(if(flip(1/2), F0(ε), pair(pair(pair(ε, o), A), D)), pair(pair(pair(sample(Σ), i), C), D)).
Morgan et al. (1987)	ACDEFoua	7d	8	10 (5)	-150.878	0.11111	0.44444	F0(x):=λx.append(pair(pair(ε, o), A), append(sample((pair(ε, D)∪ε)), append(if(flip(1/2), pair(ε, u), pair(pair(pair(ε, a), i), C)), append(sample(Σ), if(flip(1/2), pair(pair(ε, i), C), ε))))).
Morgan et al. (1987)	ACDEFoua	7d	8	100 (18)	-644.047	0.5	0.66666	F0(x):=λx.append(pair(pair(ε, o), A), append(append(if(flip(1/2), ε, pair(ε, D)), sample((pair(ε, u)∪pair(pair(pair(ε, a), i), C)))), append(if(flip(1/2), pair(ε, E), pair(if(flip(1/2), pair(ε, D), ε), F)), sample((pair(pair(ε, i), C)∪ε))))).
Morgan et al. (1987)	ACDEFoua	7d	8	1000 (18)	-3738.21	1	0.66666	F0(x):=λx.append(pair(pair(ε, o), A), append(sample(if(flip(1/2), pair(pair(if(flip(1/2), pair(ε, D), ε), u), E), append(pair(pair(pair(sample((ε∪pair(ε, D))), a), i), C), pair(sample((pair(ε, D)∪ε)), F)))), sample(if(flip(1/2), pair(pair(ε, i), C), D), ε)))).
Morgan et al. (1987)	ACDEFoua	7d	8	10000 (18)	-35988.8	1	0.66666	F0(x):=λx.append(pair(pair(ε, o), A), append(sample(if(flip(1/2), pair(pair(if(flip(1/2), pair(ε, D), ε), u), E), append(pair(pair(pair(sample((ε∪pair(ε, D))), a), i), C), pair(sample((pair(ε, D)∪ε)), F)))), sample(if(flip(1/2), pair(pair(ε, i), C), D), ε)))).
Morgan et al. (1987)	ACDEFoua	7d	8	100000 (18)	-359792	1	0.66666	F0(x):=λx.append(pair(pair(ε, o), A), append(sample(if(flip(1/2), pair(pair(if(flip(1/2), pair(ε, D), ε), u), E), append(pair(pair(pair(sample((ε∪pair(ε, D))), a), i), C), pair(sample((pair(ε, D)∪ε)), F)))), sample(if(flip(1/2), pair(pair(ε, i), C), D), ε)))).
Morgan et al. (1987)	ACDEFoua	7d	8	1 (1)	-59.6607	0.01234	0.05555	F0(x):=λx.append(pair(x, D), pair(pair(sample(Σ), i), C)).<br>F1(x):=λx.Fm0(F0(pair(pair(ε, o), A))).
Morgan et al. (1987)	ACDEFoua	7d	8	10 (5)	-150.81	1	0.33333	F0(x):=λx.append(pair(pair(ε, o), A), if(flip(1/2), pair(pair(pair(ε, D), u), E), append(pair(pair(if(flip(1/2), pair(ε, D), ε), a), i), pair(pair(ε, C), F)))).<br>F1(x):=λx.append(F0(ε), if(flip(1/2), pair(pair(ε, i), C), ε)).
Morgan et al. (1987)	ACDEFoua	7d	8	100 (18)	-403.483	1	1	F0(x):=λx.append(pair(pair(ε, o), A), append(if(flip(1/2), x, ε), if(flip(1/2), pair(pair(ε, u), E), append(pair(pair(pair(ε, a), i), C), pair(if(flip(1/2), x, ε), F))))).<br>F1(x):=λx.append(F0(pair(ε, D)), sample(if(flip(1/2), insert(if(flip(1/2), pair(ε, D), ε), pair(pair(ε, i), C)), ε))).
Morgan et al. (1987)	ACDEFoua	7d	8	1000 (18)	-2912.19	1	1	F0(x):=λx.append(pair(pair(ε, o), A), append(if(flip(1/2), x, ε), if(flip(1/2), pair(pair(ε, u), E), append(pair(pair(pair(ε, a), i), C), pair(if(flip(1/2), x, ε), F))))).<br>F1(x):=λx.append(F0(pair(ε, D)), sample(if(flip(1/2), insert(if(flip(1/2), pair(ε, D), ε), pair(pair(ε, i), C)), ε))).
Morgan et al. (1987)	ACDEFoua	7d	8	10000 (18)	-28039	1	1	F0(x):=λx.append(pair(pair(ε, o), A), append(if(flip(1/2), x, ε), if(flip(1/2), pair(pair(ε, u), E), append(pair(pair(pair(ε, a), i), C), pair(if(flip(1/2), x, ε), F))))).<br>F1(x):=λx.append(F0(pair(ε, D)), sample(if(flip(1/2), insert(if(flip(1/2), pair(ε, D), ε), pair(pair(ε, i), C)), ε))).
Morgan et al. (1987)	ACDEFoua	7d	8	100000 (18)	-279161	1	1	F0(x):=λx.append(pair(pair(ε, o), A), append(if(flip(1/2), x, ε), if(flip(1/2), pair(pair(ε, u), E), append(pair(pair(pair(ε, a), i), C), pair(if(flip(1/2), x, ε), F))))).<br>F1(x):=λx.append(F0(pair(ε, D)), sample(if(flip(1/2), insert(if(flip(1/2), pair(x, D), ε), pair(pair(ε, i), C)), ε))).
Morgan et al. (1987)	ACDEFoua	7d	8	1 (1)	-72.8652	0	0	F0(x):=λx.append(pair(if(flip(1/2), pair(F0(sample(Σ)), C), Fm1(ε)), D), pair(x, i)).<br>F1(x):=λx.pair(pair(ε, o), A).<br>F2(x):=λx.Fm0(ε).
Morgan et al. (1987)	ACDEFoua	7d	8	10 (5)	-155.454	0.34615	0.61111	F0(x):=λx.append(if(flip(5/12), x, pair(pair(x, i), C)), if(flip(1/2), ε, Fm1(ε))).<br>F1(x):=λx.if(flip(1/2), pair(Fm0(x), F), append(pair(ε, D), x)).<br>F2(x):=λx.Fm0(append(pair(pair(ε, o), A), Fm1(sample(if(flip(1/2), pair(pair(ε, u), E), pair(ε, a)))))).
Morgan et al. (1987)	ACDEFoua	7d	8	100 (18)	-439.185	0.75	1	F0(x):=λx.append(append(pair(ε, i), pair(ε, C)), sample(((pair(pair(ε, D), F)∪head(pair(x, D)))∪x))).<br>F1(x):=λx.append(pair(pair(ε, o), A), append(if(flip(1/2), ε, pair(ε, D)), if(flip(1/2), append(x, F0(pair(ε, F))), pair(pair(ε, u), E)))).<br>F2(x):=λx.append(Fm1(pair(ε, a)), if(flip(1/2), ε, Fm0(ε))).

Morgan et al. (1987)	ACDEFoua	7d	8	1000 (18)	-2905.74	1	1	F0(x):=λx.if(not(or(empty(x), flip(1/2))), x, Fm1(pair(append(if(empty(x), pair(ϵ, a), x), pair(ϵ, i)), C))).<br>F1(x):=λx.if(flip(1/2), x, pair(x, D)).<br>F2(x):=λx.F0(append(F1(pair(pair(ϵ, o), A)), if(flip(1/2), pair(Fm0(ϵ), F), pair(pair(ϵ, u), E)))).
Morgan et al. (1987)	ACDEFoua	7d	8	10000 (18)	-28032.5	1	1	F0(x):=λx.if(not(or(empty(x), flip(1/2))), x, Fm1(pair(append(if(empty(x), pair(ϵ, a), x), pair(ϵ, i)), C))).<br>F1(x):=λx.if(flip(1/2), x, pair(x, D)).<br>F2(x):=λx.F0(append(F1(pair(pair(ϵ, o), A)), if(flip(1/2), pair(Fm0(ϵ), F), pair(pair(ϵ, u), E)))).
Morgan et al. (1987)	ACDEFoua	7d	8	100000 (18)	-279154	1	1	F0(x):=λx.if(not(or(empty(x), flip(1/2))), x, Fm1(pair(append(if(empty(x), pair(ϵ, a), x), pair(ϵ, i)), C))).<br>F1(x):=λx.if(flip(1/2), x, pair(x, D)).<br>F2(x):=λx.F0(append(F1(pair(pair(ϵ, o), A)), if(flip(1/2), pair(Fm0(ϵ), F), pair(pair(ϵ, u), E)))).
Morgan et al. (1987)	ACDEFoua	7d	8	1 (1)	-77.0689	0.01234	0.05555	F0(x):=λx.append(pair(x, D), sample(Σ)).<br>F1(x):=λx.pair(pair(x, i), C).<br>F2(x):=λx.Fm1(Fm0(x)).<br>F3(x):=λx.F2(F2(pair(pair(ϵ, o), A))).
Morgan et al. (1987)	ACDEFoua	7d	8	10 (5)	-161.581	1	0.66666	F0(x):=λx.if(flip(1/2), x, pair(x, D)).<br>F1(x):=λx.append(Fm0(pair(x, A)), if(flip(1/2), append(pair(ϵ, u), pair(ϵ, E)), F2(ϵ))).<br>F2(x):=λx.pair(F0(pair(pair(pair(ϵ, a), i), C)), F).<br>F3(x):=λx.append(F1(pair(ϵ, o)), sample(if(flip(1/2), pair(pair(ϵ, i), C), ϵ))).
Morgan et al. (1987)	ACDEFoua	7d	8	100 (18)	-454.003	0.75	1	F0(x):=λx.pair(pair(ϵ, o), A).<br>F1(x):=λx.if(not((ϵ==x)), append(x, if(flip(1/2), ϵ, pair(ϵ, D))), pair(pair(pair(ϵ, a), i), C)).<br>F2(x):=λx.F1(append(Fm1(Fm0(ϵ)), if(flip(1/2), x, pair(pair(x, i), C)))).<br>F3(x):=λx.F2(sample(if(flip(1/2), pair(pair(ϵ, u), E), pair(Fm1(Fm1(ϵ)), F)))).
Morgan et al. (1987)	ACDEFoua	7d	8	1000 (18)	-3096.79	0.81818	1	F0(x):=λx.if(empty(Fm1(ϵ)), pair(pair(ϵ, u), E), pair(x, a)).<br>F1(x):=λx.if(not(flip(1/3)), pair(pair(pair(if(flip(1/2), pair(ϵ, D), ϵ), F), i), C), x).<br>F2(x):=λx.append(pair(pair(tail(ϵ), o), A), if(flip(1/2), pair(x, D), ϵ)).<br>F3(x):=λx.append(Fm2(ϵ), if(flip(1/4), pair(pair(x, u), E), append(pair(pair(Fm0(x), i), C), sample((pair(Fm1(ϵ), D) ∪ (pair(if(flip(1/2), pair(ϵ, D), ϵ), F) ∪ Fm1(x))))))).
Morgan et al. (1987)	ACDEFoua	7d	8	10000 (18)	-28073.8	1	1	F0(x):=λx.pair(x, F).<br>F1(x):=λx.append(pair(pair(ϵ, i), C), if(flip(1/2), ϵ, pair(ϵ, D))).<br>F2(x):=λx.append(pair(pair(ϵ, o), A), append(sample(if(flip(1/4), pair(x, F), if(flip(1/3), F0(pair(x, D)), pair(pair(if(flip(1/2), pair(ϵ, D), ϵ), u), E)))), sample((F1(ϵ) ∪ ϵ)))).<br>F3(x):=λx.F2(pair(pair(pair(if(flip(1/2), x, pair(ϵ, D)), a), i), C)).
Morgan et al. (1987)	ACDEFoua	7d	8	100000 (18)	-279163	1	1	F0(x):=λx.if(flip(1/2), pair(x, D), ϵ).<br>F1(x):=λx.append(append(Fm2(ϵ), if(flip(1/2), pair(pair(F0(ϵ), u), E), append(pair(Fm0(ϵ), a), pair(insert(F0(ϵ), x), F)))), if(flip(1/2), append(x, F0(ϵ)), ϵ)).<br>F2(x):=λx.pair(pair(ϵ, o), A).<br>F3(x):=λx.F1(pair(pair(ϵ, i), C)).
Simple Braine (1963)	bdrgknfGm	7d	8	1 (1)	-13.6139	1	0.04	F0(x):=λx.pair(pair(ϵ, f), n).
Simple Braine (1963)	bdrgknfGm	7d	8	10 (10)	-192.596	0.00952	0.08	F0(x):=λx.if(flip(1/2), append(pair(ϵ, G), append(sample(Σ), pair(sample(Σ), f))), append(pair(ϵ, f), sample(Σ))).
Simple Braine (1963)	bdrgknfGm	7d	8	100 (32)	-847.883	0.06372	0.6	F0(x):=λx.sample(if(flip(1/4), (pair(pair(pair(if(flip(if(flip(1/24), 7/24, 5/24)), Fm0(ϵ), ϵ), f), r), g) ∪ pair(pair(pair(ϵ, f), b), d)), if(flip(5/24), pair(pair(ϵ, f), k), if(not(flip(1/6)), append(append(pair(ϵ, G), sample(Σ)), sample(Σ)), pair(pair(ϵ, f), n))))).
Simple Braine (1963)	bdrgknfGm	7d	8	1000 (78)	-7822.84	0.3125	0.52	F0(x):=λx.if(flip(11/24), append(pair(if(flip(1/3), F0(ϵ), ϵ), G), insert(sample(Σ), sample((if(not(flip(5/24)), pair(ϵ, m), Σ) ∪ pair(ϵ, l))))), append(pair(ϵ, f), sample((pair(pair(ϵ, b), d) ∪ if(flip(1/3), pair(ϵ, n), if(flip(1/2), pair(pair(ϵ, r), g), pair(ϵ, k))))))).
Simple Braine (1963)	bdrgknfGm	7d	8	10000 (85)	-77350.5	0.3125	0.52	F0(x):=λx.if(flip(11/24), append(pair(if(flip(1/3), F0(ϵ), ϵ), G), insert(sample(Σ), sample((if(not(flip(5/24)), pair(ϵ, m), Σ) ∪ pair(ϵ, l))))), append(pair(ϵ, f), sample((pair(pair(ϵ, b), d) ∪ if(flip(1/3), pair(ϵ, n), if(flip(1/2), pair(pair(ϵ, r), g), pair(ϵ, k))))))).
Simple Braine (1963)	bdrgknfGm	7d	8	100000 (85)	-757342	0.3125	0.52	F0(x):=λx.if(flip(11/24), append(pair(if(flip(1/3), F0(ϵ), ϵ), G), insert(sample(Σ), sample((if(not(flip(5/24)), pair(ϵ, m), Σ) ∪ pair(ϵ, l))))), append(pair(ϵ, f), sample((pair(pair(ϵ, b), d) ∪ if(flip(1/3), pair(ϵ, n), if(flip(1/2), pair(pair(ϵ, r), g), pair(ϵ, k))))))).
Simple Braine (1963)	bdrgknfGm	7d	8	1 (1)	-20.2385	1	0.04	F0(x):=λx.ϵ.<br>F1(x):=λx.pair(pair(Fm0(ϵ), f), n).
Simple Braine (1963)	bdrgknfGm	7d	8	10 (10)	-193.833	0.00476	0.04	F0(x):=λx.insert(if(flip(1/2), pair(pair(ϵ, f), g), append(pair(ϵ, G), pair(sample(Σ), f))), sample(Σ)).<br>F1(x):=λx.F0(ϵ).
Simple Braine (1963)	bdrgknfGm	7d	8	100 (32)	-809.334	0.42307	0.6	F0(x):=λx.if(flip(5/12), append(pair(ϵ, G), sample(if(flip(5/12), pair(ϵ, l), if(flip(1/2), append(pair(ϵ, y), if(flip(5/12), ϵ, pair(sample(Σ), f))), pair(ϵ, m))))), pair(ϵ, f)).<br>F1(x):=λx.append(F0(x), sample(if(flip(3/8), if(flip(1/2), if(flip(1/2), pair(x, s), pair(ϵ, e)), pair(pair(ϵ, b), d)), ((pair(ϵ, k) ∪ if(flip(1/2), pair(ϵ, n), pair(ϵ, w))) ∪ pair(pair(x, r), g))))).
Simple Braine (1963)	bdrgknfGm	7d	8	1000 (78)	-6789.92	0.4	0.76	F0(x):=λx.insert(sample((((Σ\pair(ϵ, m))\pair(ϵ, f))\(pair(ϵ, d) ∪ (pair(ϵ, l) ∪ ((pair(ϵ, G) ∪ pair(ϵ, g)) ∪ pair(ϵ, y)))))), sample(if(not(flip(5/24)), (if(flip(1/2), pair(x, y), pair(x, l)) ∪ pair(ϵ, f)), pair(x, m)))).<br>F1(x):=λx.if(flip(5/12), F1(if(empty(x), if(flip(1/2), pair(ϵ, g), pair(pair(ϵ, f), n)), pair(pair(pair(ϵ, f), b), d))), append(if(flip(if((x==head(x)), 1/24, 1/2)), ϵ, Fm0(pair(sample((x ∪ ϵ)), G))), if(and((x==ϵ), flip(1/8)), pair(pair(ϵ, f), r), x))).

Simple Braine (1963)	bdrgknfGm	7d	8	10000 (85)	-65396.5	0.4	0.76	F0(x):=λx.insert(sample((((Σ\pair(ε, l))\pair(ε, g))\(head(x)∪(pair(ε, m)∪((pair(ε, d)∪pair(ε, b))∪pair(ε, y)))))), sample(if(not(flip(5/24)), (if(flip(1/2), pair(x, y), pair(x, l))∪pair(ε, f)), pair(x, m)))).<br>F1(x):=λx.if(flip(11/24), F1(if(empty(x), if(flip(1/2), pair(ε, g), pair(pair(ε, f), n)), pair(pair(pair(ε, f), b), d))), append(if(flip(if((x==head(x)), 1/24, 1/2)), ε, Fm0(pair(sample((ε∪(x\Σ))), G))), if(and(empty(x), flip(1/8)), pair(pair(x, f), r), x))).
Simple Braine (1963)	bdrgknfGm	7d	8	100000 (85)	-539116	0.41935	1	F0(x):=λx.append(pair(x, f), if(or(flip(1/2), flip(1/2)), sample(if(flip(1/3), pair(ε, n), if(flip(1/2), pair(ε, k), append(pair(ε, r), pair(ε, g))))), pair(pair(ε, b), d))).<br>F1(x):=λx.if(not(flip(3/8)), Fm0(if(flip(5/24), Fm1(ε), ε)), append(pair(ε, G), append(sample(if(flip(1/3), pair(ε, m), (pair(ε, l)∪pair(ε, y)))), sample(((((Σ\pair(ε, r))\pair(ε, y))\pair(ε, k))\(pair(ε, f)∪pair(ε, n))))))).
Simple Braine (1963)	bdrgknfGm	7d	8	1 (1)	-27.674	1	0.04	F0(x):=λx.ε.<br>F1(x):=λx.ε.<br>F2(x):=λx.pair(pair(F0(F1(ε)), f), n).
Simple Braine (1963)	bdrgknfGm	7d	8	10 (10)	-203.571	0.00476	0.04	F0(x):=λx.if(flip(1/2), x, pair(insert(sample(Σ), pair(ε, G)), f)).<br>F1(x):=λx.insert(F0(pair(pair(ε, f), g)), sample(Σ)).<br>F2(x):=λx.Fm1(ε).
Simple Braine (1963)	bdrgknfGm	7d	8	100 (32)	-736.856	0.44	0.88	F0(x):=λx.append(pair(ε, G), F1(pair(ε, y))).<br>F1(x):=λx.append(if(flip(1/2), x, sample(if(flip(1/2), pair(ε, m), pair(ε, l)))), sample(if(flip(7/24), pair(ε, s), Σ))).<br>F2(x):=λx.if(flip(1/4), Fm0(ε), append(pair(if(flip(5/24), Fm0(ε), ε), f), sample((pair(pair(ε, r), g)∪if(not(flip(7/24)), (pair(ε, k)∪pair(ε, n)), Fm1(pair(ε, b))))))).
Simple Braine (1963)	bdrgknfGm	7d	8	1000 (78)	-6490.31	0.22413	0.92	F0(x):=λx.append(pair(ε, G), append(sample(Σ), if(flip(3/8), if(flip(1/2), F2(head(x)), pair(x, n)), head(if(flip(1/24), x, x))))).<br>F1(x):=λx.pair(sample(if(flip(1/3), pair(ε, s), if(flip(1/2), pair(ε, w), pair(ε, e)))), f).<br>F2(x):=λx.if(and(empty(x), flip(1/2)), F0(Fm1(x)), append(pair(x, f), sample(if(flip(1/2), if(flip(11/24), pair(pair(ε, b), d), pair(pair(ε, r), g)), Σ)))).
Simple Braine (1963)	bdrgknfGm	7d	8	10000 (85)	-51329.3	0.64705	1	F0(x):=λx.sample((if(flip(1/4), append(Fm2(x), Fm0(x)), pair(x, k))∪if(flip(1/2), pair(x, n), append(pair(x, b), pair(ε, d))))).<br>F1(x):=λx.append(x, sample(((((Σ\pair(x, w))\(pair(ε, g)∪head(pair(x, s))))\pair(ε, f))\((pair(ε, d)∪(pair(ε, b)∪(pair(ε, n)∪(Σ\((Σ\pair(ε, k))\pair(ε, r))))))∪(pair(x, e)∪pair(ε, G)))))).<br>F2(x):=λx.sample(if(and(empty(x), not(flip(11/24))), Fm0(pair(x, f)), if(not(and((x==ε), flip(5/12))), append(pair(ε, G), Fm1(F1(ε))), append(pair(pair(if(not(flip(1/4)), ε, Fm2(sample(Σ))), f), r), pair(ε, g))))).
Simple Braine (1963)	bdrgknfGm	7d	8	100000 (85)	-504073	0.64705	1	F0(x):=λx.sample((if(flip(1/4), append(F2(x), Fm0(x)), pair(x, k))∪if(flip(1/2), pair(sample(x), n), append(pair(x, b), pair(ε, d))))).<br>F1(x):=λx.append(x, sample(((((Σ\pair(x, w))\(pair(ε, g)∪head(pair(x, s))))\pair(ε, f))\((pair(ε, d)∪(pair(ε, b)∪(pair(ε, n)∪(Σ\((Σ\pair(ε, k))\pair(ε, r))))))∪(pair(x, e)∪pair(ε, G)))))).<br>F2(x):=λx.sample(if(and(empty(x), not(flip(11/24))), Fm0(pair(ε, f)), if(not(and((ε==x), flip(5/12))), append(pair(ε, G), Fm1(F1(ε))), append(pair(pair(if(or(not(flip(1/4)), flip(1/8)), ε, Fm2(pair(ε, G))), f), r), pair(ε, g))))).
Simple Braine (1963)	bdrgknfGm	7d	8	1 (1)	-35.5672	1	0.04	F0(x):=λx.pair(ε, f).<br>F1(x):=λx.F2(ε).<br>F2(x):=λx.Fm0(ε).<br>F3(x):=λx.pair(Fm1(ε), n).
Simple Braine (1963)	bdrgknfGm	7d	8	10 (10)	-212.352	0.00952	0.08	F0(x):=λx.sample(Σ).<br>F1(x):=λx.if(flip(1/2), append(append(pair(ε, G), Fm0(ε)), pair(x, f)), append(pair(ε, f), x)).<br>F2(x):=λx.Fm1(sample(Σ)).<br>F3(x):=λx.Fm2(ε).
Simple Braine (1963)	bdrgknfGm	7d	8	100 (32)	-764.764	0.19444	0.72	F0(x):=λx.if(flip(1/2), pair(ε, f), append(pair(ε, G), if(flip(7/24), pair(Fm2(ε), m), if(flip(1/2), append(x, pair(sample(Σ), f)), x)))).<br>F1(x):=λx.if(flip(1/2), pair(ε, l), pair(ε, y)).<br>F2(x):=λx.if(flip(1/2), ε, ε).<br>F3(x):=λx.append(F0(F1(ε)), sample(if(flip(1/4), pair(pair(ε, r), g), if(flip(1/8), pair(pair(if(flip(1/2), ε, ε), b), d), Σ)))).
Simple Braine (1963)	bdrgknfGm	7d	8	1000 (78)	-5187.7	0.63333	1	F0(x):=λx.if(flip(1/3), pair(x, s), sample((pair(ε, w)∪ε))).<br>F1(x):=λx.sample(if(flip(1/4), pair(ε, r), ε)).<br>F2(x):=λx.append(append(pair(ε, G), sample(if(or(flip(11/24), flip(3/8)), if(flip(1/2), pair(ε, l), pair(head(ε), y)), pair(ε, m)))), head(pair(x, e))).<br>F3(x):=λx.if(flip(5/12), F2(F0(ε)), append(pair(if(flip(5/24), Fm3(x), x), f), append(Fm1(x), sample(if((ε==Fm1(ε)), if(flip(7/24), pair(x, n), (pair(pair(x, b), d)∪pair(ε, k))), pair(ε, g)))))).
Simple Braine (1963)	bdrgknfGm	7d	8	10000 (85)	-50222.6	0.69444	1	F0(x):=λx.if(flip(1/3), x, sample((pair(ε, e)∪pair(ε, w)))).<br>F1(x):=λx.sample(if(and(flip(1/2), flip(1/2)), pair(append(ε, ε), r), ε)).<br>F2(x):=λx.append(append(pair(ε, G), sample(if(or(flip(1/2), flip(3/8)), if(flip(1/2), pair(ε, l), pair(ε, y)), pair(ε, m)))), x).<br>F3(x):=λx.if(flip(3/8), F2(head(F0(pair(append(x, ε), s)))), append(pair(if(flip(1/2), if(flip(1/2), ε, F3(x)), ε), f), append(Fm1(x), sample(if((x==Fm1(ε)), if(flip(7/24), pair(ε, n), (pair(pair(ε, b), d)∪pair(ε, k))), pair(x, g)))))).
Simple Braine (1963)	bdrgknfGm	7d	8	100000 (85)	-474623	0.37349	0.88	F0(x):=λx.append(sample(if(and(not(or((pair(ε, w)==x), (x==pair(ε, s)))), not((pair(ε, e)==x))), pair(Fm1(x), f), pair(Fm1(x), G))), if((x==pair(ε, n)), if(flip(1/2), x, pair(ε, k)), insert(x, if((pair(ε, g)==x), pair(ε, r), Fm2(ε))))).<br>F1(x):=λx.if(flip(1/6), Fm3(x), ε).<br>F2(x):=λx.if(flip(1/4), pair(x, y), if(flip(1/3), pair(x, l), if(flip(1/2), pair(tail(Fm2(x)), b), pair(ε, m)))).<br>F3(x):=λx.Fm0(sample(((((((Σ\(pair(ε, b)∪pair(ε, r)))\pair(ε, k))\pair(ε, m))\pair(ε, l))\((pair(ε, y)∪pair(ε, f))∪pair(ε, G)))\x))).
ABA (Marcus 1999 et al.)	gGtTnNlL	4h	8	1 (1)	-17.6514	1	0	F0(x):=λx.pair(pair(pair(ε, n), T), n).
ABA (Marcus 1999 et al.)	gGtTnNlL	4h	8	10 (10)	-74.3092	1	1	F0(x):=λx.append(x, if(empty(x), Fm0(sample(Σ)), insert(x, sample(Σ)))).

ABA (Marcus 1999 et al.)	gGtTnNlL	4h	8	100 (51)	-450.417	1	1	$F0(x)$:=λx.append(x, if(empty(x), Fm0(sample(Σ)), insert(x, sample(Σ)))).
ABA (Marcus 1999 et al.)	gGtTnNlL	4h	8	1000 (64)	-4211.49	1	1	$F0(x)$:=λx.append(x, if(empty(x), Fm0(sample(Σ)), insert(x, sample(Σ)))).
ABA (Marcus 1999 et al.)	gGtTnNlL	4h	8	10000 (64)	-41822.2	1	1	$F0(x)$:=λx.append(x, if(empty(x), Fm0(sample(Σ)), insert(x, sample(Σ)))).
ABA (Marcus 1999 et al.)	gGtTnNlL	4h	8	100000 (64)	-417930	1	1	$F0(x)$:=λx.append(x, if((ϵ==x), Fm0(sample(Σ)), insert(x, sample(Σ)))).
ABA (Marcus 1999 et al.)	gGtTnNlL	4h	8	1 (1)	-24.276	1	0	$F0(x)$:=λx.pair(pair(ϵ, n), T).<br>$F1(x)$:=λx.pair(F0(ϵ), n).
ABA (Marcus 1999 et al.)	gGtTnNlL	4h	8	10 (10)	-66.8328	1	1	$F0(x)$:=λx.append(x, append(sample(Σ), x)).<br>$F1(x)$:=λx.Fm0(sample(Σ)).
ABA (Marcus 1999 et al.)	gGtTnNlL	4h	8	100 (51)	-442.94	1	1	$F0(x)$:=λx.append(x, append(sample(Σ), x)).<br>$F1(x)$:=λx.Fm0(sample(Σ)).
ABA (Marcus 1999 et al.)	gGtTnNlL	4h	8	1000 (64)	-4204.01	1	1	$F0(x)$:=λx.append(x, append(sample(Σ), x)).<br>$F1(x)$:=λx.Fm0(sample(Σ)).
ABA (Marcus 1999 et al.)	gGtTnNlL	4h	8	10000 (64)	-41814.8	1	1	$F0(x)$:=λx.append(x, append(sample(Σ), x)).<br>$F1(x)$:=λx.Fm0(sample(Σ)).
ABA (Marcus 1999 et al.)	gGtTnNlL	4h	8	100000 (64)	-417922	1	1	$F0(x)$:=λx.append(x, append(sample(Σ), x)).<br>$F1(x)$:=λx.Fm0(sample(Σ)).
ABA (Marcus 1999 et al.)	gGtTnNlL	4h	8	1 (1)	-31.7116	1	0	$F0(x)$:=λx.pair(F1(ϵ), n).<br>$F1(x)$:=λx.pair(pair(ϵ, n), T).<br>$F2(x)$:=λx.Fm0(ϵ).
ABA (Marcus 1999 et al.)	gGtTnNlL	4h	8	10 (10)	-74.2683	1	1	$F0(x)$:=λx.sample(Σ).<br>$F1(x)$:=λx.append(append(x, sample(Σ)), x).<br>$F2(x)$:=λx.Fm1(F0(ϵ)).
ABA (Marcus 1999 et al.)	gGtTnNlL	4h	8	100 (51)	-450.376	1	1	$F0(x)$:=λx.sample(Σ).<br>$F1(x)$:=λx.append(append(x, sample(Σ)), x).<br>$F2(x)$:=λx.Fm1(F0(ϵ)).
ABA (Marcus 1999 et al.)	gGtTnNlL	4h	8	1000 (64)	-4211.45	1	1	$F0(x)$:=λx.sample(Σ).<br>$F1(x)$:=λx.append(append(x, sample(Σ)), x).<br>$F2(x)$:=λx.Fm1(F0(ϵ)).
ABA (Marcus 1999 et al.)	gGtTnNlL	4h	8	10000 (64)	-41822.2	1	1	$F0(x)$:=λx.sample(Σ).<br>$F1(x)$:=λx.append(append(x, sample(Σ)), x).<br>$F2(x)$:=λx.Fm1(F0(ϵ)).
ABA (Marcus 1999 et al.)	gGtTnNlL	4h	8	100000 (64)	-417930	1	1	$F0(x)$:=λx.append(append(x, sample(Σ)), x).<br>$F1(x)$:=λx.F0(sample(Σ)).<br>$F2(x)$:=λx.F1(x).
ABA (Marcus 1999 et al.)	gGtTnNlL	4h	8	1 (1)	-39.6047	1	0	$F0(x)$:=λx.pair(pair(ϵ, n), T).<br>$F1(x)$:=λx.pair(F0(F2(ϵ)), n).<br>$F2(x)$:=λx.ϵ.<br>$F3(x)$:=λx.Fm1(ϵ).
ABA (Marcus 1999 et al.)	gGtTnNlL	4h	8	10 (10)	-82.1615	1	1	$F0(x)$:=λx.F1(sample(Σ)).<br>$F1(x)$:=λx.append(append(x, sample(Σ)), x).<br>$F2(x)$:=λx.Fm0(ϵ).<br>$F3(x)$:=λx.F2(ϵ).
ABA (Marcus 1999 et al.)	gGtTnNlL	4h	8	100 (51)	-458.269	1	1	$F0(x)$:=λx.F1(sample(Σ)).<br>$F1(x)$:=λx.append(append(x, sample(Σ)), x).<br>$F2(x)$:=λx.Fm0(ϵ).<br>$F3(x)$:=λx.F2(ϵ).
ABA (Marcus 1999 et al.)	gGtTnNlL	4h	8	1000 (64)	-4219.34	1	1	$F0(x)$:=λx.F1(sample(Σ)).<br>$F1(x)$:=λx.append(append(x, sample(Σ)), x).<br>$F2(x)$:=λx.Fm0(ϵ).<br>$F3(x)$:=λx.F2(ϵ).
ABA (Marcus 1999 et al.)	gGtTnNlL	4h	8	10000 (64)	-41830.1	1	1	$F0(x)$:=λx.F1(sample(Σ)).<br>$F1(x)$:=λx.append(append(x, sample(Σ)), x).<br>$F2(x)$:=λx.Fm0(ϵ).<br>$F3(x)$:=λx.F2(ϵ).
ABA (Marcus 1999 et al.)	gGtTnNlL	4h	8	100000 (64)	-417937	1	1	$F0(x)$:=λx.F1(sample(Σ)).<br>$F1(x)$:=λx.append(append(x, sample(Σ)), x).<br>$F2(x)$:=λx.Fm0(ϵ).<br>$F3(x)$:=λx.F2(ϵ).

ABB (Marcus 1999 et al.)	gGtTnNlL	4h	8	1 (1)	-17.6514	1	0	F0(x):=λx.pair(pair(pair(ε, L), N), N).
ABB (Marcus 1999 et al.)	gGtTnNlL	4h	8	10 (8)	-73.6614	1	1	F0(x):=λx.append(if((head(x)==x), F0(append(sample(Σ), x)), ε), x).
ABB (Marcus 1999 et al.)	gGtTnNlL	4h	8	100 (54)	-448.807	1	1	F0(x):=λx.append(if(empty(x), Fm0(sample(Σ)), sample(Σ)), append(x, x)).
ABB (Marcus 1999 et al.)	gGtTnNlL	4h	8	1000 (64)	-4209.88	1	1	F0(x):=λx.append(if(empty(x), Fm0(sample(Σ)), sample(Σ)), append(x, x)).
ABB (Marcus 1999 et al.)	gGtTnNlL	4h	8	10000 (64)	-41820.6	1	1	F0(x):=λx.append(if(empty(x), Fm0(sample(Σ)), sample(Σ)), append(x, x)).
ABB (Marcus 1999 et al.)	gGtTnNlL	4h	8	100000 (64)	-417928	1	1	F0(x):=λx.append(sample(if(empty(x), Fm0(sample(Σ)), Σ)), append(x, x)).
ABB (Marcus 1999 et al.)	gGtTnNlL	4h	8	1 (1)	-24.276	1	0	F0(x):=λx.pair(pair(ε, L), N).<br>F1(x):=λx.pair(F0(ε), N).
ABB (Marcus 1999 et al.)	gGtTnNlL	4h	8	10 (8)	-66.8328	1	1	F0(x):=λx.append(append(sample(Σ), x), x).<br>F1(x):=λx.Fm0(sample(Σ)).
ABB (Marcus 1999 et al.)	gGtTnNlL	4h	8	100 (54)	-442.94	1	1	F0(x):=λx.append(append(sample(Σ), x), x).<br>F1(x):=λx.Fm0(sample(Σ)).
ABB (Marcus 1999 et al.)	gGtTnNlL	4h	8	1000 (64)	-4204.01	1	1	F0(x):=λx.append(append(sample(Σ), x), x).<br>F1(x):=λx.Fm0(sample(Σ)).
ABB (Marcus 1999 et al.)	gGtTnNlL	4h	8	10000 (64)	-41814.8	1	1	F0(x):=λx.append(append(sample(Σ), x), x).<br>F1(x):=λx.Fm0(sample(Σ)).
ABB (Marcus 1999 et al.)	gGtTnNlL	4h	8	100000 (64)	-417922	1	1	F0(x):=λx.append(append(sample(Σ), x), x).<br>F1(x):=λx.Fm0(sample(Σ)).
ABB (Marcus 1999 et al.)	gGtTnNlL	4h	8	1 (1)	-31.7116	1	0	F0(x):=λx.pair(ε, L).<br>F1(x):=λx.pair(pair(Fm0(ε), N), N).<br>F2(x):=λx.Fm1(ε).
ABB (Marcus 1999 et al.)	gGtTnNlL	4h	8	10 (8)	-74.2683	1	1	F0(x):=λx.append(x, x).<br>F1(x):=λx.sample(Σ).<br>F2(x):=λx.append(F1(ε), F0(sample(Σ))).
ABB (Marcus 1999 et al.)	gGtTnNlL	4h	8	100 (54)	-450.376	1	1	F0(x):=λx.Fm1(sample(Σ)).<br>F1(x):=λx.append(x, x).<br>F2(x):=λx.append(sample(Σ), Fm0(ε)).
ABB (Marcus 1999 et al.)	gGtTnNlL	4h	8	1000 (64)	-4211.45	1	1	F0(x):=λx.Fm1(sample(Σ)).<br>F1(x):=λx.append(x, x).<br>F2(x):=λx.append(sample(Σ), Fm0(ε)).
ABB (Marcus 1999 et al.)	gGtTnNlL	4h	8	10000 (64)	-41822.2	1	1	F0(x):=λx.Fm1(sample(Σ)).<br>F1(x):=λx.append(x, x).<br>F2(x):=λx.append(sample(Σ), Fm0(ε)).
ABB (Marcus 1999 et al.)	gGtTnNlL	4h	8	100000 (64)	-417930	1	1	F0(x):=λx.append(F1(x), x).<br>F1(x):=λx.x.<br>F2(x):=λx.append(sample(Σ), Fm0(sample(Σ))).
ABB (Marcus 1999 et al.)	gGtTnNlL	4h	8	1 (1)	-39.6047	1	0	F0(x):=λx.ε.<br>F1(x):=λx.F0(ε).<br>F2(x):=λx.pair(pair(ε, L), N).<br>F3(x):=λx.pair(Fm2(F1(ε)), N).
ABB (Marcus 1999 et al.)	gGtTnNlL	4h	8	10 (8)	-82.1615	1	1	F0(x):=λx.append(append(F2(ε), x), x).<br>F1(x):=λx.Fm0(sample(Σ)).<br>F2(x):=λx.sample(Σ).<br>F3(x):=λx.Fm1(ε).
ABB (Marcus 1999 et al.)	gGtTnNlL	4h	8	100 (54)	-458.269	1	1	F0(x):=λx.append(append(F2(ε), x), x).<br>F1(x):=λx.Fm0(sample(Σ)).<br>F2(x):=λx.sample(Σ).<br>F3(x):=λx.Fm1(ε).
ABB (Marcus 1999 et al.)	gGtTnNlL	4h	8	1000 (64)	-4219.34	1	1	F0(x):=λx.append(sample(Σ), append(x, x)).<br>F1(x):=λx.Fm0(sample(Σ)).<br>F2(x):=λx.F1(ε).<br>F3(x):=λx.F2(ε).
ABB (Marcus 1999 et al.)	gGtTnNlL	4h	8	10000 (64)	-41830.1	1	1	F0(x):=λx.append(sample(Σ), append(x, x)).<br>F1(x):=λx.Fm0(sample(Σ)).<br>F2(x):=λx.F1(ε).<br>F3(x):=λx.F2(ε).

ABB (Marcus 1999 et al.)	gGtTnNlL	4h	8	100000 (64)	-417937	1	1	$F0(x)$:=λx.append(sample(Σ), append(x, x)).<br>$F1(x)$:=λx.Fm0(sample(Σ)).<br>$F2(x)$:=λx.F1(ϵ).<br>$F3(x)$:=λx.F2(ϵ).
Modified from Hudson Kam (2009)	!vVnd	2d	8	1 (1)	-17.2508	1	0.08333	$F0(x)$:=λx.pair(pair(pair(ϵ, !), v), n).
Modified from Hudson Kam (2009)	!vVnd	2d	8	10 (6)	-96.5934	1	0.33333	$F0(x)$:=λx.append(if(flip(1/2), ϵ, pair(ϵ, !)), pair(if(flip(1/2), pair(pair(pair(ϵ, V), n), d), pair(ϵ, v)), n)).
Modified from Hudson Kam (2009)	!vVnd	2d	8	100 (12)	-310.669	1	1	$F0(x)$:=λx.append(pair(if(and(flip(1/2), empty(x)), Fm0(pair(ϵ, V)), append(if(flip(1/2), pair(ϵ, !), ϵ), head(pair(x, v)))), n), if(flip(3/8), ϵ, pair(ϵ, d))).
Modified from Hudson Kam (2009)	!vVnd	2d	8	1000 (12)	-2459.78	1	1	$F0(x)$:=λx.append(pair(if(and(empty(x), flip(1/2)), Fm0(pair(ϵ, V)), append(if(flip(1/2), pair(ϵ, !), ϵ), head(pair(x, v)))), n), if(flip(5/12), ϵ, pair(ϵ, d))).
Modified from Hudson Kam (2009)	!vVnd	2d	8	10000 (12)	-24225.8	1	1	$F0(x)$:=λx.append(pair(if(and(empty(x), flip(1/2)), Fm0(pair(ϵ, V)), append(if(flip(1/2), pair(ϵ, !), ϵ), head(pair(x, v)))), n), if(flip(5/12), ϵ, pair(ϵ, d))).
Modified from Hudson Kam (2009)	!vVnd	2d	8	100000 (12)	-241167	1	1	$F0(x)$:=λx.append(if(or(not((x==ϵ)), flip(1/2)), ϵ, pair(ϵ, !)), append(pair(if(and((ϵ==x), flip(1/2)), F0(pair(ϵ, V)), head(pair(x, v))), n), if(or(flip(5/24), flip(1/2)), pair(ϵ, d), ϵ))).
Modified from Hudson Kam (2009)	!vVnd	2d	8	1 (1)	-23.8754	1	0.08333	$F0(x)$:=λx.pair(pair(pair(ϵ, !), v), n).<br>$F1(x)$:=λx.Fm0(ϵ).
Modified from Hudson Kam (2009)	!vVnd	2d	8	10 (6)	-99.7256	0.16666	1	$F0(x)$:=λx.append(if(flip(1/2), pair(ϵ, !), ϵ), if(flip(1/2), x, pair(x, d))).<br>$F1(x)$:=λx.F0(pair(sample(if(flip(1/2), Σ, F1(ϵ))), n)).
Modified from Hudson Kam (2009)	!vVnd	2d	8	100 (12)	-310.123	1	1	$F0(x)$:=λx.append(pair(x, n), if(flip(1/2), pair(ϵ, d), ϵ)).<br>$F1(x)$:=λx.F0(append(if(flip(1/2), pair(ϵ, !), ϵ), if(flip(1/2), pair(ϵ, v), Fm0(pair(ϵ, V))))).
Modified from Hudson Kam (2009)	!vVnd	2d	8	1000 (12)	-2459.84	1	1	$F0(x)$:=λx.append(pair(x, n), if(not(flip(5/12)), pair(ϵ, d), ϵ)).<br>$F1(x)$:=λx.F0(append(if(flip(1/2), pair(ϵ, !), ϵ), if(flip(1/2), pair(ϵ, v), Fm0(pair(ϵ, V))))).
Modified from Hudson Kam (2009)	!vVnd	2d	8	10000 (12)	-24220.3	1	1	$F0(x)$:=λx.if(or(flip(1/2), flip(5/24)), pair(pair(x, n), d), pair(x, n)).<br>$F1(x)$:=λx.append(if(flip(1/2), ϵ, pair(ϵ, !)), append(if(flip(1/2), Fm0(pair(ϵ, V)), pair(ϵ, v)), Fm0(ϵ))).
Modified from Hudson Kam (2009)	!vVnd	2d	8	100000 (12)	-241159	1	1	$F0(x)$:=λx.if(or(flip(5/24), flip(1/2)), pair(pair(x, n), d), pair(x, n)).<br>$F1(x)$:=λx.append(if(flip(1/2), ϵ, pair(ϵ, !)), append(if(flip(1/2), Fm0(pair(ϵ, V)), pair(ϵ, v)), F0(ϵ))).
Modified from Hudson Kam (2009)	!vVnd	2d	8	1 (1)	-31.311	1	0.08333	$F0(x)$:=λx.pair(pair(ϵ, !), v).<br>$F1(x)$:=λx.F0(ϵ).<br>$F2(x)$:=λx.pair(Fm1(ϵ), n).
Modified from Hudson Kam (2009)	!vVnd	2d	8	10 (6)	-98.8902	0.42857	1	$F0(x)$:=λx.pair(if(flip(1/2), x, F1(pair(ϵ, V))), n).<br>$F1(x)$:=λx.append(Fm0(x), if(flip(1/2), ϵ, pair(ϵ, d))).<br>$F2(x)$:=λx.append(if(flip(1/2), pair(ϵ, !), ϵ), Fm1(pair(ϵ, v))).
Modified from Hudson Kam (2009)	!vVnd	2d	8	100 (12)	-318.657	1	1	$F0(x)$:=λx.pair(x, n).<br>$F1(x)$:=λx.append(Fm0(x), if(flip(1/2), pair(ϵ, d), ϵ)).<br>$F2(x)$:=λx.F1(append(if(flip(1/2), pair(ϵ, !), ϵ), if(flip(1/2), pair(ϵ, v), Fm1(pair(ϵ, V))))).
Modified from Hudson Kam (2009)	!vVnd	2d	8	1000 (12)	-2467.96	1	1	$F0(x)$:=λx.if(flip(1/2), ϵ, pair(ϵ, !)).<br>$F1(x)$:=λx.if(flip(5/12), pair(x, n), pair(pair(x, n), d)).<br>$F2(x)$:=λx.Fm1(if(flip(11/24), F1(pair(F0(ϵ), V)), pair(F0(ϵ), v))).
Modified from Hudson Kam (2009)	!vVnd	2d	8	10000 (12)	-24228.1	1	1	$F0(x)$:=λx.F1(pair(pair(ϵ, V), n)).<br>$F1(x)$:=λx.if(flip(if(flip(1/2), 1/2, 7/24)), x, pair(x, d)).<br>$F2(x)$:=λx.append(sample(($\epsilon \cup$pair(ϵ, !))), F1(pair(if(flip(1/2), pair(ϵ, v), F0(ϵ)), n))).
Modified from Hudson Kam (2009)	!vVnd	2d	8	100000 (12)	-241167	1	1	$F0(x)$:=λx.F1(pair(pair(ϵ, V), n)).<br>$F1(x)$:=λx.if(flip(if(flip(1/2), 1/2, 7/24)), x, pair(x, d)).<br>$F2(x)$:=λx.append(sample(($\epsilon \cup$pair(x, !))), F1(pair(if(flip(1/2), pair(ϵ, v), F0(ϵ)), n))).
Modified from Hudson Kam (2009)	!vVnd	2d	8	1 (1)	-39.2041	1	0.08333	$F0(x)$:=λx.pair(pair(pair(ϵ, !), v), n).<br>$F1(x)$:=λx.Fm0(Fm2(ϵ)).<br>$F2(x)$:=λx.ϵ.<br>$F3(x)$:=λx.F1(ϵ).

Modified from Hudson Kam (2009)	!vVnd	2d	8	10 (6)	-117.194	1	0.5	$F0(x)$:=λx.if(flip(1/2), pair(F2(ϵ), !), ϵ).<br>$F1(x)$:=λx.append(pair(pair(x, V), n), if(flip(1/2), pair(ϵ, d), ϵ)).<br>$F2(x)$:=λx.ϵ.<br>$F3(x)$:=λx.insert(pair(Fm0(ϵ), n), if(flip(1/2), F1(ϵ), pair(ϵ, v))).
Modified from Hudson Kam (2009)	!vVnd	2d	8	100 (12)	-330.521	1	1	$F0(x)$:=λx.append(pair(x, n), F2(ϵ)).<br>$F1(x)$:=λx.ϵ.<br>$F2(x)$:=λx.if(flip(5/12), ϵ, pair(ϵ, d)).<br>$F3(x)$:=λx.append(sample((pair(Fm1(ϵ), !) $\cup$ ϵ)), append(if(flip(1/2), Fm0(pair(ϵ, V)), pair(ϵ, v)), F0(ϵ))).
Modified from Hudson Kam (2009)	!vVnd	2d	8	1000 (12)	-2473.4	1	1	$F0(x)$:=λx.append(pair(x, n), if(not(flip(5/12)), pair(ϵ, d), ϵ)).<br>$F1(x)$:=λx.if(flip(1/2), F0(pair(x, V)), pair(x, v)).<br>$F2(x)$:=λx.if(flip(1/2), pair(ϵ, !), ϵ).<br>$F3(x)$:=λx.F0(F1(Fm2(ϵ))).
Modified from Hudson Kam (2009)	!vVnd	2d	8	10000 (12)	-24228.1	1	1	$F0(x)$:=λx.append(pair(x, n), if(or(flip(5/24), flip(1/2)), pair(ϵ, d), ϵ)).<br>$F1(x)$:=λx.if(flip(1/2), F0(pair(x, V)), pair(x, v)).<br>$F2(x)$:=λx.if(flip(1/2), pair(ϵ, !), ϵ).<br>$F3(x)$:=λx.F0(F1(F2(ϵ))).
Modified from Hudson Kam (2009)	!vVnd	2d	8	100000 (12)	-241167	1	1	$F0(x)$:=λx.append(pair(x, n), if(or(flip(5/24), flip(1/2)), pair(ϵ, d), ϵ)).<br>$F1(x)$:=λx.if(flip(1/2), F0(pair(x, V)), pair(x, v)).<br>$F2(x)$:=λx.if(flip(1/2), pair(ϵ, !), ϵ).<br>$F3(x)$:=λx.F0(F1(F2(ϵ))).
Gomez (2002) 1b for $n = 2$	abcde12345	1d	8	1 (1)	-20.2464	1	0.25	$F0(x)$:=λx.pair(pair(pair(ϵ, b), 1), e).
Gomez (2002) 1b for $n = 2$	abcde12345	1d	8	10 (4)	-82.595	0.5	0.5	$F0(x)$:=λx.insert(pair(if(flip(1/2), pair(ϵ, a), pair(pair(ϵ, b), e)), d), if(flip(1/2), pair(ϵ, 1), pair(ϵ, 2))).
Gomez (2002) 1b for $n = 2$	abcde12345	1d	8	100 (4)	-212.605	1	1	$F0(x)$:=λx.if(flip(1/2), Fm0(pair(pair(ϵ, b), e)), insert(if(empty(x), pair(pair(ϵ, a), d), x), sample(if(flip(1/2), pair(ϵ, 1), pair(ϵ, 2))))).
Gomez (2002) 1b for $n = 2$	abcde12345	1d	8	1000 (4)	-1478.36	1	1	$F0(x)$:=λx.if(flip(1/2), Fm0(pair(pair(ϵ, b), e)), insert(if(empty(x), pair(pair(ϵ, a), d), x), sample(if(flip(1/2), pair(ϵ, 1), pair(ϵ, 2))))).
Gomez (2002) 1b for $n = 2$	abcde12345	1d	8	10000 (4)	-14135.9	1	1	$F0(x)$:=λx.if(flip(1/2), Fm0(pair(pair(ϵ, b), e)), insert(if(empty(x), pair(pair(ϵ, a), d), x), sample(if(flip(1/2), pair(ϵ, 1), pair(ϵ, 2))))).
Gomez (2002) 1b for $n = 2$	abcde12345	1d	8	100000 (4)	-140711	1	1	$F0(x)$:=λx.if(flip(1/2), Fm0(pair(pair(ϵ, b), e)), insert(if(empty(x), pair(pair(ϵ, a), d), x), sample(if(flip(1/2), pair(ϵ, 1), pair(ϵ, 2))))).
Gomez (2002) 1b for $n = 2$	abcde12345	1d	8	1 (1)	-26.871	1	0.25	$F0(x)$:=λx.ϵ.<br>$F1(x)$:=λx.pair(pair(pair(Fm0(ϵ), b), 1), e).
Gomez (2002) 1b for $n = 2$	abcde12345	1d	8	10 (4)	-83.3221	1	1	$F0(x)$:=λx.insert(if(flip(1/2), pair(pair(ϵ, a), d), append(pair(ϵ, b), pair(ϵ, e))), sample(if(flip(1/2), pair(ϵ, 1), pair(ϵ, 2)))).<br>$F1(x)$:=λx.F0(ϵ).
Gomez (2002) 1b for $n = 2$	abcde12345	1d	8	100 (4)	-206.045	1	1	$F0(x)$:=λx.pair(pair(ϵ, b), e).<br>$F1(x)$:=λx.insert(if(flip(1/2), F0(ϵ), pair(pair(ϵ, a), d)), sample(if(flip(1/2), pair(ϵ, 1), pair(ϵ, 2)))).
Gomez (2002) 1b for $n = 2$	abcde12345	1d	8	1000 (4)	-1471.8	1	1	$F0(x)$:=λx.pair(pair(ϵ, b), e).<br>$F1(x)$:=λx.insert(if(flip(1/2), F0(ϵ), pair(pair(ϵ, a), d)), sample(if(flip(1/2), pair(ϵ, 1), pair(ϵ, 2)))).
Gomez (2002) 1b for $n = 2$	abcde12345	1d	8	10000 (4)	-14129.3	1	1	$F0(x)$:=λx.pair(pair(ϵ, b), e).<br>$F1(x)$:=λx.insert(if(flip(1/2), F0(ϵ), pair(pair(ϵ, a), d)), sample(if(flip(1/2), pair(ϵ, 1), pair(ϵ, 2)))).
Gomez (2002) 1b for $n = 2$	abcde12345	1d	8	100000 (4)	-140704	1	1	$F0(x)$:=λx.pair(pair(ϵ, b), e).<br>$F1(x)$:=λx.insert(if(flip(1/2), F0(ϵ), pair(pair(ϵ, a), d)), sample(if(flip(1/2), pair(ϵ, 1), pair(ϵ, 2)))).
Gomez (2002) 1b for $n = 2$	abcde12345	1d	8	1 (1)	-34.3066	1	0.25	$F0(x)$:=λx.pair(ϵ, b).<br>$F1(x)$:=λx.pair(pair(F0(ϵ), 1), e).<br>$F2(x)$:=λx.Fm1(ϵ).
Gomez (2002) 1b for $n = 2$	abcde12345	1d	8	10 (4)	-96.1128	0.19047	1	$F0(x)$:=λx.pair(append(pair(ϵ, a), if(flip(1/2), pair(ϵ, 1), x)), d).<br>$F1(x)$:=λx.append(pair(ϵ, b), pair(sample(Σ), e)).<br>$F2(x)$:=λx.if(flip(1/2), Fm0(pair(ϵ, 2)), Fm1(ϵ)).
Gomez (2002) 1b for $n = 2$	abcde12345	1d	8	100 (4)	-215.447	1	1	$F0(x)$:=λx.if(flip(1/2), F1(x), pair(x, 2)).<br>$F1(x)$:=λx.pair(x, 1).<br>$F2(x)$:=λx.if(flip(1/2), append(Fm0(pair(ϵ, a)), pair(ϵ, d)), pair(F0(pair(ϵ, b)), e)).
Gomez (2002) 1b for $n = 2$	abcde12345	1d	8	1000 (4)	-1481.2	1	1	$F0(x)$:=λx.if(flip(1/2), F1(x), pair(x, 2)).<br>$F1(x)$:=λx.pair(x, 1).<br>$F2(x)$:=λx.if(flip(1/2), append(Fm0(pair(ϵ, a)), pair(ϵ, d)), pair(F0(pair(ϵ, b)), e)).
Gomez (2002) 1b for $n = 2$	abcde12345	1d	8	10000 (4)	-14138.7	1	1	$F0(x)$:=λx.if(flip(1/2), F1(x), pair(x, 2)).<br>$F1(x)$:=λx.pair(x, 1).<br>$F2(x)$:=λx.if(flip(1/2), append(Fm0(pair(ϵ, a)), pair(ϵ, d)), pair(F0(pair(ϵ, b)), e)).
Gomez (2002) 1b for $n = 2$	abcde12345	1d	8	100000 (4)	-140714	1	1	$F0(x)$:=λx.if(flip(1/2), F1(x), pair(x, 1)).<br>$F1(x)$:=λx.pair(x, 2).<br>$F2(x)$:=λx.if(flip(1/2), append(Fm0(pair(x, a)), pair(ϵ, d)), pair(F0(pair(ϵ, b)), e)).

Gomez (2002) 1b for $n = 2$	abcde12345	1d	8	1 (1)	-42.1997	1	0.25	$F0(x)$:=λx.pair(pair(Fm2(ε), 1), e).<br>$F1(x)$:=λx.pair(ε, b).<br>$F2(x)$:=λx.F1(ε).<br>$F3(x)$:=λx.Fm0(ε).
Gomez (2002) 1b for $n = 2$	abcde12345	1d	8	10 (4)	-99.3044	0.10526	1	$F0(x)$:=λx.insert(if(flip(1/2), pair(pair(ε, b), e), F2(ε)), sample(Σ)).<br>$F1(x)$:=λx.ε.<br>$F2(x)$:=λx.pair(pair(Fm1(ε), a), d).<br>$F3(x)$:=λx.F0(ε).
Gomez (2002) 1b for $n = 2$	abcde12345	1d	8	100 (4)	-221.63	1	1	$F0(x)$:=λx.pair(append(pair(ε, b), x), e).<br>$F1(x)$:=λx.if(flip(1/2), pair(ε, 2), pair(ε, 1)).<br>$F2(x)$:=λx.if(flip(1/2), F0(x), pair(append(pair(ε, a), x), d)).<br>$F3(x)$:=λx.F2(Fm1(ε)).
Gomez (2002) 1b for $n = 2$	abcde12345	1d	8	1000 (4)	-1483.06	1	1	$F0(x)$:=λx.pair(pair(ε, b), e).<br>$F1(x)$:=λx.pair(ε, 2).<br>$F2(x)$:=λx.insert(if(flip(1/2), pair(pair(ε, a), d), Fm0(ε)), if(flip(1/2), Fm1(ε), pair(ε, 1))).<br>$F3(x)$:=λx.F2(ε).
Gomez (2002) 1b for $n = 2$	abcde12345	1d	8	10000 (4)	-14140.6	1	1	$F0(x)$:=λx.pair(pair(ε, b), e).<br>$F1(x)$:=λx.pair(ε, 2).<br>$F2(x)$:=λx.insert(if(flip(1/2), pair(pair(ε, a), d), Fm0(ε)), if(flip(1/2), Fm1(ε), pair(ε, 1))).<br>$F3(x)$:=λx.F2(ε).
Gomez (2002) 1b for $n = 2$	abcde12345	1d	8	100000 (4)	-140716	1	1	$F0(x)$:=λx.pair(pair(ε, b), e).<br>$F1(x)$:=λx.pair(ε, 2).<br>$F2(x)$:=λx.insert(if(flip(1/2), pair(pair(ε, a), d), Fm0(ε)), if(flip(1/2), Fm1(ε), pair(ε, 1))).<br>$F3(x)$:=λx.F2(x).
Gomez (2002) 1b for $n = 6$	abcde12345	5d	8	1 (1)	-20.2464	1	0.08333	$F0(x)$:=λx.pair(pair(pair(ε, b), 6), e).
Gomez (2002) 1b for $n = 6$	abcde12345	5d	8	10 (9)	-77.3511	0.31578	1	$F0(x)$:=λx.insert(if(flip(1/2), pair(pair(ε, b), e), pair(pair(ε, a), d)), sample(Σ)).
Gomez (2002) 1b for $n = 6$	abcde12345	5d	8	100 (12)	-394.091	0.31578	1	$F0(x)$:=λx.insert(if(flip(1/2), pair(pair(ε, a), d), pair(pair(ε, b), e)), sample((if(flip(1/2), if(flip(1/2), pair(ε, 5), pair(ε, 2)), Σ) ∪ pair(ε, 1)))).
Gomez (2002) 1b for $n = 6$	abcde12345	5d	8	1000 (12)	-3148.65	0.6	1	$F0(x)$:=λx.insert(if(flip(11/24), pair(pair(ε, b), e), pair(pair(ε, a), d)), sample(((((Σ\pair(ε, a))\(pair(ε, c) ∪ pair(ε, x)))\((pair(ε, 0) ∪ pair(ε, y)) ∪ pair(ε, w)))\(pair(ε, z) ∪ (pair(ε, e) ∪ pair(ε, 8)))))).
Gomez (2002) 1b for $n = 6$	abcde12345	5d	8	10000 (12)	-30291.5	0.6	1	$F0(x)$:=λx.insert(if(flip(1/2), pair(pair(ε, b), e), pair(pair(ε, a), d)), sample(((((Σ\pair(ε, y))\(pair(ε, b) ∪ pair(ε, a)))\((pair(ε, z) ∪ pair(ε, 7)) ∪ pair(ε, d)))\(pair(ε, e) ∪ (pair(ε, 8) ∪ pair(ε, 0)))))).
Gomez (2002) 1b for $n = 6$	abcde12345	5d	8	100000 (12)	-301716	0.6	1	$F0(x)$:=λx.insert(if(flip(1/2), pair(pair(ε, b), e), pair(pair(ε, a), d)), sample(((((Σ\pair(ε, y))\(pair(ε, b) ∪ pair(ε, a)))\((pair(ε, z) ∪ pair(ε, 7)) ∪ pair(ε, d)))\(pair(ε, e) ∪ (pair(ε, 8) ∪ pair(ε, 0)))))).
Gomez (2002) 1b for $n = 6$	abcde12345	5d	8	1 (1)	-26.871	1	0.08333	$F0(x)$:=λx.ε.<br>$F1(x)$:=λx.pair(pair(pair(Fm0(ε), b), 6), e).
Gomez (2002) 1b for $n = 6$	abcde12345	5d	8	10 (9)	-83.9757	0.31578	1	$F0(x)$:=λx.pair(ε, a).<br>$F1(x)$:=λx.insert(if(flip(1/2), pair(F0(ε), d), pair(pair(ε, b), e)), sample(Σ)).
Gomez (2002) 1b for $n = 6$	abcde12345	5d	8	100 (12)	-384.371	1	1	$F0(x)$:=λx.append(if(flip(5/24), pair(x, 1), if(flip(1/2), if(flip(5/24), pair(x, 4), if(flip(1/2), pair(x, 5), pair(x, 3))), if(flip(1/2), pair(x, 6), pair(x, 2)))), sample(if((x==pair(ε, a)), pair(ε, d), pair(ε, e)))).<br>$F1(x)$:=λx.Fm0(sample(if(flip(1/2), pair(ε, b), pair(ε, a)))).
Gomez (2002) 1b for $n = 6$	abcde12345	5d	8	1000 (12)	-2665.66	1	1	$F0(x)$:=λx.append(if(flip(1/6), pair(x, 1), if(flip(1/2), if(flip(1/3), pair(x, 4), if(flip(1/2), pair(x, 5), pair(x, 3))), if(flip(1/2), pair(x, 6), pair(x, 2)))), sample(if((x==pair(ε, a)), pair(ε, d), pair(ε, e)))).<br>$F1(x)$:=λx.Fm0(sample(if(flip(1/2), pair(ε, b), pair(ε, a)))).
Gomez (2002) 1b for $n = 6$	abcde12345	5d	8	10000 (12)	-25350.7	1	1	$F0(x)$:=λx.append(if(flip(1/6), pair(x, 1), if(flip(1/2), if(flip(1/3), pair(x, 4), if(flip(1/2), pair(x, 5), pair(x, 3))), if(flip(1/2), pair(x, 6), pair(x, 2)))), sample(if((x==pair(ε, a)), pair(ε, d), pair(ε, e)))).<br>$F1(x)$:=λx.Fm0(sample(if(flip(1/2), pair(ε, b), pair(ε, a)))).
Gomez (2002) 1b for $n = 6$	abcde12345	5d	8	100000 (12)	-250616	1	1	$F0(x)$:=λx.if(flip(1/2), append(pair(ε, b), x), pair(append(pair(ε, a), head(x)), d)).<br>$F1(x)$:=λx.F0(pair(if(flip(1/3), if(flip(1/2), pair(ε, 3), pair(ε, 1)), if(flip(1/2), if(flip(1/2), pair(ε, 6), pair(ε, 4)), if(flip(1/2), pair(ε, 5), pair(ε, 2)))), e)).
Gomez (2002) 1b for $n = 6$	abcde12345	5d	8	1 (1)	-34.3066	1	0.08333	$F0(x)$:=λx.ε.<br>$F1(x)$:=λx.pair(pair(ε, b), 6).<br>$F2(x)$:=λx.pair(Fm1(Fm0(ε)), e).
Gomez (2002) 1b for $n = 6$	abcde12345	5d	8	10 (9)	-91.4113	0.31578	1	$F0(x)$:=λx.ε.<br>$F1(x)$:=λx.pair(ε, b).<br>$F2(x)$:=λx.insert(if(flip(1/2), pair(Fm1(Fm0(ε)), e), pair(pair(ε, a), d)), sample(Σ)).
Gomez (2002) 1b for $n = 6$	abcde12345	5d	8	100 (12)	-420.603	0.31578	1	$F0(x)$:=λx.ε.<br>$F1(x)$:=λx.pair(ε, b).<br>$F2(x)$:=λx.insert(if(flip(1/2), pair(Fm1(Fm0(ε)), e), pair(pair(ε, a), d)), sample(Σ)).

Gomez (2002) 1b for $n = 6$	abcde12345	5d	8	1000 (12)	-2623.4	1	1	$F0(x)$:=λx.sample(if(flip(1/2), Fm1(ϵ), ((pair(ϵ, 5) ∪ pair(ϵ, 1)) ∪ pair(ϵ, 3)))).<br>$F1(x)$:=λx.if(flip(3/8), pair(ϵ, 4), if(flip(1/2), pair(ϵ, 2), pair(ϵ, 6))).<br>$F2(x)$:=λx.insert(if(flip(1/2), pair(pair(ϵ, a), d), pair(pair(ϵ, b), e)), F0(ϵ)).
Gomez (2002) 1b for $n = 6$	abcde12345	5d	8	10000 (12)	-25168.7	1	1	$F0(x)$:=λx.sample(if(flip(1/2), F1(ϵ), ((x ∪ pair(ϵ, 1)) ∪ pair(ϵ, 3)))).<br>$F1(x)$:=λx.if(flip(1/3), pair(ϵ, 4), if(flip(1/2), pair(ϵ, 2), pair(ϵ, 6))).<br>$F2(x)$:=λx.insert(if(flip(1/2), pair(pair(ϵ, a), d), pair(pair(ϵ, b), e)), Fm0(pair(ϵ, 5))).
Gomez (2002) 1b for $n = 6$	abcde12345	5d	8	100000 (12)	-250619	1	1	$F0(x)$:=λx.sample(if(flip(1/2), F1(ϵ), ((x ∪ pair(ϵ, 1)) ∪ pair(ϵ, 3)))).<br>$F1(x)$:=λx.if(flip(1/3), pair(ϵ, 4), if(flip(1/2), pair(ϵ, 2), pair(ϵ, 6))).<br>$F2(x)$:=λx.insert(if(flip(1/2), pair(pair(ϵ, a), d), pair(pair(ϵ, b), e)), Fm0(pair(ϵ, 5))).
Gomez (2002) 1b for $n = 6$	abcde12345	5d	8	1 (1)	-42.1997	1	0.08333	$F0(x)$:=λx.pair(ϵ, b).<br>$F1(x)$:=λx.pair(Fm0(ϵ), 6).<br>$F2(x)$:=λx.pair(Fm1(ϵ), e).<br>$F3(x)$:=λx.Fm2(ϵ).
Gomez (2002) 1b for $n = 6$	abcde12345	5d	8	10 (9)	-99.9976	0.31578	1	$F0(x)$:=λx.ϵ.<br>$F1(x)$:=λx.if(flip(1/2), pair(x, e), Fm2(ϵ)).<br>$F2(x)$:=λx.pair(pair(Fm0(ϵ), a), d).<br>$F3(x)$:=λx.insert(F1(pair(ϵ, b)), sample(Σ)).
Gomez (2002) 1b for $n = 6$	abcde12345	5d	8	100 (12)	-394.448	1	1	$F0(x)$:=λx.pair(append(pair(ϵ, b), Fm1(ϵ)), e).<br>$F1(x)$:=λx.Fm2(if(flip(1/2), pair(if(flip(5/12), pair(ϵ, 4), ϵ), 3), ϵ)).<br>$F2(x)$:=λx.head(append(if(flip(1/2), if(flip(7/24), pair(ϵ, 2), ϵ), pair(x, 1)), pair(if(flip(1/2), ϵ, pair(ϵ, 6)), 5))).<br>$F3(x)$:=λx.sample(if(flip(1/2), pair(append(pair(ϵ, a), F1(ϵ)), d), F0(ϵ))).
Gomez (2002) 1b for $n = 6$	abcde12345	5d	8	1000 (12)	-2665.64	1	1	$F0(x)$:=λx.pair(append(pair(ϵ, b), F1(ϵ)), e).<br>$F1(x)$:=λx.Fm2(if(not(flip(3/8)), pair(if(flip(11/24), pair(ϵ, 4), ϵ), 3), ϵ)).<br>$F2(x)$:=λx.head(append(if(flip(1/2), if((x==head(ϵ)), pair(ϵ, 2), ϵ), pair(x, 1)), pair(if(flip(1/2), ϵ, pair(ϵ, 6)), 5))).<br>$F3(x)$:=λx.sample(if(not(flip(1/2)), pair(append(pair(ϵ, a), Fm1(ϵ)), d), F0(x))).
Gomez (2002) 1b for $n = 6$	abcde12345	5d	8	10000 (12)	-25260.5	1	1	$F0(x)$:=λx.pair(append(pair(ϵ, b), F1(ϵ)), e).<br>$F1(x)$:=λx.Fm2(if(not(flip(3/8)), pair(if(flip(11/24), pair(ϵ, 4), ϵ), 3), ϵ)).<br>$F2(x)$:=λx.head(append(if(flip(1/2), if((x==head(ϵ)), pair(ϵ, 2), ϵ), pair(x, 1)), pair(if(flip(1/2), ϵ, pair(ϵ, 6)), 5))).<br>$F3(x)$:=λx.sample(if(not(flip(1/2)), pair(append(pair(ϵ, a), Fm1(ϵ)), d), F0(x))).
Gomez (2002) 1b for $n = 6$	abcde12345	5d	8	100000 (12)	-250637	1	1	$F0(x)$:=λx.ϵ.<br>$F1(x)$:=λx.append(sample(((pair(ϵ, 2) ∪ (pair(ϵ, 3) ∪ ((pair(ϵ, 5) ∪ F2(ϵ)) ∪ pair(ϵ, 6)))) ∪ pair(ϵ, 4))), pair(ϵ, d)).<br>$F2(x)$:=λx.pair(ϵ, 1).<br>$F3(x)$:=λx.if(flip(1/2), pair(insert(head(Fm1(Fm0(ϵ))), pair(ϵ, b)), e), append(pair(ϵ, a), Fm1(ϵ))).
Saffran	tprglbBdkF	7d	8	1 (1)	-18.8678	1	0.04	$F0(x)$:=λx.pair(pair(pair(ϵ, P), D), T).
Saffran	tprglbBdkF	7d	8	10 (8)	-177.696	0.96153	1	$F0(x)$:=λx.append(if(not(flip(5/12)), if(flip(1/4), pair(pair(pair(Fm0(ϵ), B), d), k), ϵ), F0(pair(pair(ϵ, g), l))), sample(if(flip(1/4), pair(pair(pair(ϵ, t), p), r), if(not(empty(x)), (pair(x, b) ∪ pair(pair(pair(ϵ, P), D), T)), ϵ)))).
Saffran	tprglbBdkF	7d	8	100 (53)	-1825.31	1	1	$F0(x)$:=λx.sample(if(flip(3/8), (pair(pair(pair(x, P), D), T) ∪ pair(pair(pair(x, g), l), b)), if(and(empty(x), not(flip(1/3))), F0(F0(ϵ)), if(not(flip(5/12)), append(pair(x, B), pair(pair(ϵ, d), k)), pair(pair(pair(x, t), p), r))))).
Saffran	tprglbBdkF	7d	8	1000 (346)	-19366.4	1	1	$F0(x)$:=λx.sample(if(flip(3/8), (pair(pair(pair(x, P), D), T) ∪ pair(pair(pair(x, g), l), b)), if(and(empty(x), not(flip(1/3))), F0(F0(ϵ)), if(not(flip(5/12)), append(pair(x, B), pair(pair(ϵ, d), k)), pair(pair(pair(x, t), p), r))))).
Saffran	tprglbBdkF	7d	8	10000 (2089)	-192748	1	1	$F0(x)$:=λx.append(sample(if(flip(7/24), pair(pair(pair(ϵ, g), l), b), if(flip(1/3), pair(insert(pair(ϵ, p), pair(ϵ, t)), r), (pair(pair(pair(ϵ, P), D), T) ∪ pair(pair(pair(ϵ, B), d), k))))), if(flip(11/24), Fm0(ϵ), ϵ)).
Saffran	tprglbBdkF	7d	8	100000 (12602)	-1.96139e+06	1	1	$F0(x)$:=λx.append(sample(if(flip(7/24), pair(pair(pair(ϵ, g), l), b), if(flip(1/3), pair(insert(pair(ϵ, p), pair(sample(ϵ), t)), r), (pair(pair(pair(x, P), D), T) ∪ pair(pair(pair(ϵ, B), d), k))))), if(flip(11/24), F0(ϵ), x)).
Saffran	tprglbBdkF	7d	8	1 (1)	-25.4924	1	0.04	$F0(x)$:=λx.pair(pair(ϵ, P), D).<br>$F1(x)$:=λx.pair(F0(ϵ), T).
Saffran	tprglbBdkF	7d	8	10 (8)	-199.219	0.96153	0.56	$F0(x)$:=λx.append(sample(if(flip(1/2), (x ∪ ϵ), pair(pair(pair(if(flip(1/2), ϵ, x), t), p), r))), sample(if(flip(1/2), ϵ, if(flip(1/2), if(flip(1/2), x, pair(pair(pair(F0(ϵ), g), l), b)), pair(pair(pair(ϵ, P), D), T))))).<br>$F1(x)$:=λx.F0(pair(pair(pair(F0(ϵ), B), d), k)).
Saffran	tprglbBdkF	7d	8	100 (53)	-1820.13	1	1	$F0(x)$:=λx.pair(pair(pair(ϵ, P), D), T).<br>$F1(x)$:=λx.append(if(not(flip(1/2)), ϵ, Fm1(ϵ)), sample(if(flip(1/2), if(flip(1/2), F0(ϵ), pair(pair(pair(ϵ, g), l), b)), (pair(pair(pair(ϵ, B), d), k) ∪ pair(pair(pair(ϵ, t), p), r))))).
Saffran	tprglbBdkF	7d	8	1000 (346)	-18877.4	1	1	$F0(x)$:=λx.sample(if(flip(1/3), pair(append(pair(x, t), pair(ϵ, p)), r), (pair(pair(pair(x, g), l), b) ∪ pair(pair(pair(x, P), D), T)))).<br>$F1(x)$:=λx.if(flip(1/4), pair(pair(if(flip(5/12), pair(F1(ϵ), B), pair(if(flip(if(flip(1/3), 3/8, 1/2)), ϵ, ϵ), B)), d), k), F0(if(not(flip(5/12)), ϵ, F1(ϵ)))).
Saffran	tprglbBdkF	7d	8	10000 (2089)	-193154	1	1	$F0(x)$:=λx.sample(if(flip(1/3), pair(append(pair(x, t), pair(ϵ, p)), r), (pair(pair(pair(x, g), l), b) ∪ pair(pair(pair(x, P), D), T)))).<br>$F1(x)$:=λx.if(flip(1/4), pair(pair(if(flip(5/12), pair(F1(ϵ), B), pair(if(flip(if(flip(1/3), 3/8, 1/2)), ϵ, ϵ), B)), d), k), F0(if(not(flip(5/12)), ϵ, F1(ϵ)))).
Saffran	tprglbBdkF	7d	8	100000 (12602)	-1.9677e+06	1	1	$F0(x)$:=λx.sample(if(flip(1/3), pair(append(pair(x, t), pair(ϵ, p)), r), (pair(pair(pair(x, g), l), b) ∪ pair(pair(pair(x, P), D), T)))).<br>$F1(x)$:=λx.if(flip(1/4), pair(pair(if(flip(5/12), pair(F1(ϵ), B), pair(if(flip(if(flip(1/3), 3/8, 1/2)), x, ϵ), B)), d), k), F0(if(not(flip(5/12)), ϵ, F1(ϵ)))).
Saffran	tprglbBdkF	7d	8	1 (1)	-32.928	1	0.04	$F0(x)$:=λx.pair(pair(pair(ϵ, P), D), T).<br>$F1(x)$:=λx.Fm0(ϵ).<br>$F2(x)$:=λx.F1(ϵ).

Saffran	tprglbBdkF	7d	8	10 (8)	-192.933	1	0.72	F0(x):=λx.pair(pair(pair(ϵ, t), p), r).<br>F1(x):=λx.pair(pair(ϵ, P), D), T).<br>F2(x):=λx.if(flip(1/6), pair(pair(pair(x, g), l), b), if(not(flip(5/24)), if(not(flip(1/12)), if(not(flip(1/3)), F0(ϵ), append(Fm2(Fm1(ϵ)), F2(ϵ))), Fm1(ϵ)), pair(pair(pair(ϵ, B), d), k))).
Saffran	tprglbBdkF	7d	8	100 (53)	-1853.93	1	1	F0(x):=λx.pair(pair(pair(ϵ, t), p), r).<br>F1(x):=λx.sample(if(flip(3/8), pair(pair(pair(ϵ, P), D), T), (pair(pair(pair(ϵ, B), d), k) ∪ pair(x, b)))).<br>F2(x):=λx.append(if(flip(1/4), Fm0(ϵ), F1(pair(pair(ϵ, g), l))), if(flip(1/3), Fm2(ϵ), ϵ)).
Saffran	tprglbBdkF	7d	8	1000 (346)	-18818.2	1	1	F0(x):=λx.append(sample(if(not(empty(x)), if(flip(11/24), F2(ϵ), ϵ), pair(F1(ϵ), k))), x).<br>F1(x):=λx.pair(if(not(flip(1/3)), pair(F2(ϵ), B), pair(if(flip(3/8), ϵ, ϵ), B)), d).<br>F2(x):=λx.F0(if(flip(7/24), pair(pair(pair(ϵ, g), l), b), if(flip(3/8), pair(pair(pair(ϵ, t), p), r), if(flip(5/12), ϵ, pair(pair(pair(ϵ, P), D), T))))).
Saffran	tprglbBdkF	7d	8	10000 (2089)	-192971	1	1	F0(x):=λx.append(sample(if(not((x==ϵ)), if(flip(11/24), F2(ϵ), ϵ), pair(F1(ϵ), k))), x).<br>F1(x):=λx.pair(if(not(flip(1/3)), pair(F2(ϵ), B), pair(if(flip(3/8), ϵ, ϵ), B)), d).<br>F2(x):=λx.F0(if(flip(7/24), pair(pair(pair(ϵ, g), l), b), if(flip(3/8), pair(pair(pair(ϵ, t), p), r), if(flip(5/12), ϵ, pair(pair(pair(ϵ, P), D), T))))).
Saffran	tprglbBdkF	7d	8	100000 (12602)	-1.96419e+06	1	1	F0(x):=λx.append(sample(if(not(empty(x)), if(flip(11/24), Fm2(ϵ), ϵ), pair(F1(ϵ), k))), x).<br>F1(x):=λx.pair(if(not(flip(1/3)), pair(F2(ϵ), B), pair(if(flip(3/8), ϵ, ϵ), B)), d).<br>F2(x):=λx.F0(if(flip(7/24), pair(pair(pair(head(ϵ), g), l), b), if(flip(3/8), pair(pair(pair(ϵ, t), p), r), if(flip(5/12), ϵ, pair(pair(pair(ϵ, P), D), T))))).
Saffran	tprglbBdkF	7d	8	1 (1)	-40.8211	1	0.04	F0(x):=λx.pair(pair(pair(Fm1(Fm2(ϵ)), P), D), T).<br>F1(x):=λx.ϵ.<br>F2(x):=λx.ϵ.<br>F3(x):=λx.F0(ϵ).
Saffran	tprglbBdkF	7d	8	10 (8)	-335.206	0.07692	0.16	F0(x):=λx.append(x, sample((pair(pair(pair(if(flip(1/2), ϵ, x), B), d), k) ∪ Σ))).<br>F1(x):=λx.if(flip(1/6), pair(pair(pair(Fm2(x), t), p), r), F0(x)).<br>F2(x):=λx.x.<br>F3(x):=λx.F1(Fm1(ϵ)).
Saffran	tprglbBdkF	7d	8	100 (53)	-1838.77	1	1	F0(x):=λx.pair(pair(pair(ϵ, P), D), T).<br>F1(x):=λx.append(if(flip(3/8), F3(ϵ), ϵ), x).<br>F2(x):=λx.pair(pair(ϵ, g), l).<br>F3(x):=λx.F1(if(flip(1/4), pair(pair(pair(ϵ, B), d), k), if(not(flip(1/4)), if(flip(5/12), pair(pair(pair(ϵ, t), p), r), F0(ϵ)), pair(F2(ϵ), b)))).
Saffran	tprglbBdkF	7d	8	1000 (346)	-18893.8	1	1	F0(x):=λx.if(not(flip(3/8)), ϵ, F3(ϵ)).<br>F1(x):=λx.pair(pair(F0(ϵ), t), p).<br>F2(x):=λx.sample(if(flip(7/24), insert(pair(ϵ, b), pair(pair(if(not(flip(5/12)), Fm3(ϵ), ϵ), g), l)), if(not(flip(5/12)), pair(Fm1(ϵ), r), pair(pair(pair(F0(ϵ), P), D), T)))).<br>F3(x):=λx.if(flip(7/24), pair(pair(pair(Fm0(ϵ), B), d), k), F2(ϵ)).
Saffran	tprglbBdkF	7d	8	10000 (2089)	-193110	1	1	F0(x):=λx.append(if(flip(5/24), pair(x, k), if(flip(3/8), F2(ϵ), Fm1(if(flip(11/24), pair(ϵ, p), ϵ)))), if(flip(11/24), Fm3(ϵ), ϵ)).<br>F1(x):=λx.sample(if(empty(x), pair(pair(pair(ϵ, g), l), b), append(pair(ϵ, t), insert(pair(ϵ, r), x)))).<br>F2(x):=λx.pair(pair(ϵ, P), D), T).<br>F3(x):=λx.F0(pair(pair(ϵ, B), d)).
Saffran	tprglbBdkF	7d	8	100000 (12602)	-1.96172e+06	1	1	F0(x):=λx.append(if(flip(5/24), pair(append(ϵ, x), k), if(flip(3/8), F2(pair(ϵ, P)), Fm1(if(flip(11/24), pair(ϵ, p), ϵ)))), if(flip(11/24), F3(head(ϵ)), ϵ)).<br>F1(x):=λx.sample(if((x==ϵ), pair(pair(pair(ϵ, g), l), b), append(pair(ϵ, t), insert(pair(ϵ, r), x)))).<br>F2(x):=λx.pair(pair(x, D), T).<br>F3(x):=λx.F0(pair(pair(ϵ, B), d)).
Milne (2018)	acdgf	1d	8	1 (1)	-25.6149	1	0.08333	F0(x):=λx.pair(pair(pair(pair(pair(ϵ, a), c), f), c), g).
Milne (2018)	acdgf	1d	8	10 (8)	-91.6462	1	0.66666	F0(x):=λx.append(append(pair(ϵ, a), pair(if(flip(1/2), ϵ, pair(ϵ, d)), c)), append(pair(if(flip(1/2), pair(ϵ, g), ϵ), f), if(flip(1/2), pair(ϵ, c), ϵ))).
Milne (2018)	acdgf	1d	8	100 (12)	-320.379	1	1	F0(x):=λx.append(append(pair(ϵ, a), if(flip(1/2), ϵ, pair(ϵ, d))), append(pair(if(flip(1/2), pair(ϵ, c), pair(pair(ϵ, c), g)), f), if(flip(1/2), if(flip(1/2), pair(ϵ, c), pair(pair(ϵ, c), g)), ϵ))).
Milne (2018)	acdgf	1d	8	1000 (12)	-2529.94	1	1	F0(x):=λx.append(append(pair(ϵ, a), if(flip(1/2), ϵ, pair(ϵ, d))), append(pair(if(flip(1/2), pair(ϵ, c), pair(pair(ϵ, c), g)), f), if(flip(1/2), if(flip(1/2), pair(ϵ, c), pair(pair(ϵ, c), g)), ϵ))).
Milne (2018)	acdgf	1d	8	10000 (12)	-24382.9	1	1	F0(x):=λx.append(pair(append(pair(append(pair(ϵ, a), if(flip(1/2), ϵ, pair(ϵ, d))), c), if(flip(1/2), pair(ϵ, g), ϵ)), f), sample(if(flip(1/2), append(pair(ϵ, c), if(flip(1/2), ϵ, pair(ϵ, g))), ϵ))).
Milne (2018)	acdgf	1d	8	100000 (12)	-244347	1	1	F0(x):=λx.append(pair(append(pair(append(pair(ϵ, a), if(flip(1/2), x, pair(ϵ, d))), c), if(flip(1/2), pair(ϵ, g), ϵ)), f), sample(if(flip(1/2), append(pair(ϵ, c), if(flip(1/2), ϵ, pair(ϵ, g))), ϵ))).
Milne (2018)	acdgf	1d	8	1 (1)	-32.2395	1	0.08333	F0(x):=λx.pair(pair(ϵ, a), c).<br>F1(x):=λx.pair(pair(pair(F0(ϵ), f), c), g).
Milne (2018)	acdgf	1d	8	10 (8)	-94.7924	0.66666	1	F0(x):=λx.if(flip(1/4), x, insert(if(flip(1/2), pair(ϵ, g), ϵ), pair(x, c))).<br>F1(x):=λx.F0(pair(Fm0(append(pair(ϵ, a), if(flip(1/2), ϵ, pair(ϵ, d)))), f)).
Milne (2018)	acdgf	1d	8	100 (12)	-316.329	1	1	F0(x):=λx.append(pair(ϵ, c), append(if(flip(1/2), pair(ϵ, g), ϵ), x)).<br>F1(x):=λx.append(pair(ϵ, a), append(append(if(flip(1/2), pair(ϵ, d), ϵ), Fm0(pair(ϵ, f))), if(flip(1/2), Fm0(ϵ), ϵ))).
Milne (2018)	acdgf	1d	8	1000 (12)	-2525.89	1	1	F0(x):=λx.append(pair(ϵ, c), append(if(flip(1/2), pair(ϵ, g), ϵ), x)).<br>F1(x):=λx.append(pair(ϵ, a), append(append(if(flip(1/2), pair(ϵ, d), ϵ), Fm0(pair(ϵ, f))), if(flip(1/2), Fm0(ϵ), ϵ))).
Milne (2018)	acdgf	1d	8	10000 (12)	-24380.3	1	1	F0(x):=λx.append(pair(ϵ, c), append(if(flip(1/2), pair(ϵ, g), ϵ), x)).<br>F1(x):=λx.append(pair(ϵ, a), append(append(if(flip(1/2), pair(ϵ, d), ϵ), Fm0(pair(ϵ, f))), if(flip(1/2), Fm0(ϵ), ϵ))).

Milne (2018)	acdgf	1d	8	100000 (12)	-244344	1	1	F0(x):=λx.append(pair(ε, c), append(if(flip(1/2), pair(ε, g), ε), x)).<br>F1(x):=λx.append(pair(ε, a), append(append(if(flip(1/2), pair(ε, d), ε), Fm0(pair(ε, f))), if(flip(1/2), Fm0(ε), ε))).
Milne (2018)	acdgf	1d	8	1 (1)	-39.675	1	0.08333	F0(x):=λx.pair(pair(ε, a), c).<br>F1(x):=λx.pair(F0(ε), f).<br>F2(x):=λx.pair(pair(F1(ε), c), g).
Milne (2018)	acdgf	1d	8	10 (8)	-102.46	0.6	1	F0(x):=λx.append(if(flip(1/2), x, pair(x, d)), append(pair(ε, c), if(flip(1/2), ε, pair(ε, g)))).<br>F1(x):=λx.ε.<br>F2(x):=λx.append(pair(F0(pair(ε, a)), f), if(flip(1/2), Fm0(ε), F1(ε))).
Milne (2018)	acdgf	1d	8	100 (12)	-320.884	1	1	F0(x):=λx.append(F1(x), if(flip(1/2), ε, pair(ε, g))).<br>F1(x):=λx.pair(if(or(flip(1/2), empty(x)), x, pair(x, d)), c).<br>F2(x):=λx.append(pair(F0(pair(ε, a)), f), if(flip(1/2), Fm0(ε), ε)).
Milne (2018)	acdgf	1d	8	1000 (12)	-2530.45	1	1	F0(x):=λx.append(F1(x), if(flip(1/2), ε, pair(ε, g))).<br>F1(x):=λx.pair(if(or(flip(1/2), empty(x)), x, pair(x, d)), c).<br>F2(x):=λx.append(pair(F0(pair(ε, a)), f), if(flip(1/2), Fm0(ε), ε)).
Milne (2018)	acdgf	1d	8	10000 (12)	-24384.8	1	1	F0(x):=λx.append(F1(x), if(flip(1/2), ε, pair(ε, g))).<br>F1(x):=λx.pair(if(or(flip(1/2), empty(x)), x, pair(x, d)), c).<br>F2(x):=λx.append(pair(F0(pair(ε, a)), f), if(flip(1/2), Fm0(ε), ε)).
Milne (2018)	acdgf	1d	8	100000 (12)	-244349	1	1	F0(x):=λx.append(Fm1(x), if(flip(1/2), ε, pair(ε, g))).<br>F1(x):=λx.pair(if(or(flip(1/2), (x==ε)), x, pair(x, d)), c).<br>F2(x):=λx.append(pair(F0(pair(ε, a)), f), if(flip(1/2), F0(ε), ε)).
Milne (2018)	acdgf	1d	8	1 (1)	-47.5682	1	0.08333	F0(x):=λx.ε.<br>F1(x):=λx.pair(pair(pair(Fm0(ε), a), c), f).<br>F2(x):=λx.pair(pair(Fm1(ε), c), g).<br>F3(x):=λx.Fm2(ε).
Milne (2018)	acdgf	1d	8	10 (8)	-108.668	1	0.66666	F0(x):=λx.if(flip(1/2), pair(x, d), x).<br>F1(x):=λx.if(flip(1/2), pair(x, c), x).<br>F2(x):=λx.pair(Fm0(pair(ε, a)), c).<br>F3(x):=λx.append(Fm2(ε), F1(pair(if(flip(1/2), pair(ε, g), ε), f))).
Milne (2018)	acdgf	1d	8	100 (12)	-331.331	1	1	F0(x):=λx.append(append(pair(ε, a), Fm2(ε)), x).<br>F1(x):=λx.if(flip(1/2), pair(x, g), x).<br>F2(x):=λx.Fm1(pair(if(flip(1/2), x, head(pair(x, d))), c)).<br>F3(x):=λx.Fm0(if(flip(1/2), pair(ε, f), F2(pair(ε, f)))).
Milne (2018)	acdgf	1d	8	1000 (12)	-2540.89	1	1	F0(x):=λx.append(append(pair(ε, a), Fm2(ε)), x).<br>F1(x):=λx.if(flip(1/2), pair(x, g), x).<br>F2(x):=λx.Fm1(pair(if(flip(1/2), x, head(pair(x, d))), c)).<br>F3(x):=λx.Fm0(if(flip(1/2), pair(ε, f), F2(pair(ε, f)))).
Milne (2018)	acdgf	1d	8	10000 (12)	-24395.3	1	1	F0(x):=λx.append(append(pair(ε, a), Fm2(ε)), x).<br>F1(x):=λx.if(flip(1/2), pair(x, g), x).<br>F2(x):=λx.Fm1(pair(if(flip(1/2), x, head(pair(x, d))), c)).<br>F3(x):=λx.Fm0(if(flip(1/2), pair(ε, f), F2(pair(ε, f)))).
Milne (2018)	acdgf	1d	8	100000 (12)	-244355	1	1	F0(x):=λx.if(flip(1/2), x, pair(x, g)).<br>F1(x):=λx.ε.<br>F2(x):=λx.append(append(pair(F1(ε), a), if(flip(1/2), ε, pair(ε, d))), append(pair(Fm0(x), f), if(flip(1/2), F0(x), ε))).<br>F3(x):=λx.Fm2(pair(x, c)).
Simple Elman (1990)	badigu	5d	8	1 (1)	-31.3955	1	0.04	F0(x):=λx.pair(pair(pair(pair(pair(pair(ε, d), i), i), b), a), a).
Simple Elman (1990)	badigu	5d	8	10 (9)	-139.157	0.84615	0.72	F0(x):=λx.if(flip(1/2), sample((x∪(pair(pair(pair(ε, d), i), i)∪pair(append(pair(ε, g), pair(pair(ε, u), u)), u)))), append(Fm0(append(pair(ε, b), pair(pair(x, a), a))), Fm0(ε))).
Simple Elman (1990)	badigu	5d	8	100 (52)	-1517.56	1	1	F0(x):=λx.if(flip(1/4), pair(pair(pair(x, b), a), a), sample(if(and(empty(x), not(flip(3/8))), Fm0(Fm0(ε)), (pair(pair(pair(pair(x, g), u), u), u)∪pair(pair(pair(x, d), i), i))))).
Simple Elman (1990)	badigu	5d	8	1000 (277)	-13893.6	1	1	F0(x):=λx.if(and(empty(x), flip(7/24)), Fm0(Fm0(ε)), sample(if(and(empty(x), not(flip(1/2))), pair(pair(pair(pair(if(flip(5/12), ε, Fm0(ε)), g), u), u), u), if(not(flip(11/24)), pair(pair(pair(x, b), a), a), pair(pair(pair(x, d), i), i))))).
Simple Elman (1990)	badigu	5d	8	10000 (1526)	-147689	1	1	F0(x):=λx.sample(if(flip(1/3), pair(pair(pair(if(not(flip(11/24)), ε, F0(ε)), d), i), i), if(flip(1/2), pair(pair(pair(if(flip(5/12), Fm0(ε), ε), b), a), a), pair(pair(append(pair(if(not(flip(5/12)), F0(ε), x), g), pair(ε, u)), u), u)))).
Simple Elman (1990)	badigu	5d	8	100000 (8441)	-1.50502e+06	1	1	F0(x):=λx.sample(if(flip(1/3), pair(pair(pair(if(not(flip(11/24)), ε, Fm0(x)), d), i), i), if(flip(1/2), pair(pair(pair(if(flip(5/12), F0(x), ε), b), a), a), pair(pair(append(pair(if(not(flip(5/12)), F0(x), ε), g), pair(x, u)), u), u)))).
Simple Elman (1990)	badigu	5d	8	1 (1)	-38.0202	1	0.04	F0(x):=λx.pair(pair(pair(pair(pair(ε, d), i), i), b), a).<br>F1(x):=λx.pair(Fm0(ε), a).
Simple Elman (1990)	badigu	5d	8	10 (9)	-127.184	1	1	F0(x):=λx.pair(pair(pair(ε, d), i), i).<br>F1(x):=λx.append(if(flip(1/2), ε, F1(ε)), if(flip(1/2), append(pair(ε, g), pair(pair(pair(ε, u), u), u)), if(flip(1/2), pair(pair(pair(ε, b), a), a), Fm0(ε)))).

Simple Elman (1990)	badigu	5d	8	100 (52)	-1521.7	1	1	F0(x):=λx.sample(if(not(flip(3/8)), if(flip(11/24), pair(pair(pair(pair(x, g), u), u), u), pair(append(pair(x, d), pair(ϵ, i)), i)), pair(append(x, pair(pair(ϵ, b), a)), a))).<br>F1(x):=λx.F0(if(flip(1/2), ϵ, F1(ϵ))).
Simple Elman (1990)	badigu	5d	8	1000 (277)	-14142.1	1	1	F0(x):=λx.if(not(flip(5/12)), append(pair(ϵ, g), pair(pair(pair(ϵ, u), u), u)), pair(pair(pair(ϵ, b), a), a)).<br>F1(x):=λx.append(sample(if(flip(1/2), ϵ, Fm1(ϵ))), sample(if(not(flip(1/3)), F0(ϵ), pair(pair(pair(ϵ, d), i), i)))).
Simple Elman (1990)	badigu	5d	8	10000 (1526)	-148048	1	1	F0(x):=λx.if(not(flip(5/12)), append(pair(ϵ, g), pair(pair(x, u), u)), pair(pair(pair(ϵ, b), a), a)).<br>F1(x):=λx.append(sample(if(flip(1/2), ϵ, Fm1(ϵ))), sample(if(not(flip(1/3)), F0(pair(ϵ, u)), pair(pair(pair(ϵ, d), i), i)))).
Simple Elman (1990)	badigu	5d	8	100000 (8441)	-1.50239e+06	1	1	F0(x):=λx.append(sample(if(flip(1/2), ϵ, Fm1(x))), x).<br>F1(x):=λx.F0(sample(if(flip(3/8), append(pair(pair(ϵ, g), u), pair(pair(ϵ, u), u)), (pair(pair(pair(ϵ, b), a), a) ∪ pair(pair(pair(ϵ, d), i), i))))).
Simple Elman (1990)	badigu	5d	8	1 (1)	-45.4557	1	0.04	F0(x):=λx.pair(pair(pair(pair(ϵ, d), i), i), b).<br>F1(x):=λx.pair(pair(F0(ϵ), a), a).<br>F2(x):=λx.Fm1(ϵ).
Simple Elman (1990)	badigu	5d	8	10 (9)	-153.205	0.96153	0.68	F0(x):=λx.if(and(empty(x), flip(1/2)), pair(pair(pair(Fm1(ϵ), d), i), i), x).<br>F1(x):=λx.Fm0(if(flip(1/2), ϵ, pair(pair(pair(pair(F2(ϵ), g), u), u), u))).<br>F2(x):=λx.if(not(flip(1/6)), Fm1(ϵ), pair(pair(pair(F1(ϵ), b), a), a)).
Simple Elman (1990)	badigu	5d	8	100 (52)	-1596.9	1	1	F0(x):=λx.pair(x, a).<br>F1(x):=λx.if(flip(11/24), F2(ϵ), ϵ).<br>F2(x):=λx.append(F1(ϵ), if(flip(1/3), pair(Fm0(pair(ϵ, b)), a), if(flip(1/2), pair(pair(pair(pair(ϵ, g), u), u), u), pair(pair(pair(ϵ, d), i), i)))).
Simple Elman (1990)	badigu	5d	8	1000 (277)	-14070.6	1	1	F0(x):=λx.ϵ.<br>F1(x):=λx.pair(pair(pair(if(flip(5/24), ϵ, ϵ), d), i), i).<br>F2(x):=λx.append(if(not(flip(1/2)), F2(ϵ), ϵ), sample(((pair(pair(pair(pair(ϵ, g), u), u), u) ∪ Fm1(ϵ)) ∪ append(pair(F0(ϵ), b), pair(pair(ϵ, a), a))))).
Simple Elman (1990)	badigu	5d	8	10000 (1526)	-147195	1	1	F0(x):=λx.if(not(flip(1/2)), Fm1(ϵ), pair(pair(pair(ϵ, d), i), i)).<br>F1(x):=λx.if((x==ϵ), pair(pair(pair(ϵ, b), a), a), append(x, if(not(flip(1/2)), Fm2(ϵ), ϵ))).<br>F2(x):=λx.F1(if(not(flip(3/8)), F0(ϵ), insert(pair(ϵ, u), pair(pair(pair(ϵ, g), u), u)))).
Simple Elman (1990)	badigu	5d	8	100000 (8441)	-1.50051e+06	1	1	F0(x):=λx.if(not(flip(1/2)), Fm1(ϵ), pair(pair(pair(x, d), i), i)).<br>F1(x):=λx.if(empty(x), pair(pair(pair(head(ϵ), b), a), a), append(x, if(not(flip(1/2)), F2(ϵ), ϵ))).<br>F2(x):=λx.F1(if(not(flip(3/8)), F0(x), insert(pair(head(ϵ), u), pair(pair(pair(ϵ, g), u), u)))).
Simple Elman (1990)	badigu	5d	8	1 (1)	-53.3489	1	0.04	F0(x):=λx.pair(pair(pair(Fm1(ϵ), i), i), b).<br>F1(x):=λx.pair(ϵ, d).<br>F2(x):=λx.F0(ϵ).<br>F3(x):=λx.pair(pair(Fm2(ϵ), a), a).
Simple Elman (1990)	badigu	5d	8	10 (9)	-148.168	1	0.88	F0(x):=λx.if(not(flip(1/6)), append(x, if(flip(1/2), ϵ, Fm2(ϵ))), pair(pair(pair(ϵ, b), a), a)).<br>F1(x):=λx.if(flip(1/2), pair(pair(pair(ϵ, d), i), i), x).<br>F2(x):=λx.F0(F1(pair(pair(pair(pair(ϵ, g), u), u), u))).<br>F3(x):=λx.F0(F2(ϵ)).
Simple Elman (1990)	badigu	5d	8	100 (52)	-1522.8	1	1	F0(x):=λx.sample(if(not(flip(1/2)), ϵ, F3(ϵ))).<br>F1(x):=λx.pair(pair(sample(if(flip(3/8), F3(ϵ), ϵ)), b), a).<br>F2(x):=λx.pair(pair(pair(ϵ, u), u), u).<br>F3(x):=λx.if(flip(1/4), insert(pair(ϵ, i), pair(pair(F0(ϵ), d), i)), if(flip(11/24), append(pair(F0(ϵ), g), Fm2(ϵ)), pair(Fm1(ϵ), a))).
Simple Elman (1990)	badigu	5d	8	1000 (277)	-14235	1	1	F0(x):=λx.append(append(F1(ϵ), sample(if(not((x==pair(ϵ, a))), if((x==pair(ϵ, u)), append(pair(ϵ, g), x), pair(ϵ, d)), pair(ϵ, b)))), append(x, x)).<br>F1(x):=λx.if(flip(1/2), ϵ, Fm3(ϵ)).<br>F2(x):=λx.Fm0(if(flip(7/24), pair(ϵ, i), sample(if(not(flip(3/8)), pair(ϵ, u), pair(ϵ, a))))).<br>F3(x):=λx.F2(ϵ).
Simple Elman (1990)	badigu	5d	8	10000 (1526)	-149043	1	1	F0(x):=λx.append(append(F1(ϵ), sample(if(not((x==pair(ϵ, a))), if((x==pair(ϵ, u)), append(pair(ϵ, g), x), pair(ϵ, d)), pair(ϵ, b)))), append(x, x)).<br>F1(x):=λx.if(flip(1/2), ϵ, Fm3(ϵ)).<br>F2(x):=λx.Fm0(if(flip(7/24), pair(ϵ, i), sample(if(not(flip(3/8)), pair(ϵ, u), pair(ϵ, a))))).<br>F3(x):=λx.F2(ϵ).
Simple Elman (1990)	badigu	5d	8	100000 (8441)	-1.50074e+06	1	1	F0(x):=λx.sample(if(flip(7/24), pair(append(Fm1(x), pair(head(x), a)), a), (pair(pair(pair(F1(x), d), i), i) ∪ pair(pair(append(pair(Fm1(x), g), pair(ϵ, u)), u), u)))).<br>F1(x):=λx.Fm2(append(x, pair(x, g))).<br>F2(x):=λx.if(not(flip(11/24)), Fm3(x), head(ϵ)).<br>F3(x):=λx.Fm0(pair(x, b)).
man, am, an, a, mam	man	5d	8	1 (1)	-14.7089	1	0.2	F0(x):=λx.pair(pair(pair(ϵ, m), a), n).
man, am, an, a, mam	man	5d	8	10 (5)	-45.0539	0.66666	0.8	F0(x):=λx.append(pair(if(flip(1/2), ϵ, pair(ϵ, m)), a), sample(Σ)).
man, am, an, a, mam	man	5d	8	100 (5)	-202.807	1	1	F0(x):=λx.insert(append(if(flip(3/8), pair(ϵ, m), ϵ), sample(((Σ ∪ ϵ)\pair(ϵ, a)))), pair(ϵ, a)).
man, am, an, a, mam	man	5d	8	1000 (5)	-1667.43	1	1	F0(x):=λx.if(flip(5/24), pair(ϵ, a), append(pair(if(flip(1/2), ϵ, pair(ϵ, m)), a), sample((Σ\pair(ϵ, a))))).
man, am, an, a, mam	man	5d	8	10000 (5)	-16285.6	1	1	F0(x):=λx.if(and(flip(1/2), flip(3/8)), pair(ϵ, a), append(pair(if(flip(1/2), pair(ϵ, m), ϵ), a), sample((Σ\pair(ϵ, a))))).
man, am, an, a, mam	man	5d	8	100000 (5)	-162466	1	1	F0(x):=λx.if(or(flip(1/12), flip(1/8)), pair(ϵ, a), append(pair(if(flip(1/2), pair(ϵ, m), ϵ), a), sample((Σ\pair(x, a))))).
man, am, an, a, mam	man	5d	8	1 (1)	-21.3335	1	0.2	F0(x):=λx.ϵ.<br>F1(x):=λx.pair(pair(pair(Fm0(ϵ), m), a), n).

man, am, an, a, mam	man	5d	8	10 (5)	-51.6786	0.66666	0.8	$F0(x)$:=λx.ε.<br>$F1(x)$:=λx.append(pair(if(flip(1/2), pair(F0(ε), m), ε), a), sample(Σ)).
man, am, an, a, mam	man	5d	8	100 (5)	-204.393	1	1	$F0(x)$:=λx.append(pair(x, a), sample((x∪(Σ\pair(ε, a))))).<br>$F1(x)$:=λx.F0(if(flip(1/4), pair(ε, m), ε)).
man, am, an, a, mam	man	5d	8	1000 (5)	-1670.43	1	1	$F0(x)$:=λx.append(pair(x, a), sample(((Σ\pair(ε, a))∪x))).<br>$F1(x)$:=λx.Fm0(if(flip(3/8), pair(ε, m), ε)).
man, am, an, a, mam	man	5d	8	10000 (5)	-16290.5	1	1	$F0(x)$:=λx.if(and(flip(1/2), flip(3/8)), x, append(if(flip(1/2), append(pair(ε, m), x), x), sample((Σ\x)))).<br>$F1(x)$:=λx.Fm0(pair(ε, a)).
man, am, an, a, mam	man	5d	8	100000 (5)	-162469	1	1	$F0(x)$:=λx.append(pair(x, a), sample(((Σ∪x)\pair(ε, a)))).<br>$F1(x)$:=λx.F0(if(or(flip(1/24), flip(3/8)), pair(ε, m), ε)).
man, am, an, a, mam	man	5d	8	1 (1)	-28.769	1	0.2	$F0(x)$:=λx.ε.<br>$F1(x)$:=λx.ε.<br>$F2(x)$:=λx.pair(pair(pair(Fm1(Fm0(ε)), m), a), n).
man, am, an, a, mam	man	5d	8	10 (5)	-59.1141	0.66666	0.8	$F0(x)$:=λx.ε.<br>$F1(x)$:=λx.pair(if(flip(1/2), pair(ε, m), Fm0(ε)), a).<br>$F2(x)$:=λx.append(Fm1(ε), sample(Σ)).
man, am, an, a, mam	man	5d	8	100 (5)	-211.828	1	1	$F0(x)$:=λx.append(pair(x, a), sample(((Σ∪x)\pair(Fm1(ε), a)))).<br>$F1(x)$:=λx.ε.<br>$F2(x)$:=λx.Fm0(if(flip(1/4), pair(ε, m), ε)).
man, am, an, a, mam	man	5d	8	1000 (5)	-1677.87	1	1	$F0(x)$:=λx.ε.<br>$F1(x)$:=λx.append(pair(x, a), sample((x∪(Σ\pair(F0(ε), a))))).<br>$F2(x)$:=λx.Fm1(if(flip(3/8), pair(ε, m), ε)).
man, am, an, a, mam	man	5d	8	10000 (5)	-16296.9	1	1	$F0(x)$:=λx.if(and(flip(1/2), flip(3/8)), x, append(F1(ε), sample((Σ\x)))).<br>$F1(x)$:=λx.pair(if(flip(1/2), ε, pair(ε, m)), a).<br>$F2(x)$:=λx.F0(pair(ε, a)).
man, am, an, a, mam	man	5d	8	100000 (5)	-162477	1	1	$F0(x)$:=λx.if(or(flip(1/12), flip(1/8)), x, append(Fm1(ε), sample((Σ\x)))).<br>$F1(x)$:=λx.pair(if(flip(1/2), pair(ε, m), ε), a).<br>$F2(x)$:=λx.Fm0(pair(ε, a)).
man, am, an, a, mam	man	5d	8	1 (1)	-36.6622	1	0.2	$F0(x)$:=λx.pair(pair(ε, m), a).<br>$F1(x)$:=λx.ε.<br>$F2(x)$:=λx.Fm1(ε).<br>$F3(x)$:=λx.pair(Fm0(Fm2(ε)), n).
man, am, an, a, mam	man	5d	8	10 (5)	-67.0073	0.66666	0.8	$F0(x)$:=λx.append(Fm1(ε), sample(Σ)).<br>$F1(x)$:=λx.pair(if(flip(1/2), pair(Fm2(ε), m), ε), a).<br>$F2(x)$:=λx.ε.<br>$F3(x)$:=λx.F0(ε).
man, am, an, a, mam	man	5d	8	100 (5)	-219.721	1	1	$F0(x)$:=λx.append(pair(x, a), sample(((Σ∪x)\pair(Fm2(ε), a)))).<br>$F1(x)$:=λx.ε.<br>$F2(x)$:=λx.ε.<br>$F3(x)$:=λx.Fm0(if(flip(1/4), pair(ε, m), F1(ε))).
man, am, an, a, mam	man	5d	8	1000 (5)	-1685.76	1	1	$F0(x)$:=λx.append(pair(x, a), sample((x∪(Σ\pair(F1(ε), a))))).<br>$F1(x)$:=λx.ε.<br>$F2(x)$:=λx.pair(ε, m).<br>$F3(x)$:=λx.Fm0(if(flip(3/8), Fm2(ε), ε)).
man, am, an, a, mam	man	5d	8	10000 (5)	-16304.8	1	1	$F0(x)$:=λx.if(and(flip(1/2), flip(3/8)), x, append(pair(Fm1(F2(ε)), a), sample((Σ\x)))).<br>$F1(x)$:=λx.if(flip(1/2), ε, pair(ε, m)).<br>$F2(x)$:=λx.ε.<br>$F3(x)$:=λx.Fm0(pair(ε, a)).
man, am, an, a, mam	man	5d	8	100000 (5)	-162486	1	1	$F0(x)$:=λx.append(pair(x, a), sample(((Σ∪x)\pair(F2(ε), a)))).<br>$F1(x)$:=λx.if(flip(if(flip(5/24), 1/2, 3/8)), pair(ε, m), ε).<br>$F2(x)$:=λx.ε.<br>$F3(x)$:=λx.F0(F1(ε)).
Gomez (2002) 1b for $n = 12$	abcde12345	5d	8	1 (1)	-20.2464	1	0.04166	$F0(x)$:=λx.pair(pair(pair(ε, b), 4), e).
Gomez (2002) 1b for $n = 12$	abcde12345	5d	8	10 (8)	-77.3511	0.63157	1	$F0(x)$:=λx.insert(if(flip(1/2), pair(pair(ε, b), e), pair(pair(ε, a), d)), sample(Σ)).
Gomez (2002) 1b for $n = 12$	abcde12345	5d	8	100 (23)	-406.542	0.63157	1	$F0(x)$:=λx.insert(if(flip(1/2), pair(pair(ε, b), e), pair(pair(ε, a), d)), sample(Σ)).
Gomez (2002) 1b for $n = 12$	abcde12345	5d	8	1000 (24)	-3310.85	1	1	$F0(x)$:=λx.insert(if(flip(1/2), pair(pair(ε, a), d), pair(pair(ε, b), e)), sample(((((((Σ\(pair(ε, y)∪pair(ε, d)))\pair(ε, a))\pair(ε, z))\pair(ε, b))\pair(ε, c))\pair(ε, e)))).
Gomez (2002) 1b for $n = 12$	abcde12345	5d	8	10000 (24)	-32094.2	1	1	$F0(x)$:=λx.insert(if(flip(1/2), pair(pair(ε, a), d), pair(pair(ε, b), e)), sample(((((((Σ\(pair(ε, y)∪pair(ε, d)))\pair(ε, a))\pair(ε, z))\pair(ε, b))\pair(ε, c))\pair(ε, e)))).

Gomez (2002) 1b for $n = 12$	abcde12345	5d	8	100000 (24)	-319928	1	1	F0(x):=λx.insert(if(flip(1/2), pair(pair(ϵ, a), d), pair(pair(ϵ, b), e)), sample((((((Σ\(pair(x, y)∪pair(ϵ, d)))\pair(ϵ, a))\pair(ϵ, z))\pair(ϵ, b))\pair(ϵ, c))\pair(ϵ, e)))).
Gomez (2002) 1b for $n = 12$	abcde12345	5d	8	1 (1)	-26.871	1	0.04166	F0(x):=λx.ϵ.<br>F1(x):=λx.pair(pair(pair(Fm0(ϵ), b), 4), e).
Gomez (2002) 1b for $n = 12$	abcde12345	5d	8	10 (8)	-83.9757	0.63157	1	F0(x):=λx.pair(pair(ϵ, b), e).<br>F1(x):=λx.insert(if(flip(1/2), pair(pair(ϵ, a), d), Fm0(ϵ)), sample(Σ)).
Gomez (2002) 1b for $n = 12$	abcde12345	5d	8	100 (23)	-413.167	0.63157	1	F0(x):=λx.ϵ.<br>F1(x):=λx.insert(if(flip(1/2), pair(pair(F0(ϵ), b), e), pair(pair(ϵ, a), d)), sample(Σ)).
Gomez (2002) 1b for $n = 12$	abcde12345	5d	8	1000 (24)	-3315.27	1	1	F0(x):=λx.insert(sample((pair(x, e)∪pair(pair(ϵ, a), d))), sample(((Σ\x)\(((pair(ϵ, a)∪(pair(ϵ, y)∪pair(ϵ, e)))∪(pair(ϵ, c)∪pair(ϵ, d)))∪pair(ϵ, z))))).<br>F1(x):=λx.F0(pair(ϵ, b)).
Gomez (2002) 1b for $n = 12$	abcde12345	5d	8	10000 (24)	-32098.6	1	1	F0(x):=λx.insert(sample((pair(x, e)∪pair(pair(ϵ, a), d))), sample(((Σ\x)\(((pair(ϵ, a)∪(pair(ϵ, y)∪pair(ϵ, e)))∪(pair(ϵ, c)∪pair(ϵ, d)))∪pair(ϵ, z))))).<br>F1(x):=λx.F0(pair(ϵ, b)).
Gomez (2002) 1b for $n = 12$	abcde12345	5d	8	100000 (24)	-319932	1	1	F0(x):=λx.insert(sample((pair(x, e)∪pair(pair(ϵ, a), d))), sample(((Σ\x)\(((pair(ϵ, a)∪(pair(ϵ, y)∪pair(ϵ, e)))∪(pair(ϵ, c)∪pair(ϵ, d)))∪pair(ϵ, z))))).<br>F1(x):=λx.F0(pair(ϵ, b)).
Gomez (2002) 1b for $n = 12$	abcde12345	5d	8	1 (1)	-34.3066	1	0.04166	F0(x):=λx.pair(ϵ, b).<br>F1(x):=λx.pair(pair(F0(ϵ), 4), e).<br>F2(x):=λx.Fm1(ϵ).
Gomez (2002) 1b for $n = 12$	abcde12345	5d	8	10 (8)	-91.4113	0.63157	1	F0(x):=λx.sample(Σ).<br>F1(x):=λx.insert(if(flip(1/2), pair(pair(ϵ, a), d), pair(pair(ϵ, b), e)), Fm0(ϵ)).<br>F2(x):=λx.Fm1(ϵ).
Gomez (2002) 1b for $n = 12$	abcde12345	5d	8	100 (23)	-420.603	0.63157	1	F0(x):=λx.sample(Σ).<br>F1(x):=λx.insert(if(flip(1/2), pair(pair(ϵ, a), d), pair(pair(ϵ, b), e)), Fm0(ϵ)).<br>F2(x):=λx.Fm1(ϵ).
Gomez (2002) 1b for $n = 12$	abcde12345	5d	8	1000 (24)	-3325.6	1	1	F0(x):=λx.if(flip(1/2), pair(pair(ϵ, a), d), pair(pair(ϵ, b), e)).<br>F1(x):=λx.sample((((((Σ\pair(ϵ, a))\pair(ϵ, z))\pair(ϵ, c))\(pair(ϵ, e)∪pair(ϵ, y)))\pair(ϵ, d))\x)).<br>F2(x):=λx.insert(F0(ϵ), F1(pair(ϵ, b))).
Gomez (2002) 1b for $n = 12$	abcde12345	5d	8	10000 (24)	-32108.9	1	1	F0(x):=λx.if(flip(1/2), pair(pair(ϵ, a), d), pair(pair(ϵ, b), e)).<br>F1(x):=λx.sample((((((Σ\pair(ϵ, a))\pair(ϵ, z))\pair(ϵ, c))\(pair(ϵ, e)∪pair(ϵ, y)))\pair(ϵ, d))\x)).<br>F2(x):=λx.insert(F0(ϵ), F1(pair(ϵ, b))).
Gomez (2002) 1b for $n = 12$	abcde12345	5d	8	100000 (24)	-319942	1	1	F0(x):=λx.if(flip(1/2), pair(pair(ϵ, a), d), pair(pair(ϵ, b), e)).<br>F1(x):=λx.sample((((((Σ\pair(ϵ, a))\pair(ϵ, z))\pair(ϵ, c))\(pair(ϵ, e)∪pair(ϵ, y)))\pair(ϵ, d))\x)).<br>F2(x):=λx.insert(F0(ϵ), F1(pair(ϵ, b))).
Gomez (2002) 1b for $n = 12$	abcde12345	5d	8	1 (1)	-42.1997	1	0.04166	F0(x):=λx.pair(ϵ, b).<br>F1(x):=λx.pair(Fm0(ϵ), 4).<br>F2(x):=λx.pair(Fm1(ϵ), e).<br>F3(x):=λx.Fm2(ϵ).
Gomez (2002) 1b for $n = 12$	abcde12345	5d	8	10 (8)	-99.3044	0.63157	1	F0(x):=λx.ϵ.<br>F1(x):=λx.insert(if(flip(1/2), pair(pair(Fm2(ϵ), a), d), pair(pair(ϵ, b), e)), sample(Σ)).<br>F2(x):=λx.F0(ϵ).<br>F3(x):=λx.F1(ϵ).
Gomez (2002) 1b for $n = 12$	abcde12345	5d	8	100 (23)	-428.496	0.63157	1	F0(x):=λx.insert(if(flip(1/2), pair(pair(F1(ϵ), a), d), pair(Fm2(ϵ), e)), sample(Σ)).<br>F1(x):=λx.ϵ.<br>F2(x):=λx.pair(ϵ, b).<br>F3(x):=λx.Fm0(ϵ).
Gomez (2002) 1b for $n = 12$	abcde12345	5d	8	1000 (24)	-3336.95	1	1	F0(x):=λx.ϵ.<br>F1(x):=λx.pair(ϵ, a).<br>F2(x):=λx.if(flip(1/2), append(pair(F0(ϵ), b), pair(x, e)), pair(append(Fm1(ϵ), x), d)).<br>F3(x):=λx.Fm2(sample((((((Σ\pair(ϵ, b))\pair(ϵ, y))\pair(ϵ, z))\pair(ϵ, c))\(pair(ϵ, e)∪F1(x)))\pair(ϵ, d)))).
Gomez (2002) 1b for $n = 12$	abcde12345	5d	8	10000 (24)	-32116.8	1	1	F0(x):=λx.ϵ.<br>F1(x):=λx.ϵ.<br>F2(x):=λx.sample((((((Σ\x)\pair(ϵ, c))\pair(ϵ, a))\(pair(ϵ, e)∪pair(ϵ, y)))\(pair(ϵ, b)∪pair(Fm1(ϵ), z)))).<br>F3(x):=λx.insert(if(flip(1/2), pair(pair(ϵ, b), e), pair(pair(F0(ϵ), a), d)), F2(pair(ϵ, d))).
Gomez (2002) 1b for $n = 12$	abcde12345	5d	8	100000 (24)	-319949	1	1	F0(x):=λx.if(flip(1/2), x, pair(pair(ϵ, a), d)).<br>F1(x):=λx.insert(F0(x), sample(((Σ\(pair(ϵ, a)∪(pair(ϵ, e)∪pair(ϵ, c))))\(pair(ϵ, z)∪((pair(Fm2(ϵ), d)∪head(x))∪pair(ϵ, y)))))).<br>F2(x):=λx.x.<br>F3(x):=λx.F1(pair(pair(ϵ, b), e)).
Reeder et al. (2013)	aAsbBnxXc	5d	8	1 (1)	-24.7502	1	0	F0(x):=λx.pair(pair(pair(pair(ϵ, q), A), x), B).
Reeder et al. (2013)	aAsbBnxXc	5d	8	10 (9)	-172.323	0.14705	0.48	F0(x):=λx.append(append(sample((ϵ∪if(flip(1/2), pair(pair(ϵ, Q), A), Σ))), if(flip(1/2), sample(Σ), pair(if(flip(1/3), pair(ϵ, a), pair(ϵ, s)), X))), if(flip(1/2), pair(ϵ, B), pair(ϵ, n))).

Reeder et al. (2013)	aAsbBnxXc	5d	8	100 (71)	-1139.76	0.16666	0.88	F0(x):=λx.append(sample(((pair(ϵ, Q) ∪ ϵ) ∪ pair(ϵ, q))), append(if(not(flip(1/2)), pair(ϵ, s), if(flip(1/2), pair(ϵ, a), pair(ϵ, A))), append(sample((Σ\pair(ϵ, s))), if(flip(1/2), if(not(flip(1/2)), pair(ϵ, B), pair(ϵ, b)), pair(ϵ, n))))).
Reeder et al. (2013)	aAsbBnxXc	5d	8	1000 (160)	-11029.4	0.16666	0.88	F0(x):=λx.append(sample(((pair(ϵ, Q) ∪ ϵ) ∪ pair(ϵ, q))), append(if(not(flip(1/2)), pair(ϵ, s), if(flip(1/2), pair(ϵ, a), pair(ϵ, A))), append(sample((Σ\pair(ϵ, s))), if(flip(1/2), if(not(flip(1/2)), pair(ϵ, B), pair(ϵ, b)), pair(ϵ, n))))).
Reeder et al. (2013)	aAsbBnxXc	5d	8	10000 (162)	-106717	0.16666	0.88	F0(x):=λx.append(sample(((pair(ϵ, Q) ∪ ϵ) ∪ pair(x, q))), append(if(not(flip(1/2)), pair(ϵ, s), if(flip(1/2), pair(ϵ, a), pair(ϵ, A))), append(sample((Σ\pair(ϵ, s))), if(flip(1/2), if(not(flip(1/2)), pair(ϵ, B), pair(ϵ, b)), pair(ϵ, n))))).
Reeder et al. (2013)	aAsbBnxXc	5d	8	100000 (162)	-1.07064e+06	0.16666	0.88	F0(x):=λx.append(sample(((pair(ϵ, Q) ∪ x) ∪ pair(ϵ, q))), append(if(not(flip(1/2)), pair(ϵ, s), if(flip(1/2), pair(ϵ, a), pair(x, A))), append(sample((Σ\pair(ϵ, n))), if(flip(1/2), if(not(flip(1/2)), pair(x, B), pair(ϵ, b)), pair(ϵ, n))))).
Reeder et al. (2013)	aAsbBnxXc	5d	8	1 (1)	-31.3748	1	0	F0(x):=λx.pair(pair(ϵ, q), A).<br>F1(x):=λx.pair(pair(F0(ϵ), x), B).
Reeder et al. (2013)	aAsbBnxXc	5d	8	10 (9)	-180.272	0.06153	0.16	F0(x):=λx.ϵ.<br>F1(x):=λx.append(if(flip(1/2), if(flip(1/2), pair(ϵ, q), ϵ), pair(F0(ϵ), Q)), insert(if(flip(1/2), pair(if(flip(1/2), pair(ϵ, s), pair(ϵ, A)), n), pair(ϵ, X)), sample(Σ))).
Reeder et al. (2013)	aAsbBnxXc	5d	8	100 (71)	-1040.33	0.22222	0.92	F0(x):=λx.insert(sample(((Σ\pair(ϵ, R))\(x ∪ (pair(ϵ, n) ∪ pair(ϵ, q))))), append(if(flip(1/4), pair(ϵ, q), if(flip(1/2), ϵ, pair(ϵ, Q))), x)).<br>F1(x):=λx.append(F0(if(flip(1/2), if(flip(1/2), pair(ϵ, A), pair(ϵ, s)), pair(ϵ, a))), if(flip(1/3), pair(ϵ, B), if(flip(1/2), pair(ϵ, n), pair(ϵ, b)))).
Reeder et al. (2013)	aAsbBnxXc	5d	8	1000 (160)	-9335.2	0.22222	0.92	F0(x):=λx.insert(sample(((Σ\pair(ϵ, n))\(x ∪ (pair(ϵ, B) ∪ pair(ϵ, q))))), append(if(flip(1/4), pair(ϵ, q), if(not(flip(1/3)), ϵ, pair(ϵ, Q))), x)).<br>F1(x):=λx.append(F0(if(not(flip(3/8)), if(flip(1/2), pair(ϵ, A), pair(ϵ, s)), pair(ϵ, a))), if(flip(1/3), pair(x, B), if(flip(1/2), pair(x, n), head(pair(ϵ, b))))).
Reeder et al. (2013)	aAsbBnxXc	5d	8	10000 (162)	-86400.5	0.33333	0.92	F0(x):=λx.if(and(empty(x), flip(1/2)), Fm0(pair(x, a)), append(head(pair(if(not(flip(1/3)), x, pair(ϵ, A)), s)), if(flip(1/3), pair(ϵ, x), if(flip(1/2), pair(ϵ, X), pair(ϵ, c))))).<br>F1(x):=λx.append(append(sample(if(flip(1/2), (pair(ϵ, q) ∪ pair(ϵ, Q)), ϵ)), F0(ϵ)), sample((((Σ\pair(ϵ, Q))\((pair(ϵ, q) ∪ pair(ϵ, a)) ∪ pair(ϵ, x)))\(pair(ϵ, A) ∪ (pair(x, c) ∪ pair(ϵ, R)))))).
Reeder et al. (2013)	aAsbBnxXc	5d	8	100000 (162)	-864115	0.33333	0.92	F0(x):=λx.if(and(empty(x), flip(1/2)), Fm0(pair(x, a)), append(head(pair(if(not(flip(1/3)), x, pair(ϵ, A)), s)), if(flip(1/3), pair(ϵ, x), if(flip(1/2), pair(ϵ, X), pair(ϵ, c))))).<br>F1(x):=λx.append(append(sample(if(flip(1/2), (pair(ϵ, q) ∪ pair(ϵ, Q)), ϵ)), F0(x)), sample((((Σ\pair(ϵ, Q))\((pair(ϵ, q) ∪ pair(ϵ, a)) ∪ pair(ϵ, r)))\(pair(ϵ, A) ∪ (pair(x, c) ∪ pair(ϵ, R)))))).
Reeder et al. (2013)	aAsbBnxXc	5d	8	1 (1)	-38.8103	1	0	F0(x):=λx.pair(F1(ϵ), B).<br>F1(x):=λx.pair(pair(pair(ϵ, q), A), x).<br>F2(x):=λx.Fm0(ϵ).
Reeder et al. (2013)	aAsbBnxXc	5d	8	10 (9)	-194.718	0.07692	0.24	F0(x):=λx.append(sample(Σ), if(flip(7/24), ϵ, x)).<br>F1(x):=λx.F0(pair(if(flip(1/6), pair(pair(ϵ, A), c), Fm0(pair(ϵ, X))), n)).<br>F2(x):=λx.F1(ϵ).
Reeder et al. (2013)	aAsbBnxXc	5d	8	100 (71)	-1028.16	0.65384	0.92	F0(x):=λx.sample(((pair(x, A) ∪ pair(x, a)) ∪ pair(x, s))).<br>F1(x):=λx.append(F0(if(flip(11/24), tail(ϵ), if(flip(1/2), x, pair(ϵ, q)))), sample(((if(flip(7/24), pair(ϵ, X), Σ)\pair(ϵ, B))\(pair(ϵ, n) ∪ x)))).<br>F2(x):=λx.append(F1(pair(ϵ, Q)), if(not(flip(1/4)), if(flip(1/2), pair(ϵ, n), pair(ϵ, B)), pair(ϵ, b))).
Reeder et al. (2013)	aAsbBnxXc	5d	8	1000 (160)	-8327.97	0.69047	0.92	F0(x):=λx.if(flip(1/3), F1(x), append(head(F1(ϵ)), sample(if(flip(1/2), pair(ϵ, n), pair(ϵ, B))))).<br>F1(x):=λx.pair(if(not(flip(1/3)), if(flip(1/2), pair(x, X), pair(ϵ, c)), pair(ϵ, x)), b).<br>F2(x):=λx.append(if(flip(5/12), if(not(flip(5/12)), pair(sample((pair(ϵ, q) ∪ x)), A), pair(head(F2(pair(ϵ, q))), a)), sample(if(not(flip(5/24)), if(flip(1/2), pair(if(flip(7/24), pair(ϵ, q), x), s), if(flip(1/2), pair(pair(ϵ, Q), A), pair(pair(ϵ, Q), s))), pair(x, a)))), Fm0(ϵ)).
Reeder et al. (2013)	aAsbBnxXc	5d	8	10000 (162)	-71065.5	0.66666	0.96	F0(x):=λx.append(if(flip(1/3), pair(if(flip(1/2), x, ϵ), a), if(flip(1/2), pair(if(flip(1/2), ϵ, x), A), pair(if(flip(1/2), x, ϵ), s))), append(if(flip(1/3), pair(ϵ, x), if(flip(1/2), pair(ϵ, X), pair(ϵ, c))), Fm1(pair(ϵ, b)))).<br>F1(x):=λx.sample((if(flip(1/3), pair(if(flip(1/2), pair(ϵ, n), x), r), pair(ϵ, B)) ∪ if(flip(1/2), x, pair(ϵ, n)))).<br>F2(x):=λx.Fm0(if(flip(1/2), pair(ϵ, Q), pair(ϵ, q))).
Reeder et al. (2013)	aAsbBnxXc	5d	8	100000 (162)	-712583	0.66666	0.96	F0(x):=λx.append(if(flip(1/3), pair(if(flip(1/2), x, ϵ), a), if(flip(1/2), pair(if(flip(1/2), ϵ, x), A), pair(if(flip(1/2), x, ϵ), s))), append(if(flip(1/3), pair(ϵ, x), if(flip(1/2), pair(ϵ, X), pair(ϵ, c))), Fm1(pair(ϵ, b)))).<br>F1(x):=λx.sample((if(flip(1/3), pair(if(flip(1/2), pair(ϵ, n), x), r), pair(ϵ, B)) ∪ if(flip(1/2), x, pair(ϵ, n)))).<br>F2(x):=λx.Fm0(if(flip(1/2), pair(ϵ, Q), pair(ϵ, q))).
Reeder et al. (2013)	aAsbBnxXc	5d	8	1 (1)	-46.7035	1	0	F0(x):=λx.pair(pair(pair(Fm1(F2(ϵ)), q), A), x).<br>F1(x):=λx.ϵ.<br>F2(x):=λx.ϵ.<br>F3(x):=λx.pair(Fm0(ϵ), B).
Reeder et al. (2013)	aAsbBnxXc	5d	8	10 (9)	-194.404	0.15384	0.64	F0(x):=λx.if(flip(1/2), pair(x, X), if(flip(1/2), pair(append(x, ϵ), x), pair(x, c))).<br>F1(x):=λx.Fm0(F2(pair(ϵ, Q))).<br>F2(x):=λx.append(if(flip(1/2), if(flip(1/2), pair(ϵ, q), ϵ), x), sample(Σ)).<br>F3(x):=λx.append(F1(ϵ), if(flip(1/2), pair(ϵ, B), pair(ϵ, n))).
Reeder et al. (2013)	aAsbBnxXc	5d	8	100 (71)	-992.503	0.64516	0.92	F0(x):=λx.sample(if(not(flip(7/24)), if(flip(1/2), pair(if(flip(1/2), ϵ, head(x)), a), x), pair(head(if(flip(1/2), append(x, ϵ), ϵ)), A))).<br>F1(x):=λx.if(flip(1/4), append(x, pair(ϵ, B)), if(flip(1/2), pair(x, n), pair(x, b))).<br>F2(x):=λx.if(flip(11/24), pair(Fm0(x), X), if(flip(1/2), pair(F0(x), c), pair(F0(x), x))).<br>F3(x):=λx.Fm1(Fm2(if(flip(1/3), pair(ϵ, s), pair(sample(if(flip(1/2), pair(x, Q), pair(ϵ, q))), s)))).
Reeder et al. (2013)	aAsbBnxXc	5d	8	1000 (160)	-8437.54	0.68	0.92	F0(x):=λx.sample(if(not(flip(7/24)), if(flip(1/2), pair(if(flip(1/2), ϵ, head(x)), a), sample(x)), pair(head(if(flip(1/2), x, ϵ)), A))).<br>F1(x):=λx.if(flip(3/8), append(x, pair(ϵ, B)), if(flip(1/2), pair(append(ϵ, x), n), pair(x, b))).<br>F2(x):=λx.if(flip(3/8), pair(Fm0(x), X), if(flip(1/2), pair(F0(x), c), pair(F0(x), x))).<br>F3(x):=λx.Fm1(Fm2(if(flip(7/24), head(pair(x, s)), pair(sample(if(flip(1/2), pair(ϵ, Q), pair(ϵ, q))), s)))).

Reeder et al. (2013)	aAsbBnxXc	5d	8	10000 (162)	-80971.7	0.625	0.92	F0(x):=λx.sample(if(not(flip(1/3)), if(flip(1/2), pair(if(flip(1/2), ε, append(ε, head(x))), a), x), pair(head(if(flip(11/24), x, ε)), A))).<br>F1(x):=λx.if(flip(1/3), append(x, pair(ε, B)), if(flip(1/2), pair(x, n), append(x, pair(ε, b)))).<br>F2(x):=λx.if(flip(3/8), pair(Fm0(x), X), if(flip(1/2), pair(F0(x), c), pair(F0(append(x, ε)), x))).<br>F3(x):=λx.Fm1(Fm2(if(flip(7/24), pair(ε, s), pair(sample(if(flip(1/2), pair(ε, Q), pair(x, q))), s)))).
Reeder et al. (2013)	aAsbBnxXc	5d	8	100000 (162)	-809889	0.66666	0.92	F0(x):=λx.sample(if(or(flip(3/8), flip(1/2)), if(flip(1/2), pair(if(flip(1/2), ε, head(x)), a), append(x, ε)), pair(if(flip(1/2), head(append(ε, x)), ε), A))).<br>F1(x):=λx.if(flip(1/3), append(x, pair(ε, B)), if(flip(1/2), pair(x, n), append(x, pair(ε, b)))).<br>F2(x):=λx.if(flip(1/3), pair(Fm0(x), X), if(flip(1/2), pair(F0(sample(x)), c), pair(F0(x), x))).<br>F3(x):=λx.Fm1(Fm2(if(or(flip(1/8), flip(5/24)), pair(ε, s), pair(sample(if(flip(1/2), pair(x, Q), pair(x, q))), s)))).
Reber (1967)	PSTVX	7d	8	1 (1)	-25.6149	1	0.04	F0(x):=λx.pair(pair(pair(pair(pair(ε, V), X), V), P), S).
Reber (1967)	PSTVX	7d	8	10 (8)	-217.104	0.44	0.24	F0(x):=λx.sample(if(flip(1/2), append(pair(F0(pair(head(x), P)), X), if(flip(1/2), append(pair(ε, V), head(pair(x, S))), pair(ε, X))), (append(pair(ε, T), pair(if(flip(1/6), pair(x, P), ε), T)) ∪ pair(ε, V)))).
Reber (1967)	PSTVX	7d	8	100 (45)	-1300.91	0.32142	0.48	F0(x):=λx.sample(if(not(flip(1/4)), if(flip(5/12), append(pair(if(not(flip(11/24)), pair(Fm0(pair(x, P)), X), pair(ε, V)), V), head(pair(x, S))), pair(F0(pair(x, P)), X)), ((pair(pair(append(pair(ε, T), x), P), T) ∪ pair(pair(ε, T), T)) ∪ pair(ε, V)))).
Reber (1967)	PSTVX	7d	8	1000 (165)	-13132.3	0.32142	0.48	F0(x):=λx.sample(if(not(flip(7/24)), if(flip(3/8), append(pair(if(not(flip(11/24)), pair(Fm0(pair(x, P)), X), pair(ε, V)), V), head(pair(x, S))), pair(F0(pair(x, P)), X)), ((pair(pair(append(pair(ε, T), x), P), T) ∪ pair(pair(ε, T), T)) ∪ pair(ε, V)))).
Reber (1967)	PSTVX	7d	8	10000 (660)	-133443	0.32142	0.48	F0(x):=λx.sample(if(not(flip(7/24)), if(flip(3/8), append(pair(if(not(flip(11/24)), pair(Fm0(pair(x, P)), X), pair(ε, V)), V), head(pair(x, S))), pair(F0(pair(x, P)), X)), ((pair(pair(append(pair(ε, T), x), P), T) ∪ pair(pair(ε, T), T)) ∪ pair(ε, V)))).
Reber (1967)	PSTVX	7d	8	100000 (2414)	-1.33386e+06	0.32142	0.48	F0(x):=λx.sample(if(not(flip(7/24)), if(flip(3/8), append(pair(if(not(flip(11/24)), pair(Fm0(pair(x, P)), X), pair(append(ε, ε), V)), V), head(pair(x, S))), pair(F0(pair(x, P)), X)), ((pair(pair(append(pair(ε, T), x), P), T) ∪ pair(pair(ε, T), T)) ∪ pair(ε, V)))).
Reber (1967)	PSTVX	7d	8	1 (1)	-32.2395	1	0.04	F0(x):=λx.pair(ε, V).<br>F1(x):=λx.pair(pair(pair(pair(Fm0(ε), X), V), P), S).
Reber (1967)	PSTVX	7d	8	10 (8)	-248.44	0.08888	0.36	F0(x):=λx.sample(Σ).<br>F1(x):=λx.if(flip(7/24), append(Fm0(ε), if(flip(1/3), pair(pair(x, V), P), head(pair(ε, S)))), append(Fm0(x), Fm1(pair(ε, X)))).
Reber (1967)	PSTVX	7d	8	100 (45)	-1049.69	0.5	1	F0(x):=λx.if(flip(1/4), pair(append(if(flip(1/2), ε, pair(pair(sample((ε ∪ pair(pair(ε, P), P))), P), P)), if((x==head(x)), ε, pair(ε, P))), T), append(sample(if(not(flip(1/3)), pair(Fm0(if(flip(7/24), pair(pair(ε, V), P), ε)), X), ε)), x)).<br>F1(x):=λx.pair(append(sample((pair(ε, V) ∪ pair(ε, T))), F0(sample(if(flip(1/4), pair(pair(sample(ε), V), P), pair(ε, V)))))), S).
Reber (1967)	PSTVX	7d	8	1000 (165)	-7750.95	0.96	1	F0(x):=λx.append(x, if(flip(1/8), Fm0(insert(pair(ε, P), pair(ε, V))), sample(if(or((x==head(x)), not(flip(1/2))), if(not(flip(1/2)), pair(pair(if(flip(1/12), ε, pair(ε, X)), V), S), F0(pair(ε, X))), pair(ε, S))))).<br>F1(x):=λx.F0(if(not(flip(3/8)), pair(x, V), pair(if(flip(5/12), pair(x, T), append(pair(ε, T), append(pair(if(flip(7/24), append(if(flip(1/2), pair(x, P), x), pair(ε, P)), ε), P), sample((pair(pair(pair(x, P), P), P) ∪ ε)))))), T))).
Reber (1967)	PSTVX	7d	8	10000 (660)	-67401.8	0.88	1	F0(x):=λx.if(flip(1/4), pair(ε, V), pair(if(not(flip(3/8)), Fm0(x), if(not(flip(1/3)), x, pair(pair(F0(x), V), P))), X)).<br>F1(x):=λx.if(flip(if(empty(x), 1/2, 3/8)), append(pair(F0(pair(head(pair(x, T)), T)), V), pair(if(flip(1/6), pair(ε, P), ε), S)), append(head(pair(x, T)), if(flip(1/2), Fm1(pair(head(x), P)), pair(pair(if(or(flip(7/24), flip(1/2)), x, pair(x, P)), T), S)))).
Reber (1967)	PSTVX	7d	8	100000 (2414)	-678870	0.88	1	F0(x):=λx.if(flip(1/4), pair(ε, V), pair(if(not(flip(3/8)), Fm0(x), if(not(flip(1/3)), x, pair(pair(F0(x), V), P))), X)).<br>F1(x):=λx.if(flip(if((x==ε), 1/2, 3/8)), append(pair(F0(pair(head(pair(x, T)), T)), V), pair(if(flip(1/6), pair(ε, P), ε), S)), append(head(pair(x, T)), if(flip(1/2), Fm1(pair(head(x), P)), pair(pair(if(or(flip(1/4), flip(1/2)), x, pair(x, P)), T), S)))).
Reber (1967)	PSTVX	7d	8	1 (1)	-39.675	1	0.04	F0(x):=λx.pair(pair(pair(ε, V), X), V).<br>F1(x):=λx.pair(pair(F0(ε), P), S).<br>F2(x):=λx.F1(ε).
Reber (1967)	PSTVX	7d	8	10 (8)	-226.631	0.57692	0.8	F0(x):=λx.append(pair(ε, T), pair(if(flip(1/2), pair(if(flip(11/24), pair(x, P), ε), P), ε), T)).<br>F1(x):=λx.append(if(flip(1/2), pair(Fm1(if((head(x)==x), ε, if(flip(1/2), x, ε))), X), sample((F0(pair(ε, P)) ∪ pair(ε, V)))), x).<br>F2(x):=λx.pair(Fm1(if(flip(1/2), pair(ε, V), if(flip(1/2), pair(pair(ε, V), P), ε))), S).
Reber (1967)	PSTVX	7d	8	100 (45)	-1244.02	0.33333	0.56	F0(x):=λx.F1(insert(sample(((Σ\(pair(ε, X) ∪ pair(x, T)))\pair(ε, V))), pair(ε, V))).<br>F1(x):=λx.append(if(not(flip(3/8)), pair(if(flip(if(not(empty(x)), 1/12, 1/2)), Fm2(ε), F1(ε)), X), pair(ε, V)), x).<br>F2(x):=λx.if(not(flip(7/24)), Fm0(ε), pair(if(flip(1/2), append(pair(pair(ε, T), P), sample(if(flip(1/3), ε, pair(if(flip(1/2), insert(pair(ε, P), sample(if(flip(3/8), ε, pair(ε, P)))), ε), P)))), pair(ε, T)), T)).
Reber (1967)	PSTVX	7d	8	1000 (165)	-8089.86	0.72	1	F0(x):=λx.sample(if(not(flip(1/4)), append(if(flip(1/2), pair(Fm0(x), X), x), if(flip(5/24), pair(x, P), ε)), F1(F1(ε)))).<br>F1(x):=λx.append(if(or(empty(x), not(flip(1/12))), pair(ε, T), pair(pair(x, P), P)), append(if(flip(1/3), pair(if(or(empty(x), flip(1/2)), pair(ε, P), ε), P), ε), head(pair(x, P)))).<br>F2(x):=λx.append(Fm0(pair(ε, V)), if(flip(1/2), pair(pair(if(flip(1/8), x, pair(ε, X)), V), S), pair(ε, S))).
Reber (1967)	PSTVX	7d	8	10000 (660)	-78613.8	0.72	1	F0(x):=λx.sample(if(not(flip(1/4)), append(if(flip(1/2), pair(Fm0(x), X), x), if(flip(5/24), pair(x, P), ε)), F1(F1(ε)))).<br>F1(x):=λx.append(if(or(empty(x), not(flip(1/12))), pair(ε, T), pair(pair(x, P), P)), append(if(flip(1/3), pair(if(or(empty(x), flip(1/2)), pair(ε, P), ε), P), ε), head(pair(x, P)))).<br>F2(x):=λx.append(Fm0(pair(ε, V)), if(flip(1/2), pair(pair(if(flip(1/8), x, pair(ε, X)), V), S), pair(ε, S))).
Reber (1967)	PSTVX	7d	8	100000 (2414)	-789218	0.72	1	F0(x):=λx.sample(if(not(flip(1/4)), append(if(flip(1/2), pair(Fm0(x), X), x), if(flip(5/24), pair(x, P), ε)), F1(F1(ε)))).<br>F1(x):=λx.append(if(or((x==ε), not(flip(1/12))), pair(ε, T), pair(pair(x, P), P)), append(if(flip(1/3), pair(if(or(empty(x), flip(1/2)), pair(ε, P), ε), P), ε), head(pair(x, P)))).<br>F2(x):=λx.append(Fm0(pair(ε, V)), if(flip(1/2), pair(pair(if(flip(1/8), ε, pair(ε, X)), V), S), pair(ε, S))).
Reber (1967)	PSTVX	7d	8	1 (1)	-47.5682	1	0.04	F0(x):=λx.pair(pair(pair(pair(pair(F1(Fm2(ε)), V), X), V), P), S).<br>F1(x):=λx.ε.<br>F2(x):=λx.ε.<br>F3(x):=λx.F0(ε).

Reber (1967)	PSTVX	7d	8	10 (8)	-240.347	0.61538	0.76	F0(x):=λx.if(not(flip(1/4)), append(pair(F2(if(flip(1/24), ϵ, x)), X), x), pair(F1(pair(ϵ, T)), T)).<br>F1(x):=λx.if(flip(1/2), x, pair(x, P)).<br>F2(x):=λx.if(flip(7/24), append(pair(ϵ, V), x), F0(x)).<br>F3(x):=λx.pair(F2(Fm1(pair(ϵ, V))), S).
Reber (1967)	PSTVX	7d	8	100 (45)	-1102.41	0.69230	1	F0(x):=λx.Fm3(append(append(x, Fm2(pair(x, P))), if(flip(1/2), pair(Fm1(x), V), x))).<br>F1(x):=λx.append(if(flip(1/2), Fm1(x), if((pair(ϵ, T)==x), x, ϵ)), pair(ϵ, X)).<br>F2(x):=λx.if(not(flip(1/3)), if(flip(1/24), append(if(flip(11/24), ϵ, F1(ϵ)), append(pair(x, X), if(flip(1/2), x, ϵ))), ϵ), pair(Fm2(ϵ), P)).<br>F3(x):=λx.append(x, if(not(empty(x)), pair(if(flip(1/3), pair(ϵ, P), ϵ), S), F0(sample(((Σ\(pair(ϵ, P)∪pair(ϵ, S)))\pair(x, X)))))).
Reber (1967)	PSTVX	7d	8	1000 (165)	-8026.11	0.73076	1	F0(x):=λx.Fm3(append(append(x, Fm2(pair(x, P))), if(not(flip(1/2)), pair(Fm1(x), V), x))).<br>F1(x):=λx.append(if(flip(1/2), Fm1(x), if((pair(ϵ, T)==x), x, ϵ)), pair(ϵ, X)).<br>F2(x):=λx.if(not(flip(1/3)), if(flip(1/24), append(if(flip(1/2), ϵ, F1(x)), append(pair(x, X), append(ϵ, if(flip(1/3), x, ϵ)))), ϵ), pair(Fm2(ϵ), P)).<br>F3(x):=λx.append(x, if(not((ϵ==x)), pair(if(flip(1/4), pair(ϵ, P), ϵ), S), F0(sample(((Σ\(pair(ϵ, P)∪pair(x, S)))\pair(ϵ, X)))))).
Reber (1967)	PSTVX	7d	8	10000 (660)	-61429.4	1	1	F0(x):=λx.if(not(flip(1/2)), pair(append(x, F0(ϵ)), P), x).<br>F1(x):=λx.sample(if(not(flip(1/4)), append(if(flip(1/4), pair(ϵ, V), pair(F1(ϵ), X)), if(not(flip(1/6)), x, Fm2(append(pair(ϵ, V), pair(ϵ, P))))), pair(Fm0(pair(ϵ, T)), T))).<br>F2(x):=λx.x.<br>F3(x):=λx.append(F1(pair(ϵ, V)), pair(ϵ, S)).
Reber (1967)	PSTVX	7d	8	100000 (2414)	-612690	1	1	F0(x):=λx.if(not(flip(1/2)), pair(append(x, F0(ϵ)), P), x).<br>F1(x):=λx.sample(if(not(flip(1/4)), append(if(flip(1/4), pair(ϵ, V), pair(F1(ϵ), X)), if(not(flip(1/6)), x, Fm2(append(pair(ϵ, V), pair(ϵ, P))))), pair(Fm0(pair(ϵ, T)), T))).<br>F2(x):=λx.x.<br>F3(x):=λx.append(F1(pair(ϵ, V)), pair(ϵ, S)).
Berwick & Pilato (1987)	JgGdDeiW	7d	8	1 (1)	-26.6685	1	0.04	F0(x):=λx.pair(pair(pair(pair(ϵ, J), i), v), o).
Berwick & Pilato (1987)	JgGdDeiW	7d	8	10 (8)	-124.031	0.09523	0.68	F0(x):=λx.append(pair(ϵ, J), append(sample(Σ), pair(if(flip(1/2), ϵ, if(flip(1/2), pair(ϵ, v), if(flip(1/2), pair(ϵ, V), pair(sample(if(flip(1/2), Σ, ϵ)), N)))), o))).
Berwick & Pilato (1987)	JgGdDeiW	7d	8	100 (24)	-861.893	0.14285	0.76	F0(x):=λx.append(pair(ϵ, J), append(sample(Σ), pair(if(flip(3/8), pair(if(flip(1/8), pair(ϵ, b), if(flip(5/12), pair(sample(Σ), b), if(flip(7/24), pair(ϵ, B), ϵ))), N), if(flip(1/2), ϵ, if(not(flip(5/24)), if(flip(1/2), pair(ϵ, V), pair(pair(ϵ, e), N)), pair(ϵ, v)))), o))).
Berwick & Pilato (1987)	JgGdDeiW	7d	8	1000 (36)	-9282.36	0.19047	0.88	F0(x):=λx.append(pair(ϵ, J), sample(if(not(flip(1/12)), append(sample(Σ), pair(if(flip(3/8), pair(if(flip(11/24), sample(Σ), ϵ), N), sample(if(flip(if(flip(5/24), 1/2, 1/2)), if(flip(11/24), (if(flip(7/24), ϵ, pair(ϵ, v))∪ϵ), pair(pair(ϵ, B), v)), pair(ϵ, V)))), o)), (Σ∪ϵ)))).
Berwick & Pilato (1987)	JgGdDeiW	7d	8	10000 (36)	-90026.4	0.19047	0.88	F0(x):=λx.append(pair(ϵ, J), sample(if(not(flip(1/12)), append(sample(Σ), pair(if(flip(3/8), pair(if(flip(11/24), sample(Σ), ϵ), N), sample(if(flip(if(flip(5/24), 1/2, 1/2)), if(flip(11/24), (if(flip(7/24), ϵ, pair(ϵ, v))∪ϵ), pair(pair(ϵ, B), v)), pair(ϵ, V)))), o)), (Σ∪ϵ)))).
Berwick & Pilato (1987)	JgGdDeiW	7d	8	100000 (36)	-911152	0.19047	0.88	F0(x):=λx.append(pair(ϵ, J), sample(if(not(flip(1/12)), append(sample(Σ), pair(if(flip(3/8), pair(if(flip(11/24), sample(Σ), ϵ), N), sample(if(flip(if(flip(5/24), 1/2, 1/2)), if(flip(11/24), (if(flip(7/24), ϵ, pair(ϵ, v))∪ϵ), pair(pair(ϵ, B), v)), pair(ϵ, V)))), o)), (Σ∪ϵ)))).
Berwick & Pilato (1987)	JgGdDeiW	7d	8	1 (1)	-33.2931	1	0.04	F0(x):=λx.pair(pair(pair(ϵ, J), i), v).<br>F1(x):=λx.pair(Fm0(ϵ), o).
Berwick & Pilato (1987)	JgGdDeiW	7d	8	10 (8)	-137.819	0.05468	0.48	F0(x):=λx.append(pair(ϵ, J), append(if(flip(1/2), ϵ, sample(Σ)), x)).<br>F1(x):=λx.pair(F0(sample((pair(pair(ϵ, E), N)∪Σ))), o).
Berwick & Pilato (1987)	JgGdDeiW	7d	8	100 (24)	-747.032	0.14285	0.92	F0(x):=λx.if(flip(1/2), ϵ, pair(if(flip(7/24), pair(ϵ, B), if(flip(1/2), pair(ϵ, E), ϵ)), b)).<br>F1(x):=λx.append(pair(ϵ, J), pair(append(sample(Σ), if(flip(1/8), pair(pair(ϵ, e), N), if(flip(1/6), pair(head(Fm0(ϵ)), v), if(flip(1/2), if(flip(1/2), ϵ, pair(ϵ, V)), pair(if(not(flip(1/4)), F0(x), pair(ϵ, B)), N))))), o)).
Berwick & Pilato (1987)	JgGdDeiW	7d	8	1000 (36)	-6510.6	0.22222	1	F0(x):=λx.if(flip(1/6), pair(pair(ϵ, e), N), if(flip(1/2), if(not(flip(3/8)), pair(if(flip(1/2), pair(if(flip(1/2), ϵ, pair(x, j)), B), if(flip(1/2), pair(ϵ, E), ϵ)), v), pair(pair(if(or(flip(7/24), flip(1/12)), pair(ϵ, B), ϵ), b), N)), pair(ϵ, V))).<br>F1(x):=λx.if(not(empty(x)), append(pair(ϵ, J), append(sample((((Σ\pair(ϵ, e))\pair(ϵ, W))\(pair(ϵ, N)∪(pair(ϵ, J)∪head(x))))), if(flip(7/24), pair(if(empty(if((x==head(x)), x, ϵ)), head(x), ϵ), N), if(flip(1/4), ϵ, x)))), pair(Fm1(F0(ϵ)), o)).
Berwick & Pilato (1987)	JgGdDeiW	7d	8	10000 (36)	-64082.5	0.15789	0.96	F0(x):=λx.if(flip(1/4), if(not(flip(7/24)), pair(if(not(flip(1/3)), head(ϵ), pair(ϵ, B)), b), pair(ϵ, j)), if(flip(7/24), pair(x, e), sample((if(flip(5/24), pair(x, B), ϵ)∪if(not(flip(1/3)), if(flip(1/4), pair(pair(ϵ, E), b), ϵ), pair(x, E)))))).<br>F1(x):=λx.append(append(pair(x, J), sample((Σ\(pair(ϵ, W)∪pair(ϵ, o))))), if(flip(11/24), pair(if(flip(3/8), pair(ϵ, V), if(flip(1/3), pair(if(flip(1/3), pair(ϵ, B), ϵ), v), ϵ)), o), if(not(flip(1/12)), pair(pair(F0(ϵ), N), o), pair(pair(ϵ, j), B)))).
Berwick & Pilato (1987)	JgGdDeiW	7d	8	100000 (36)	-639335	0.15789	0.96	F0(x):=λx.if(flip(1/4), if(or(flip(1/2), flip(1/2)), pair(if(not(flip(3/8)), x, pair(ϵ, B)), b), pair(ϵ, j)), if(flip(7/24), pair(ϵ, e), sample((if(flip(1/4), pair(ϵ, B), x)∪if(not(flip(1/3)), if(flip(1/4), pair(pair(x, E), b), ϵ), pair(ϵ, E)))))).<br>F1(x):=λx.append(append(pair(ϵ, J), sample(((Σ\ϵ)\(pair(ϵ, v)∪pair(ϵ, e))))), if(flip(11/24), pair(if(flip(1/3), pair(ϵ, V), if(flip(1/3), pair(if(flip(1/3), pair(ϵ, B), ϵ), v), x)), o), if(not(flip(1/12)), pair(pair(Fm0(ϵ), N), o), pair(pair(ϵ, j), B)))).
Berwick & Pilato (1987)	JgGdDeiW	7d	8	1 (1)	-40.7286	1	0.04	F0(x):=λx.pair(pair(F1(ϵ), i), v).<br>F1(x):=λx.pair(ϵ, J).<br>F2(x):=λx.pair(F0(ϵ), o).
Berwick & Pilato (1987)	JgGdDeiW	7d	8	10 (8)	-141.03	0.14285	0.6	F0(x):=λx.pair(if(flip(1/2), x, pair(x, N)), o).<br>F1(x):=λx.sample(Σ).<br>F2(x):=λx.append(pair(ϵ, J), F0(append(if(flip(7/24), if(flip(1/2), pair(ϵ, M), ϵ), if(flip(1/2), ϵ, Fm1(ϵ))), sample(Σ)))).
Berwick & Pilato (1987)	JgGdDeiW	7d	8	100 (24)	-747.481	0.09523	0.92	F0(x):=λx.if(flip(1/2), if(flip(1/2), pair(ϵ, E), pair(x, B)), ϵ).<br>F1(x):=λx.pair(ϵ, J).<br>F2(x):=λx.append(F1(x), append(sample(Σ), if(flip(1/2), pair(pair(if(flip(7/24), pair(ϵ, e), sample((pair(Fm0(ϵ), b)∪Fm0(ϵ)))), N), o), pair(if(flip(1/2), if(flip(1/2), pair(F0(x), v), pair(x, V)), ϵ), o)))).

Berwick & Pilato (1987)	JgGdDeiW	7d	8	1000 (36)	-5048.36	0.33333	1	F0(x):=λx.append(x, sample((((((((Σ\pair(ε, E))\x)\pair(ε, N))\pair(ε, W))\pair(ε, V))\pair(ε, o))\pair(tail(ε), j))\(pair(ε, v)∪(pair(ε, b)∪(pair(ε, B)∪pair(ε, e))))))).<br>F1(x):=λx.if(not(flip(1/3)), if(not(flip(5/24)), sample(if(flip(1/8), pair(x, j), x)), pair(ε, E)), pair(if(flip(7/24), pair(x, j), ε), B)).<br>F2(x):=λx.append(Fm0(pair(ε, J)), if(flip(1/2), pair(pair(if(or(flip(1/2), flip(1/2)), if(flip(3/8), pair(F1(ε), b), if(flip(5/12), ε, F1(ε))), pair(ε, e)), N), o), pair(if(flip(7/24), pair(ε, V), if(not(flip(1/3)), ε, pair(F1(ε), v))), o))).
Berwick & Pilato (1987)	JgGdDeiW	7d	8	10000 (36)	-47562.7	0.33333	1	F0(x):=λx.append(x, sample((((((((Σ\pair(ε, E))\x)\pair(ε, N))\pair(ε, W))\pair(ε, V))\pair(ε, o))\pair(ε, j))\(pair(ε, v)∪(pair(ε, b)∪(pair(ε, B)∪pair(ε, e))))))).<br>F1(x):=λx.if(not(flip(1/3)), if(not(flip(5/24)), sample(if(flip(1/8), pair(ε, j), ε)), pair(x, E)), pair(if(flip(1/4), pair(x, j), sample(x)), B)).<br>F2(x):=λx.append(Fm0(pair(ε, J)), if(flip(1/2), pair(pair(if(or(not(flip(1/2)), or(flip(1/2), flip(1/12))), if(flip(3/8), pair(F1(ε), b), if(flip(11/24), x, F1(ε))), pair(ε, e)), N), o), pair(if(flip(1/3), pair(ε, V), if(not(flip(1/3)), ε, pair(F1(x), v))), o))).
Berwick & Pilato (1987)	JgGdDeiW	7d	8	100000 (36)	-473631	0.33333	1	F0(x):=λx.append(x, sample((((((((Σ\pair(ε, E))\x)\pair(ε, N))\pair(ε, W))\pair(ε, V))\pair(ε, o))\pair(ε, j))\(pair(ε, v)∪(pair(ε, b)∪(pair(ε, B)∪pair(ε, e))))))).<br>F1(x):=λx.if(not(flip(1/3)), if(not(flip(5/24)), sample(if(flip(1/8), pair(ε, j), ε)), pair(x, E)), pair(if(flip(1/4), pair(x, j), sample(x)), B)).<br>F2(x):=λx.append(Fm0(pair(ε, J)), if(flip(1/2), pair(pair(if(or(not(flip(1/2)), or(flip(1/2), flip(1/12))), if(flip(3/8), pair(F1(ε), b), if(flip(11/24), x, F1(ε))), pair(ε, e)), N), o), pair(if(flip(1/3), pair(ε, V), if(not(flip(1/3)), ε, pair(F1(x), v))), o))).
Berwick & Pilato (1987)	JgGdDeiW	7d	8	1 (1)	-48.6218	1	0.04	F0(x):=λx.pair(pair(Fm1(Fm2(ε)), i), v).<br>F1(x):=λx.pair(ε, J).<br>F2(x):=λx.ε.<br>F3(x):=λx.pair(Fm0(ε), o).
Berwick & Pilato (1987)	JgGdDeiW	7d	8	10 (8)	-153.007	0.07142	0.44	F0(x):=λx.append(sample((Fm2(ε)∪if(flip(1/2), Σ, pair(pair(ε, i), b)))), x).<br>F1(x):=λx.pair(sample(Σ), o).<br>F2(x):=λx.ε.<br>F3(x):=λx.append(pair(ε, J), Fm0(Fm1(ε))).
Berwick & Pilato (1987)	JgGdDeiW	7d	8	100 (24)	-774.469	0.12	0.84	F0(x):=λx.if(flip(7/24), sample(if(flip(1/2), pair(if(flip(1/2), ε, pair(ε, E)), v), pair(ε, V))), if(flip(7/24), ε, pair(if(flip(1/4), if(flip(5/24), pair(ε, B), x), if(flip(1/2), ε, pair(ε, e))), N))).<br>F1(x):=λx.append(pair(ε, J), x).<br>F2(x):=λx.F0(pair(if(flip(1/2), ε, x), b)).<br>F3(x):=λx.Fm1(if(not(flip(1/12)), pair(append(sample(Σ), Fm2(pair(ε, E))), o), pair(append(pair(pair(ε, H), B), Fm2(x)), o))).
Berwick & Pilato (1987)	JgGdDeiW	7d	8	1000 (36)	-5126.32	0.30303	1	F0(x):=λx.append(F1(ε), if(and(not((x==head(x))), flip(7/24)), pair(ε, V), x)).<br>F1(x):=λx.sample((((((Σ\pair(ε, v))\(pair(ε, N)∪pair(ε, J)))\(pair(x, W)∪pair(ε, B)))\pair(ε, b))\(pair(head(ε), E)∪(pair(ε, e)∪pair(ε, o))))\append(pair(ε, j), ε))).<br>F2(x):=λx.append(pair(ε, J), pair(Fm0(sample((pair(head(x), N)∪((((pair(x, N)∪head(x))\Σ)∪pair(pair(x, b), N))∪if(flip(1/2), pair(pair(ε, e), N), pair(x, v)))))), o)).<br>F3(x):=λx.F2(if(flip(7/24), pair(if(flip(1/3), pair(ε, j), ε), B), if(not(flip(1/8)), ε, pair(x, E)))).
Berwick & Pilato (1987)	JgGdDeiW	7d	8	10000 (36)	-48456.8	0.30303	1	F0(x):=λx.append(F1(ε), if(and(not((x==head(x))), flip(7/24)), pair(ε, V), x)).<br>F1(x):=λx.sample((((((Σ\pair(ε, j))\(pair(ε, N)∪pair(ε, J)))\(pair(ε, W)∪pair(ε, B)))\pair(ε, b))\(pair(x, E)∪(pair(x, e)∪pair(ε, o))))\pair(ε, v))).<br>F2(x):=λx.append(pair(ε, J), pair(Fm0(sample((pair(head(x), N)∪((((pair(x, N)∪head(x))\Σ)∪pair(pair(x, b), N))∪if(flip(11/24), pair(pair(ε, e), N), pair(x, v)))))), o)).<br>F3(x):=λx.F2(if(flip(7/24), pair(if(flip(1/3), append(pair(sample(ε), j), x), x), B), if(not(flip(1/6)), ε, pair(ε, E)))).
Berwick & Pilato (1987)	JgGdDeiW	7d	8	100000 (36)	-482529	0.30303	1	F0(x):=λx.append(F1(ε), if(and(not((x==head(x))), flip(7/24)), pair(ε, V), x)).<br>F1(x):=λx.sample((((((Σ\pair(ε, V))\(pair(head(sample(ε)), e)∪pair(tail(x), J)))\(pair(ε, v)∪pair(ε, B)))\pair(ε, N))\(pair(ε, W)∪(pair(x, b)∪pair(ε, o))))\pair(insert(ε, ε), E))).<br>F2(x):=λx.append(pair(ε, J), pair(Fm0(sample((pair(head(x), N)∪((((pair(x, N)∪head(head(x)))\Σ)∪pair(pair(x, b), N))∪if(flip(11/24), pair(pair(ε, e), N), pair(x, v)))))), o)).<br>F3(x):=λx.F2(if(flip(7/24), pair(if(flip(3/8), append(pair(x, j), ε), sample(ε)), B), if(not(flip(1/8)), ε, pair(ε, E)))).
